# Supplementary material for: Rarγ-Foxa1 signaling promotes luminal identity in prostate progenitors and is disrupted in prostate cancer
Source: EMBO Rep. 2024 Dec 4;26(2):443–69. doi: 10.1038/s44319-024-00335-y (PMC11772605; doi:10.1038/s44319-024-00335-y)
Supplement: Supplementary file 1 — Appendix [file 44319_2024_335_MOESM1_ESM.pdf]

APPENDIX

Table of Content:

Page 2: **Appendix Figure S1.** *Gene Set Enrichment Analysis highlights the role of RA for prostate epithelium differentiation.*

Page 3: **Appendix Figure S2.** *Single cells RNA-seq analysis of RARs expression in human and mouse adult prostate cells.*

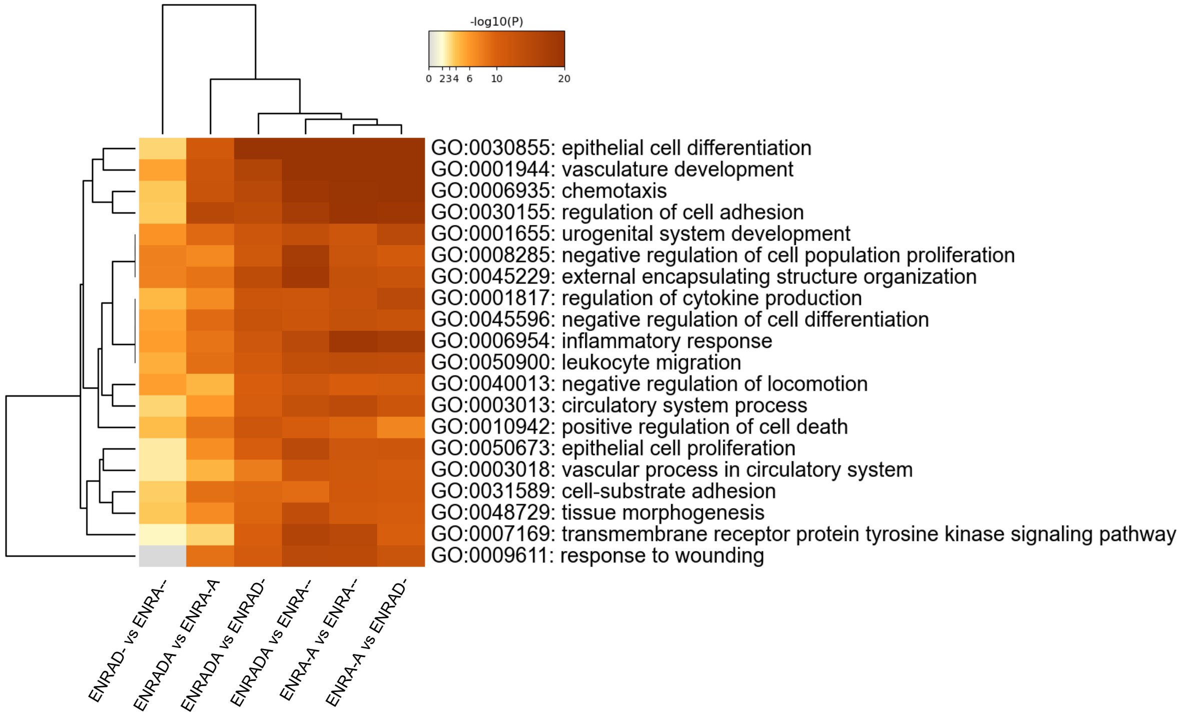

Appendix Figure S1. *Gene Set Enrichment Analysis highlights the role of RA for prostate epithelium differentiation.*

Most enriched GO terms associated with in the comparison between androgen and retinoic acid signaling in mouse prostate organoids. Metascape.

Human Prostate - Total cells in population: Basal: 1747 , Lum D: 1961, Lum P 241

Gene	Cluster	Count_exp_cells	Tot. cells	Percentage
RARa	Basal	357	1747	20.435
RARa	Lum D	241	1961	12.290
RARa	Lum P	69	241	28.631
RARb	Basal	269	1747	15.398
RARb	Lum D	33	1961	1.683
RARb	Lum P	26	241	10.788
RARg	Basal	325	1747	18.603
RARg	Lum D	77	1961	3.927
RARg	Lum P	66	241	27.386

Mouse Dorsal Prostate - Total cells in population: Basal: 140, Lum D: 780, Lum P 98

Gene	Cluster	Count_exp_cells	Tot. cells	Percentage
Rara	Basal	4	140	2.857
Rara	Lum D	115	780	14.738
Rara	Lum P	27	98	27.551
Rarb	Basal	4	140	2.857
Rarb	Lum D	2	780	0.256
Rarb	Lum P	10	98	10.204
Rarg	Basal	27	140	19.286
Rarg	Lum D	130	780	16.667
Rarg	Lum P	50	98	51.020

Mouse Anterior Prostate - Total cells in population: Basal: 804, Lum A: 544, Lum P 223, Pru: 73

Gene	Cluster	Count_exp_cells	Tot. cells	Percentage
Rara	Basal	33	804	4.104
Rara	Lum A	77	544	14.154
Rara	Lum P	45	223	20.179
Rara	Pru	5	73	6.849
Rarb	Basal	19	804	2.363
Rarb	Lum A	0	544	0
Rarb	Lum P	6	223	2.691
Rarb	Pru	1	73	1.37
Rarg	Basal	194	804	24.129
Rarg	Lum A	45	544	8.272
Rarg	Lum P	99	223	44.395
Rarg	Pru	31	73	42.466

Appendix Figure S2. *Single cells RNA-seq analysis of RARs expression in human and mouse adult prostate cells.*

Tables showing number of cells expressing the three different RARs in the Basal, Luminal Distal and Luminal Proximal clusters of cell populations in human (upper) and mouse (lower) adult prostate (Data ref: Crowley et al., 2020).
