# Supplementary figures and images for: Rarγ-Foxa1 signaling promotes luminal identity in prostate progenitors and is disrupted in prostate cancer (part 1 of 2)
Source: EMBO Rep. 2024 Dec 4;26(2):443–69. doi: 10.1038/s44319-024-00335-y (PMC11772605; doi:10.1038/s44319-024-00335-y)

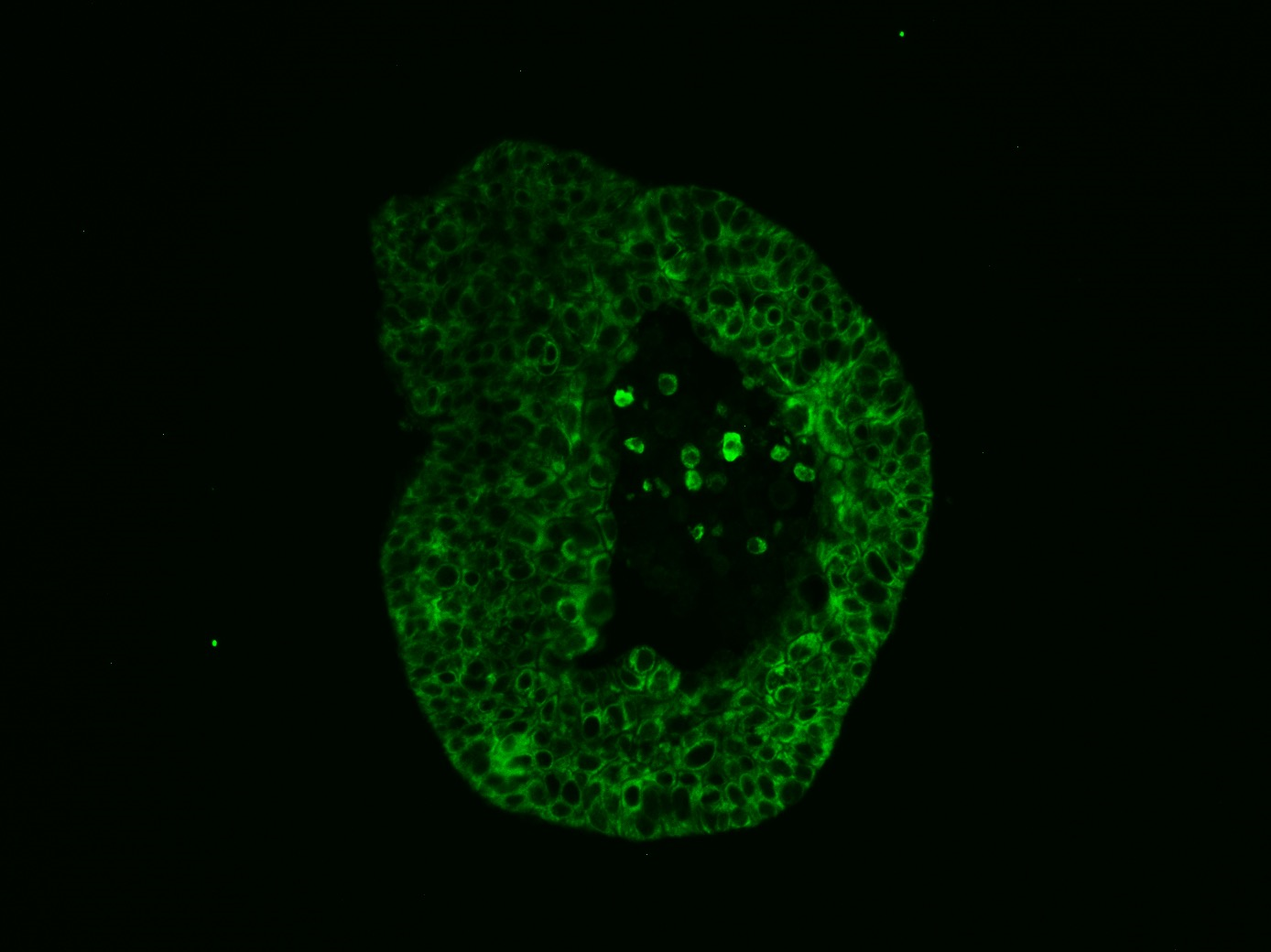

Supplement: Supplementary file 7 — Source data Fig. 1 [file 44319_2024_335_MOESM7_ESM.zip › Figure 1/1F/Ck5 ENRA-A.tif]

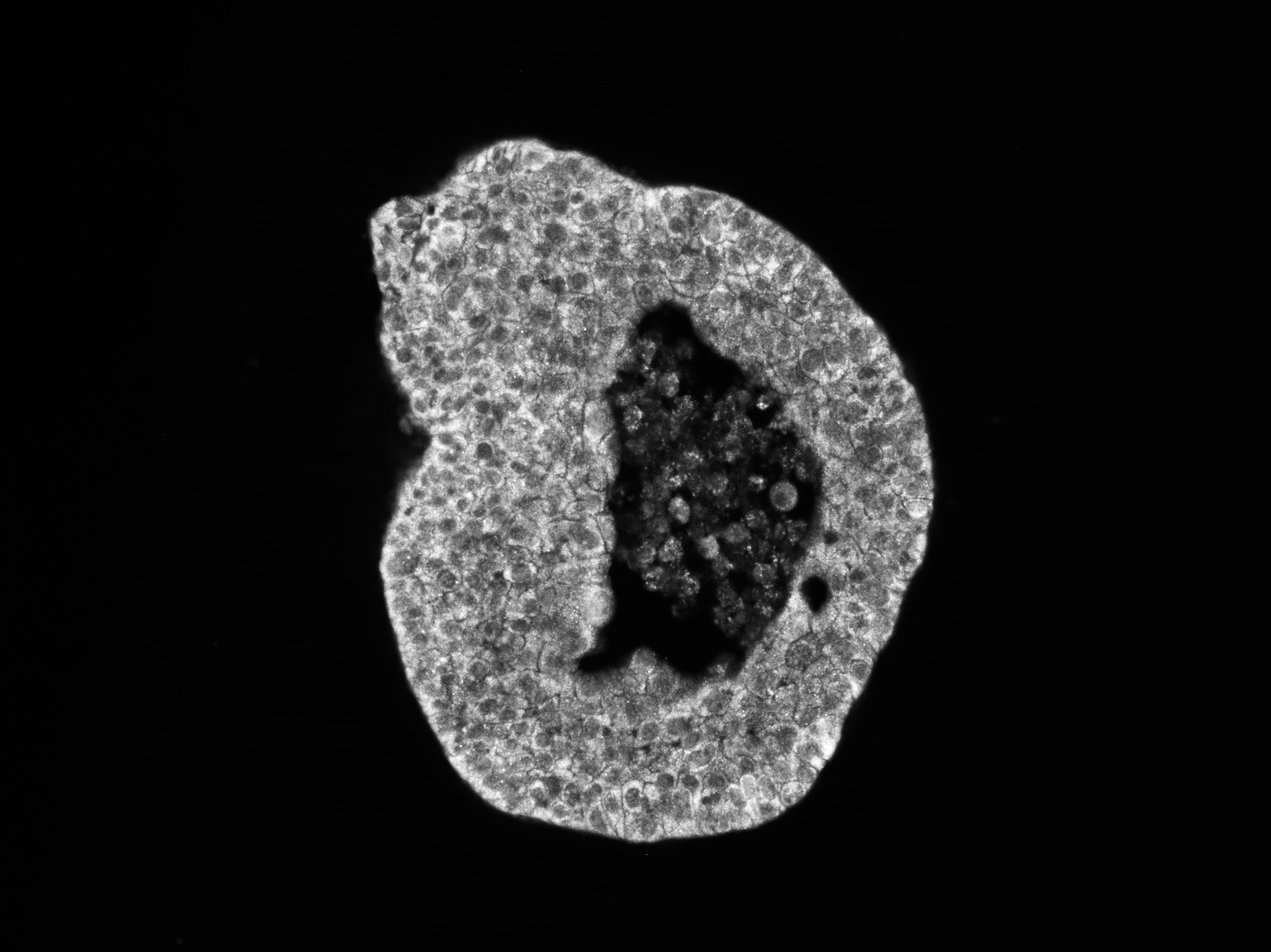

Supplement: Supplementary file 7 — Source data Fig. 1 [file 44319_2024_335_MOESM7_ESM.zip › Figure 1/1F/AR ENRA-A.tif]

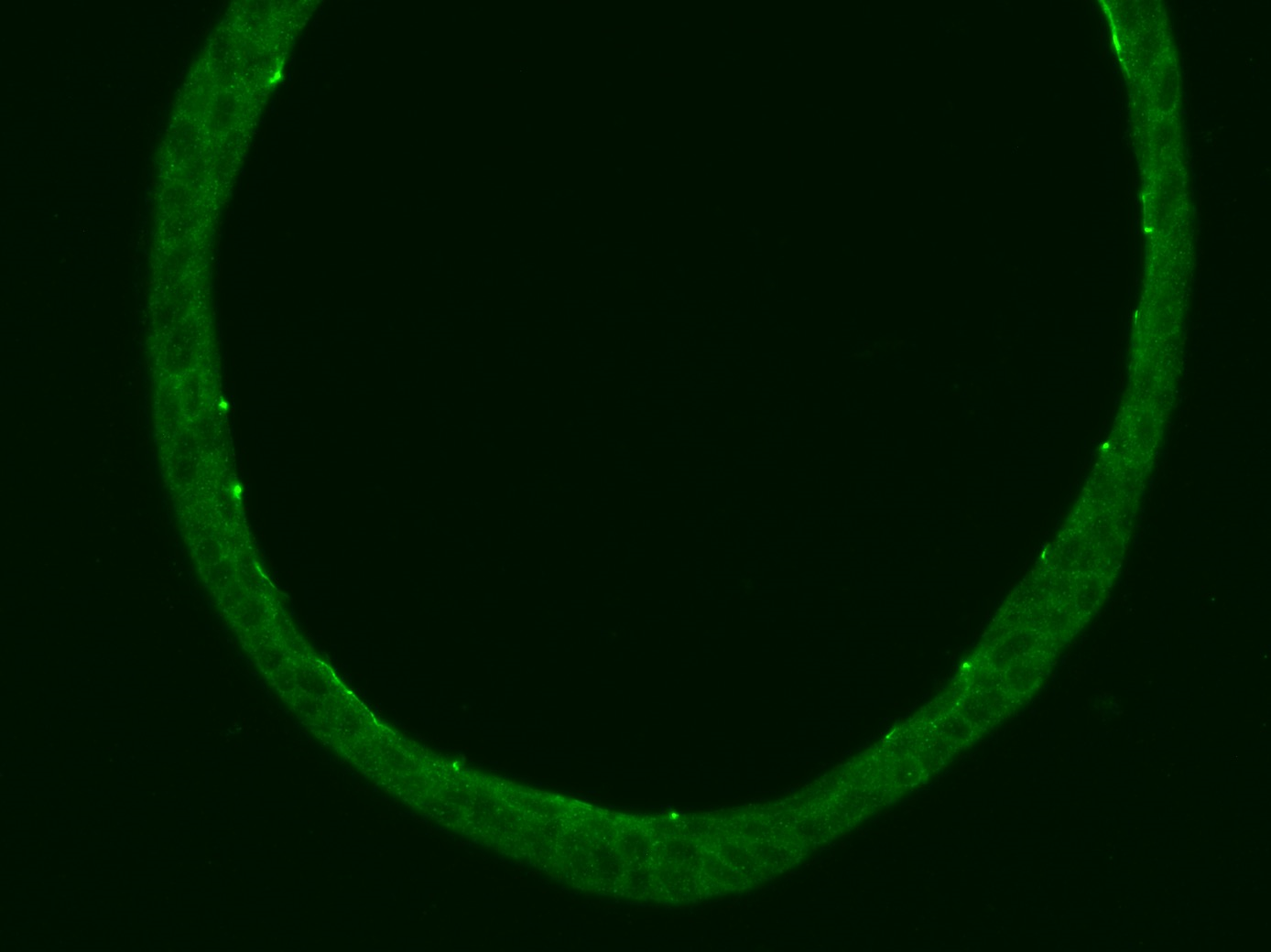

Supplement: Supplementary file 7 — Source data Fig. 1 [file 44319_2024_335_MOESM7_ESM.zip › Figure 1/1F/Zo3 ENRADA.tif]

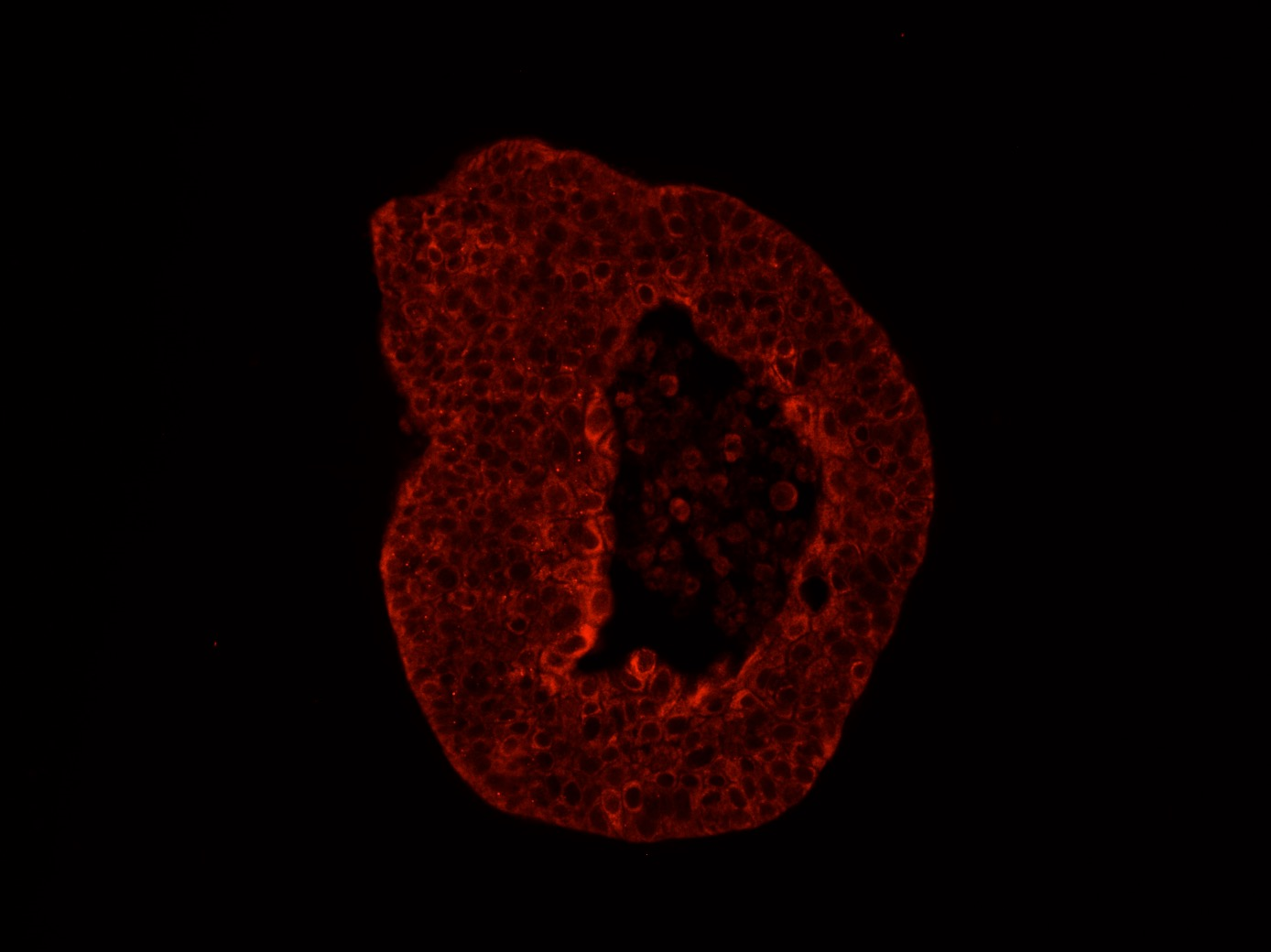

Supplement: Supplementary file 7 — Source data Fig. 1 [file 44319_2024_335_MOESM7_ESM.zip › Figure 1/1F/Ck8 ENRA-A.tif]

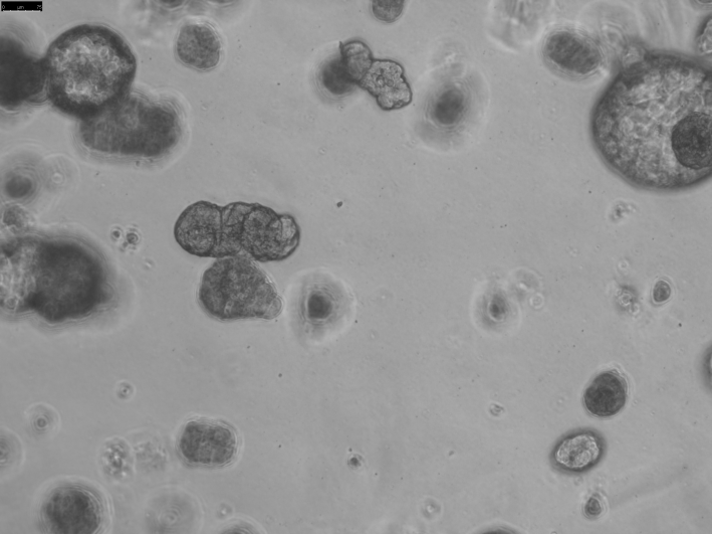

Supplement: Supplementary file 7 — Source data Fig. 1 [file 44319_2024_335_MOESM7_ESM.zip › Figure 1/1F/bf ENRA-A.tif]

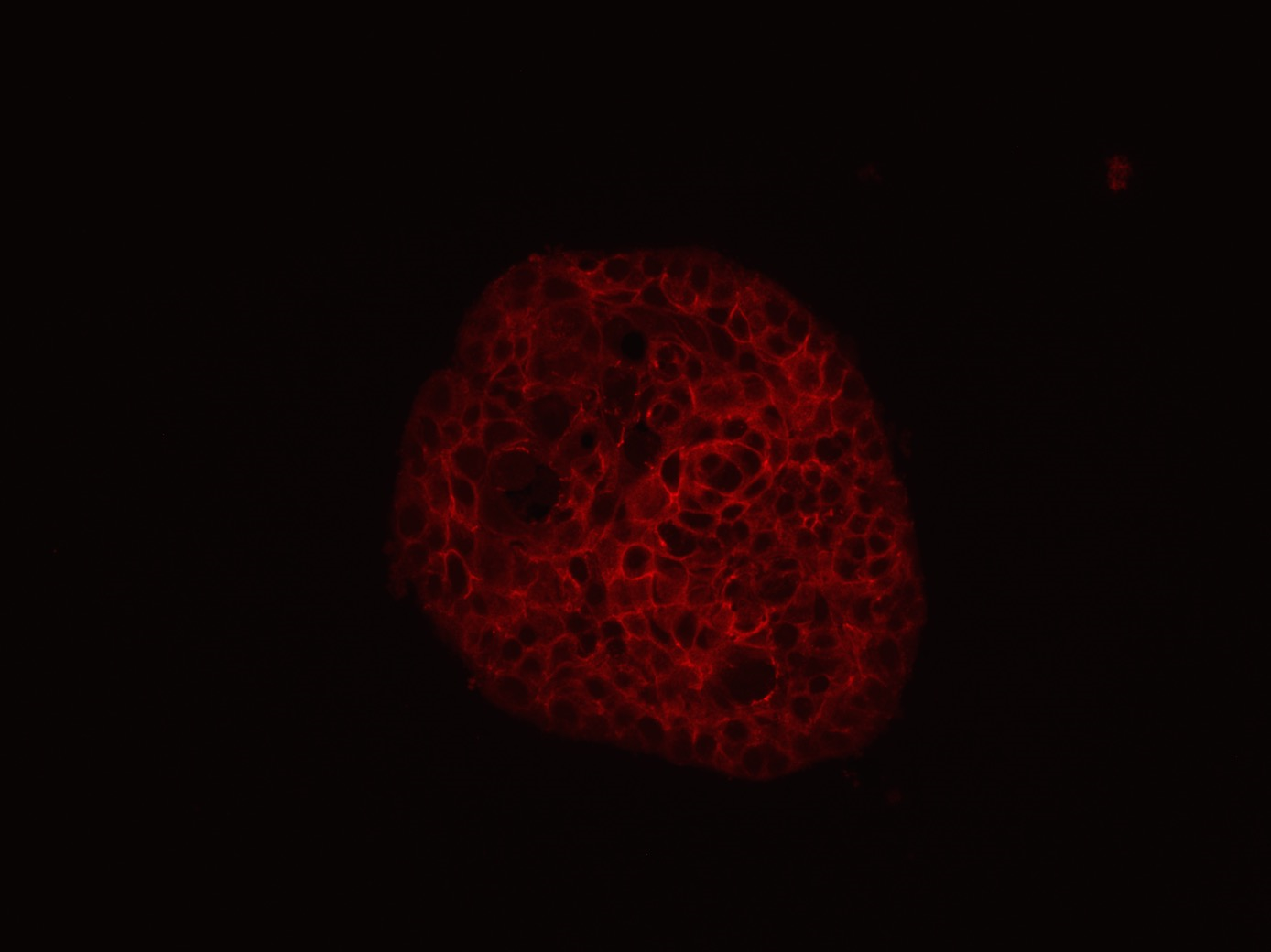

Supplement: Supplementary file 7 — Source data Fig. 1 [file 44319_2024_335_MOESM7_ESM.zip › Figure 1/1F/Zo1 ENRA-A.tif]

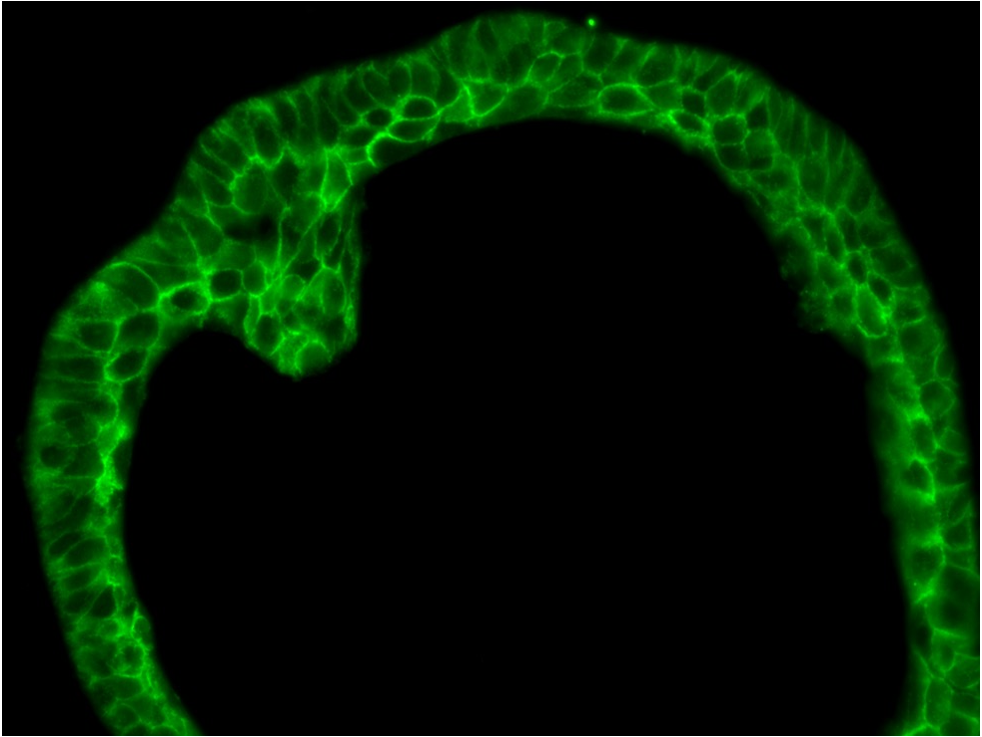

Supplement: Supplementary file 7 — Source data Fig. 1 [file 44319_2024_335_MOESM7_ESM.zip › Figure 1/1F/Cldn7 ENRADA.tif]

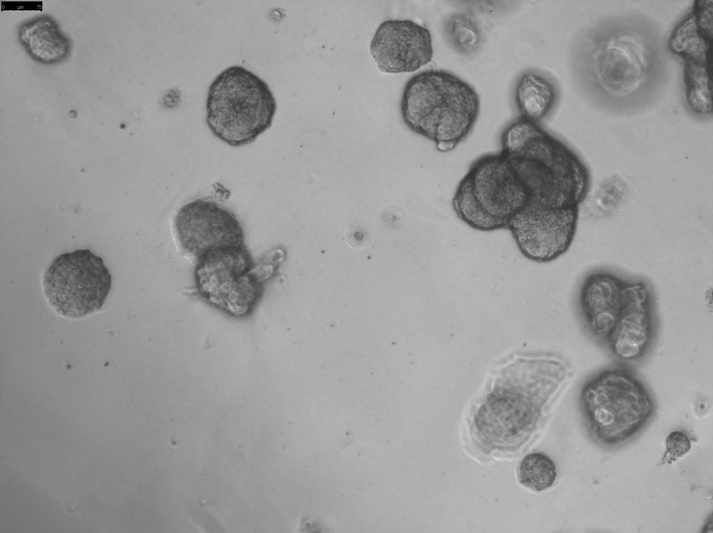

Supplement: Supplementary file 7 — Source data Fig. 1 [file 44319_2024_335_MOESM7_ESM.zip › Figure 1/1F/bf ENRA-.tif]

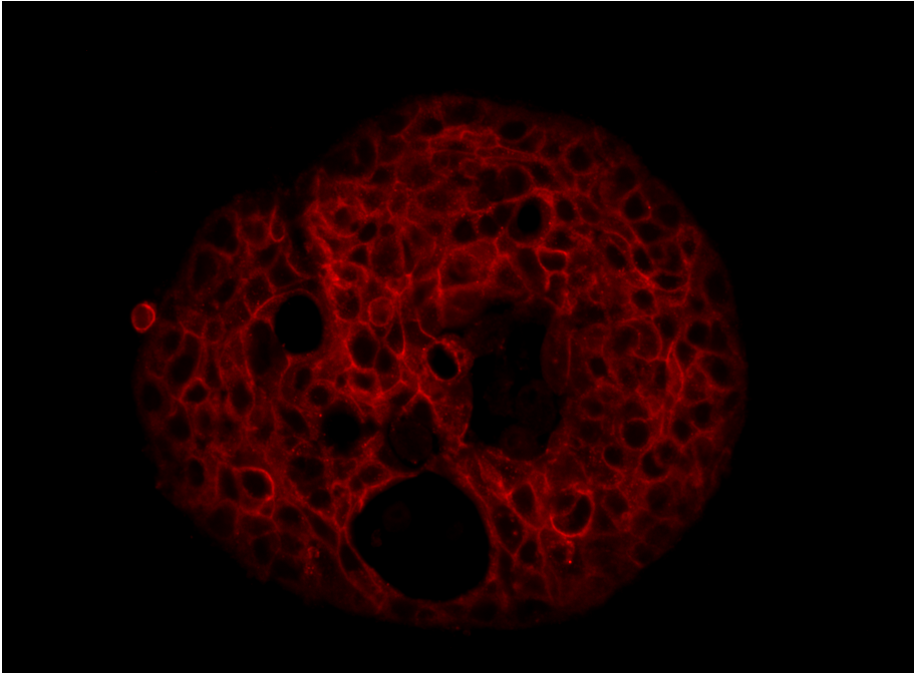

Supplement: Supplementary file 7 — Source data Fig. 1 [file 44319_2024_335_MOESM7_ESM.zip › Figure 1/1F/Cldn4 ENRAD-.tif]

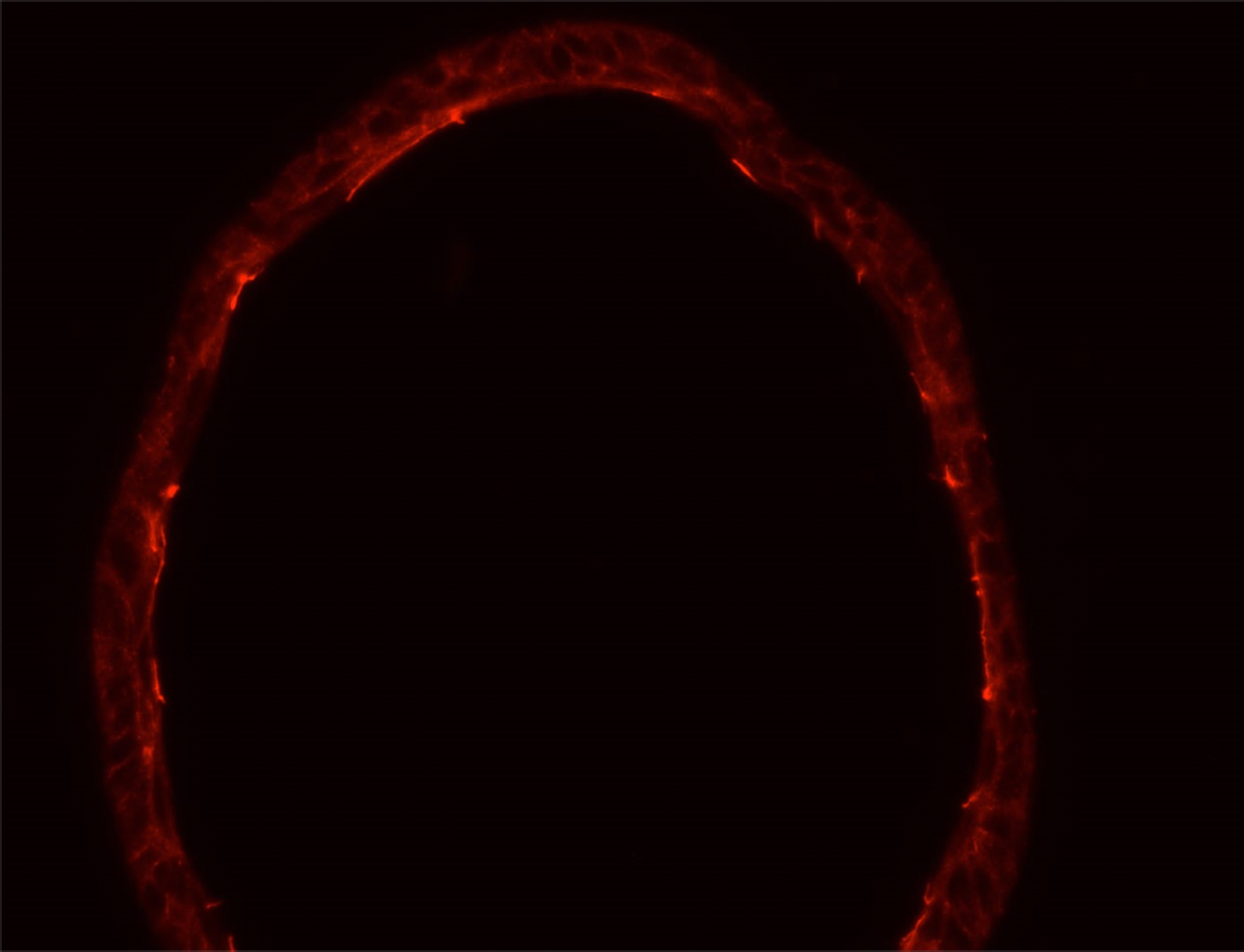

Supplement: Supplementary file 7 — Source data Fig. 1 [file 44319_2024_335_MOESM7_ESM.zip › Figure 1/1F/Zo1 ENRADA.tif]

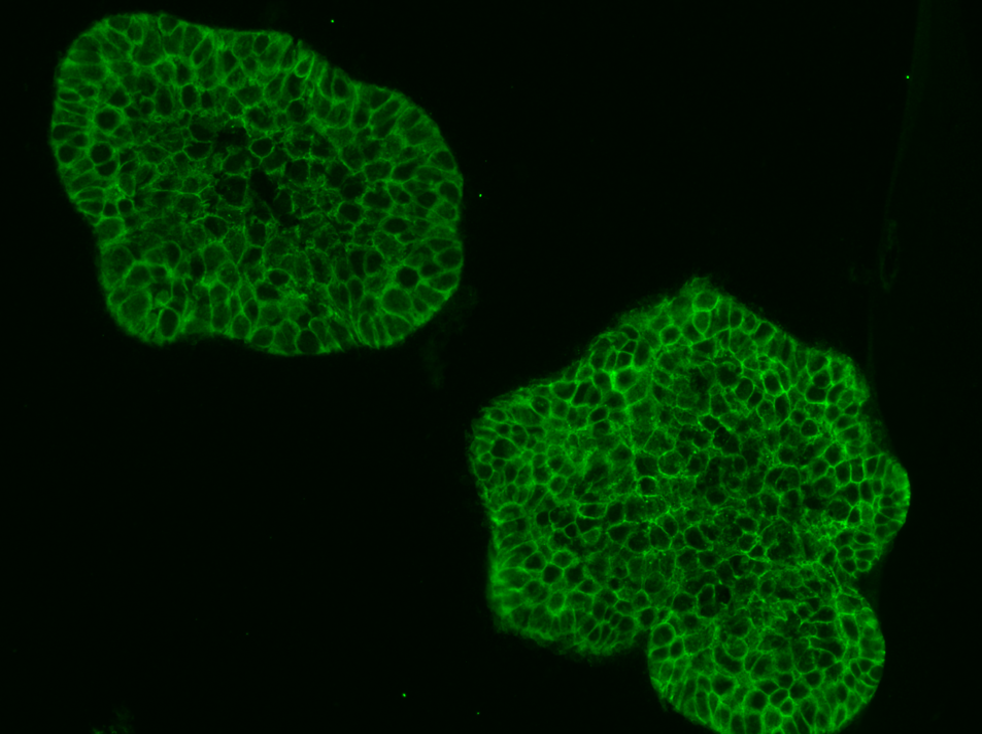

Supplement: Supplementary file 7 — Source data Fig. 1 [file 44319_2024_335_MOESM7_ESM.zip › Figure 1/1F/Cldn7 ENRA-A.tif]

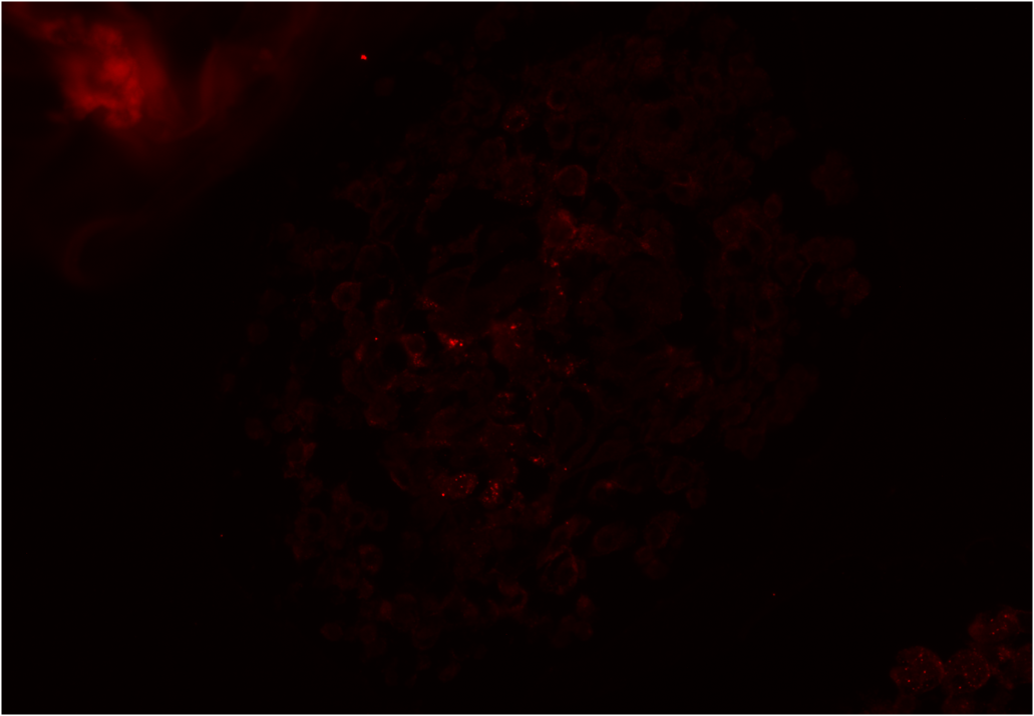

Supplement: Supplementary file 7 — Source data Fig. 1 [file 44319_2024_335_MOESM7_ESM.zip › Figure 1/1F/Cldn4 ENRA--.tif]

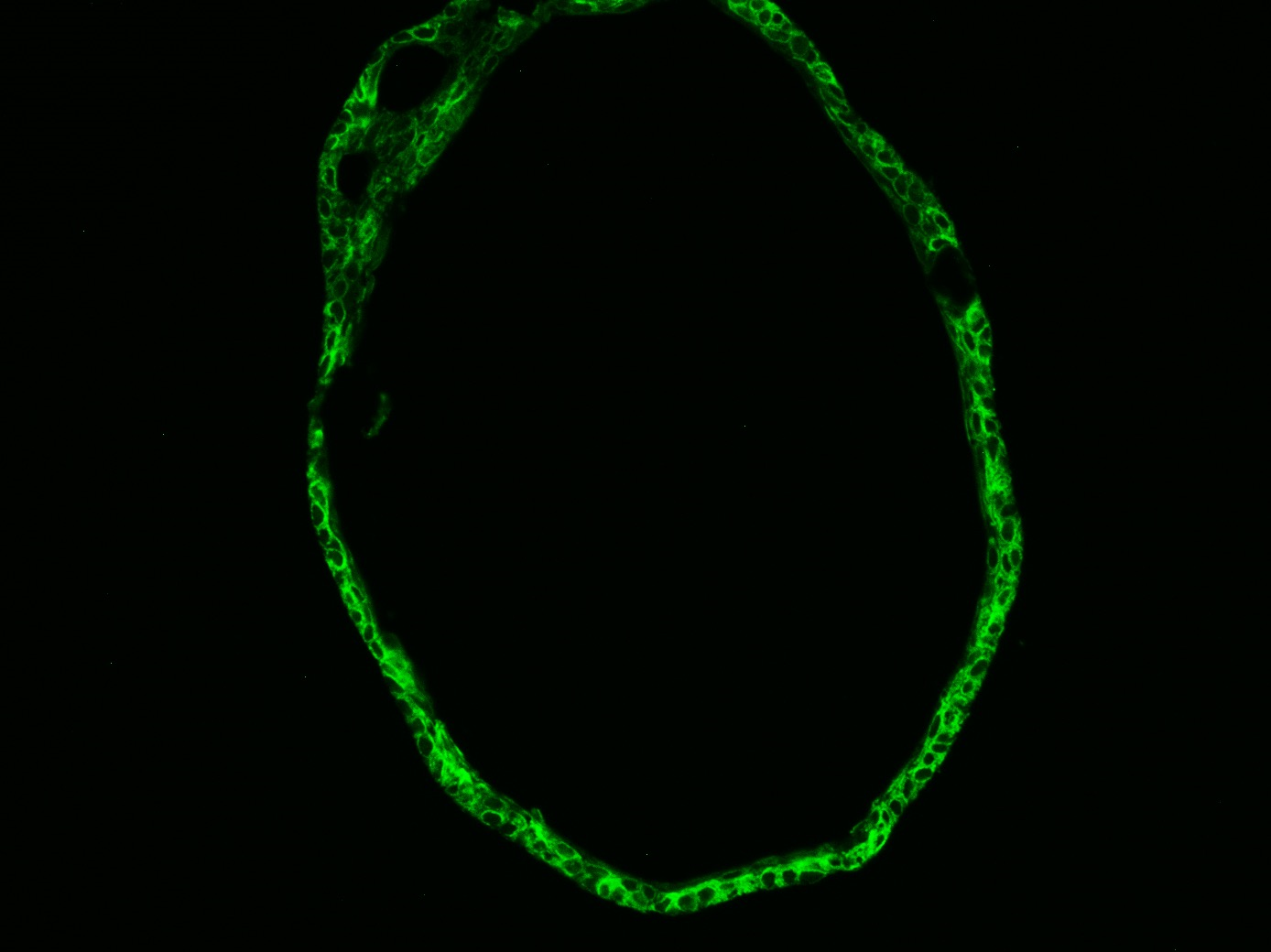

Supplement: Supplementary file 7 — Source data Fig. 1 [file 44319_2024_335_MOESM7_ESM.zip › Figure 1/1F/Ck5 ENRADA.tif]

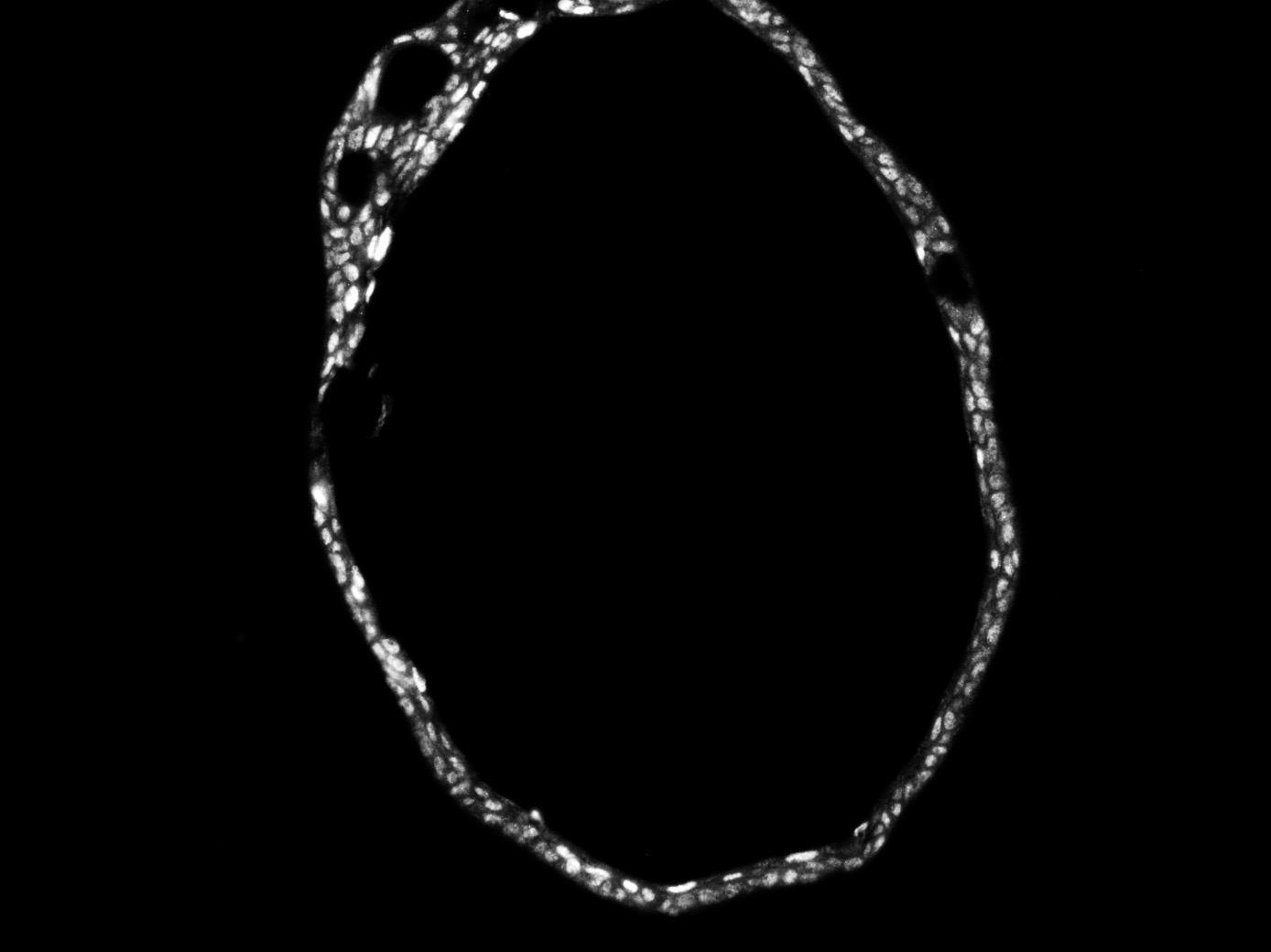

Supplement: Supplementary file 7 — Source data Fig. 1 [file 44319_2024_335_MOESM7_ESM.zip › Figure 1/1F/AR ENRADA.tif]

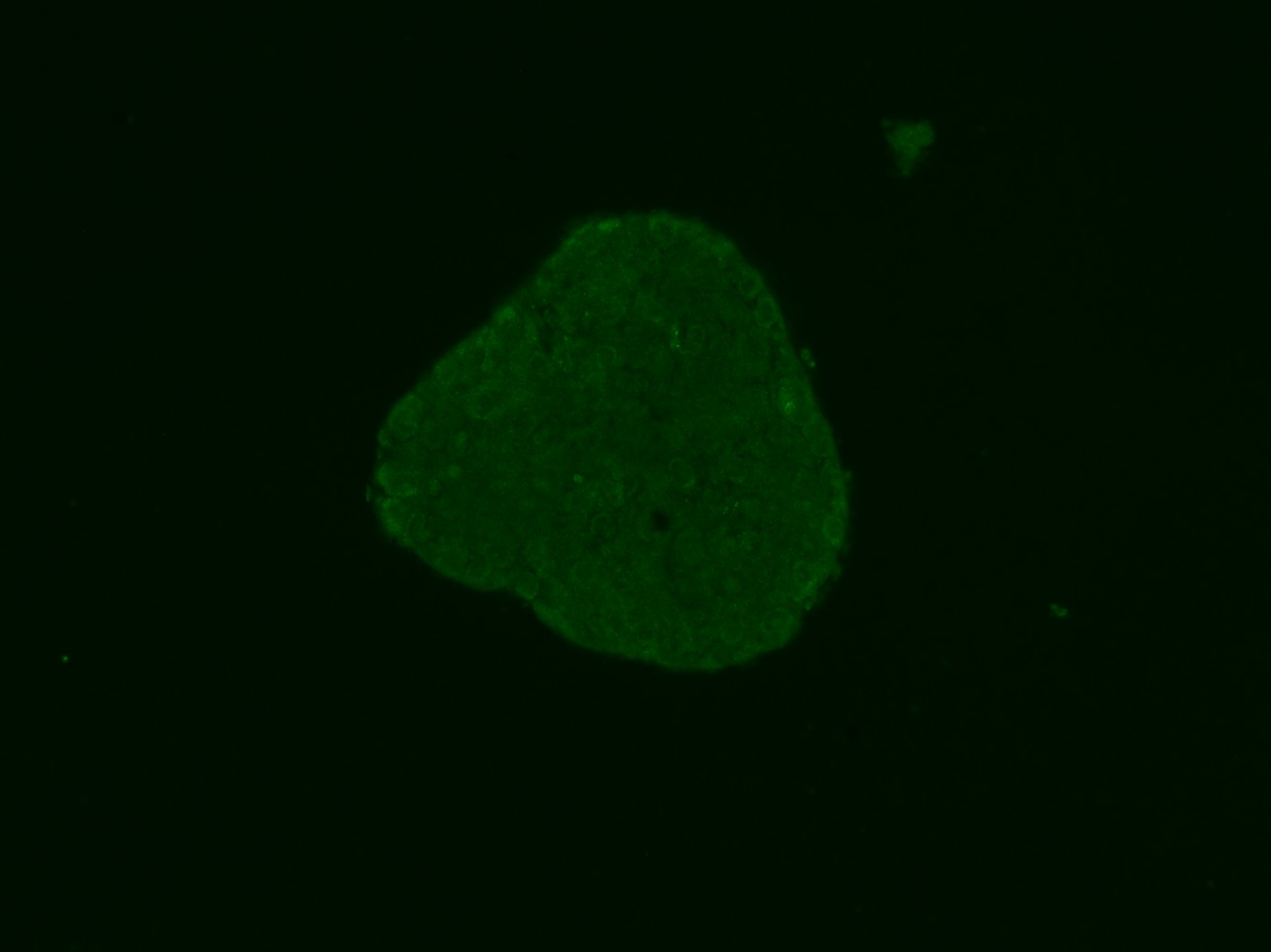

Supplement: Supplementary file 7 — Source data Fig. 1 [file 44319_2024_335_MOESM7_ESM.zip › Figure 1/1F/Zo3 ENRA-A.tif]

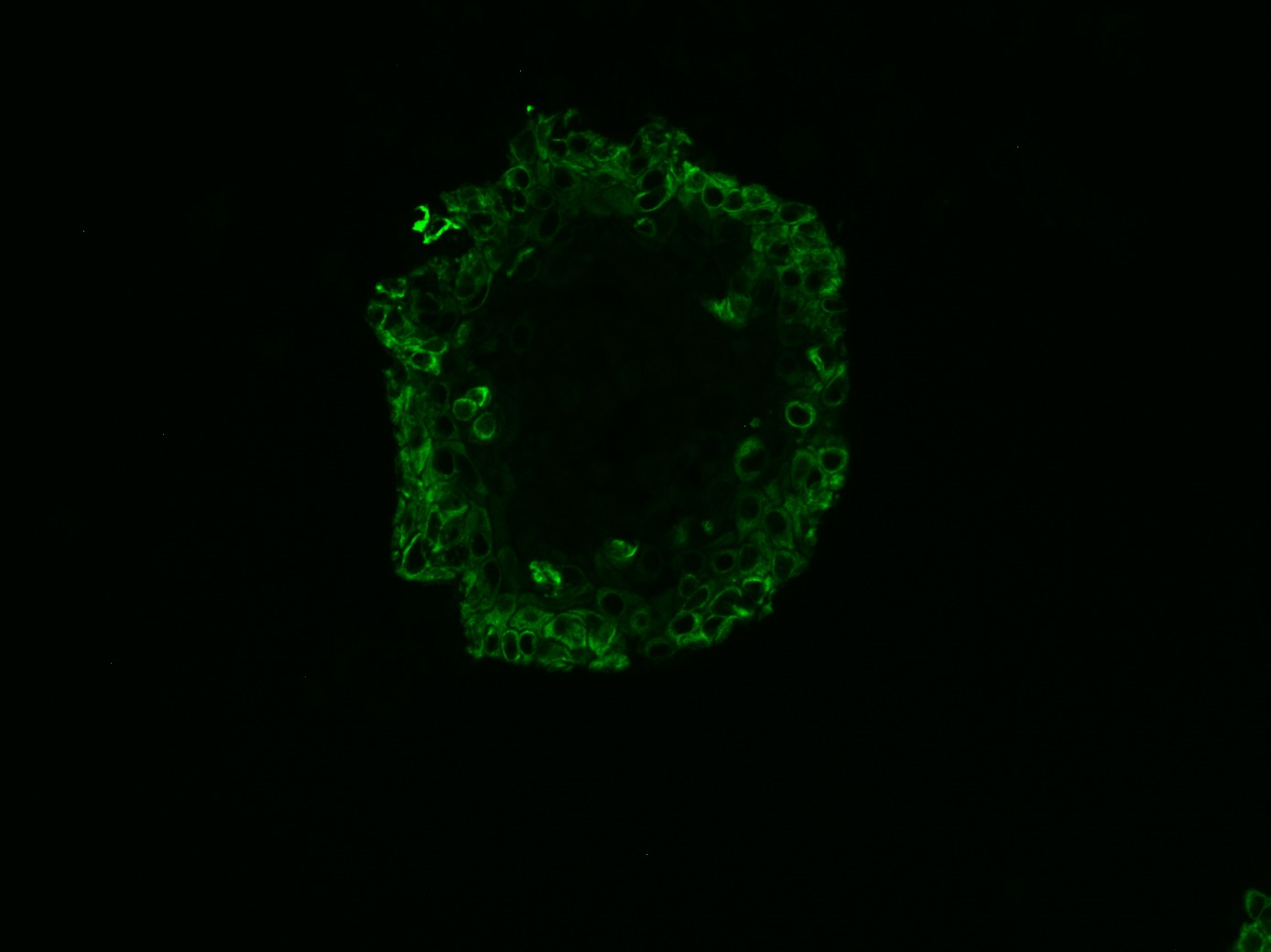

Supplement: Supplementary file 7 — Source data Fig. 1 [file 44319_2024_335_MOESM7_ESM.zip › Figure 1/1F/Ck5 ENRAD.tif]

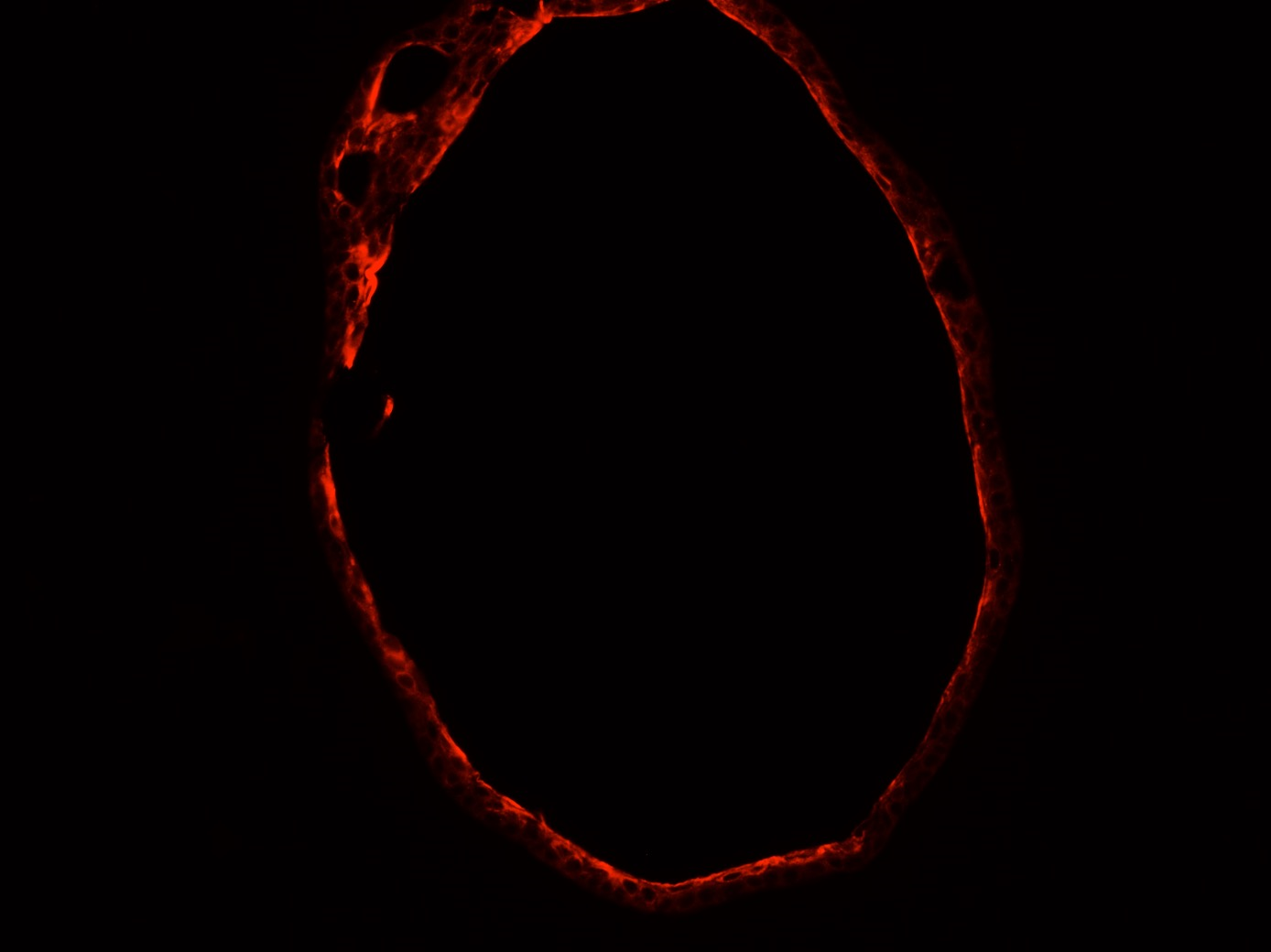

Supplement: Supplementary file 7 — Source data Fig. 1 [file 44319_2024_335_MOESM7_ESM.zip › Figure 1/1F/Ck8 ENRADA.tif]

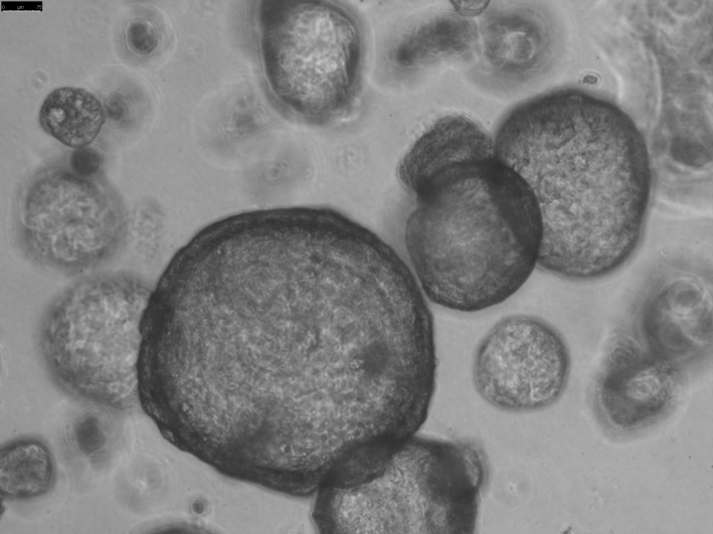

Supplement: Supplementary file 7 — Source data Fig. 1 [file 44319_2024_335_MOESM7_ESM.zip › Figure 1/1F/bf ENRADA.tif]

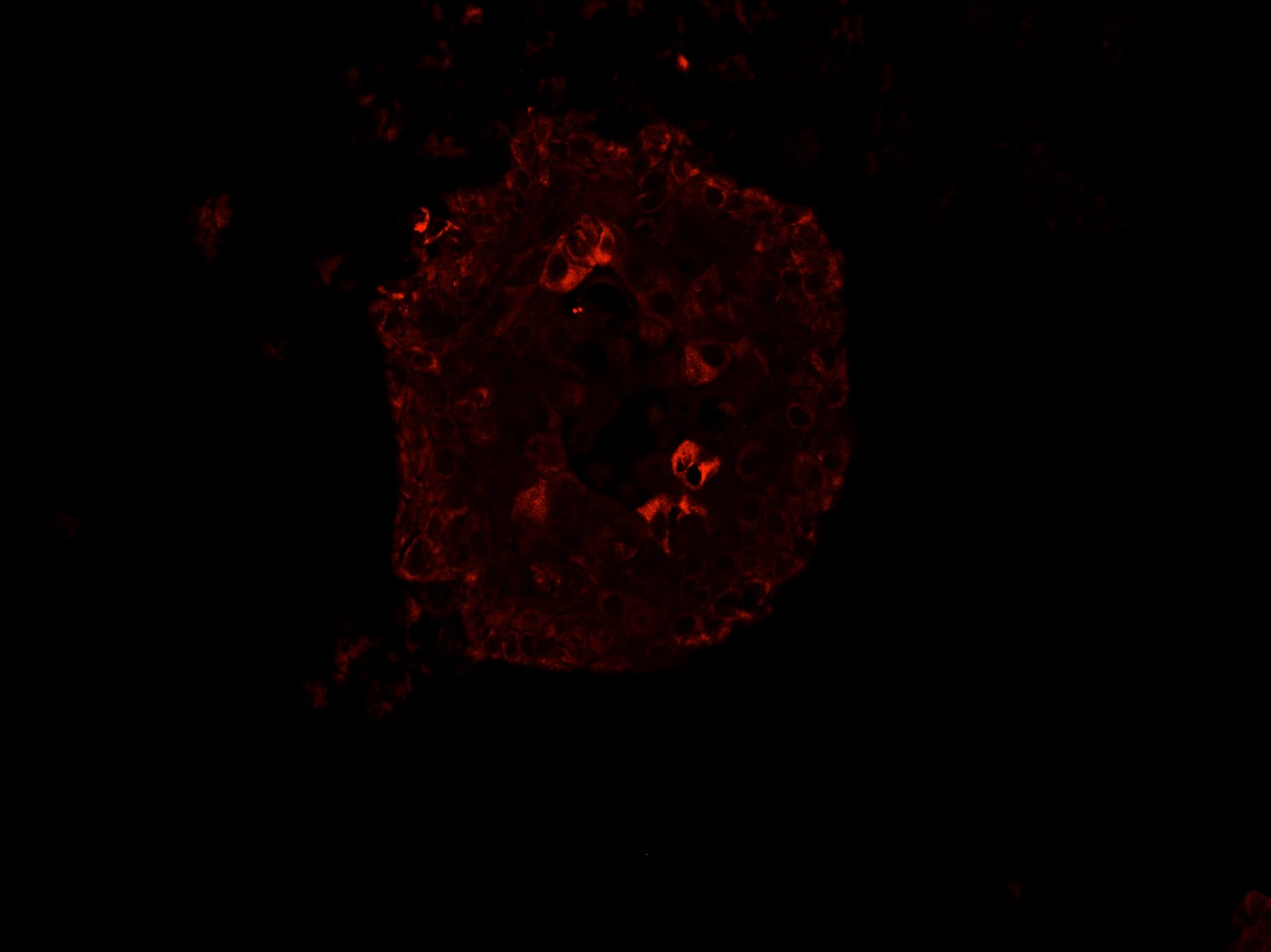

Supplement: Supplementary file 7 — Source data Fig. 1 [file 44319_2024_335_MOESM7_ESM.zip › Figure 1/1F/Ck8 ENRAD-.tif]

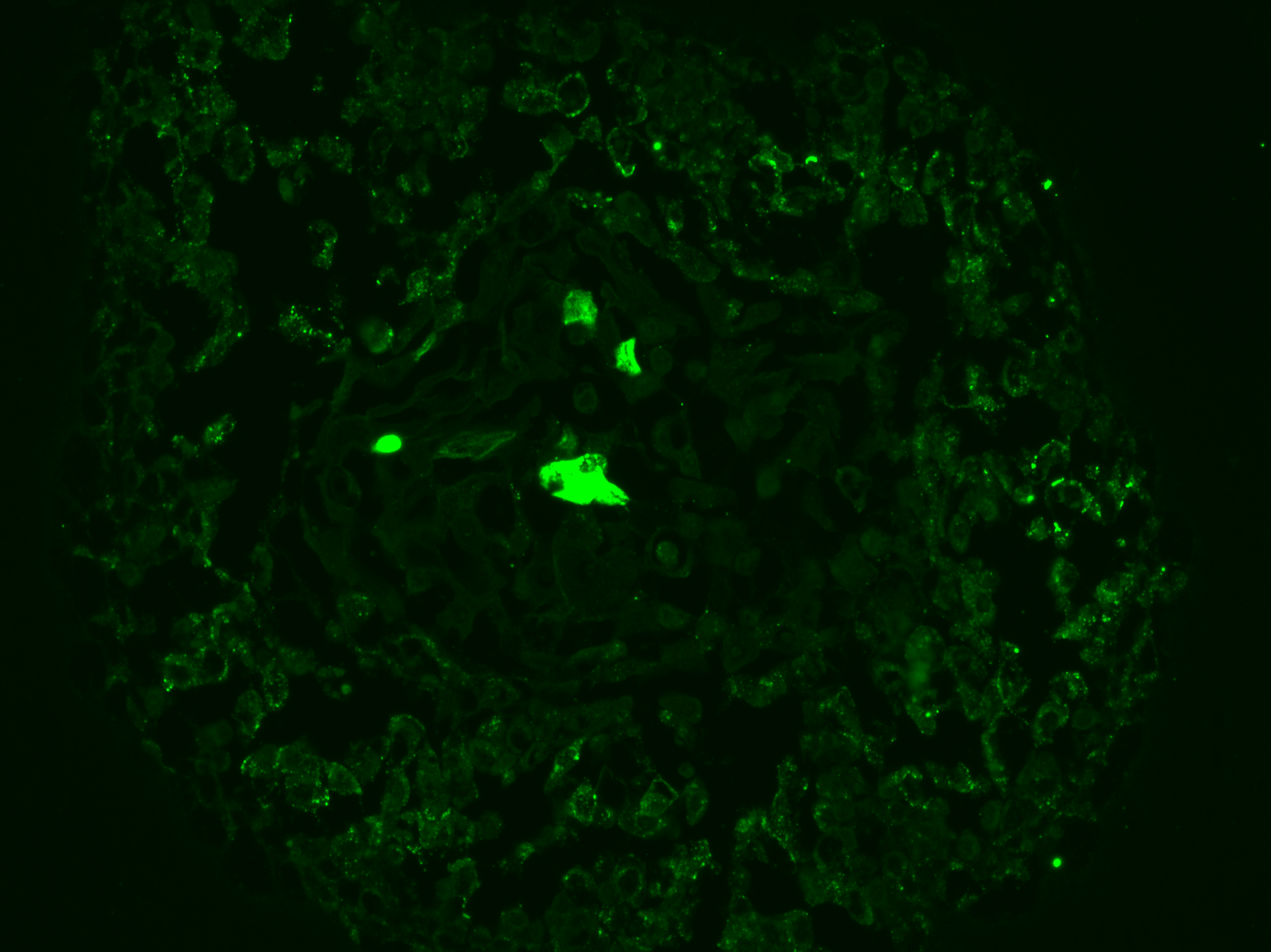

Supplement: Supplementary file 7 — Source data Fig. 1 [file 44319_2024_335_MOESM7_ESM.zip › Figure 1/1F/Zo3 ENRA--.tif]

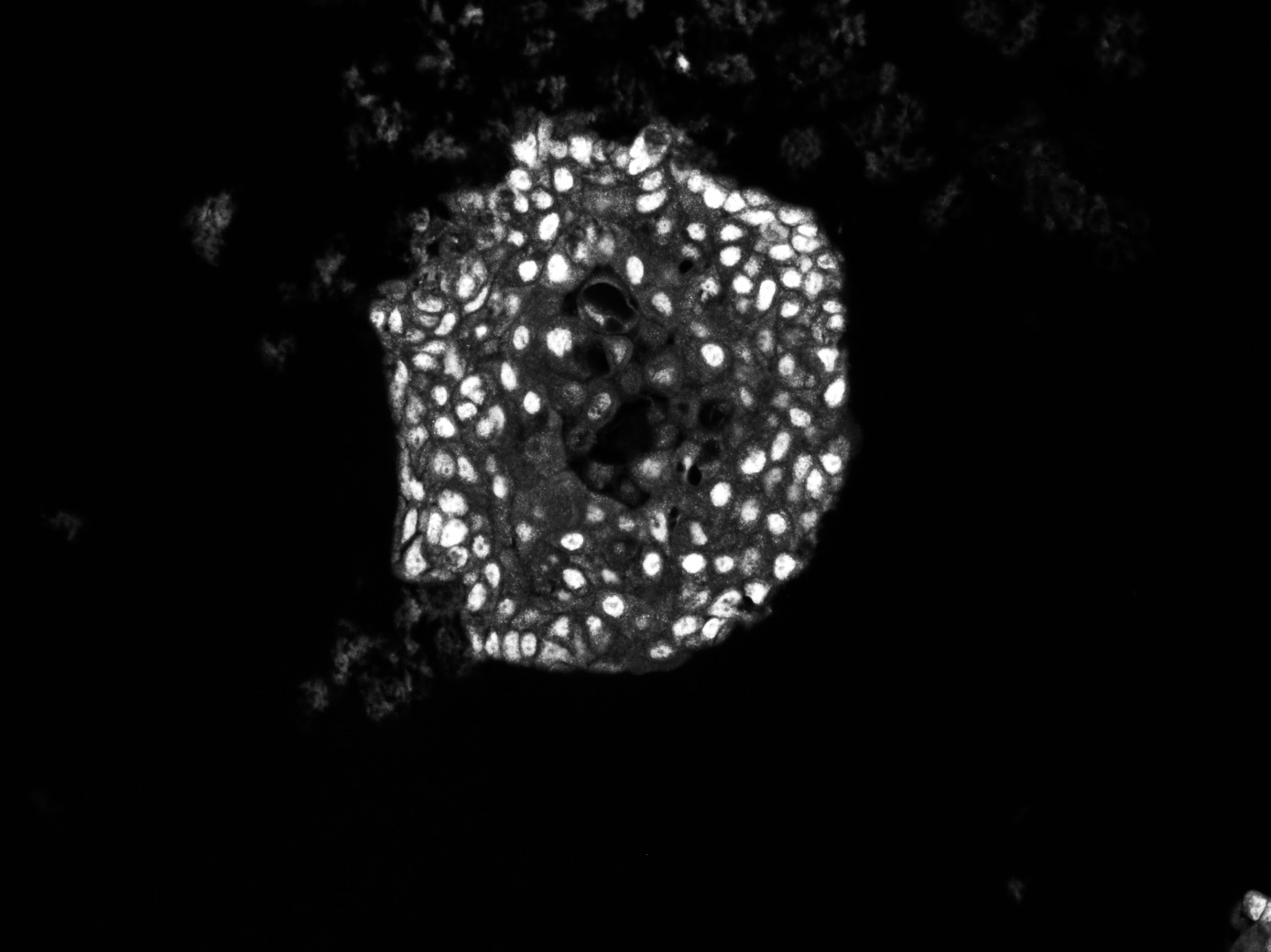

Supplement: Supplementary file 7 — Source data Fig. 1 [file 44319_2024_335_MOESM7_ESM.zip › Figure 1/1F/AR ENRAD-.tif]

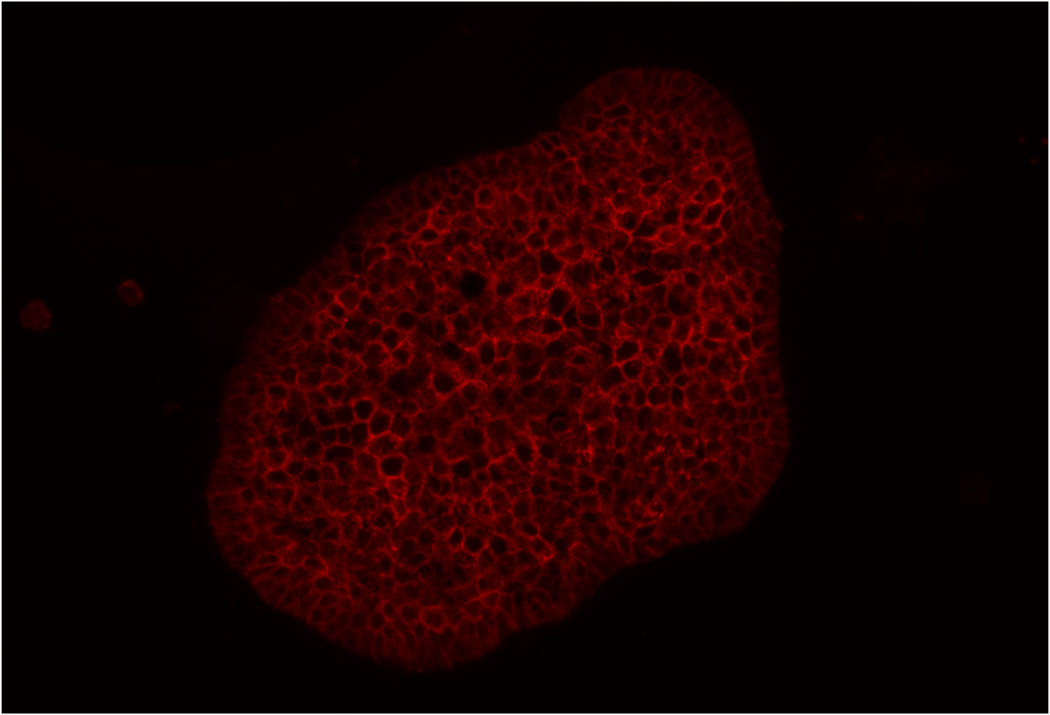

Supplement: Supplementary file 7 — Source data Fig. 1 [file 44319_2024_335_MOESM7_ESM.zip › Figure 1/1F/Cldn4 ENRA-A.tif]

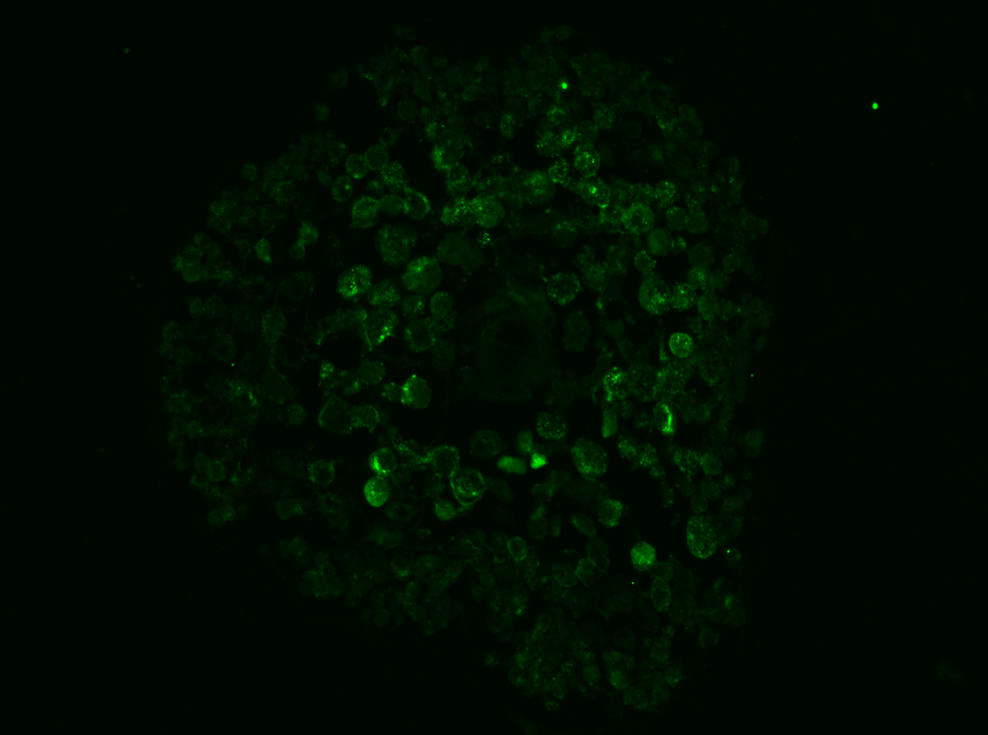

Supplement: Supplementary file 7 — Source data Fig. 1 [file 44319_2024_335_MOESM7_ESM.zip › Figure 1/1F/Cldn7 ENRA--.tif]

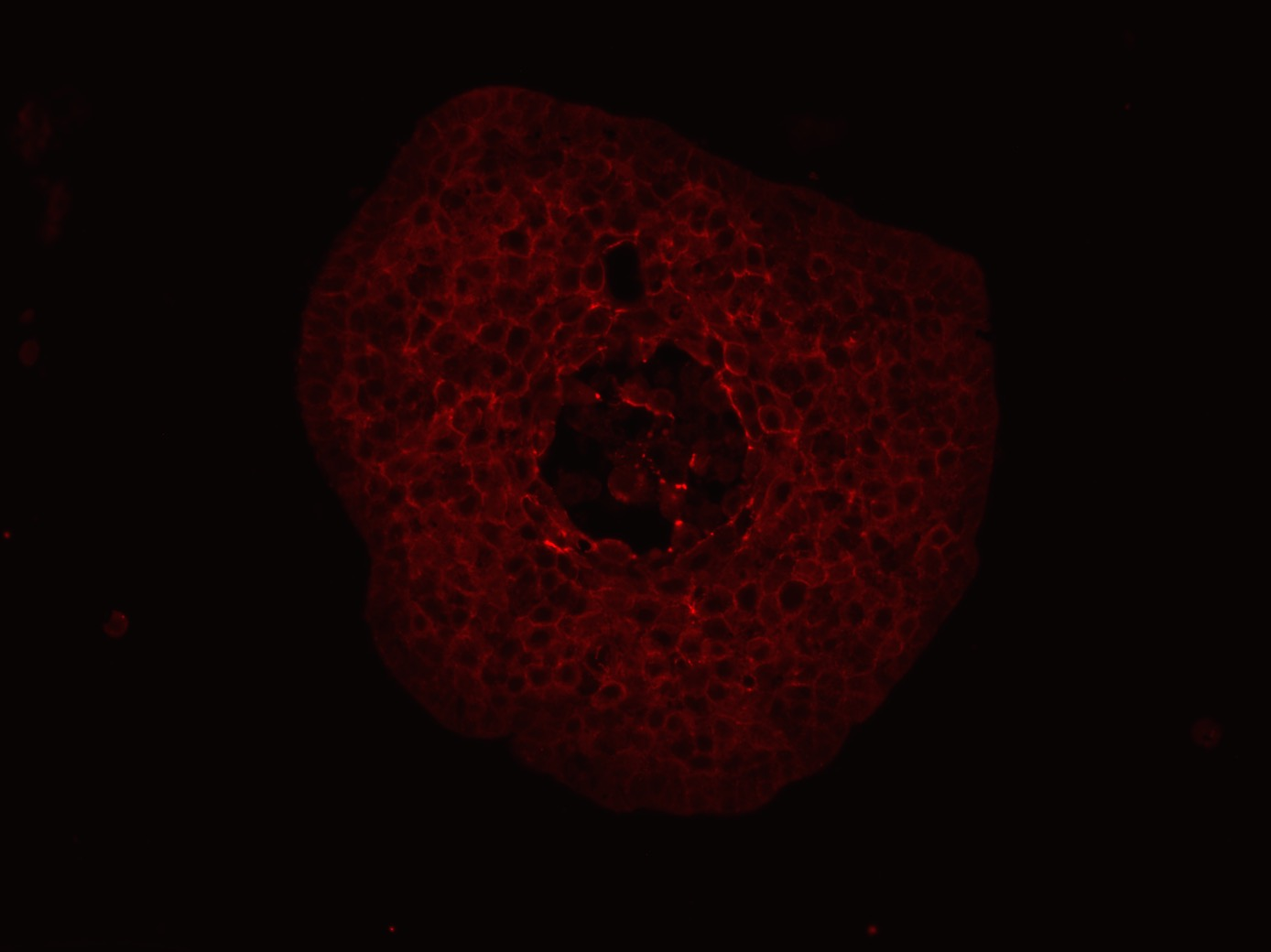

Supplement: Supplementary file 7 — Source data Fig. 1 [file 44319_2024_335_MOESM7_ESM.zip › Figure 1/1F/Zo1 ENRAD-.tif]

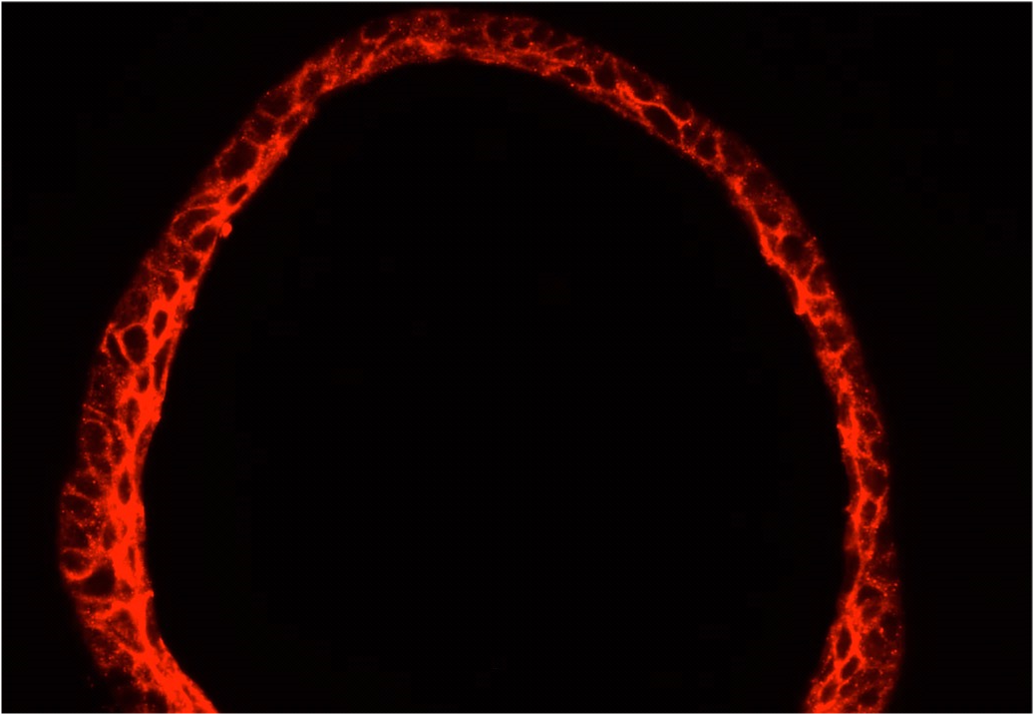

Supplement: Supplementary file 7 — Source data Fig. 1 [file 44319_2024_335_MOESM7_ESM.zip › Figure 1/1F/Cldn4 ENRADA.tif]

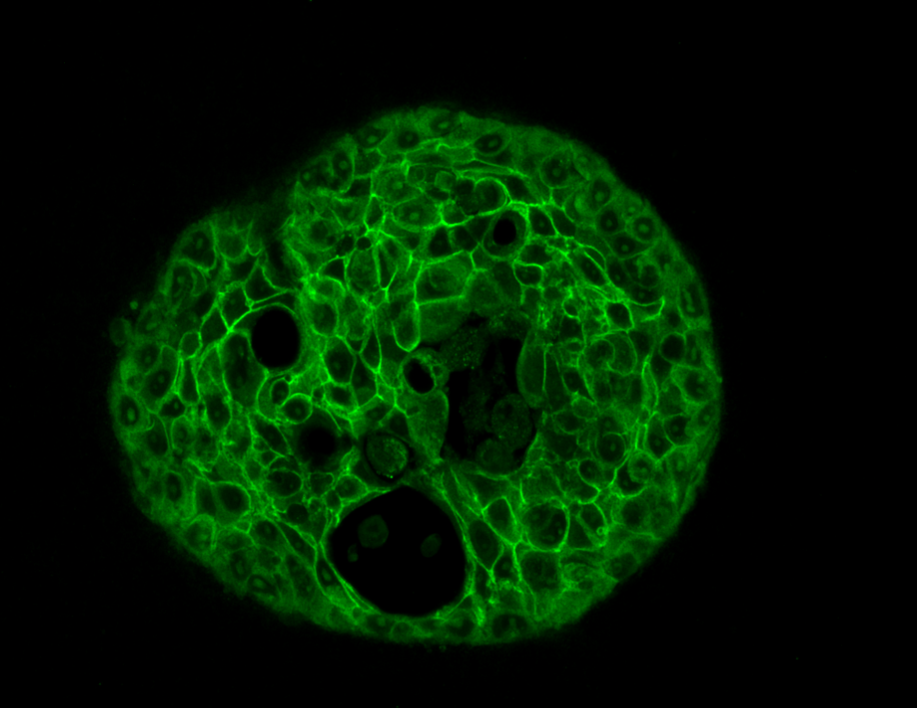

Supplement: Supplementary file 7 — Source data Fig. 1 [file 44319_2024_335_MOESM7_ESM.zip › Figure 1/1F/Cldn7 ENRAD-.tif]

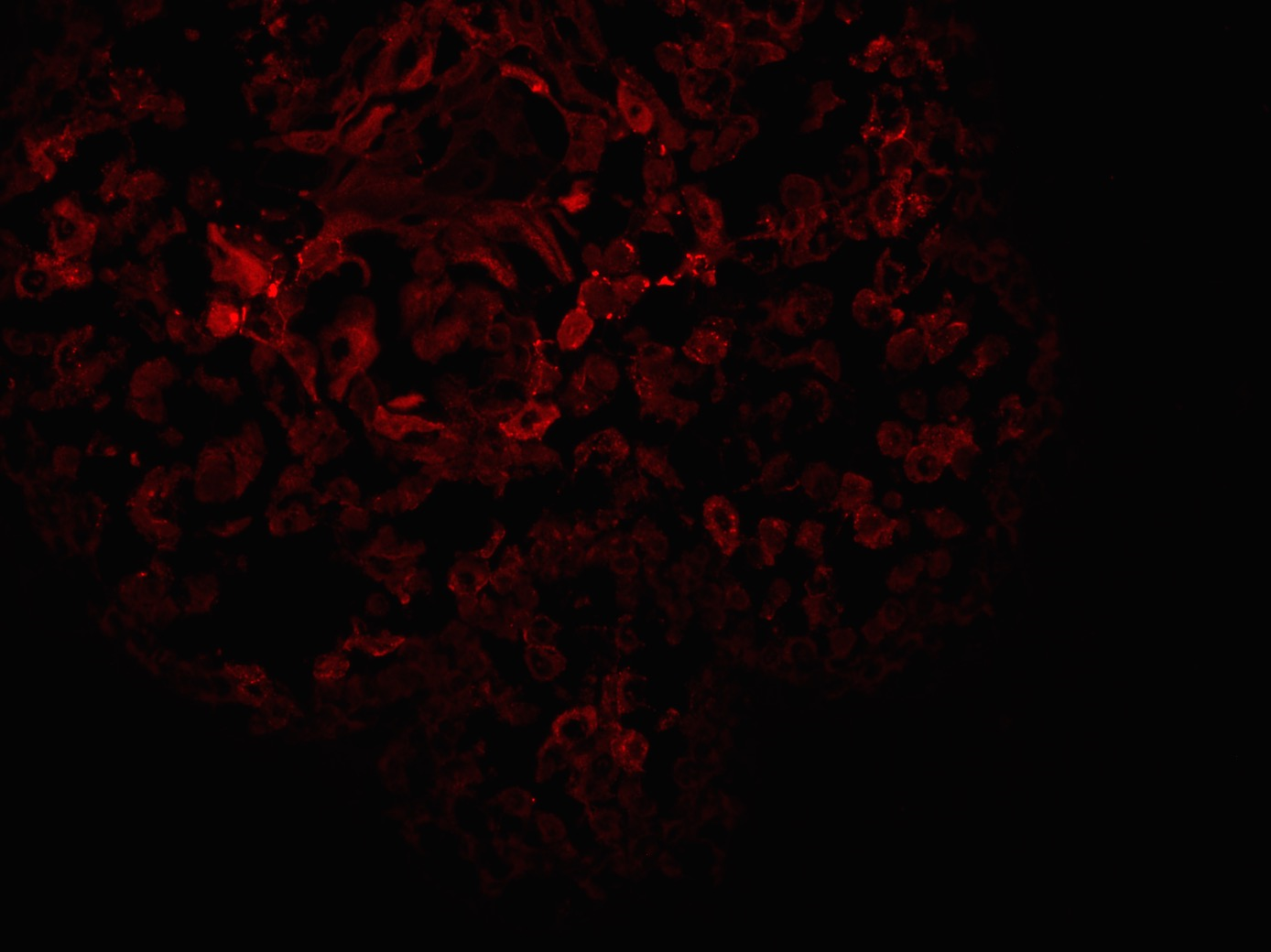

Supplement: Supplementary file 7 — Source data Fig. 1 [file 44319_2024_335_MOESM7_ESM.zip › Figure 1/1F/Zo1 ENRA--.tif]

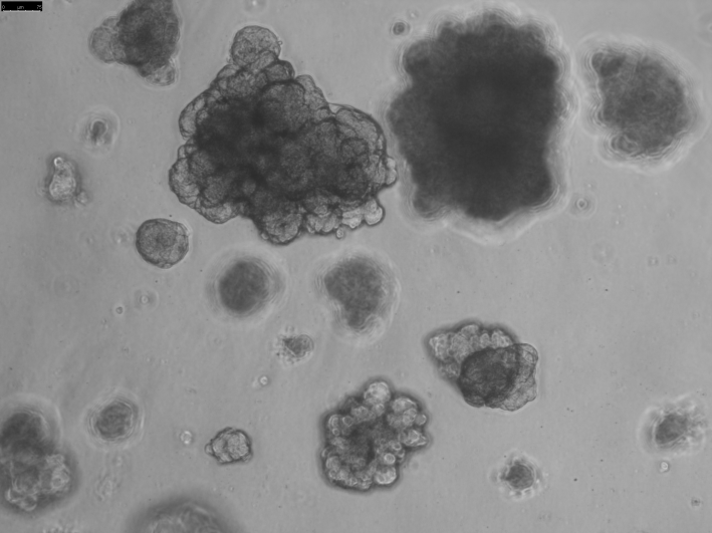

Supplement: Supplementary file 7 — Source data Fig. 1 [file 44319_2024_335_MOESM7_ESM.zip › Figure 1/1F/bf ENRA--.tif]

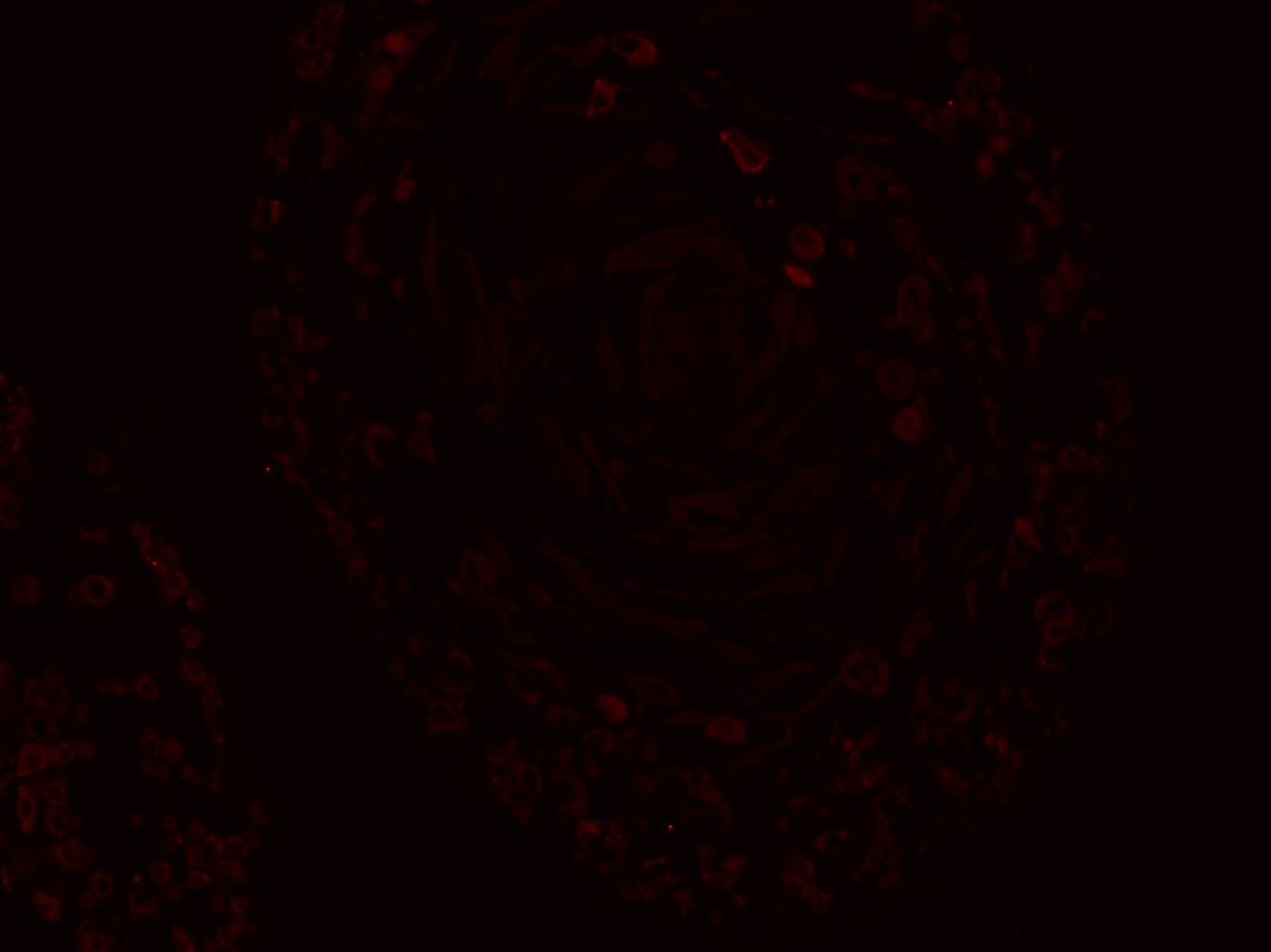

Supplement: Supplementary file 7 — Source data Fig. 1 [file 44319_2024_335_MOESM7_ESM.zip › Figure 1/1F/Ck8 ENRA--.tif]

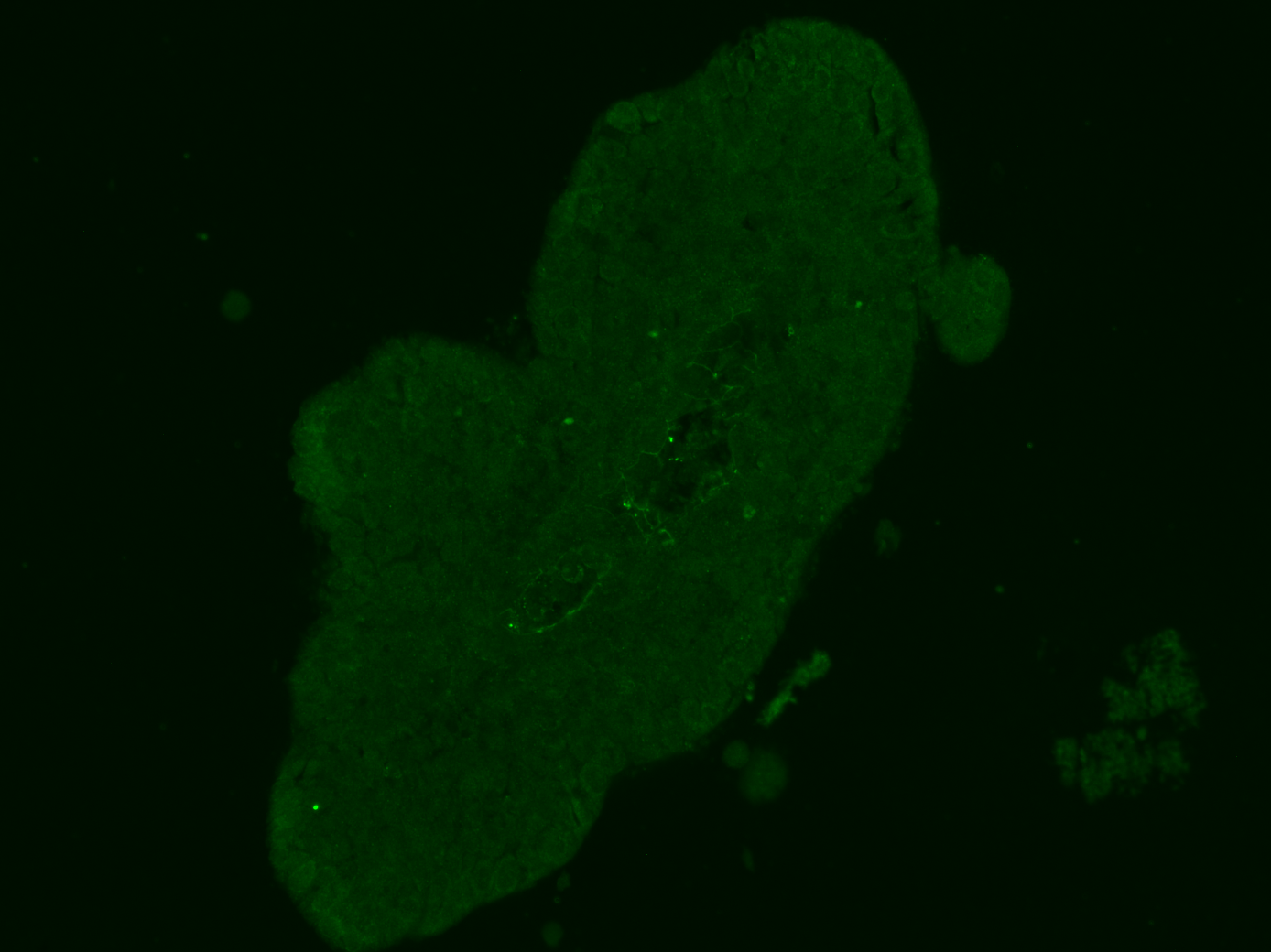

Supplement: Supplementary file 7 — Source data Fig. 1 [file 44319_2024_335_MOESM7_ESM.zip › Figure 1/1F/Zo3 ENRAD-.tif]

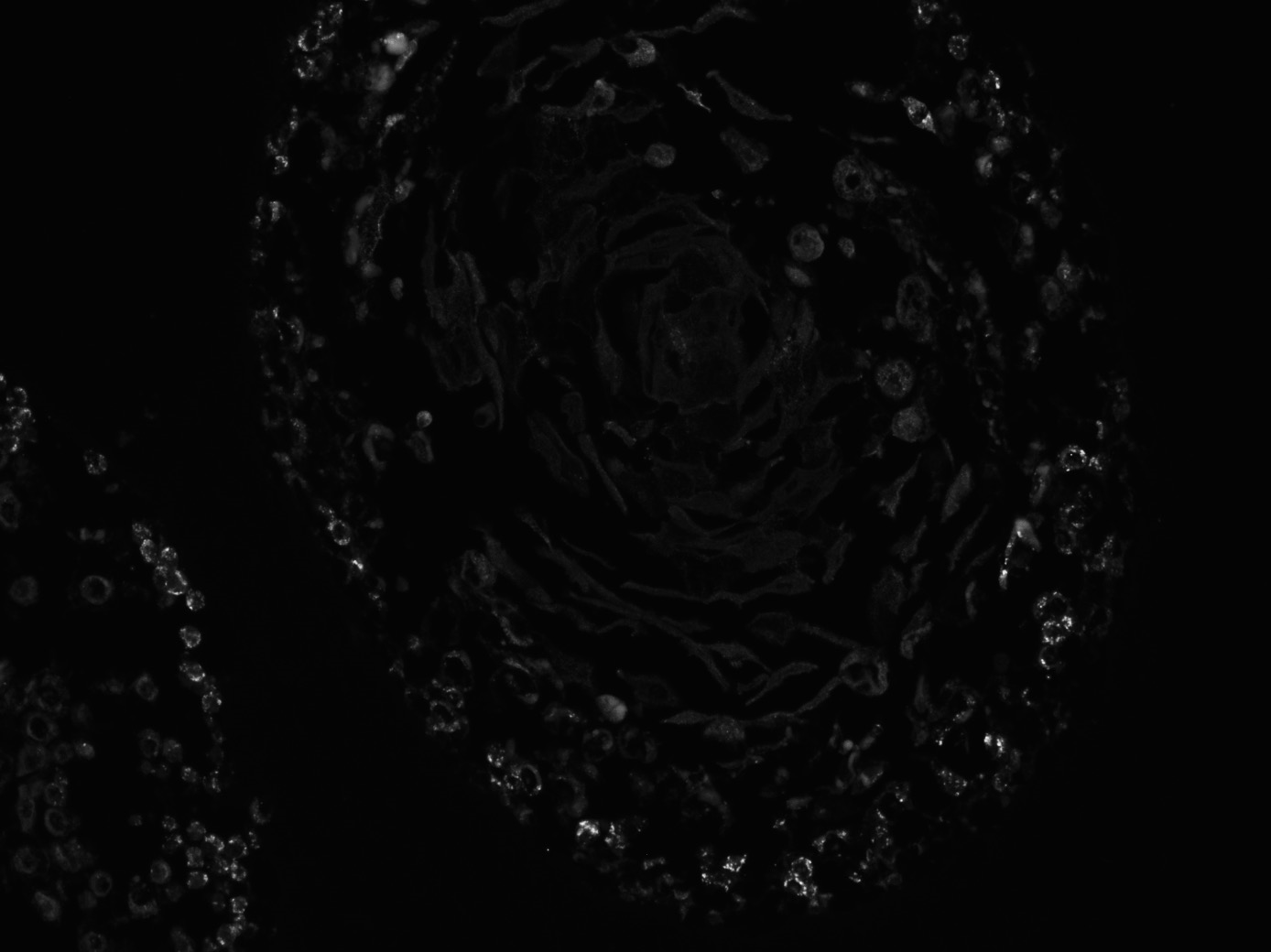

Supplement: Supplementary file 7 — Source data Fig. 1 [file 44319_2024_335_MOESM7_ESM.zip › Figure 1/1F/AR ENRA--.tif]

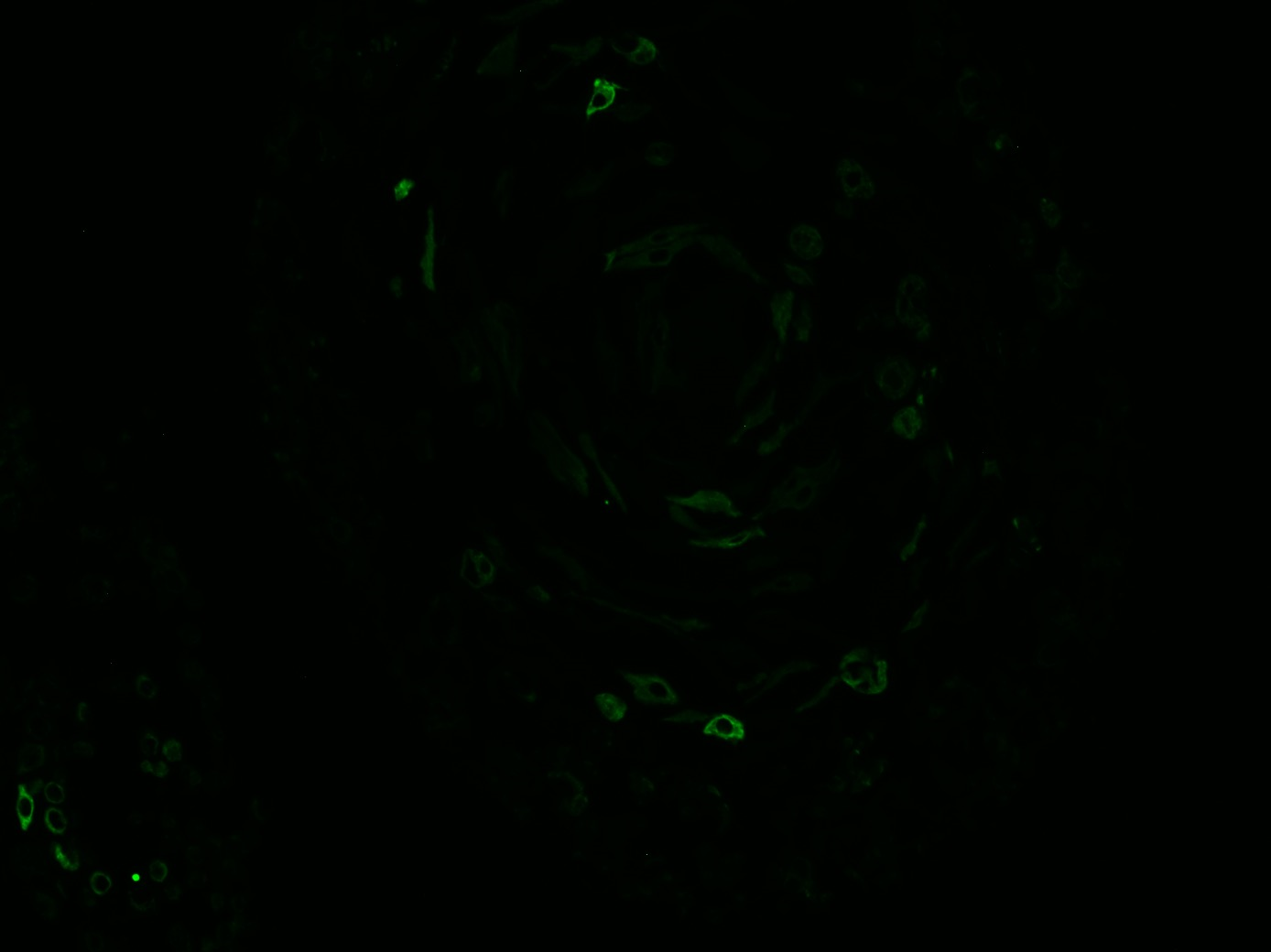

Supplement: Supplementary file 7 — Source data Fig. 1 [file 44319_2024_335_MOESM7_ESM.zip › Figure 1/1F/Ck5 ENRA--.tif]

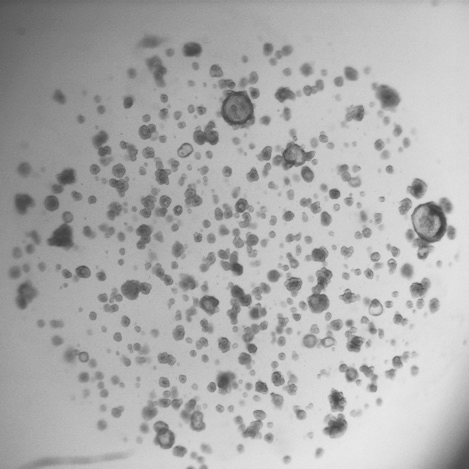

Supplement: Supplementary file 7 — Source data Fig. 1 [file 44319_2024_335_MOESM7_ESM.zip › Figure 1/1G/8.tif]

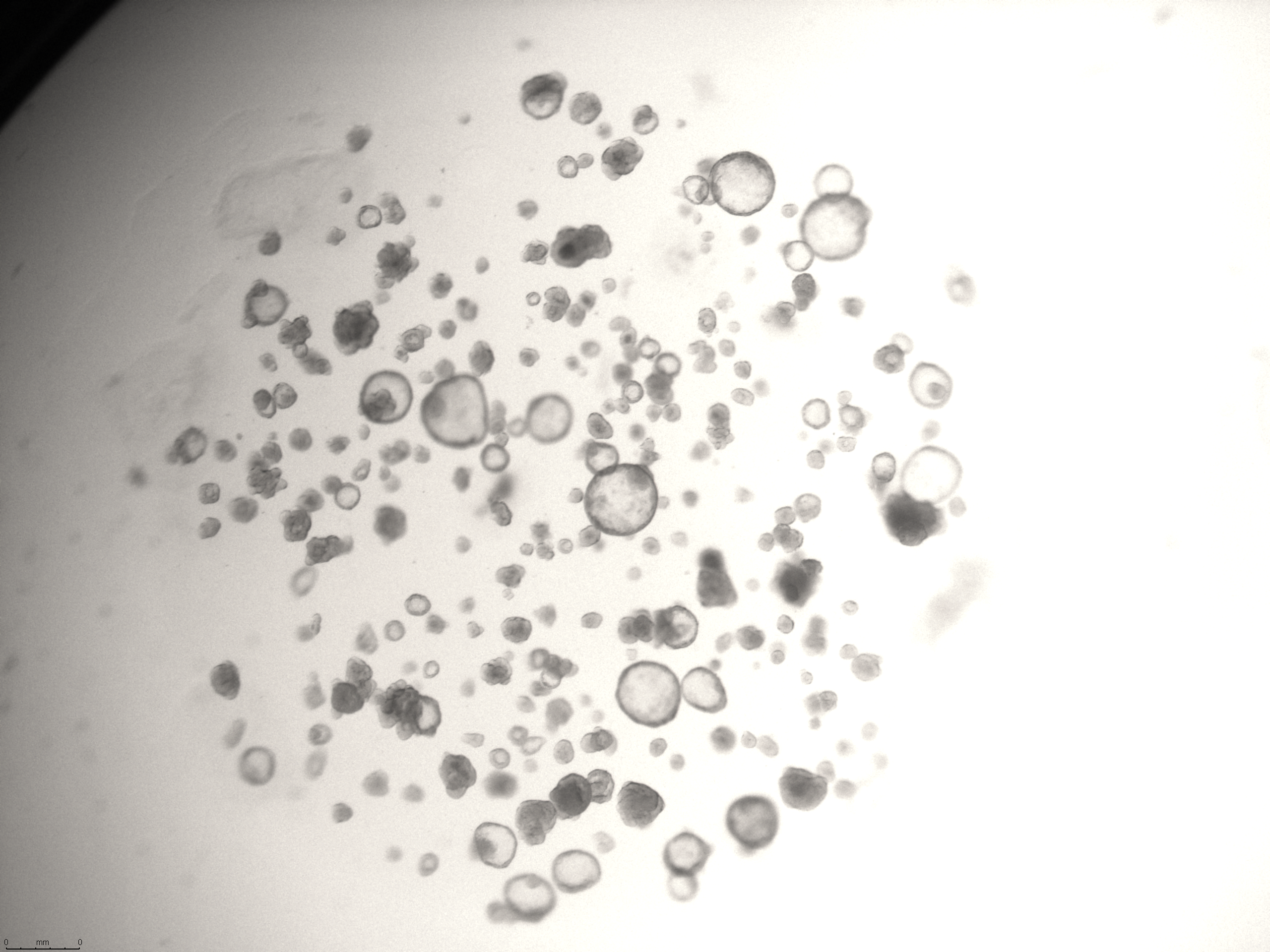

Supplement: Supplementary file 7 — Source data Fig. 1 [file 44319_2024_335_MOESM7_ESM.zip › Figure 1/1G/3.tif]

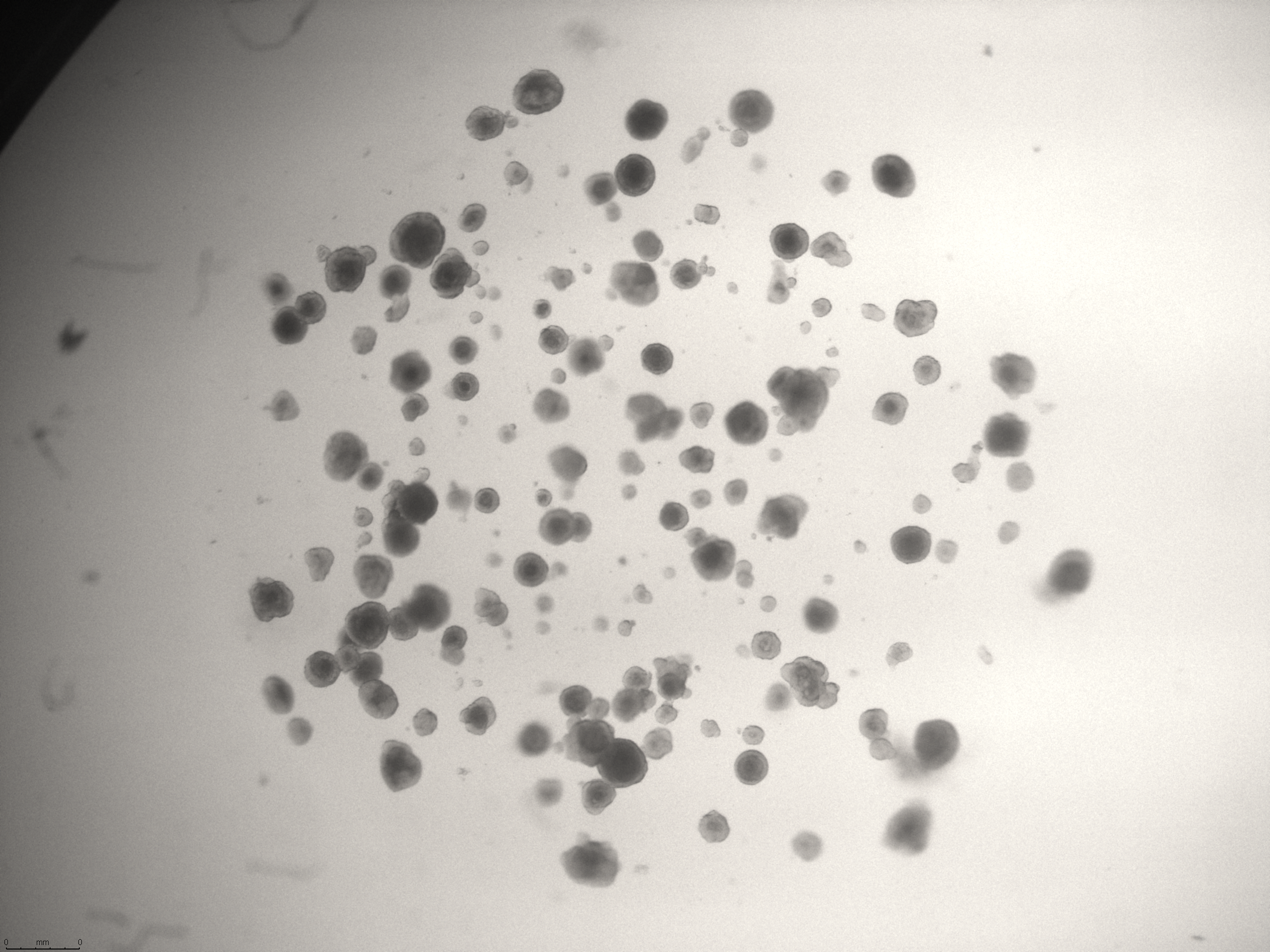

Supplement: Supplementary file 7 — Source data Fig. 1 [file 44319_2024_335_MOESM7_ESM.zip › Figure 1/1G/2.tif]

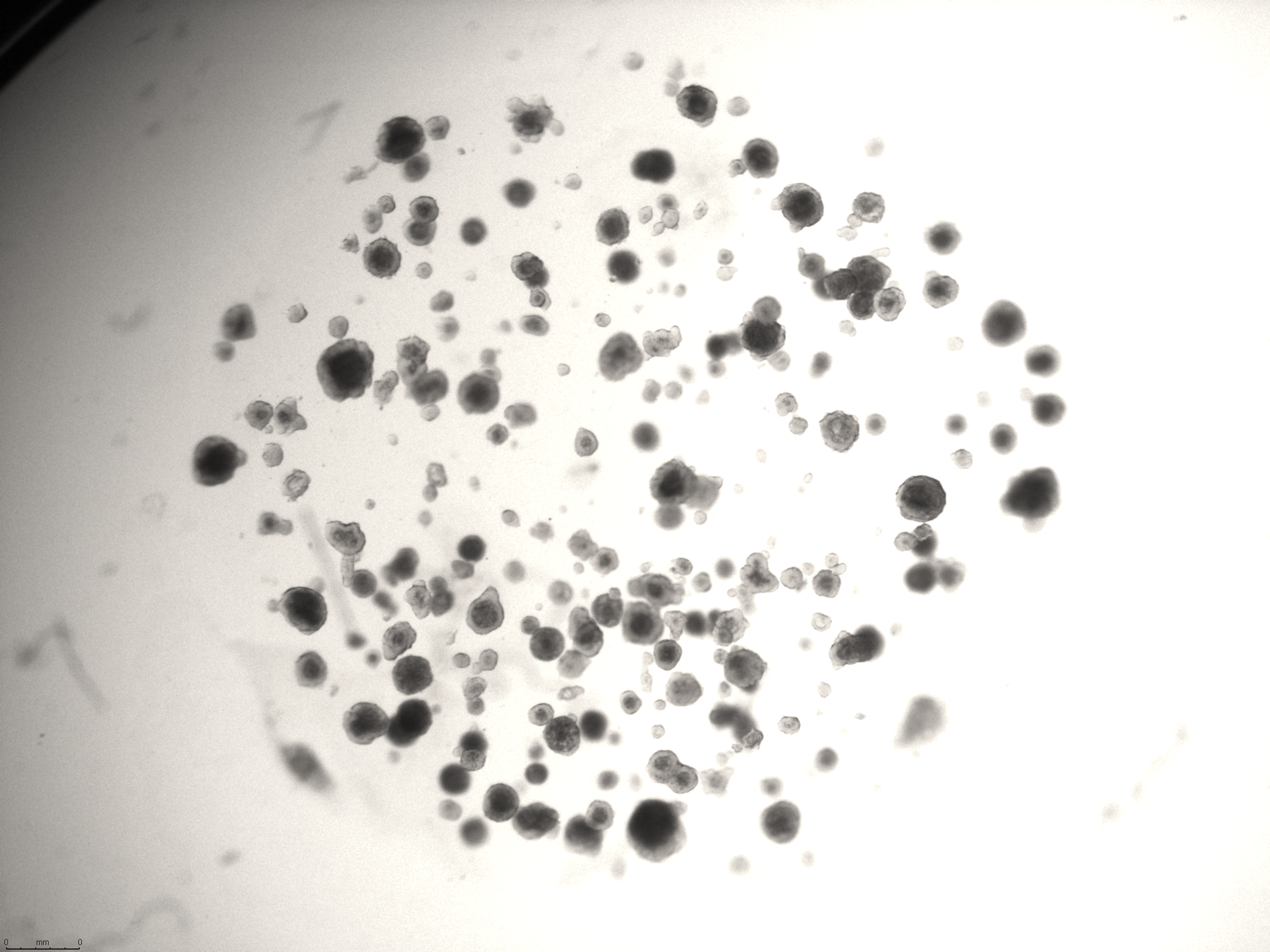

Supplement: Supplementary file 7 — Source data Fig. 1 [file 44319_2024_335_MOESM7_ESM.zip › Figure 1/1G/1.tif]

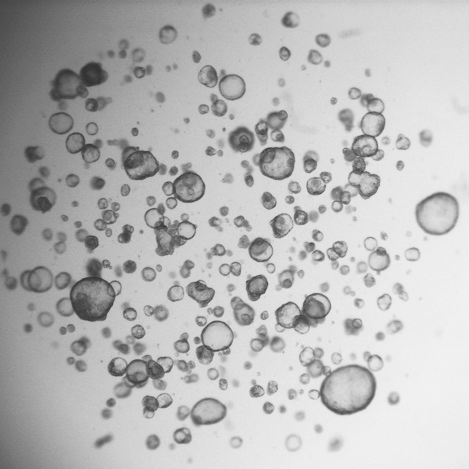

Supplement: Supplementary file 7 — Source data Fig. 1 [file 44319_2024_335_MOESM7_ESM.zip › Figure 1/1G/5.tif]

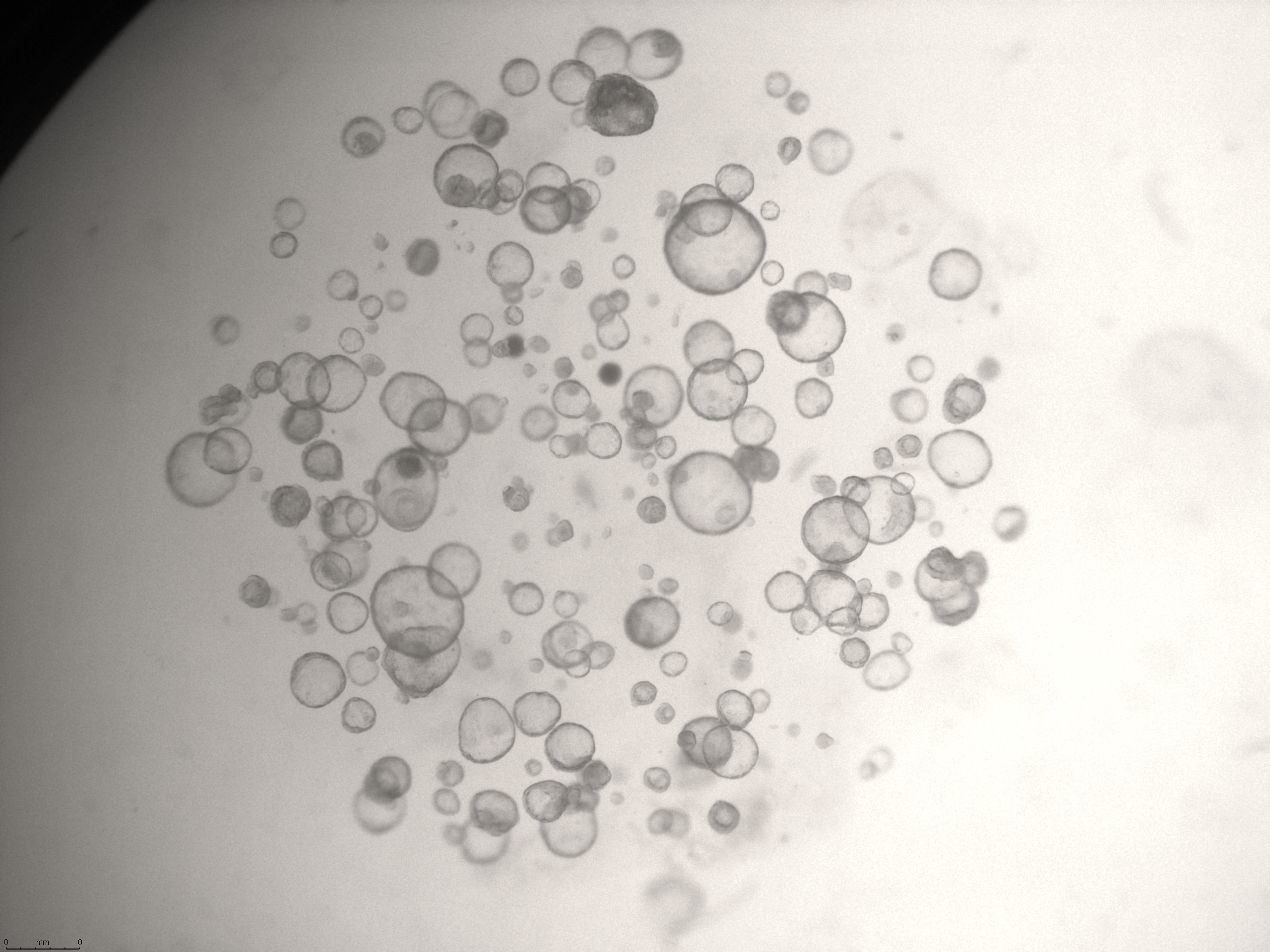

Supplement: Supplementary file 7 — Source data Fig. 1 [file 44319_2024_335_MOESM7_ESM.zip › Figure 1/1G/4.tif]

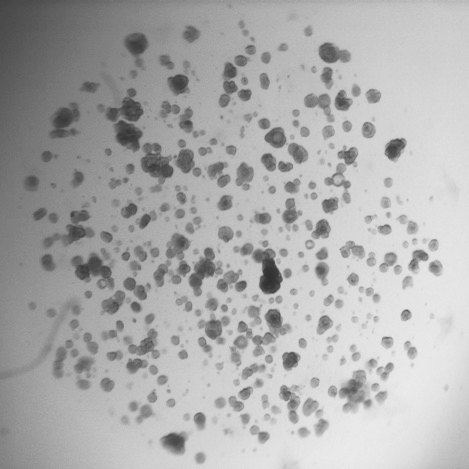

Supplement: Supplementary file 7 — Source data Fig. 1 [file 44319_2024_335_MOESM7_ESM.zip › Figure 1/1G/6.tif]

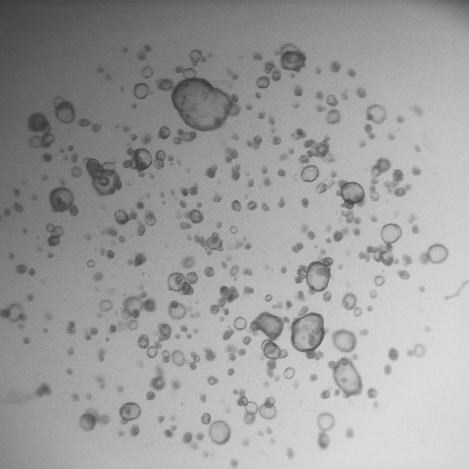

Supplement: Supplementary file 7 — Source data Fig. 1 [file 44319_2024_335_MOESM7_ESM.zip › Figure 1/1G/7.tif]

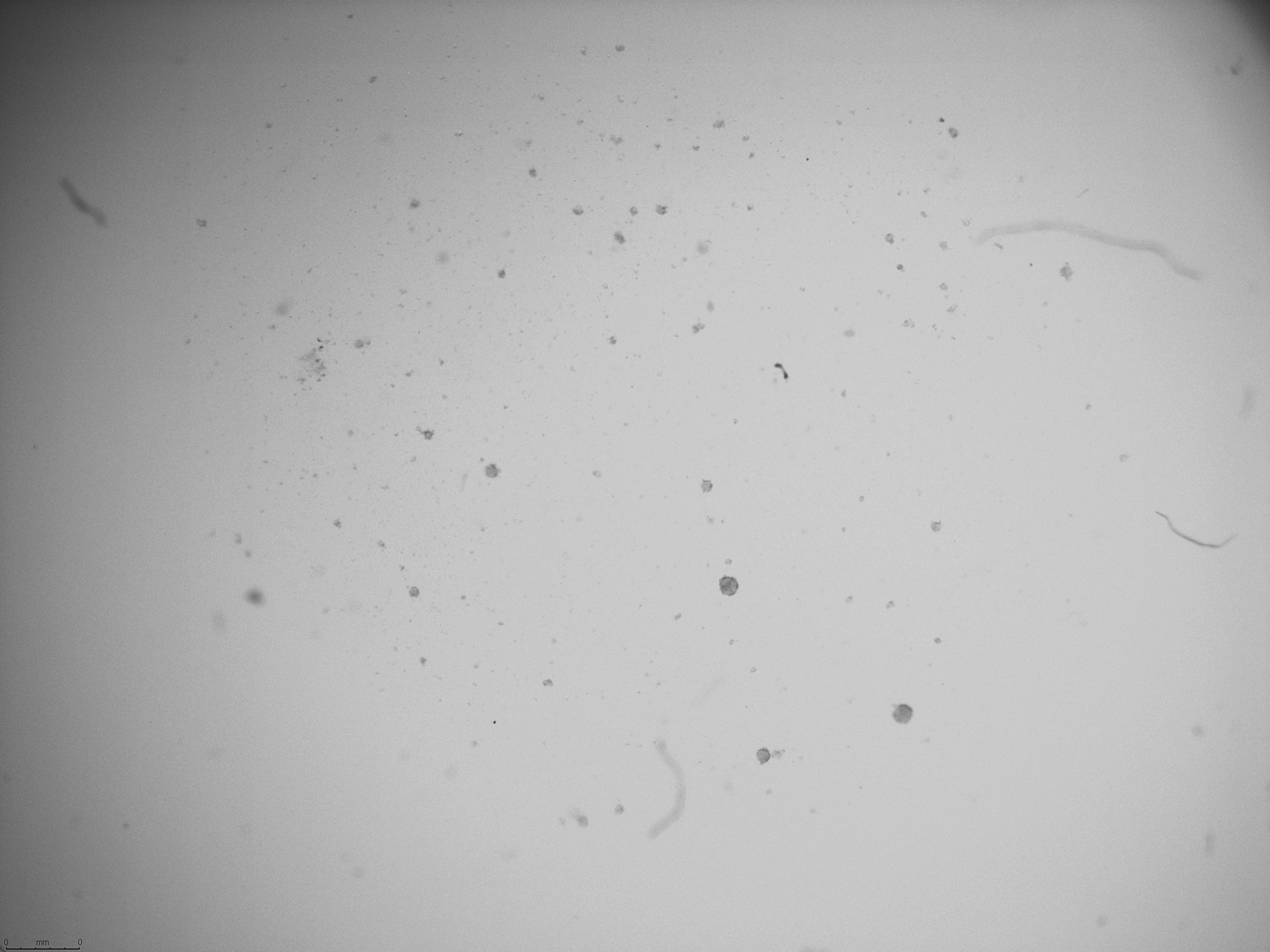

Supplement: Supplementary file 7 — Source data Fig. 1 [file 44319_2024_335_MOESM7_ESM.zip › Figure 1/1B/day1.tif]

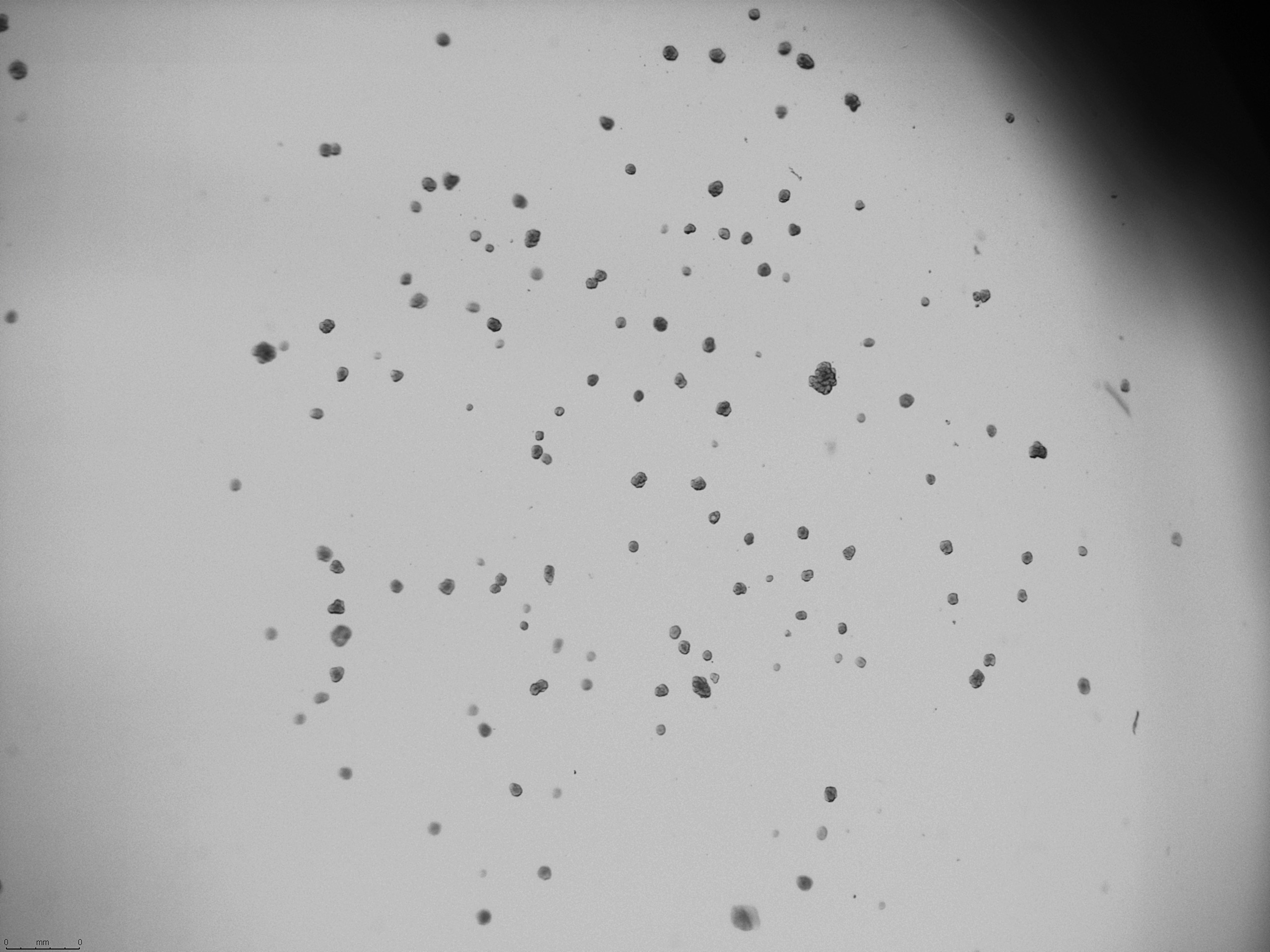

Supplement: Supplementary file 7 — Source data Fig. 1 [file 44319_2024_335_MOESM7_ESM.zip › Figure 1/1B/day3.tif]

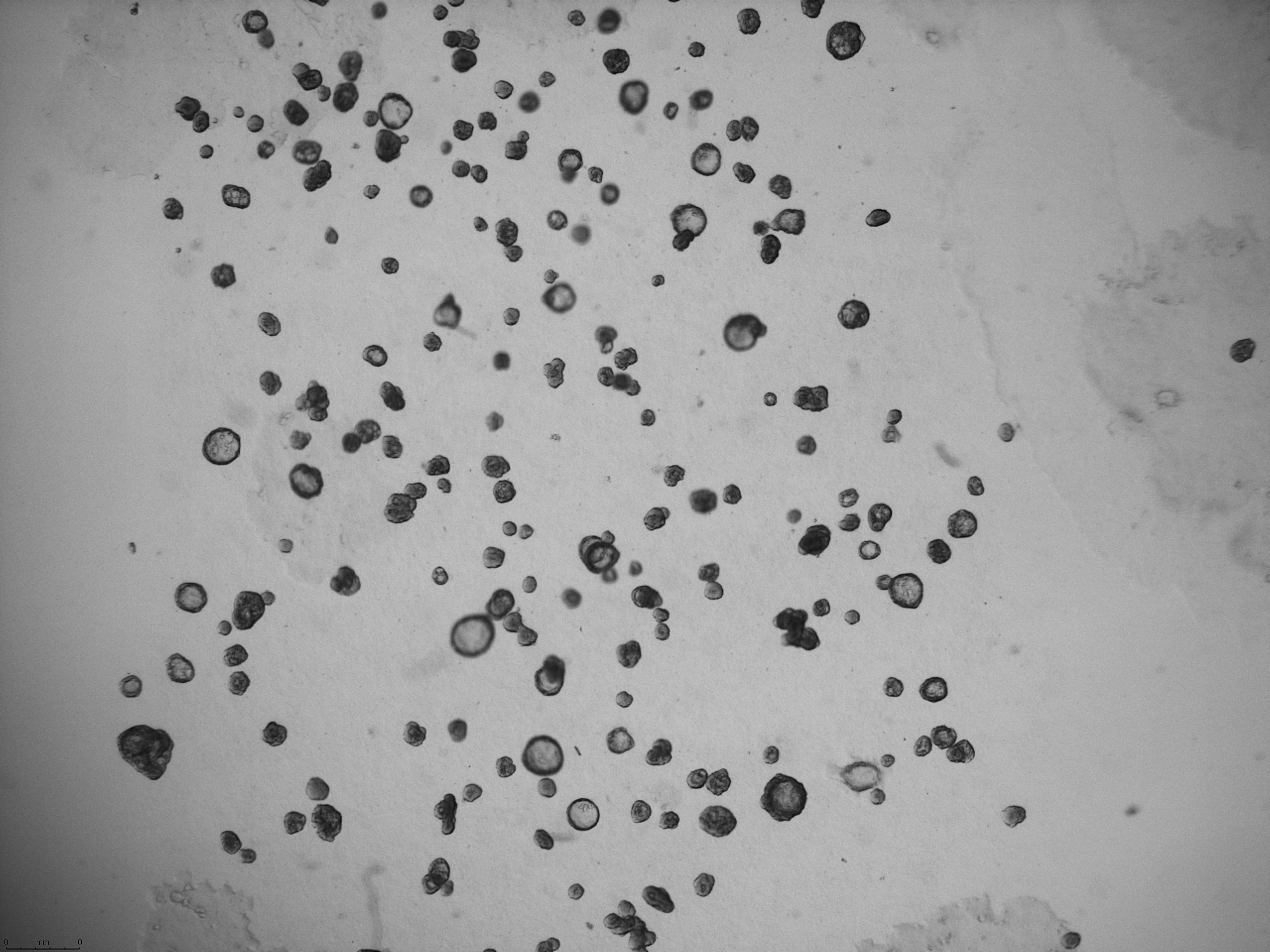

Supplement: Supplementary file 7 — Source data Fig. 1 [file 44319_2024_335_MOESM7_ESM.zip › Figure 1/1B/day7.tif]

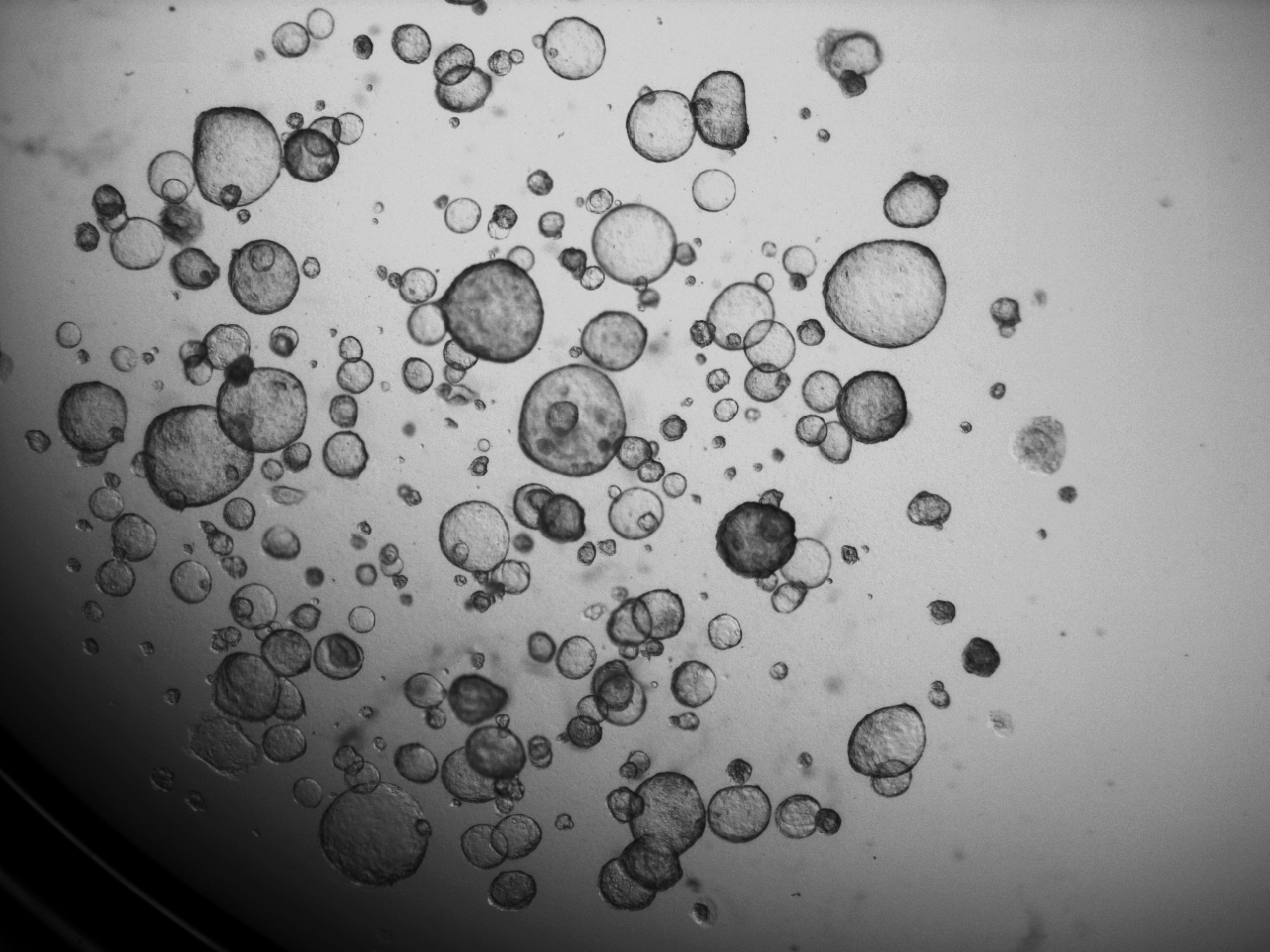

Supplement: Supplementary file 7 — Source data Fig. 1 [file 44319_2024_335_MOESM7_ESM.zip › Figure 1/1E/ATRA_16.tif]

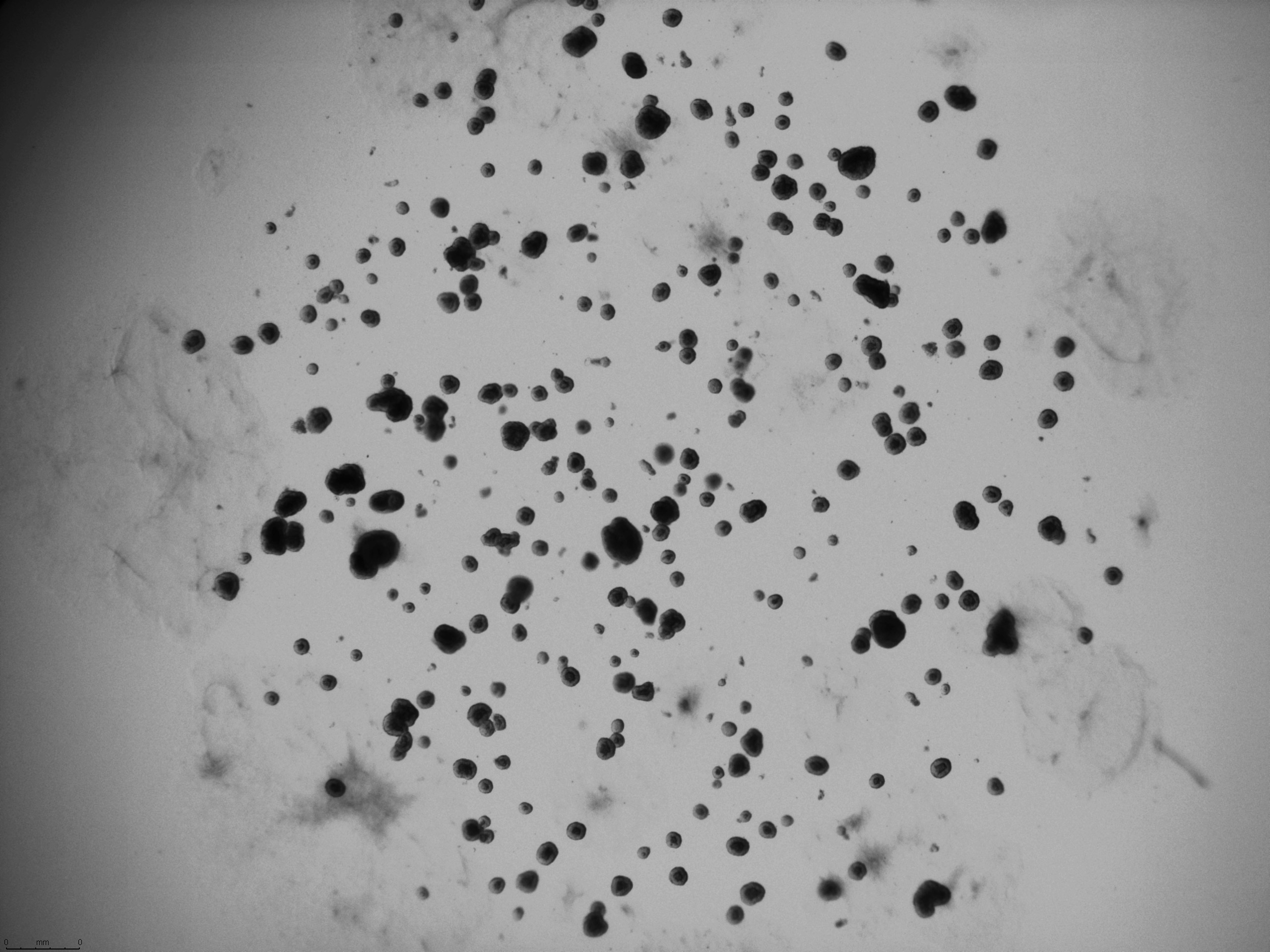

Supplement: Supplementary file 7 — Source data Fig. 1 [file 44319_2024_335_MOESM7_ESM.zip › Figure 1/1E/ATRA_0.tif]

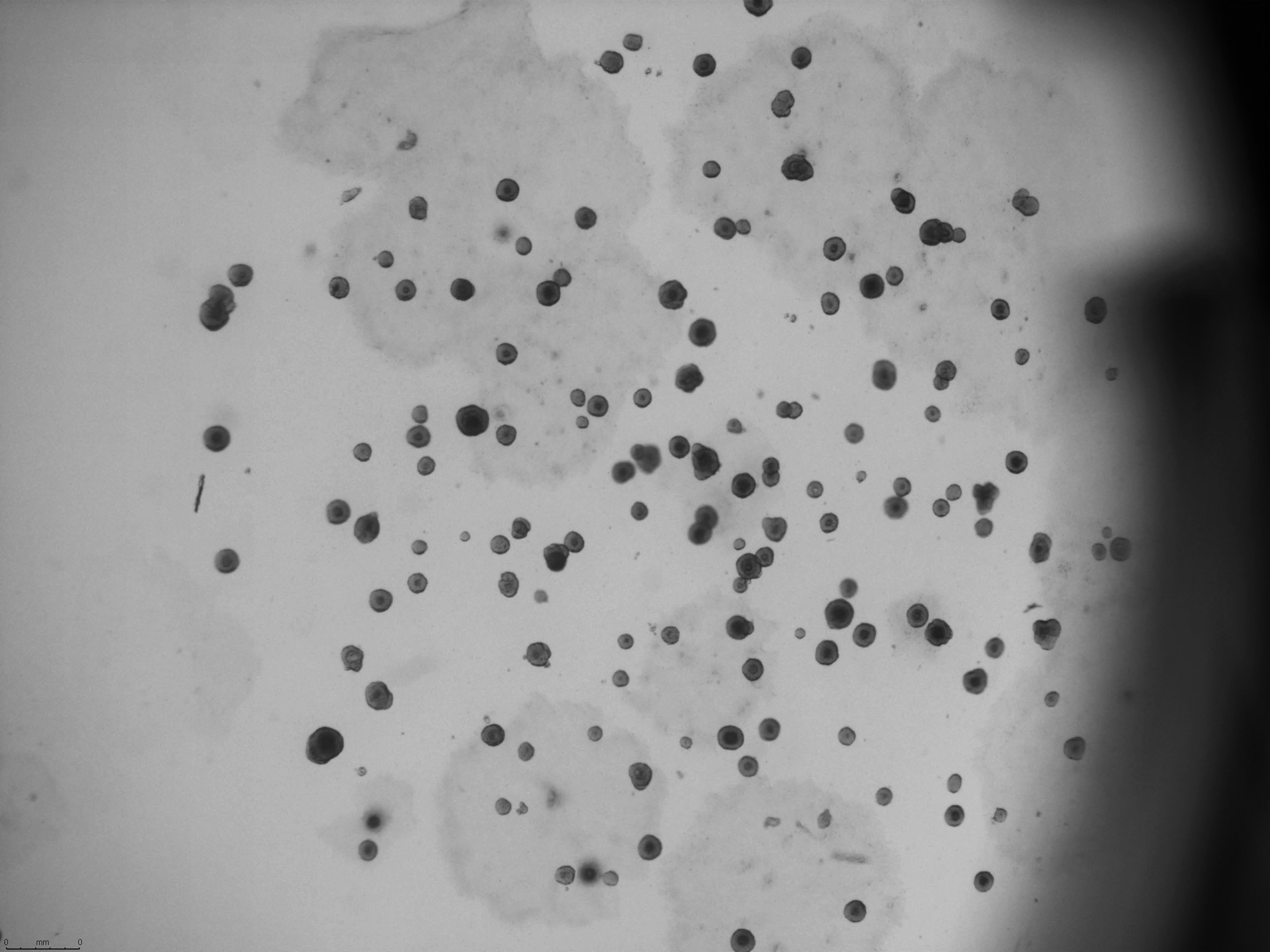

Supplement: Supplementary file 7 — Source data Fig. 1 [file 44319_2024_335_MOESM7_ESM.zip › Figure 1/1D/B27_0.tif]

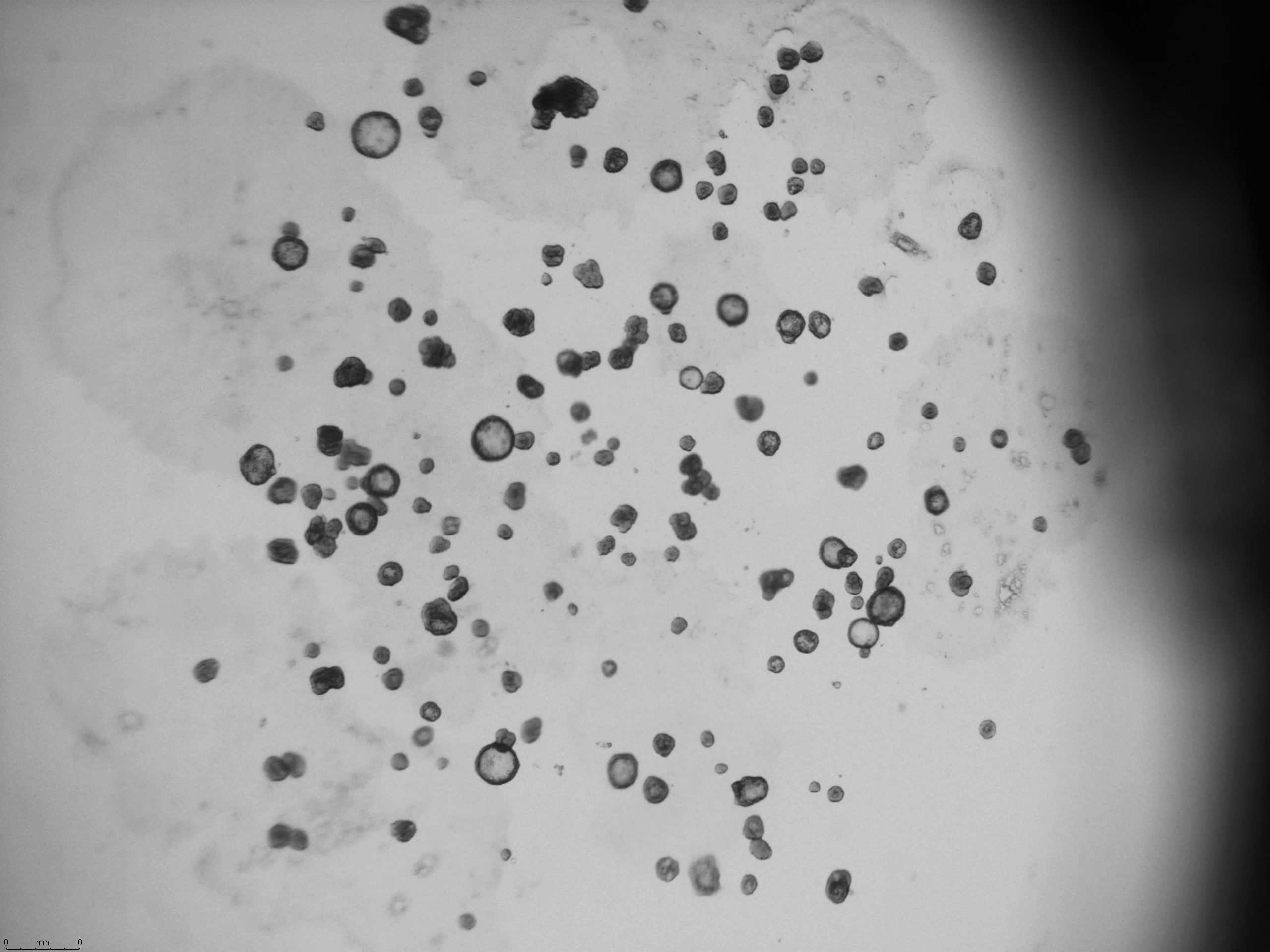

Supplement: Supplementary file 7 — Source data Fig. 1 [file 44319_2024_335_MOESM7_ESM.zip › Figure 1/1D/B27.tif]

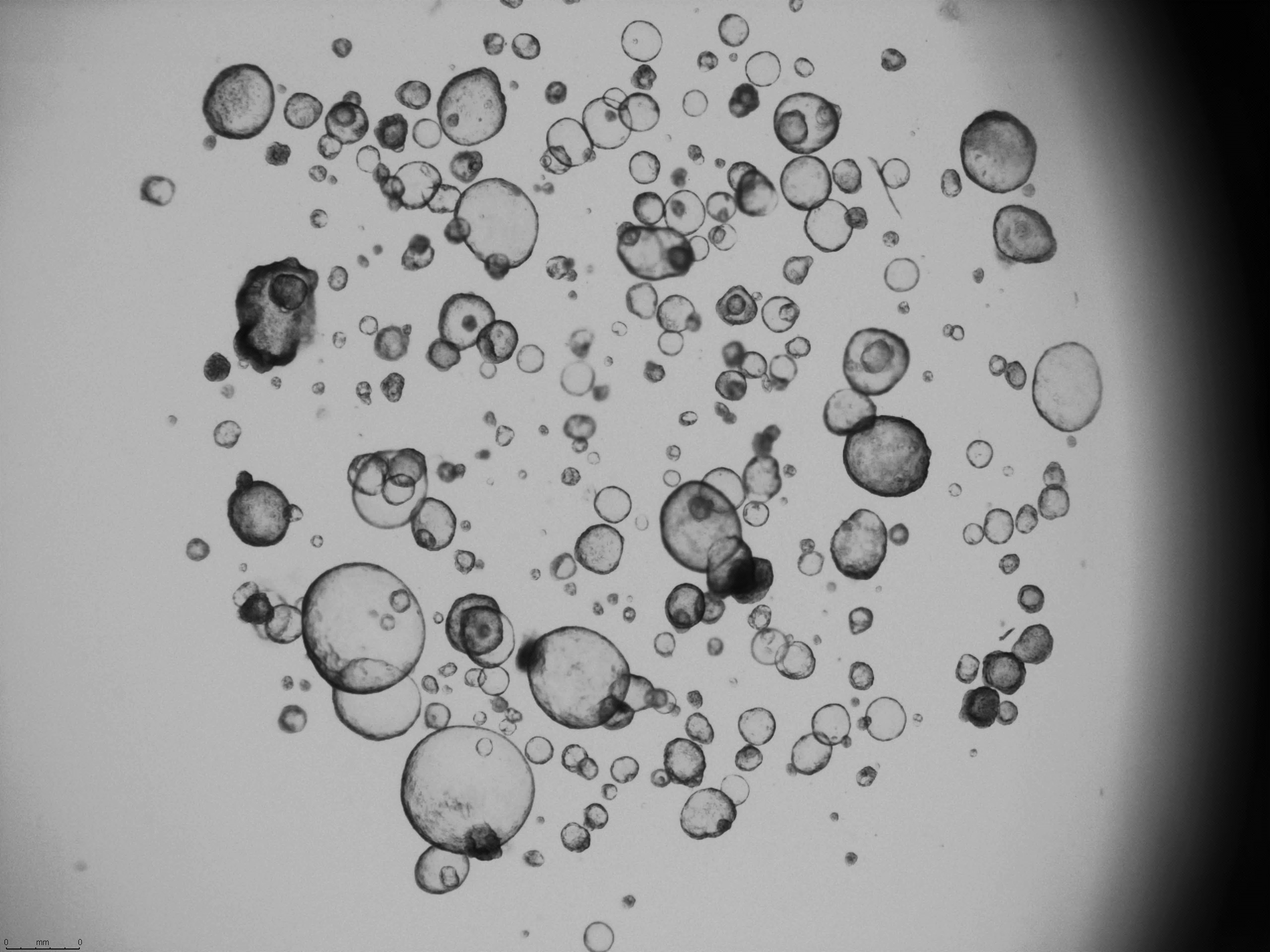

Supplement: Supplementary file 7 — Source data Fig. 1 [file 44319_2024_335_MOESM7_ESM.zip › Figure 1/1D/B27_plus.tif]

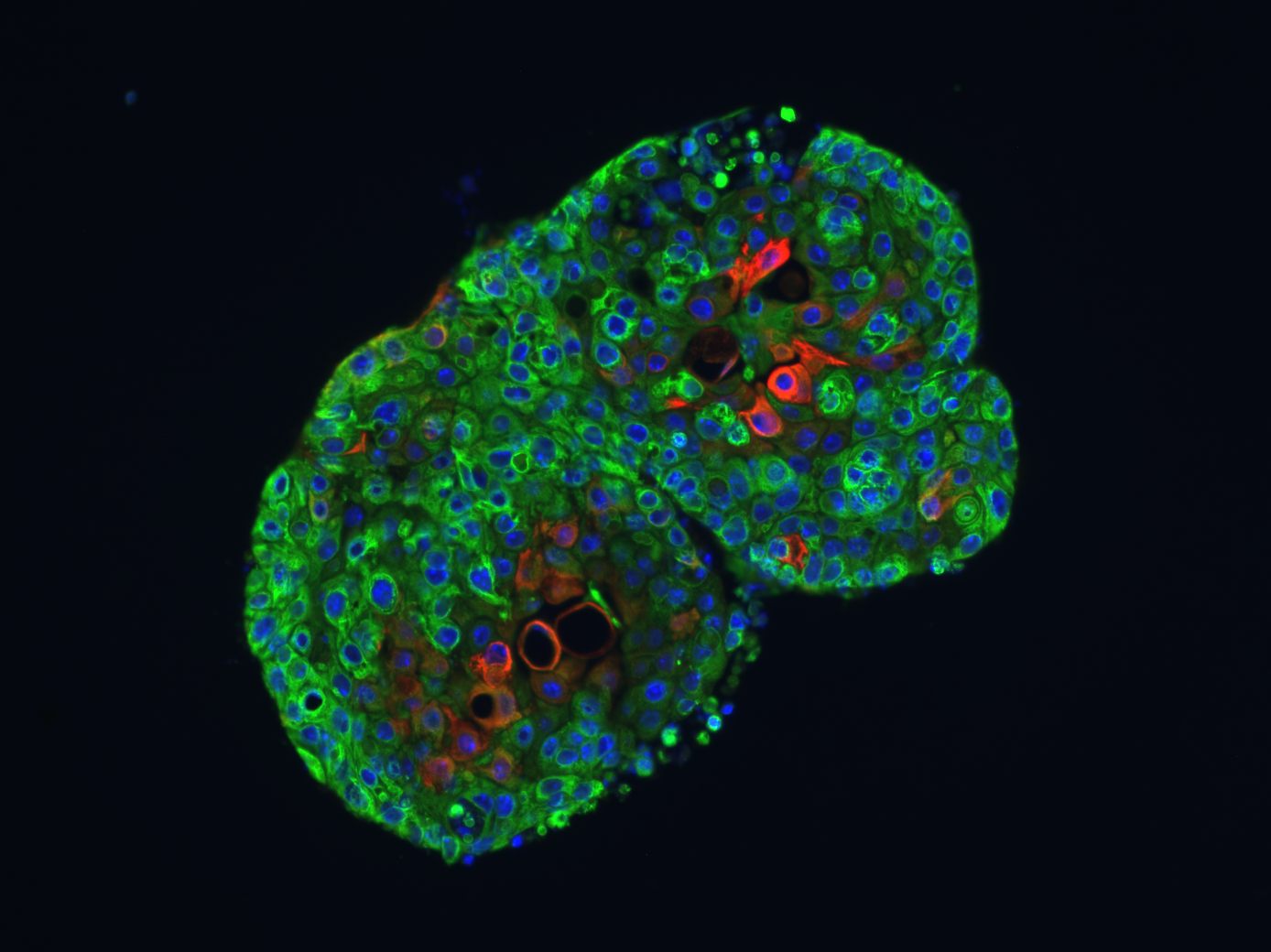

Supplement: Supplementary file 7 — Source data Fig. 1 [file 44319_2024_335_MOESM7_ESM.zip › Figure 1/1C/SPHEROID mPrOs_4 ENRAD- Ck5_v Ck8_r 20x 1_(c1+c2+c3).TIF]

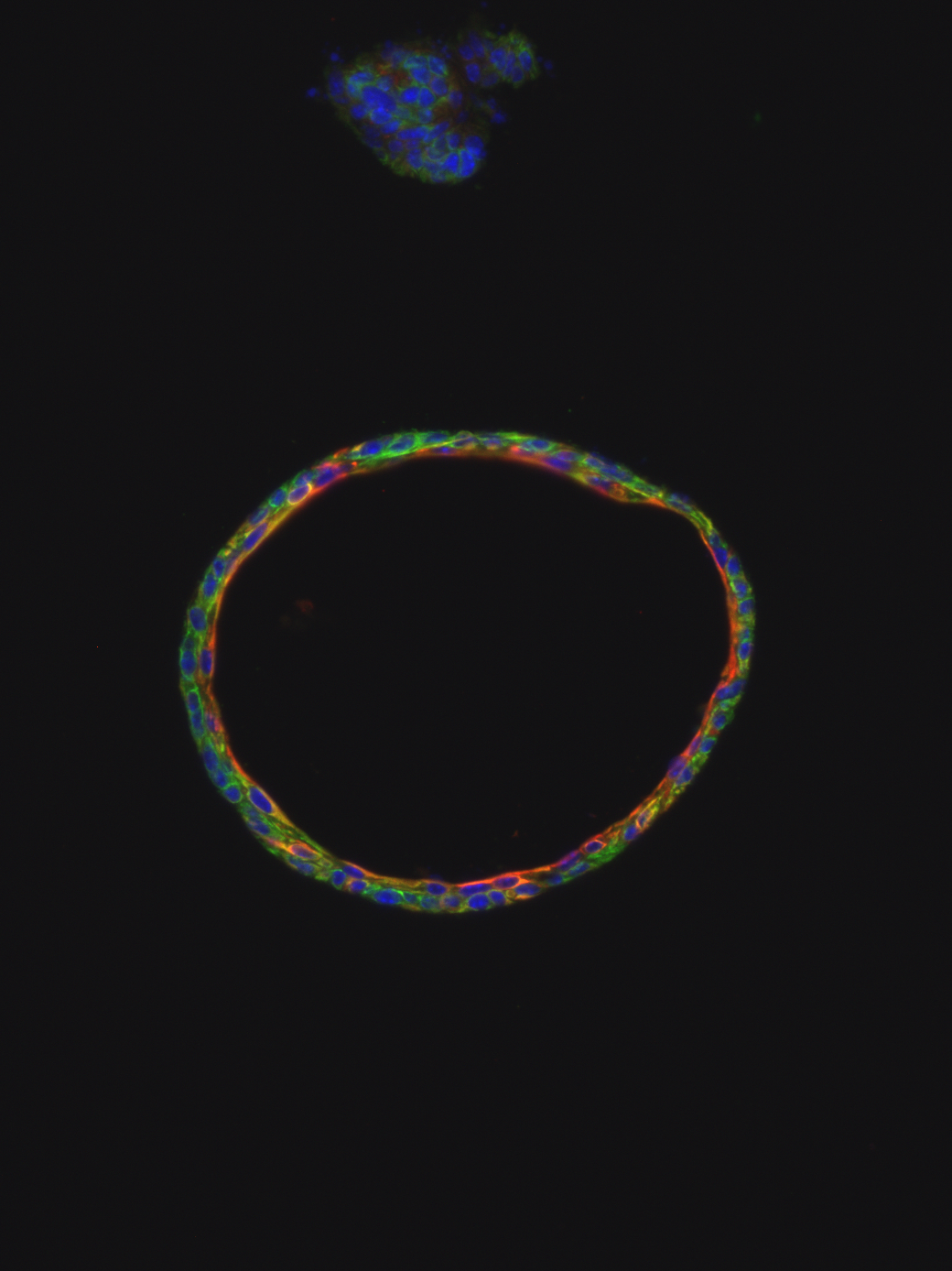

Supplement: Supplementary file 7 — Source data Fig. 1 [file 44319_2024_335_MOESM7_ESM.zip › Figure 1/1C/mPrOs ENRADA Ck5_v Ck8-18_r 001B 210918-0030_(c1+c2+c3).TIF]

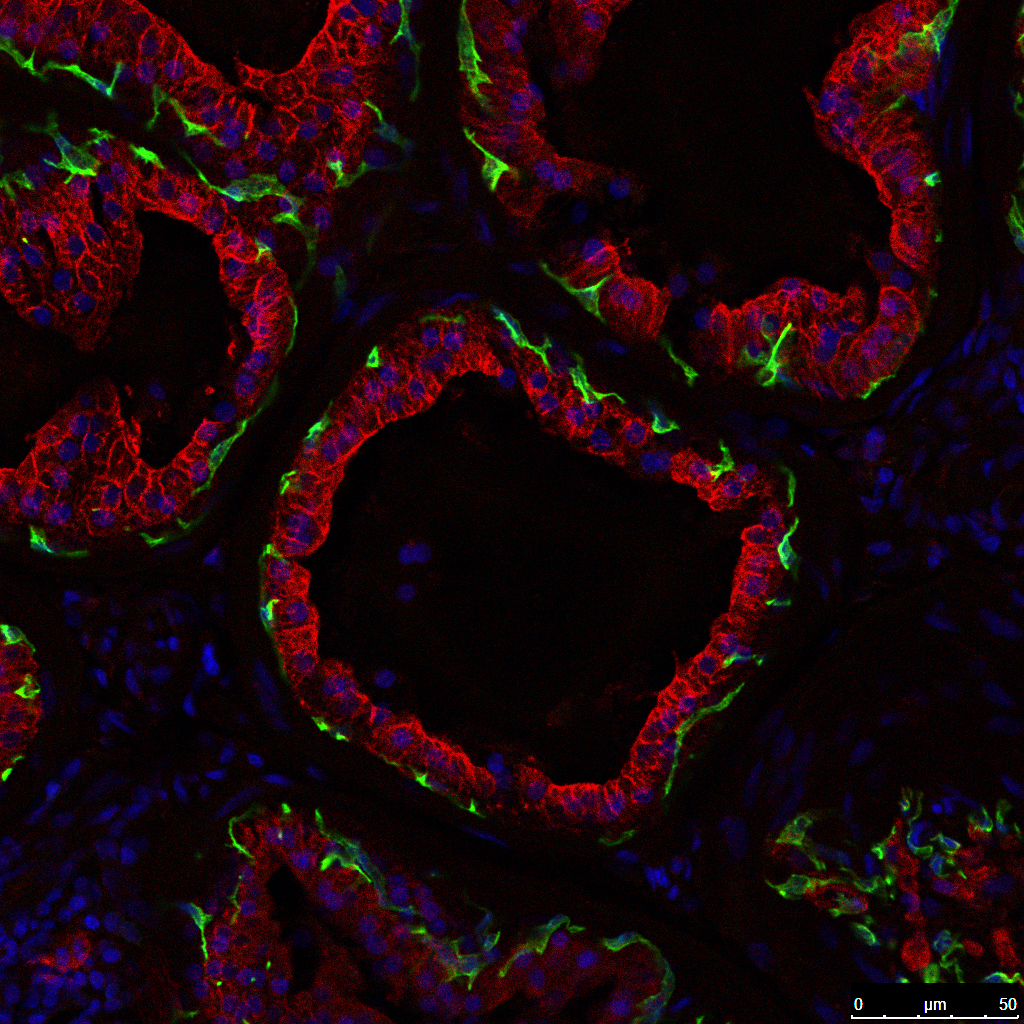

Supplement: Supplementary file 7 — Source data Fig. 1 [file 44319_2024_335_MOESM7_ESM.zip › Figure 1/1C/MOUSE PROSTATE Experiment_mProstate VP CD1 Ck5v Ck8_18r MERGE.tif]

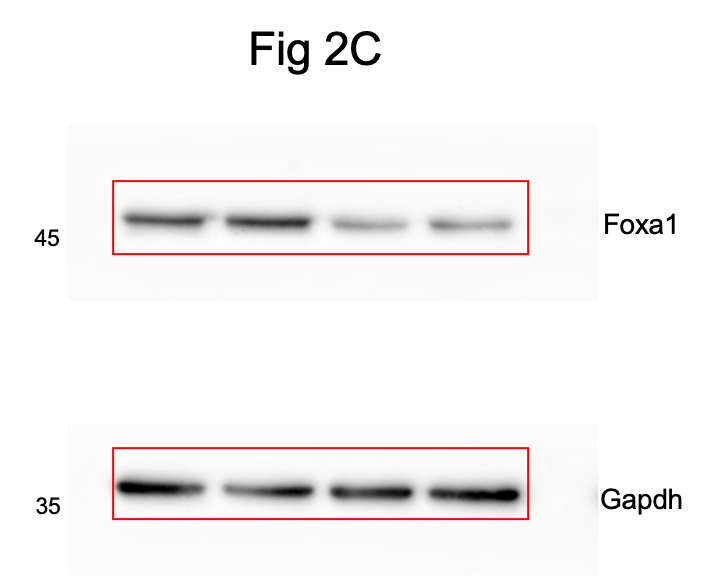

Supplement: Supplementary file 8 — Source data Fig. 2 [file 44319_2024_335_MOESM8_ESM.zip › Figure 2/2C/2C.png]

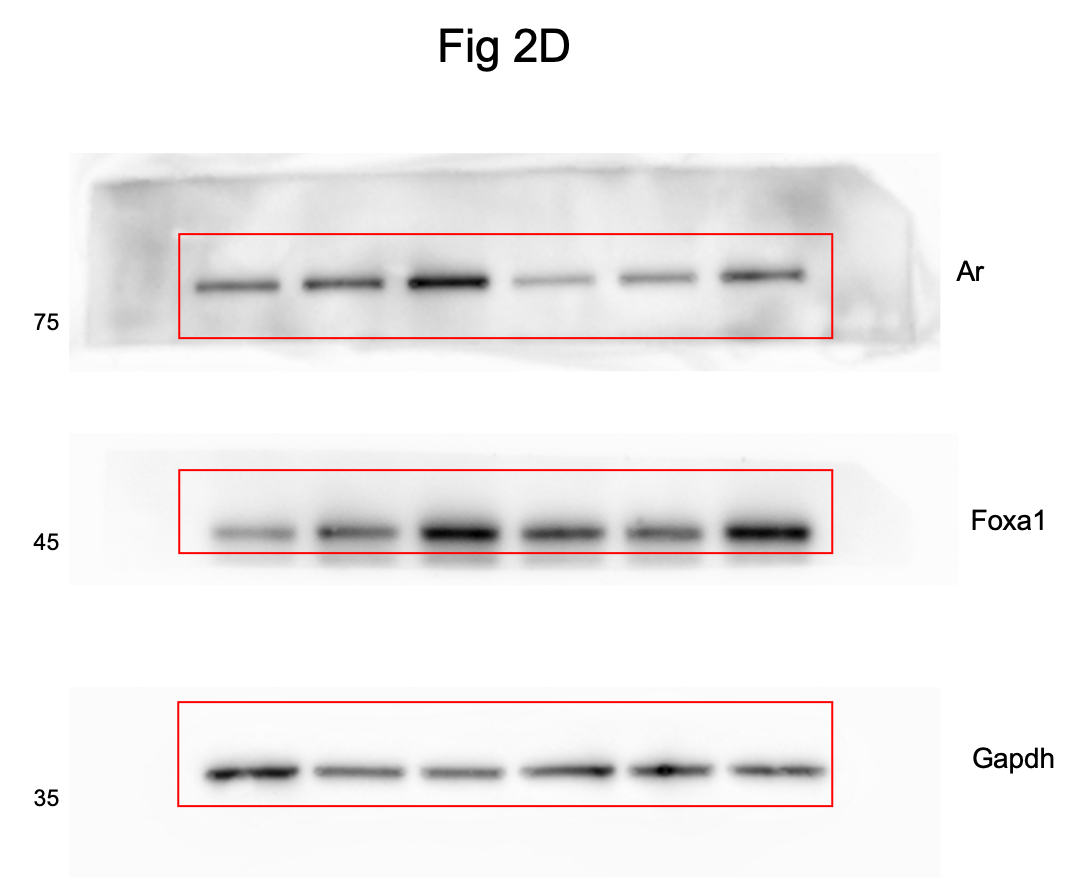

Supplement: Supplementary file 8 — Source data Fig. 2 [file 44319_2024_335_MOESM8_ESM.zip › Figure 2/2D/2D.png]

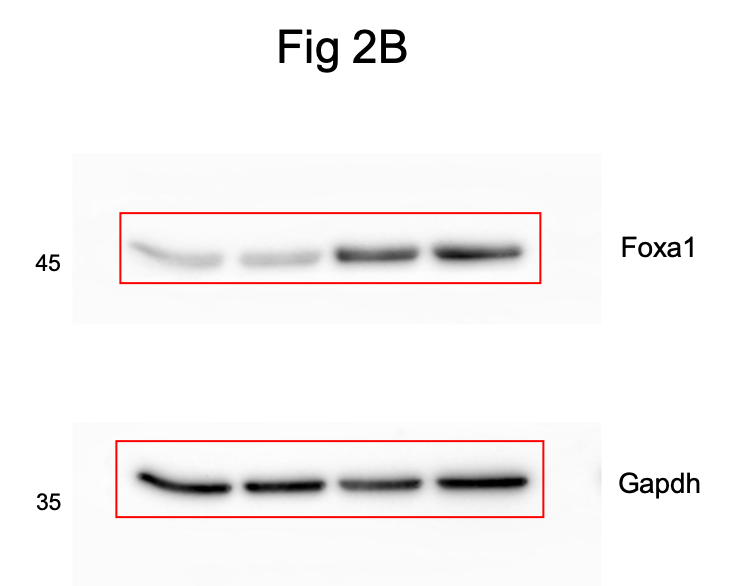

Supplement: Supplementary file 8 — Source data Fig. 2 [file 44319_2024_335_MOESM8_ESM.zip › Figure 2/2B/Fig2B.png]

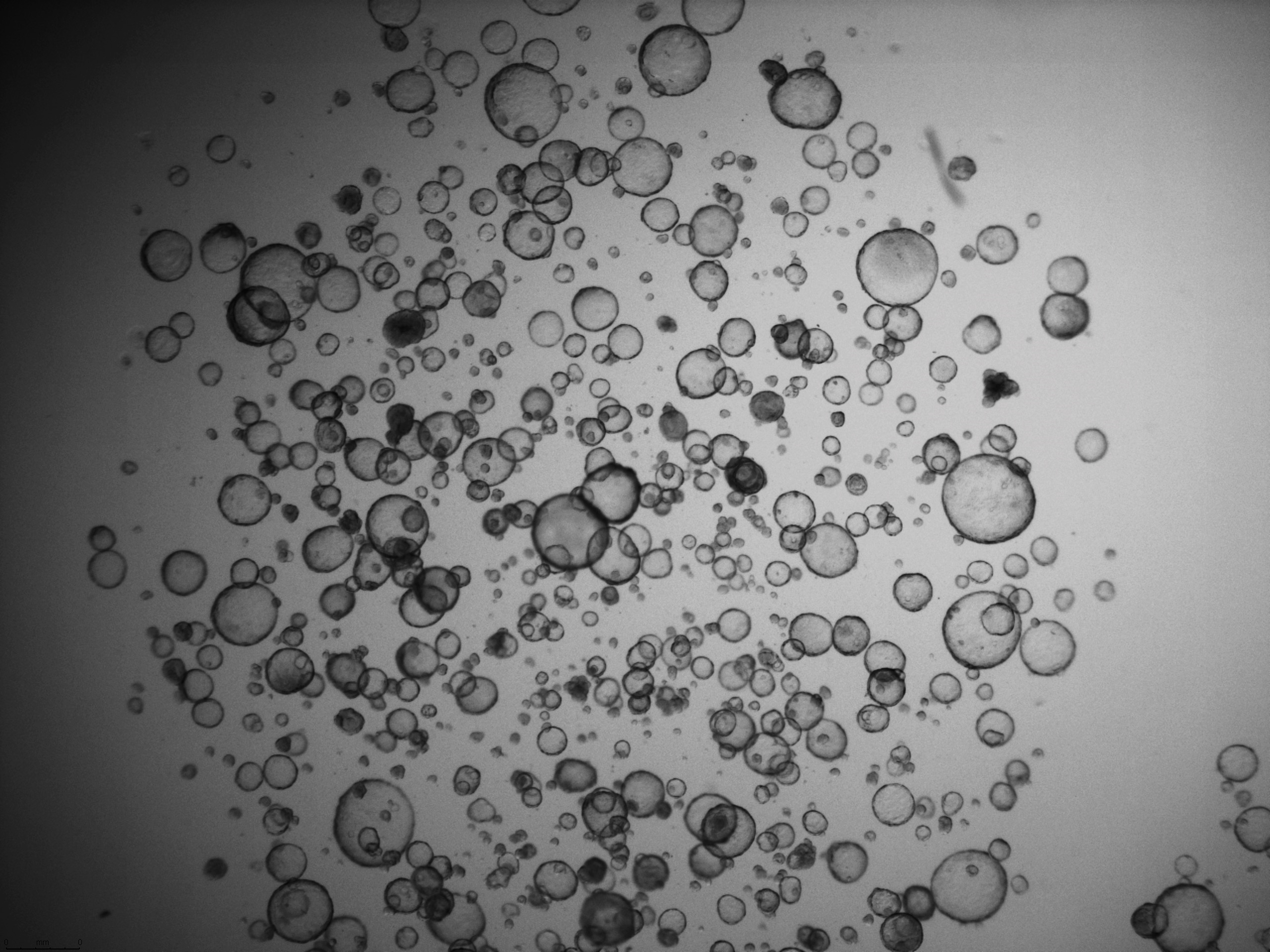

Supplement: Supplementary file 9 — Source data Fig. 3 [file 44319_2024_335_MOESM9_ESM.zip › Figure 3/3E/2i shCntrl.tif]

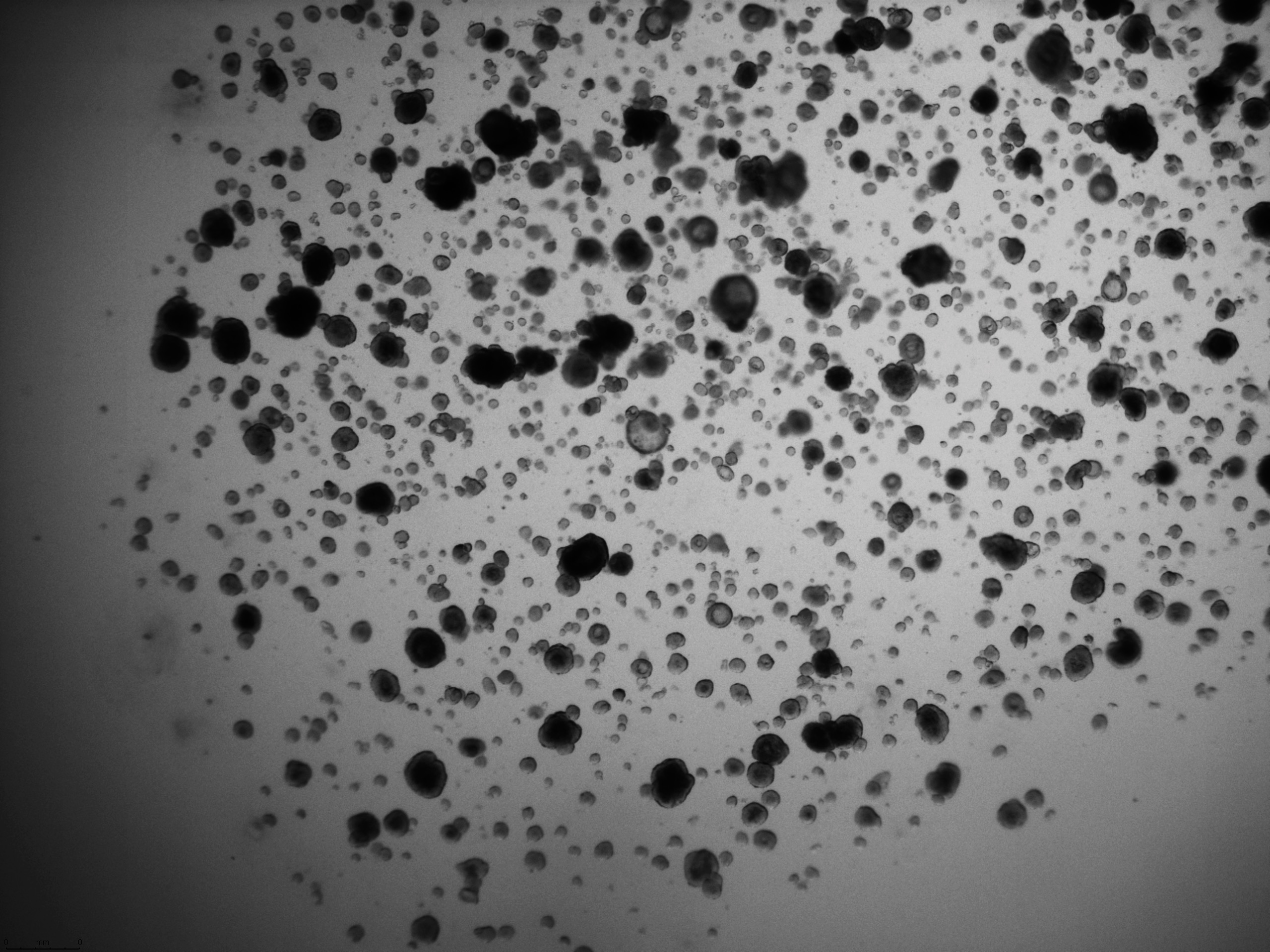

Supplement: Supplementary file 9 — Source data Fig. 3 [file 44319_2024_335_MOESM9_ESM.zip › Figure 3/3E/2I shFOXA1.tif]

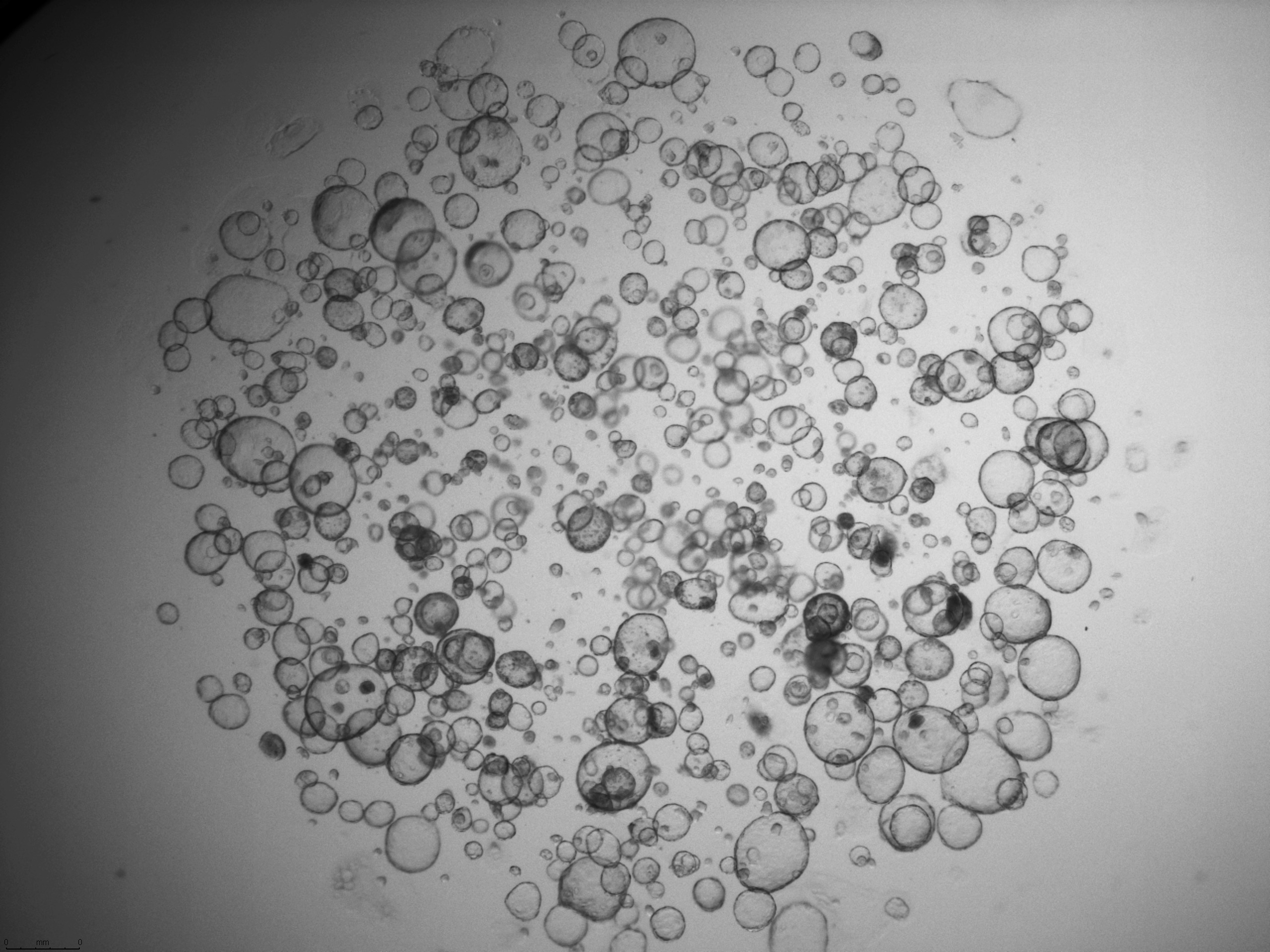

Supplement: Supplementary file 9 — Source data Fig. 3 [file 44319_2024_335_MOESM9_ESM.zip › Figure 3/3E/2i Untr.tif]

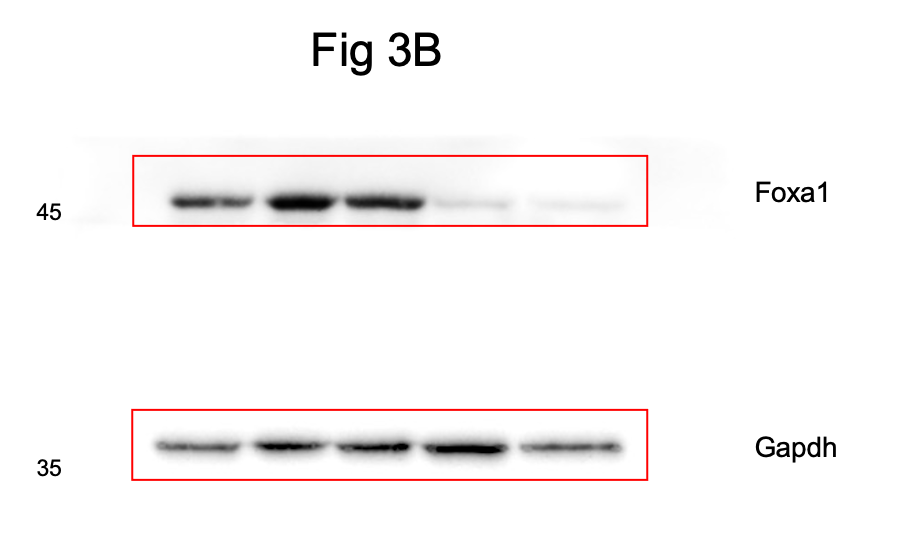

Supplement: Supplementary file 9 — Source data Fig. 3 [file 44319_2024_335_MOESM9_ESM.zip › Figure 3/3B/3B.png]

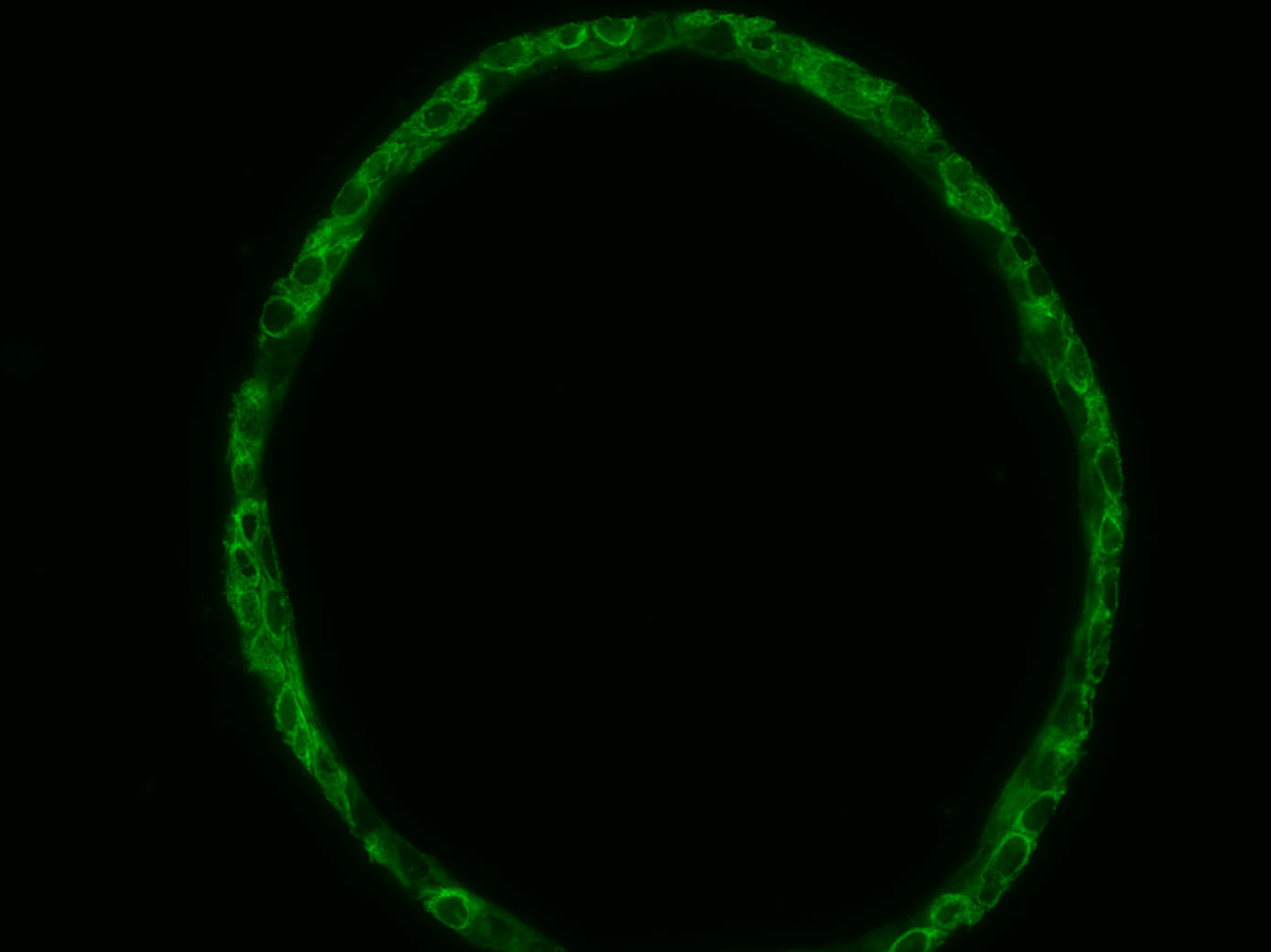

Supplement: Supplementary file 9 — Source data Fig. 3 [file 44319_2024_335_MOESM9_ESM.zip › Figure 3/3J/ENRAD- FOXA1 Ck5.tif]

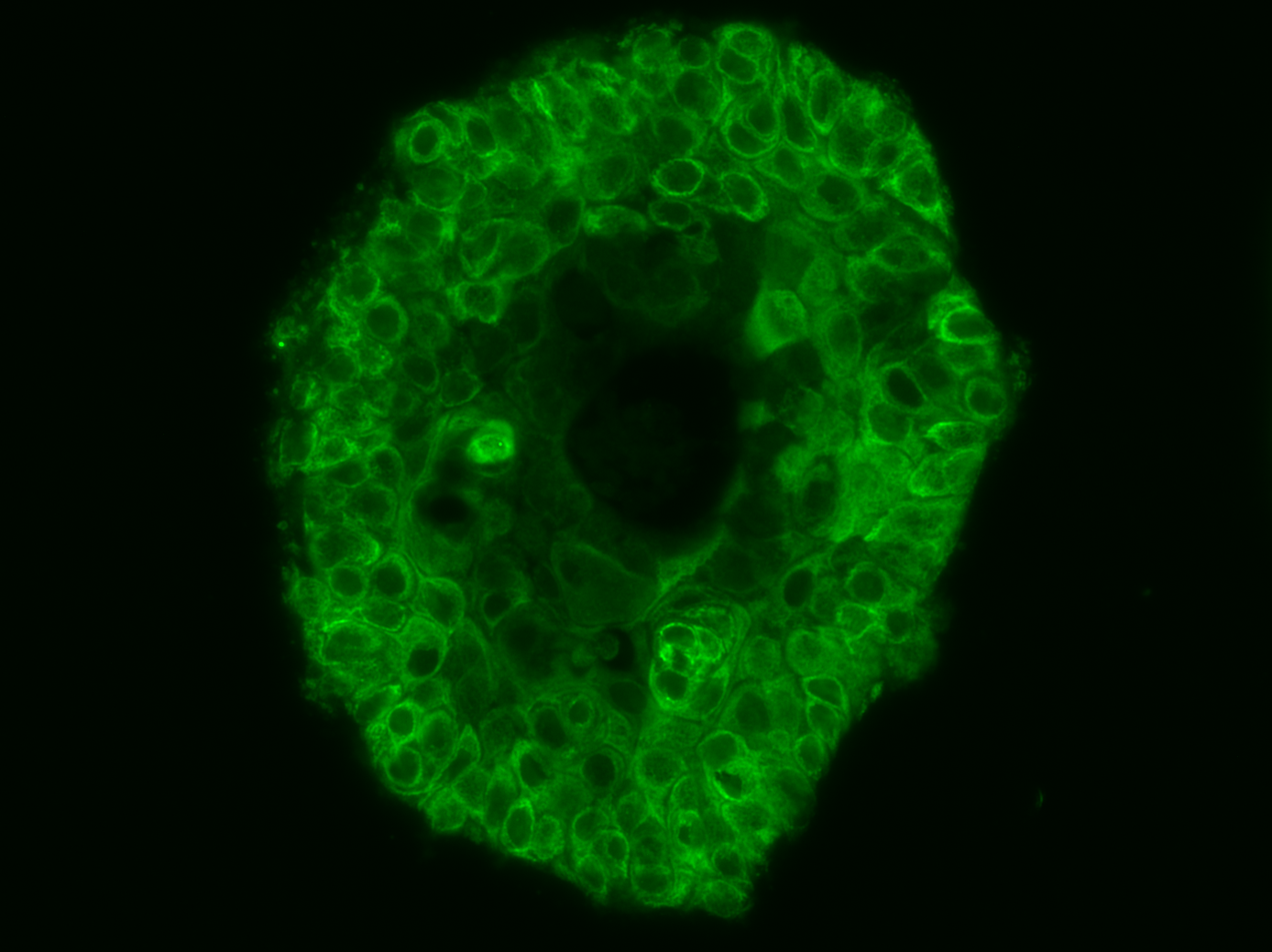

Supplement: Supplementary file 9 — Source data Fig. 3 [file 44319_2024_335_MOESM9_ESM.zip › Figure 3/3J/ENRA-- FOXA1 Ck5.tif]

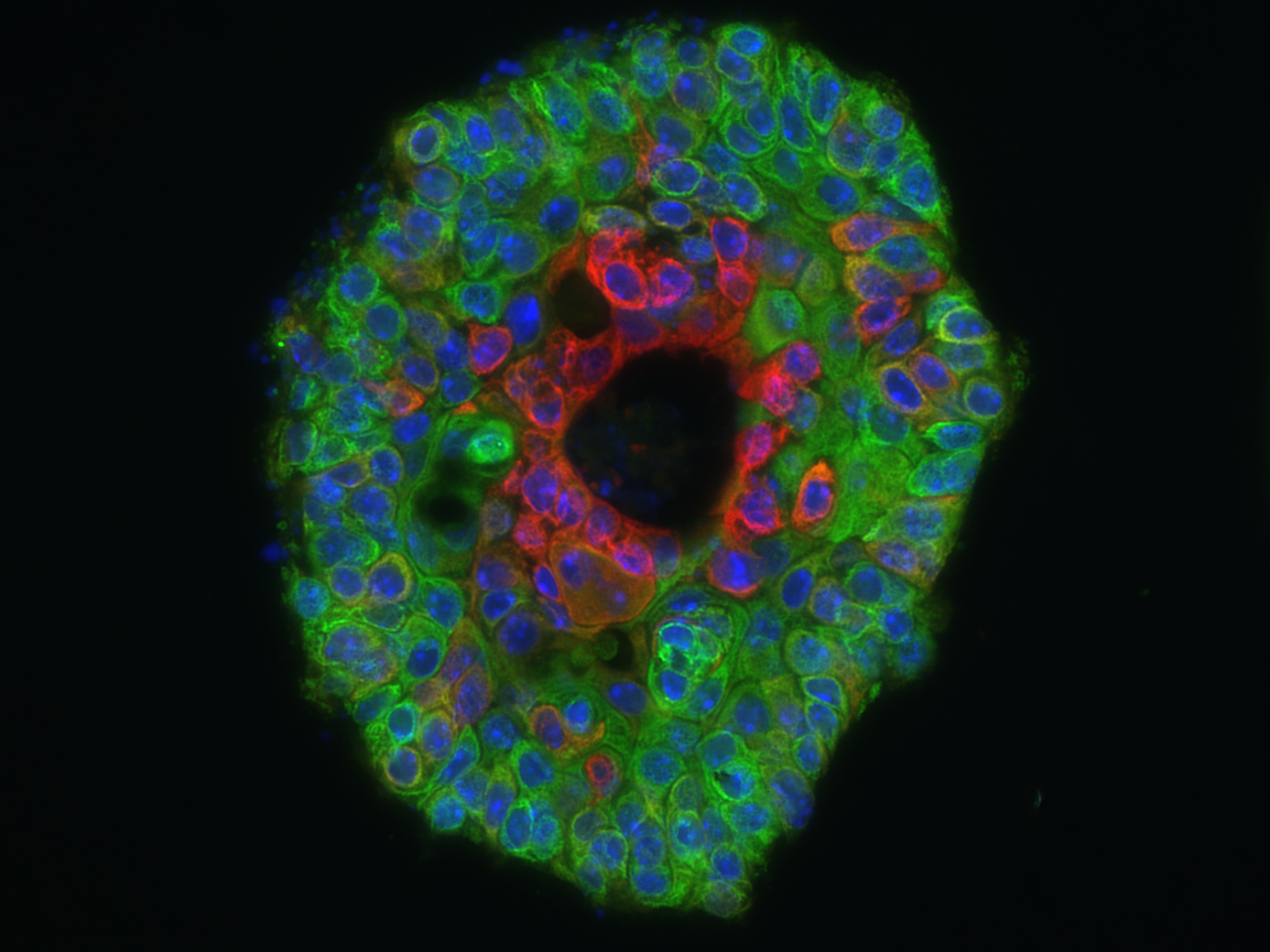

Supplement: Supplementary file 9 — Source data Fig. 3 [file 44319_2024_335_MOESM9_ESM.zip › Figure 3/3J/ENRA-- FOXA1 merge.tif]

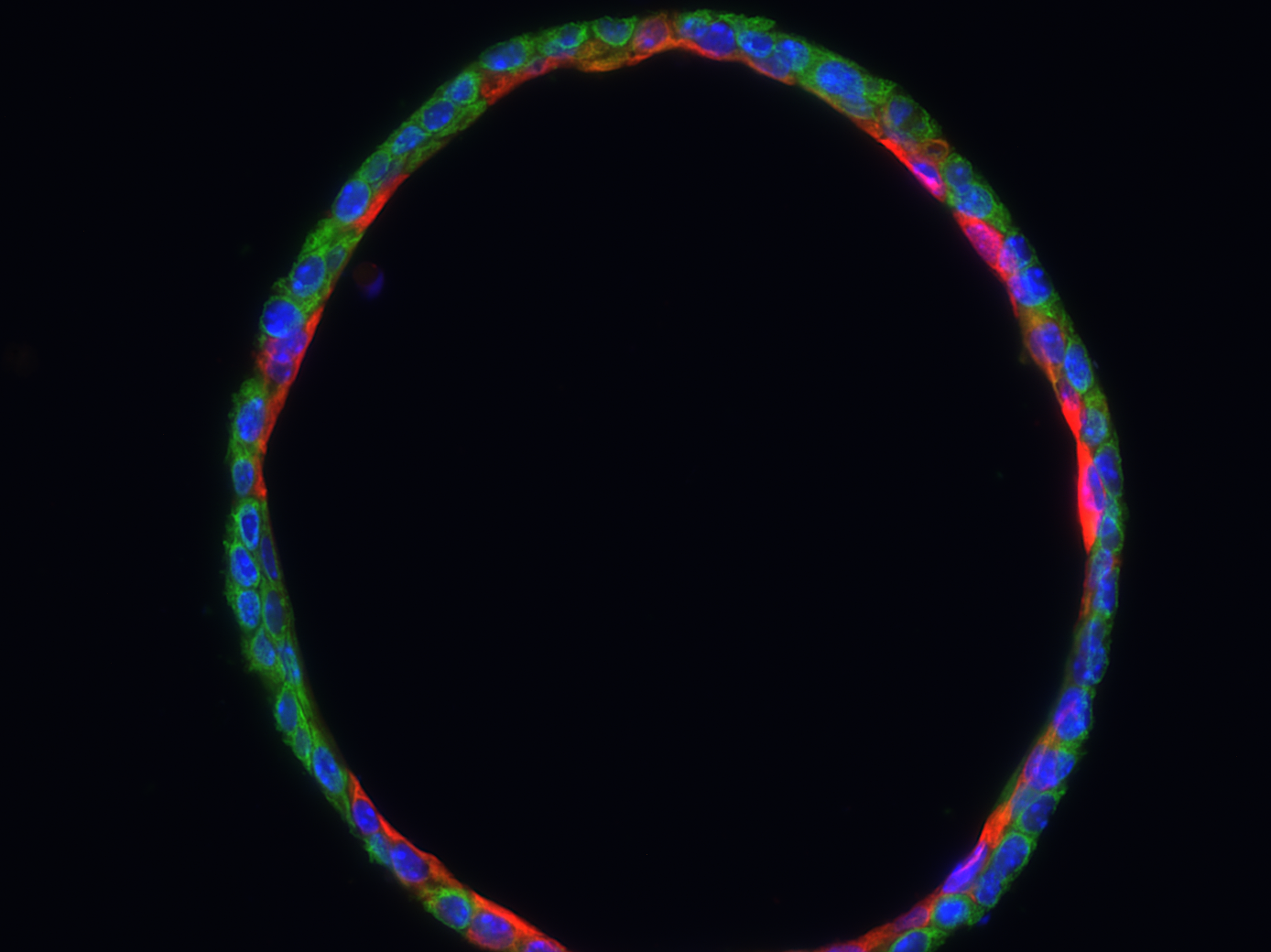

Supplement: Supplementary file 9 — Source data Fig. 3 [file 44319_2024_335_MOESM9_ESM.zip › Figure 3/3J/ENRAD- FOXA1 merge.tif]

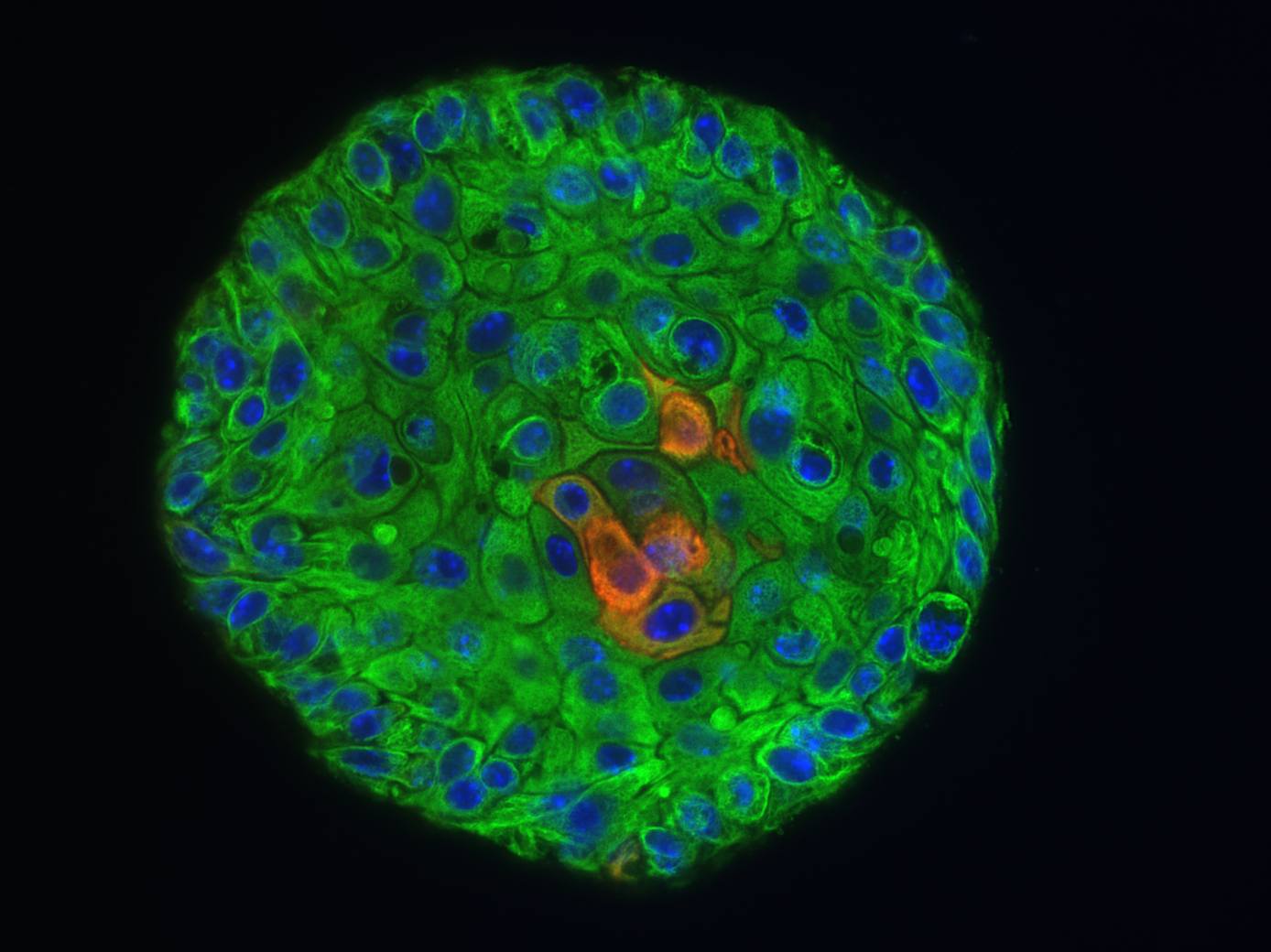

Supplement: Supplementary file 9 — Source data Fig. 3 [file 44319_2024_335_MOESM9_ESM.zip › Figure 3/3J/ENRAD- EV merge.tif]

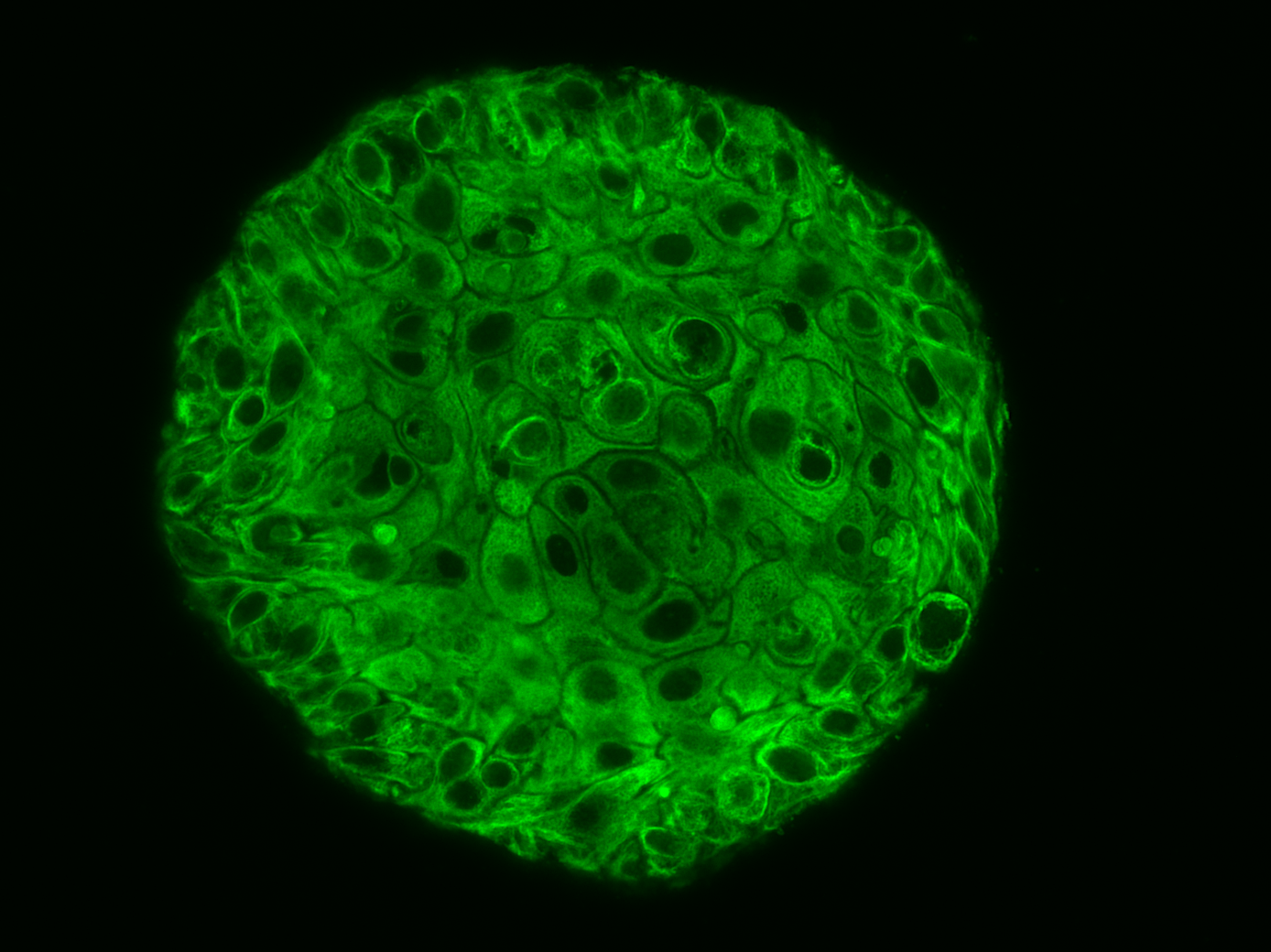

Supplement: Supplementary file 9 — Source data Fig. 3 [file 44319_2024_335_MOESM9_ESM.zip › Figure 3/3J/ENRAD- EV Ck5.tif]

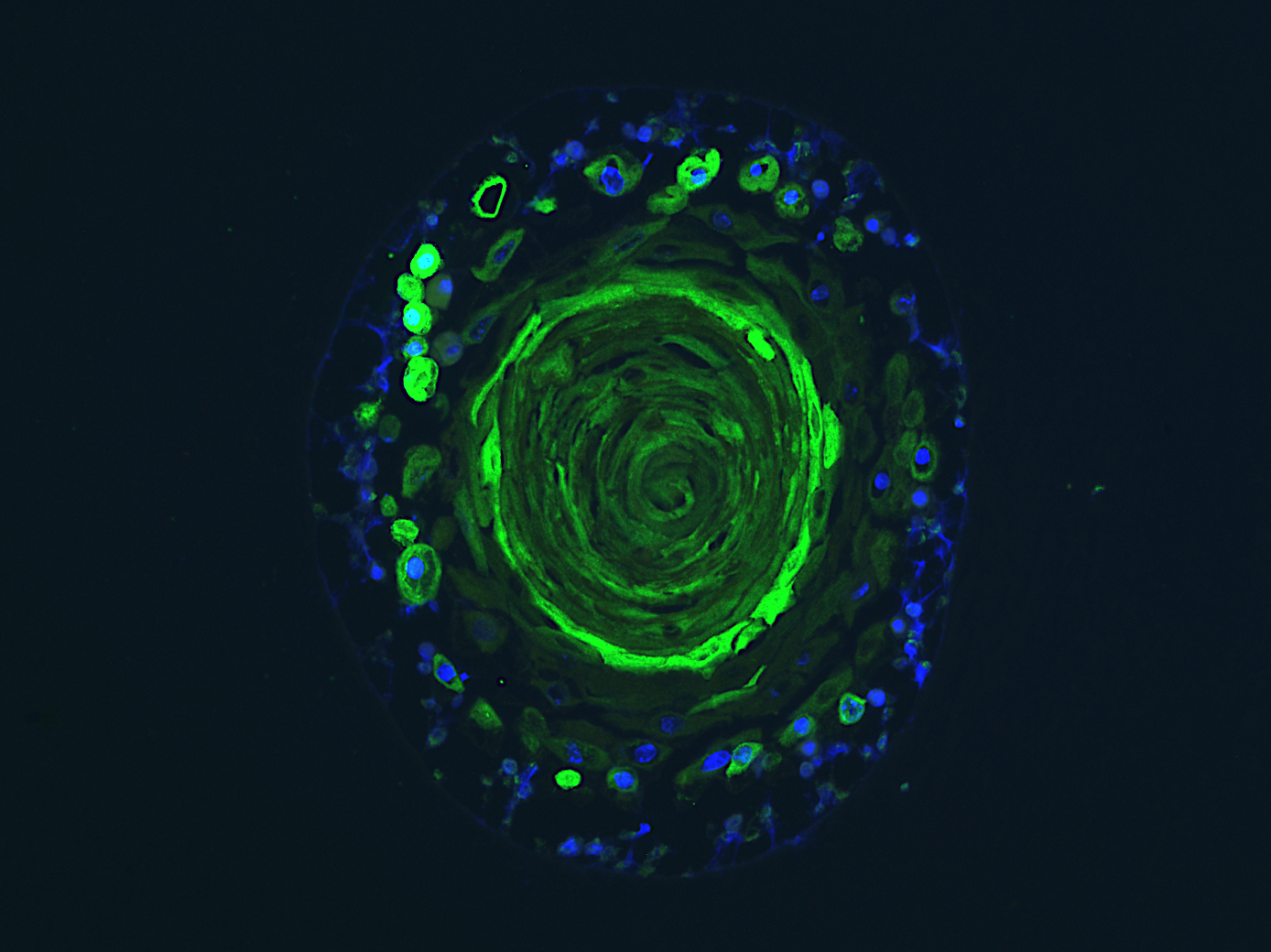

Supplement: Supplementary file 9 — Source data Fig. 3 [file 44319_2024_335_MOESM9_ESM.zip › Figure 3/3J/ENRA-- EV merge.tif]

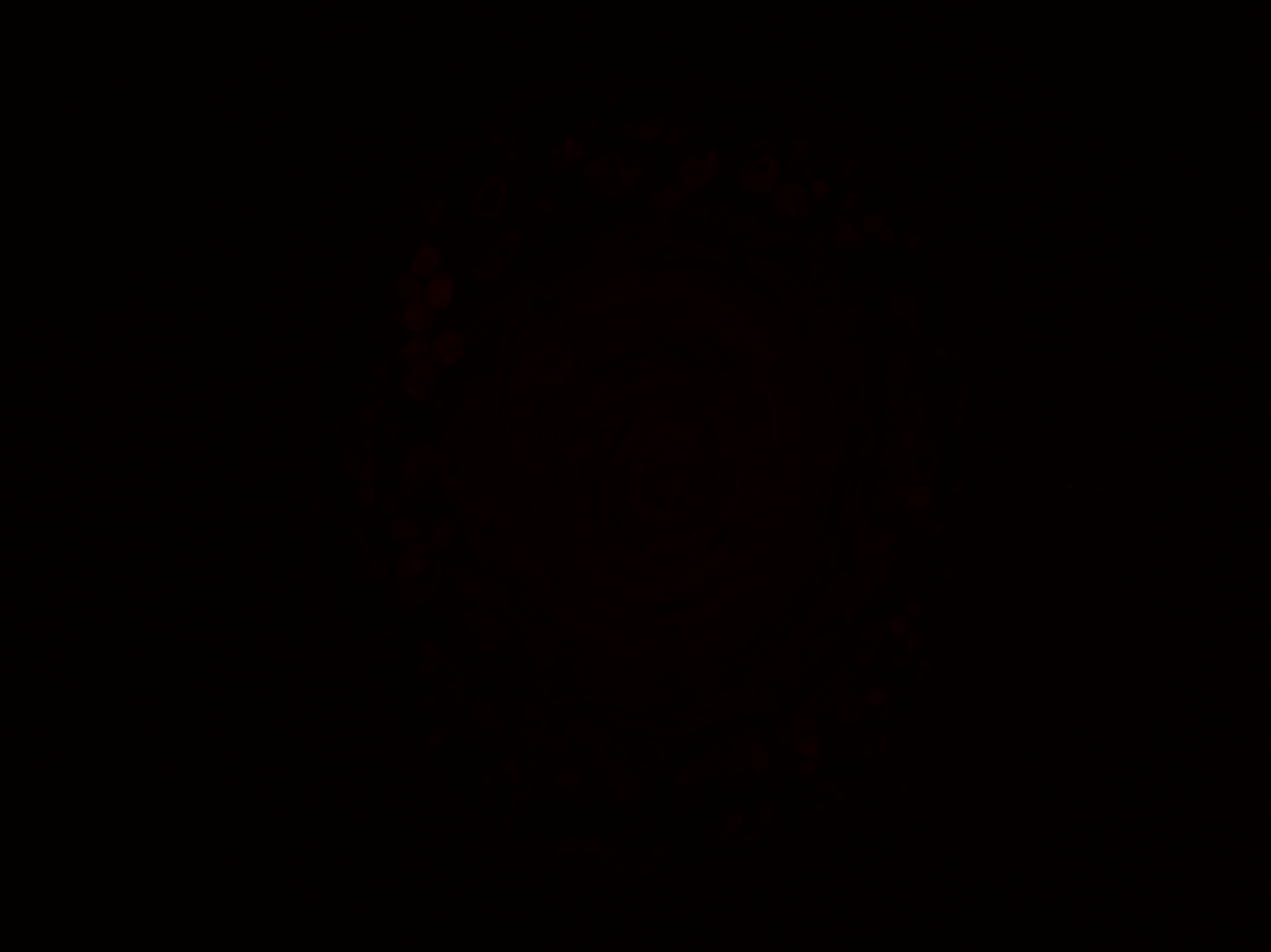

Supplement: Supplementary file 9 — Source data Fig. 3 [file 44319_2024_335_MOESM9_ESM.zip › Figure 3/3J/ENRA-- EV Ck8.tif]

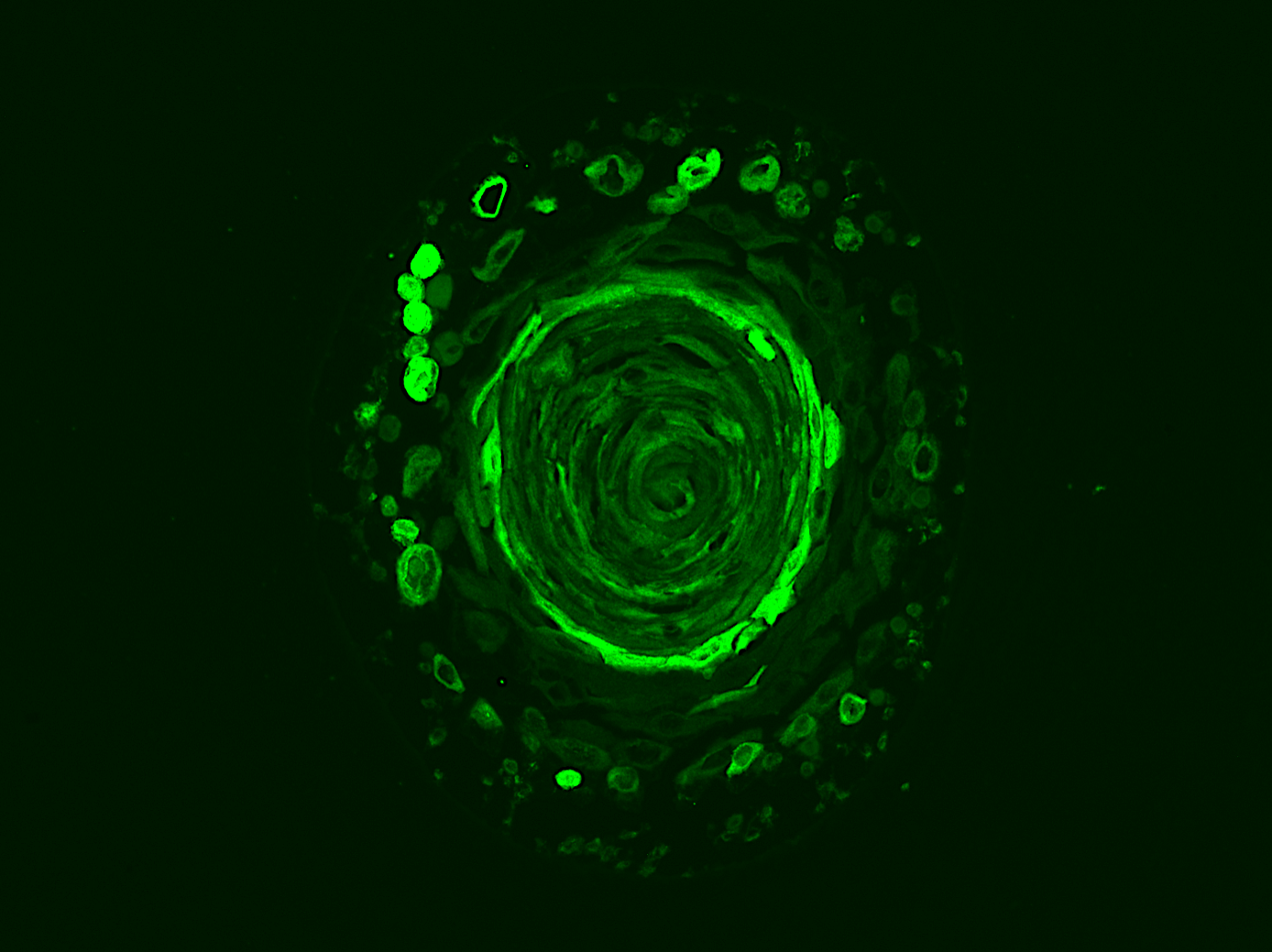

Supplement: Supplementary file 9 — Source data Fig. 3 [file 44319_2024_335_MOESM9_ESM.zip › Figure 3/3J/ENRA-- EV Ck5.tif]

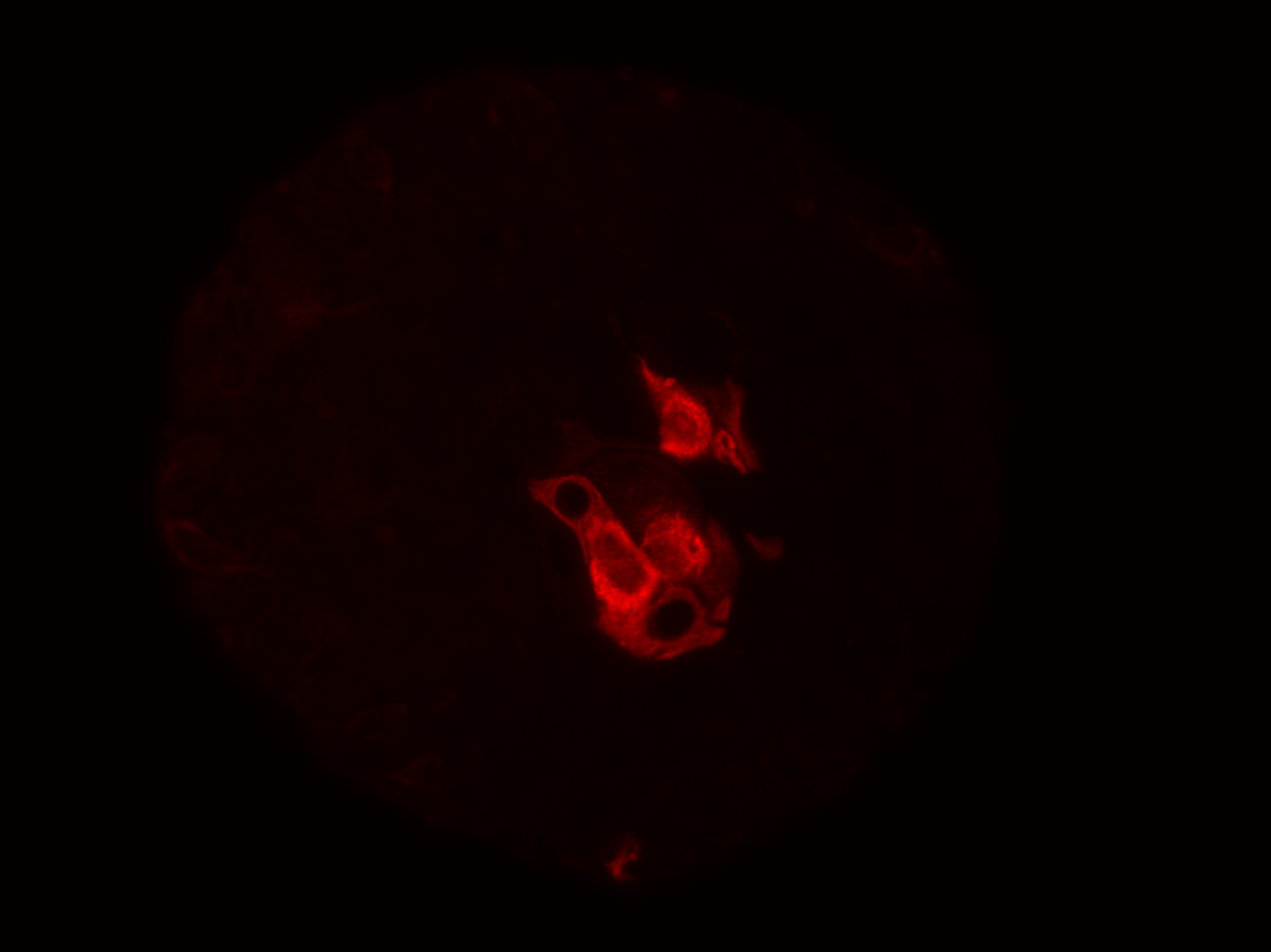

Supplement: Supplementary file 9 — Source data Fig. 3 [file 44319_2024_335_MOESM9_ESM.zip › Figure 3/3J/ENRAD- EV Ck8.tif]

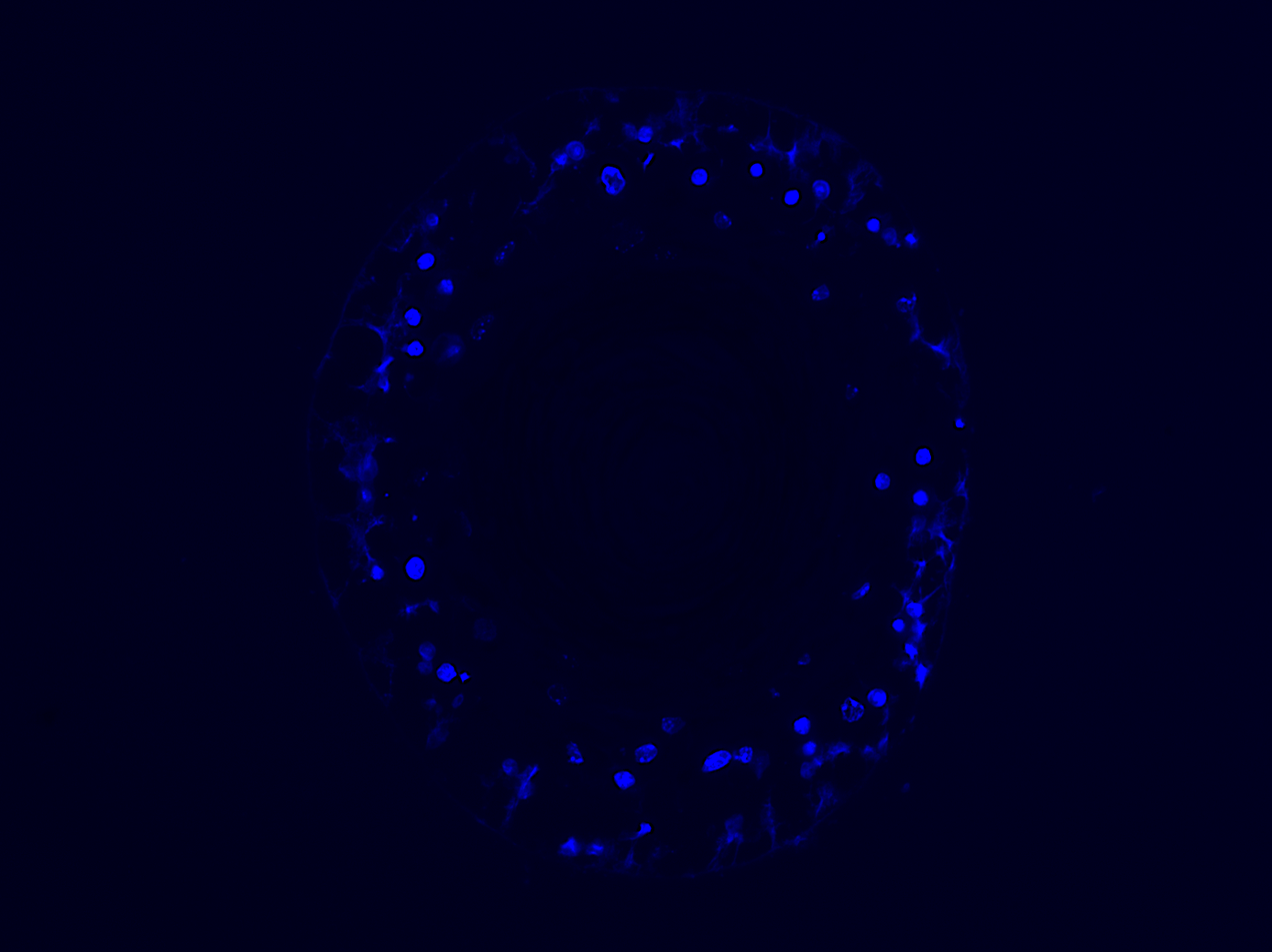

Supplement: Supplementary file 9 — Source data Fig. 3 [file 44319_2024_335_MOESM9_ESM.zip › Figure 3/3J/ENRA-- EV Dapi.tif]

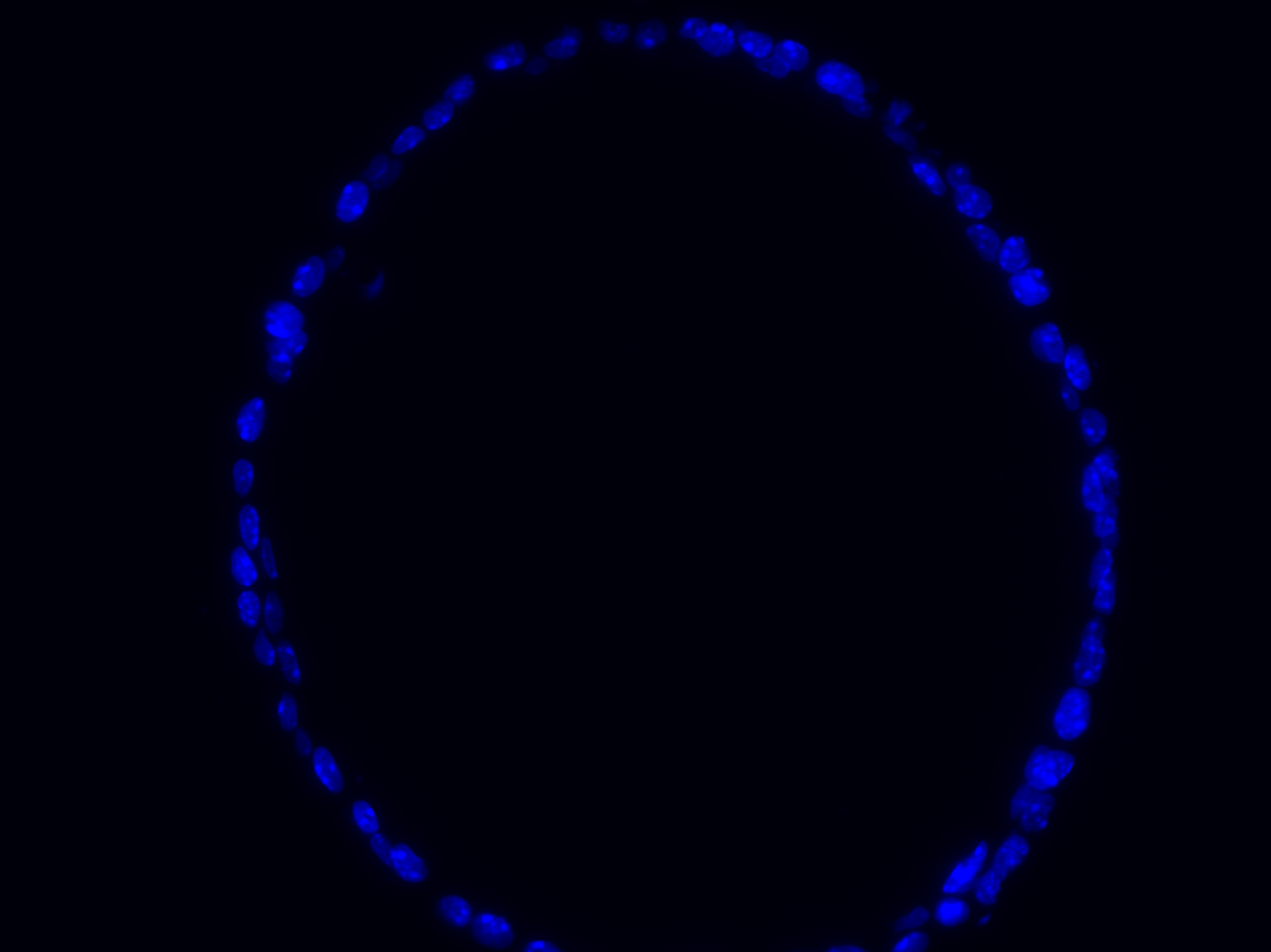

Supplement: Supplementary file 9 — Source data Fig. 3 [file 44319_2024_335_MOESM9_ESM.zip › Figure 3/3J/ENRAD- FOXA1 Dapi.tif]

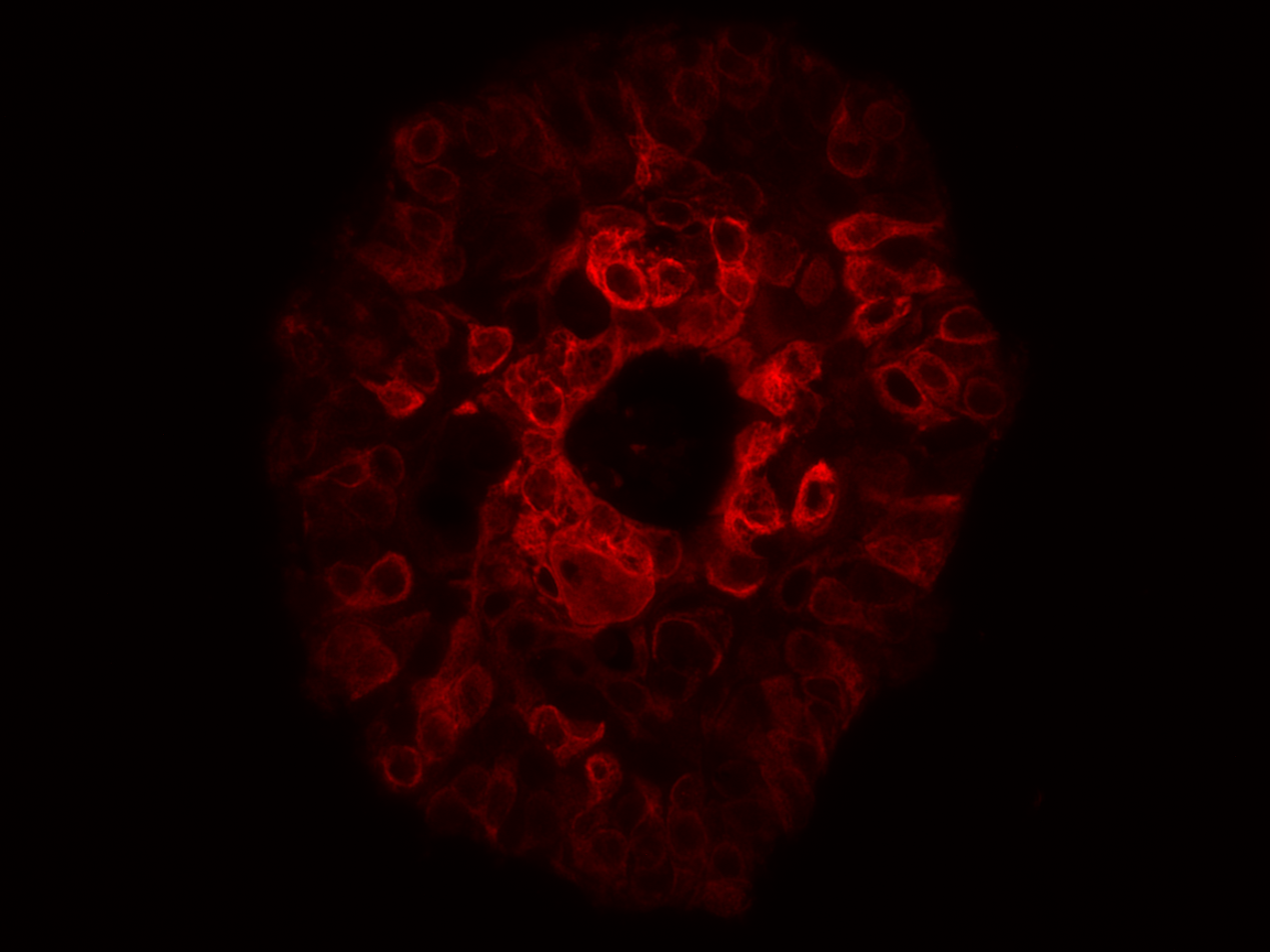

Supplement: Supplementary file 9 — Source data Fig. 3 [file 44319_2024_335_MOESM9_ESM.zip › Figure 3/3J/ENRA-- FOXA1 Ck8.tif]

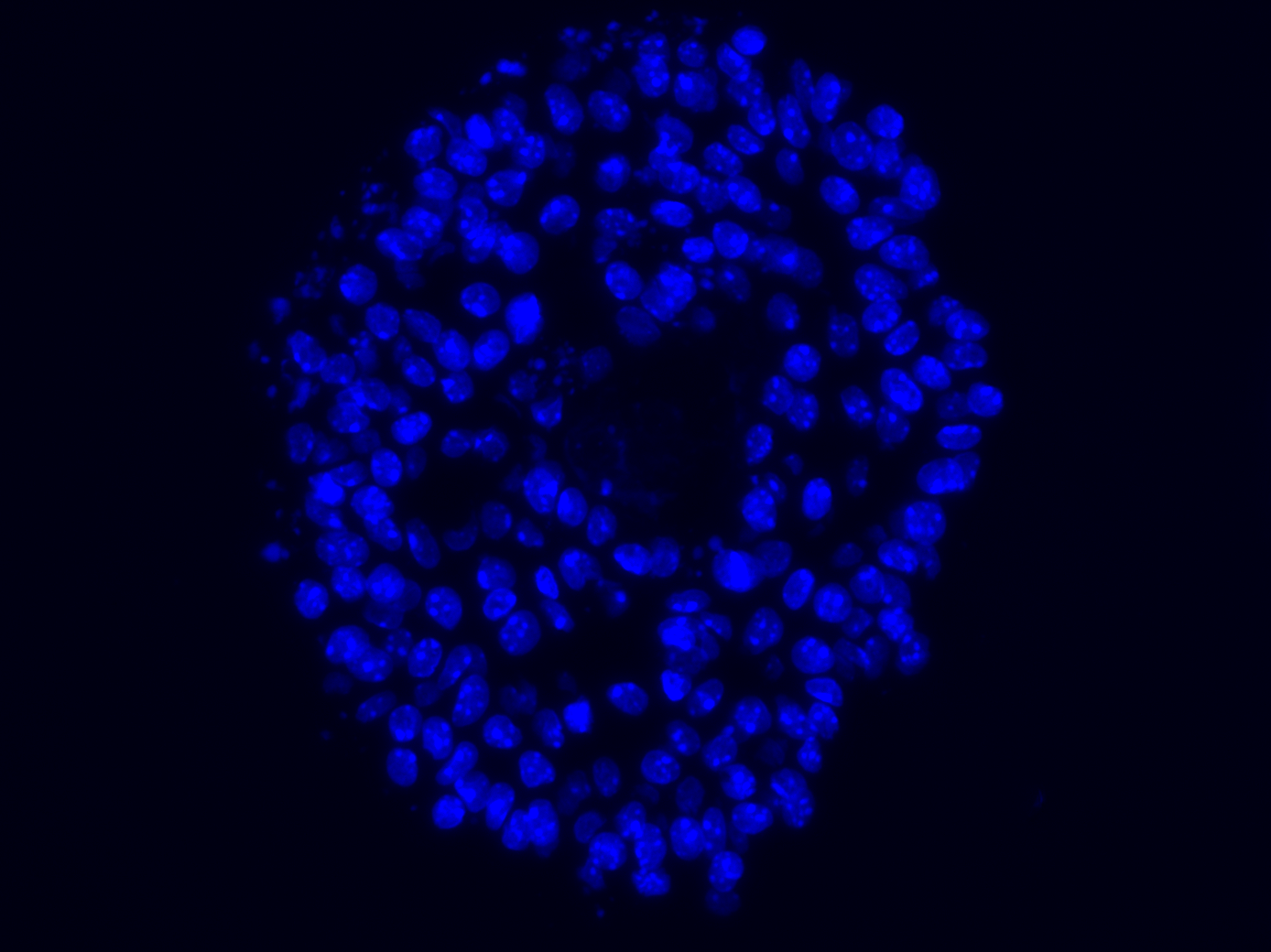

Supplement: Supplementary file 9 — Source data Fig. 3 [file 44319_2024_335_MOESM9_ESM.zip › Figure 3/3J/ENRA-- FOXA1 Dapi.tif]

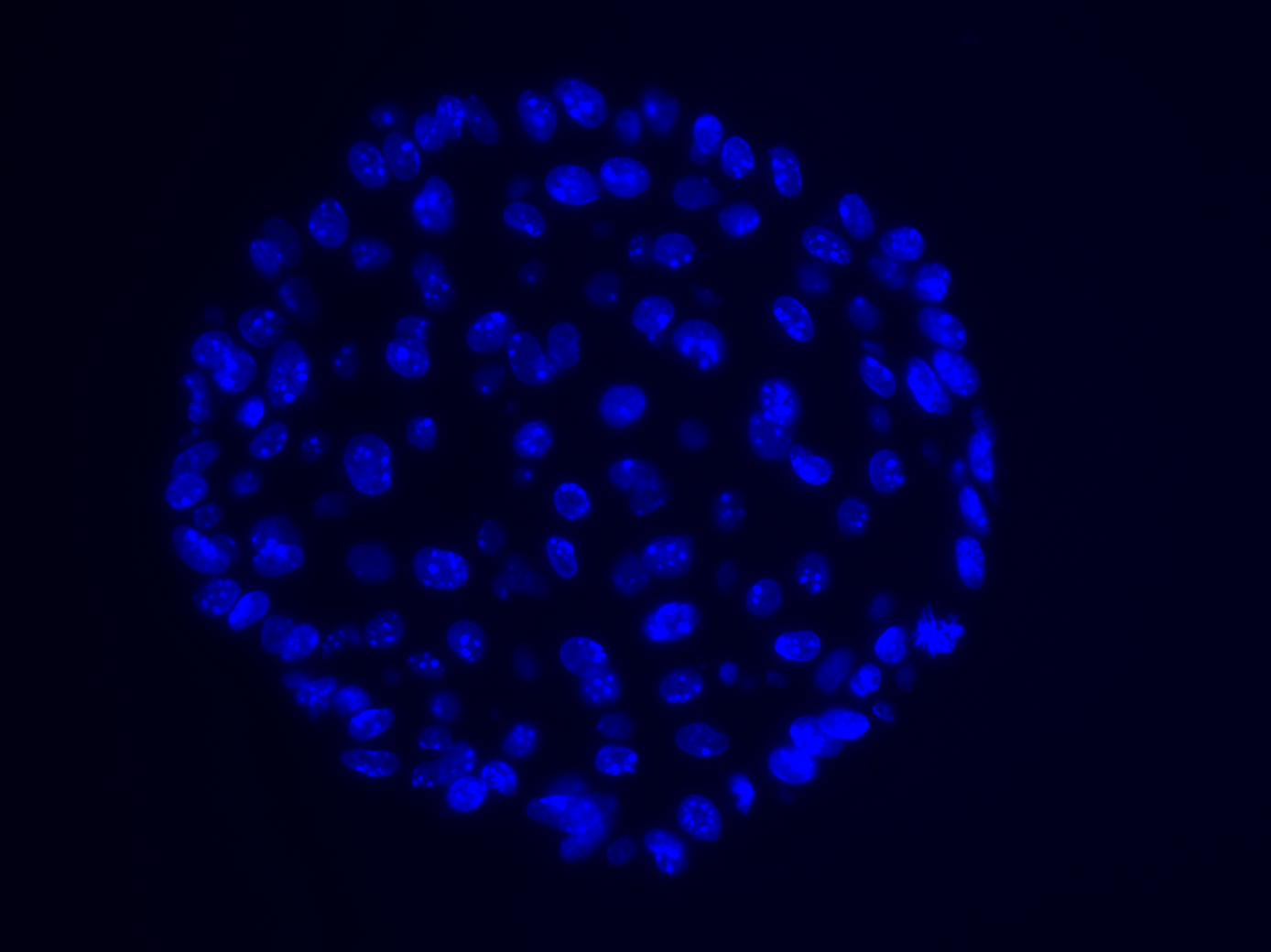

Supplement: Supplementary file 9 — Source data Fig. 3 [file 44319_2024_335_MOESM9_ESM.zip › Figure 3/3J/ENRAD- EV Dapi.tif]

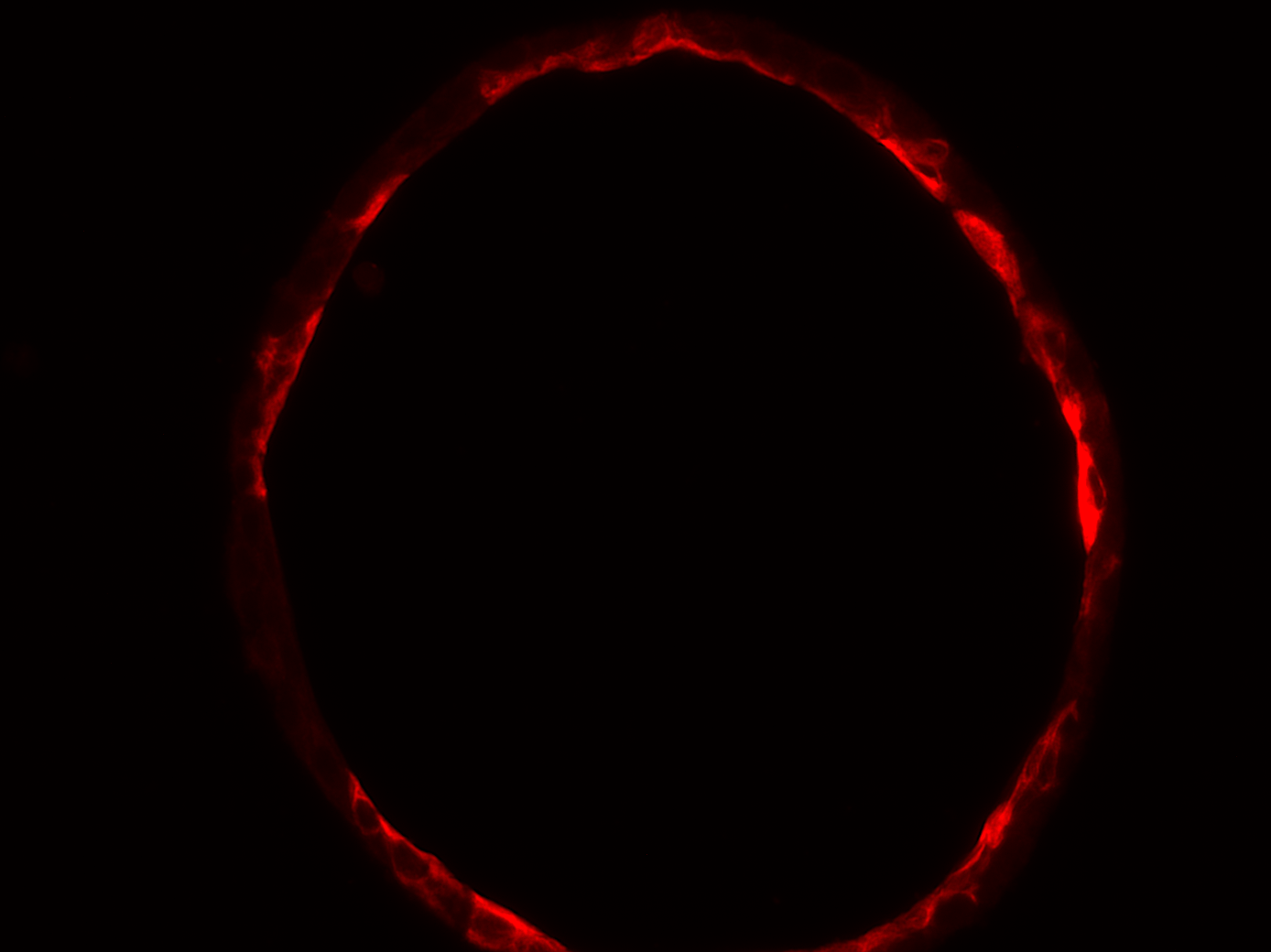

Supplement: Supplementary file 9 — Source data Fig. 3 [file 44319_2024_335_MOESM9_ESM.zip › Figure 3/3J/ENRAD- FOXA1 Ck8.tif]

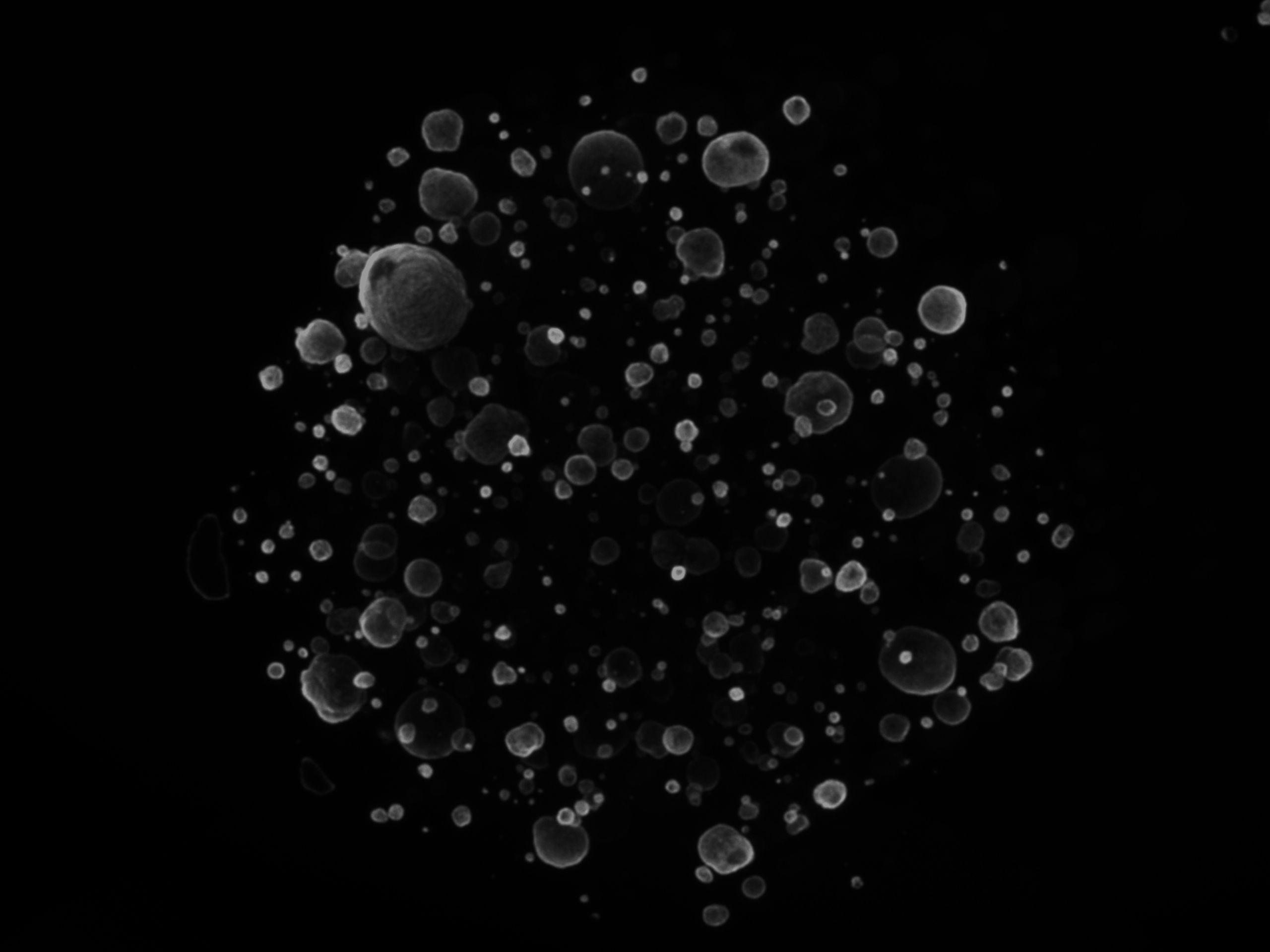

Supplement: Supplementary file 9 — Source data Fig. 3 [file 44319_2024_335_MOESM9_ESM.zip › Figure 3/3A/210212_RAR_inhibition_100nM_CalceinAM_d7_C57#1_mix_p23_CM+RARb_inh_100nM_d7_Calcein_5uM_1h_3.tif]

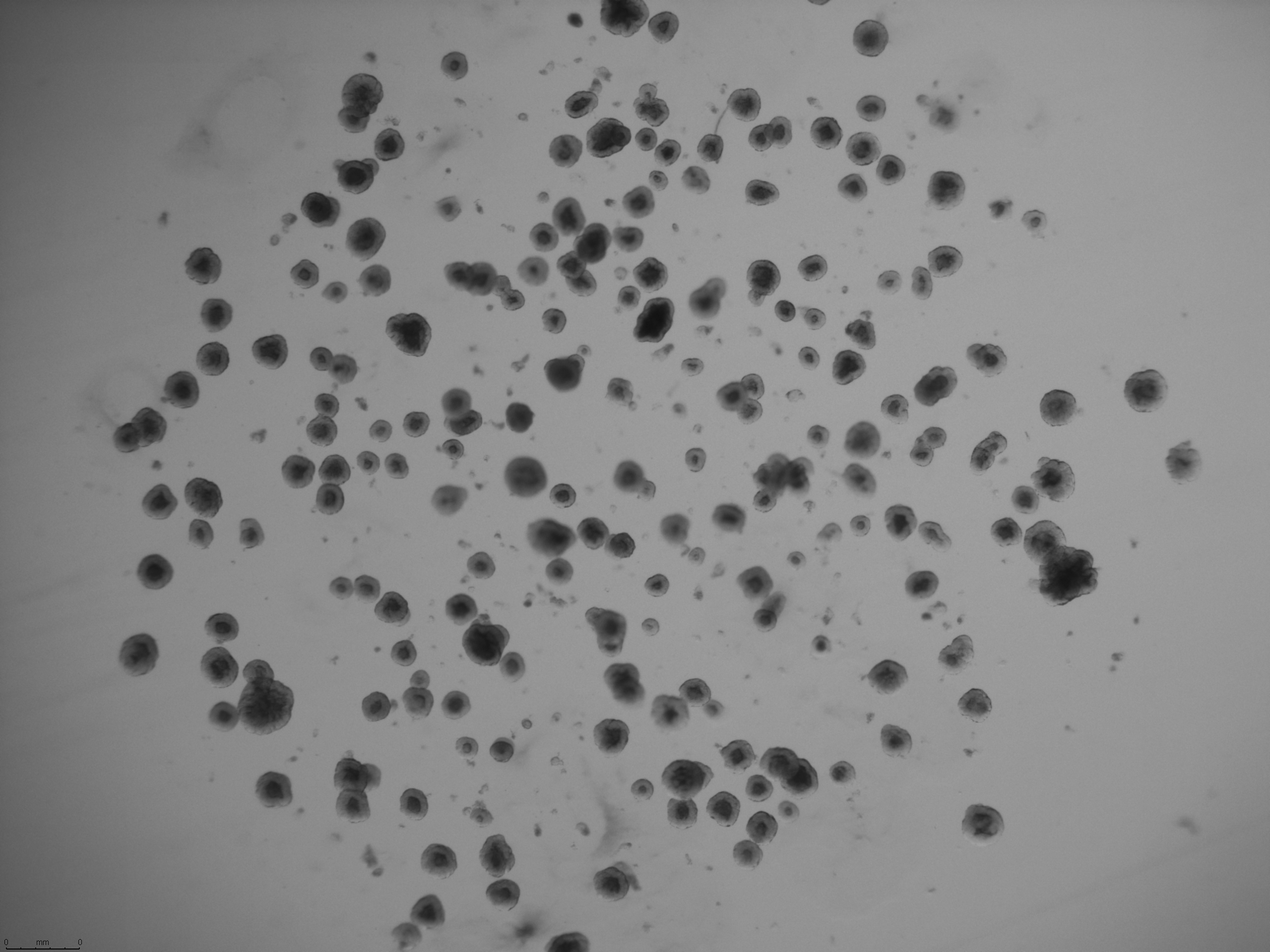

Supplement: Supplementary file 9 — Source data Fig. 3 [file 44319_2024_335_MOESM9_ESM.zip › Figure 3/3A/191209_Pan_RAR_inhibitor_C57#1_mix_p18_cm_panRAR_inh_1uM_d7.tif]

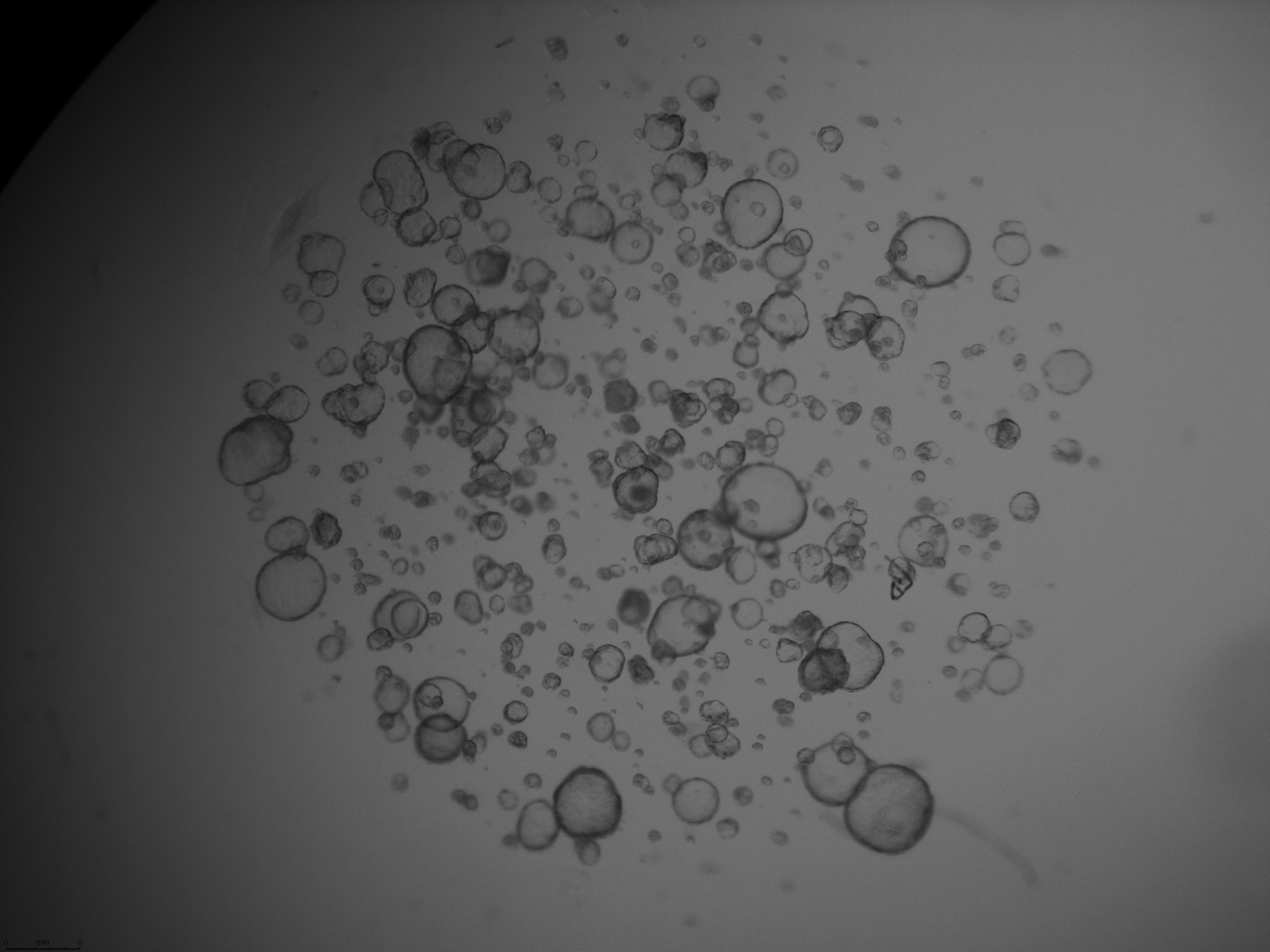

Supplement: Supplementary file 9 — Source data Fig. 3 [file 44319_2024_335_MOESM9_ESM.zip › Figure 3/3A/210212_RAR_inhibition_100nM_morphology_d7_C57#1_mix_p23_CM+RARa_inh_100nM_d7_1.tif]

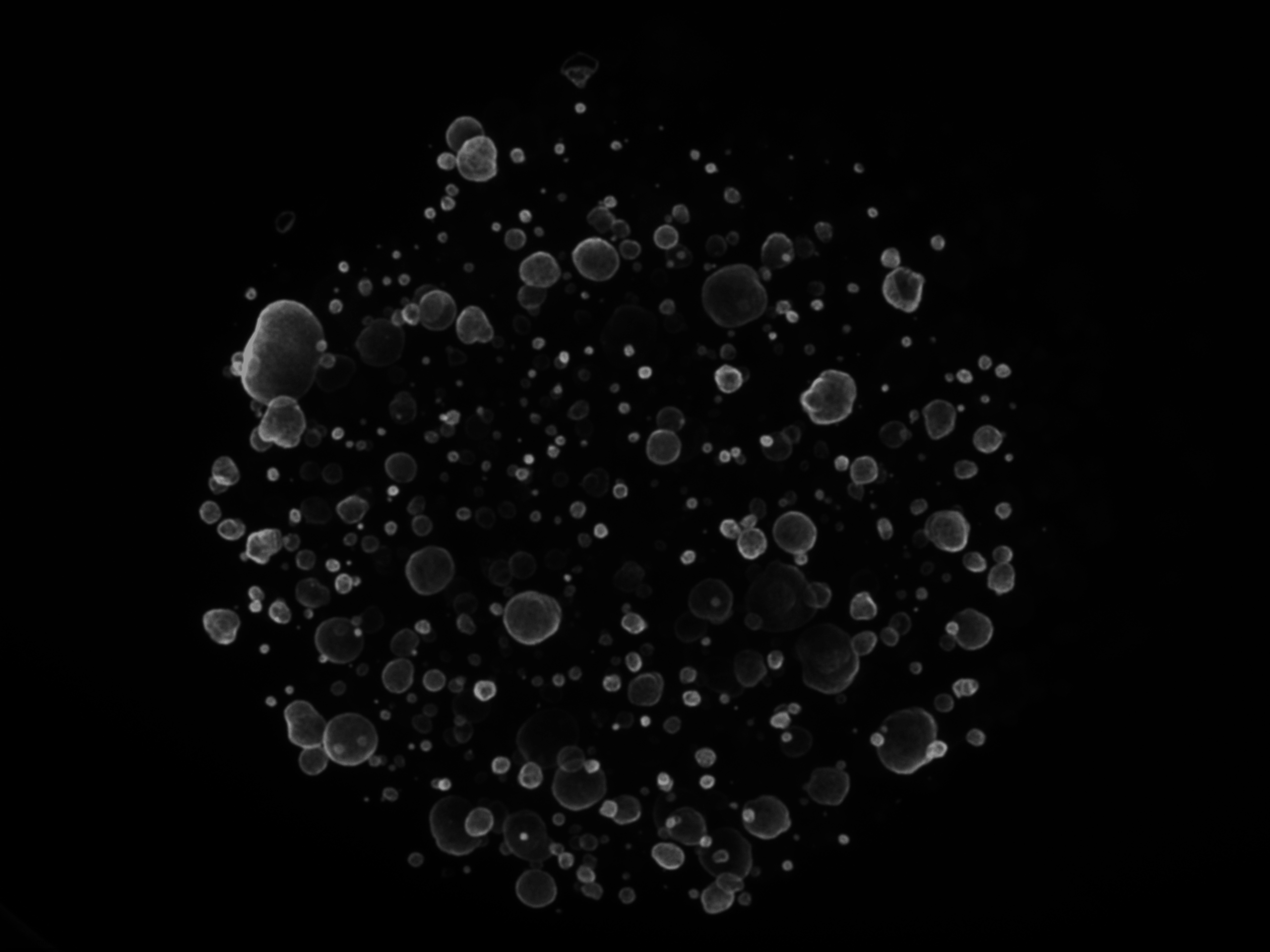

Supplement: Supplementary file 9 — Source data Fig. 3 [file 44319_2024_335_MOESM9_ESM.zip › Figure 3/3A/210212_RAR_inhibition_100nM_CalceinAM_d7_C57#1_mix_p23_CM_d7_Calcein_5uM_DMSO_1h_3.tif]

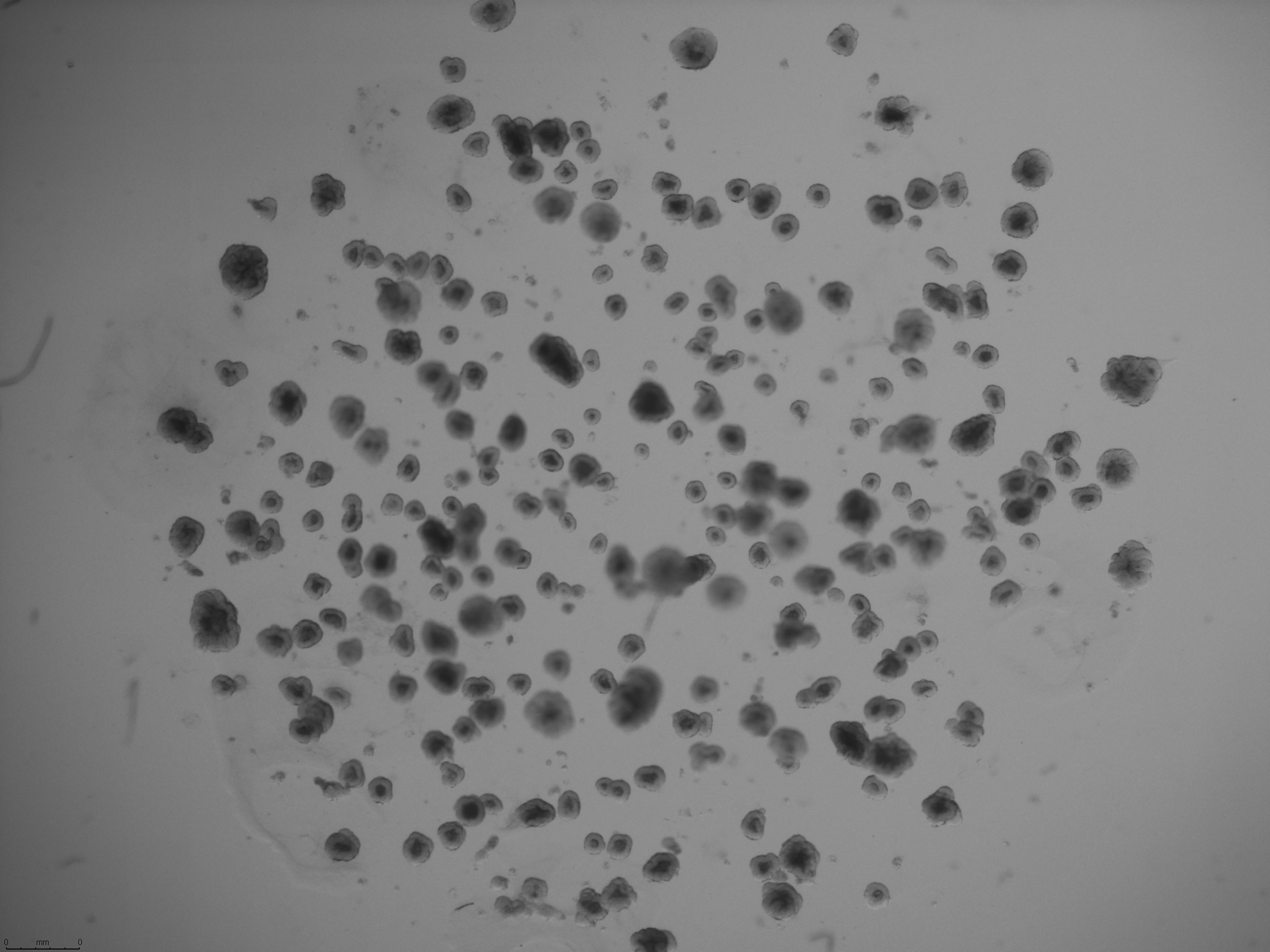

Supplement: Supplementary file 9 — Source data Fig. 3 [file 44319_2024_335_MOESM9_ESM.zip › Figure 3/3A/191209_RARg_inhibitor_C57#1_mix_p18_cm_RARg_inh_1uM_d7.tif]

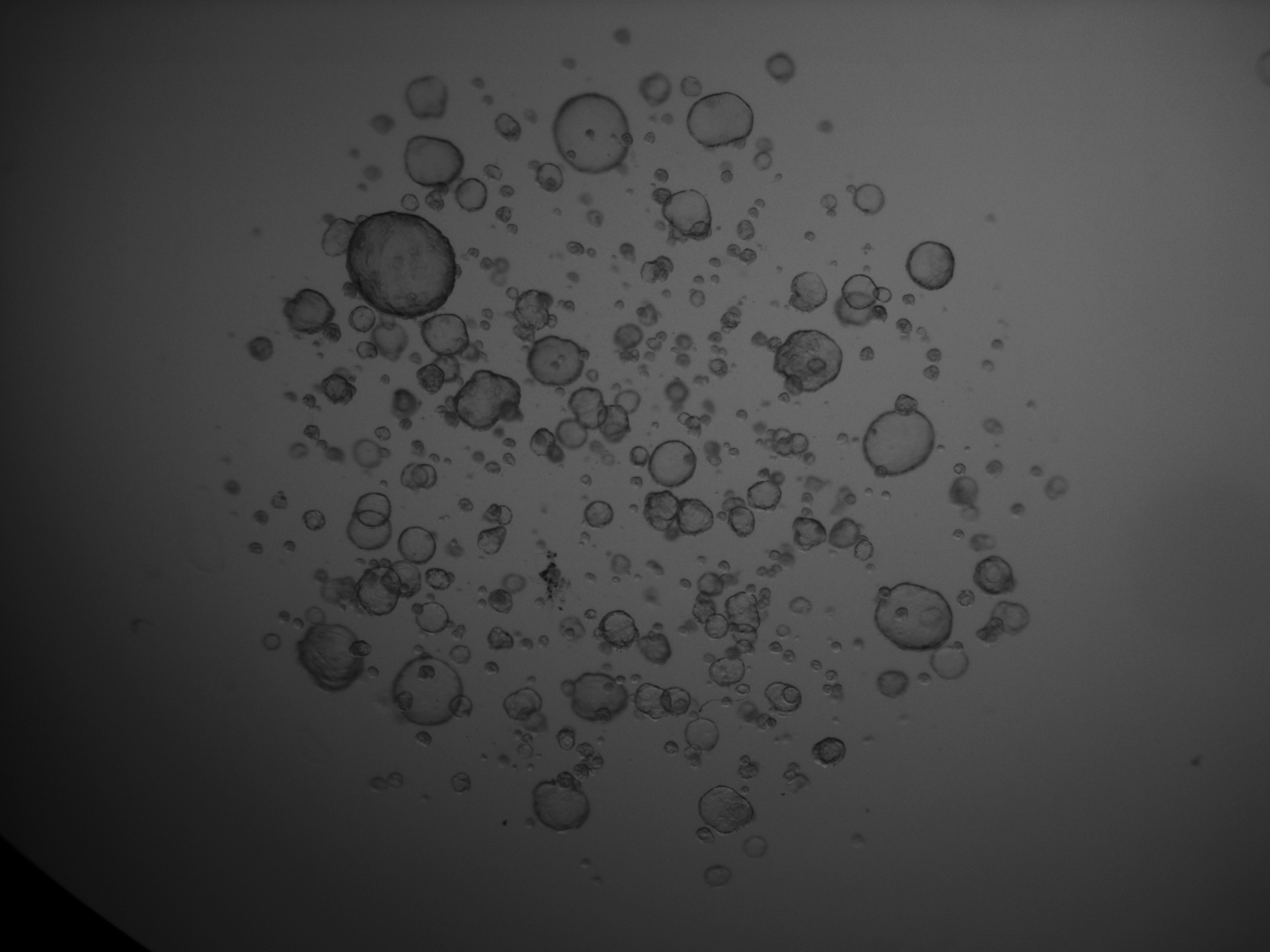

Supplement: Supplementary file 9 — Source data Fig. 3 [file 44319_2024_335_MOESM9_ESM.zip › Figure 3/3A/210212_RAR_inhibition_100nM_morphology_d7_C57#1_mix_p23_CM+RARb_inh_100nM_d7_3.tif]

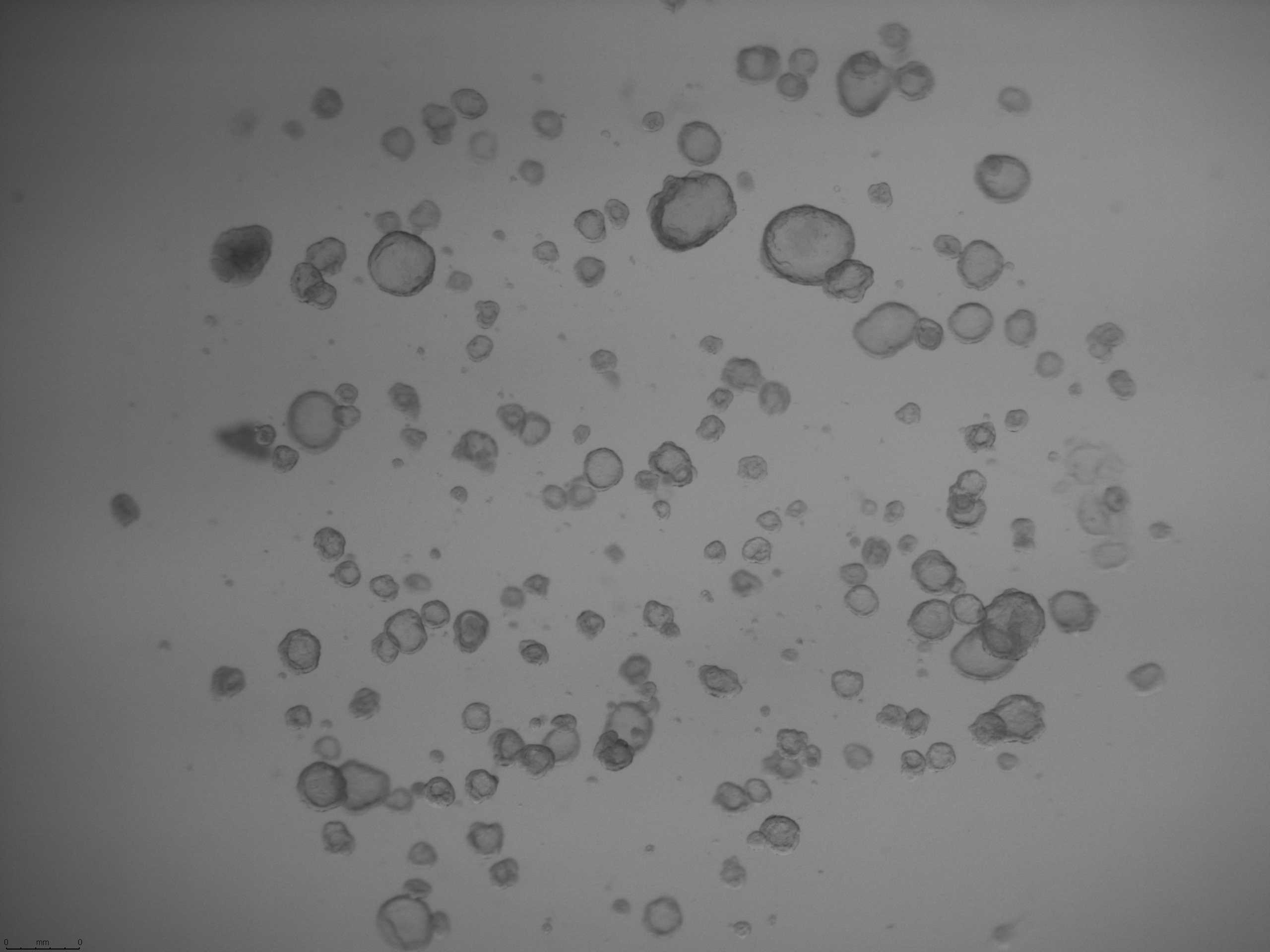

Supplement: Supplementary file 9 — Source data Fig. 3 [file 44319_2024_335_MOESM9_ESM.zip › Figure 3/3A/191209_RARb_inhibitor_C57#1_mix_p18_cm_RARb_inh_1uM_d7.tif]

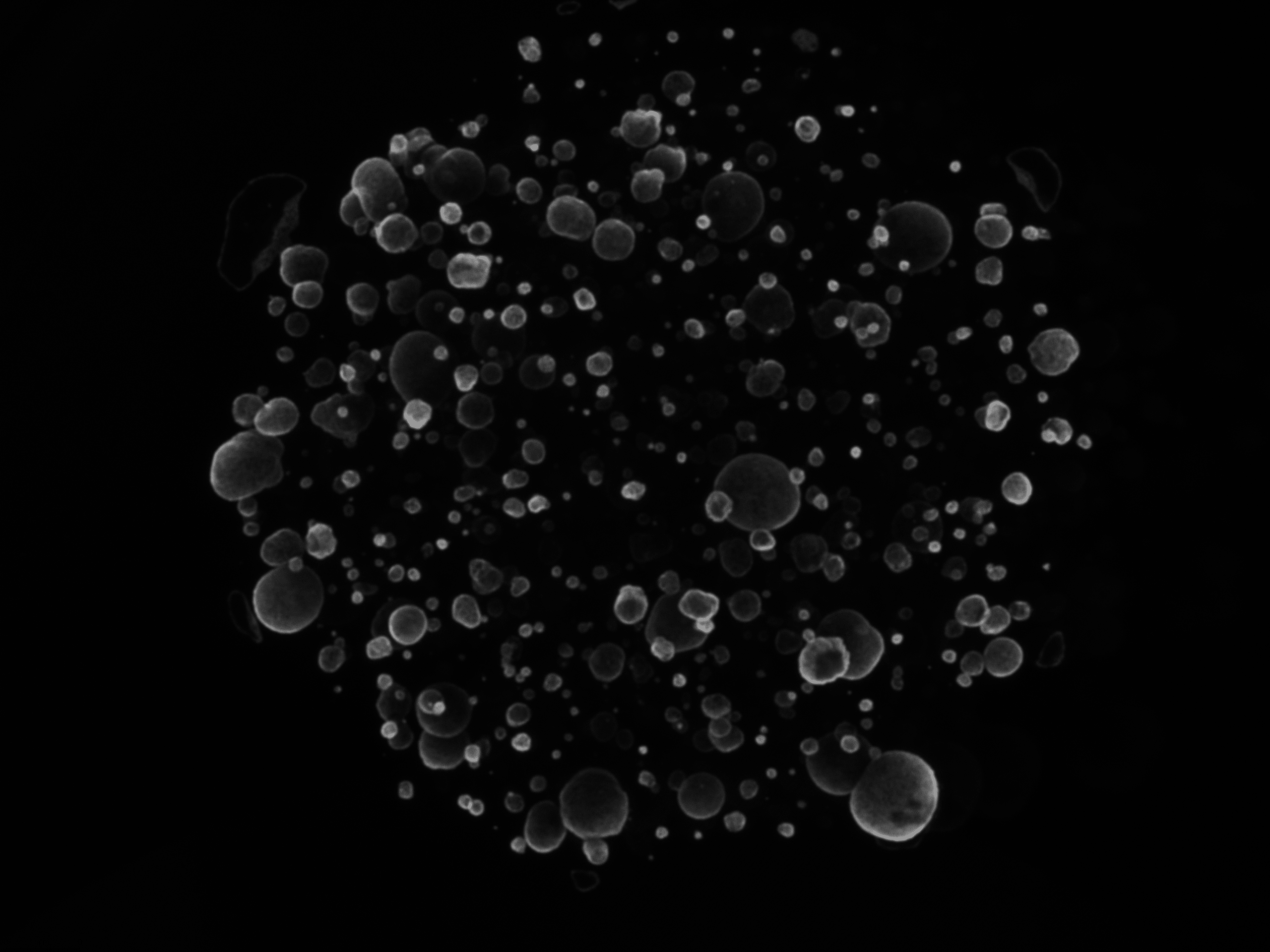

Supplement: Supplementary file 9 — Source data Fig. 3 [file 44319_2024_335_MOESM9_ESM.zip › Figure 3/3A/210212_RAR_inhibition_100nM_CalceinAM_d7_C57#1_mix_p23_CM+RARa_inh_100nM_d7_Calcein_5uM_1h_1.tif]

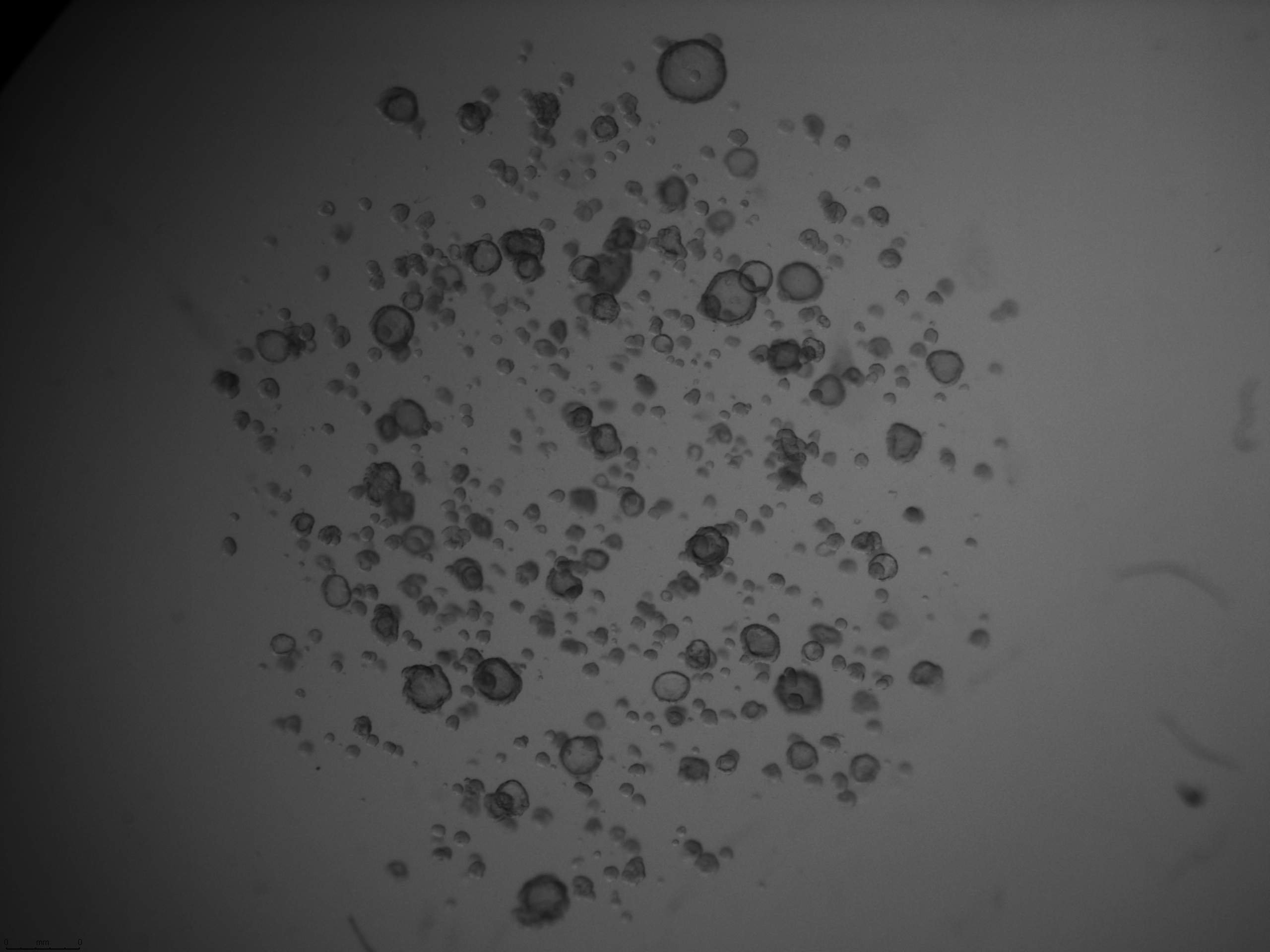

Supplement: Supplementary file 9 — Source data Fig. 3 [file 44319_2024_335_MOESM9_ESM.zip › Figure 3/3A/210212_RAR_inhibition_100nM_morphology_d7_C57#1_mix_p23_CM+RARg_inh_100nM_d7_1.tif]

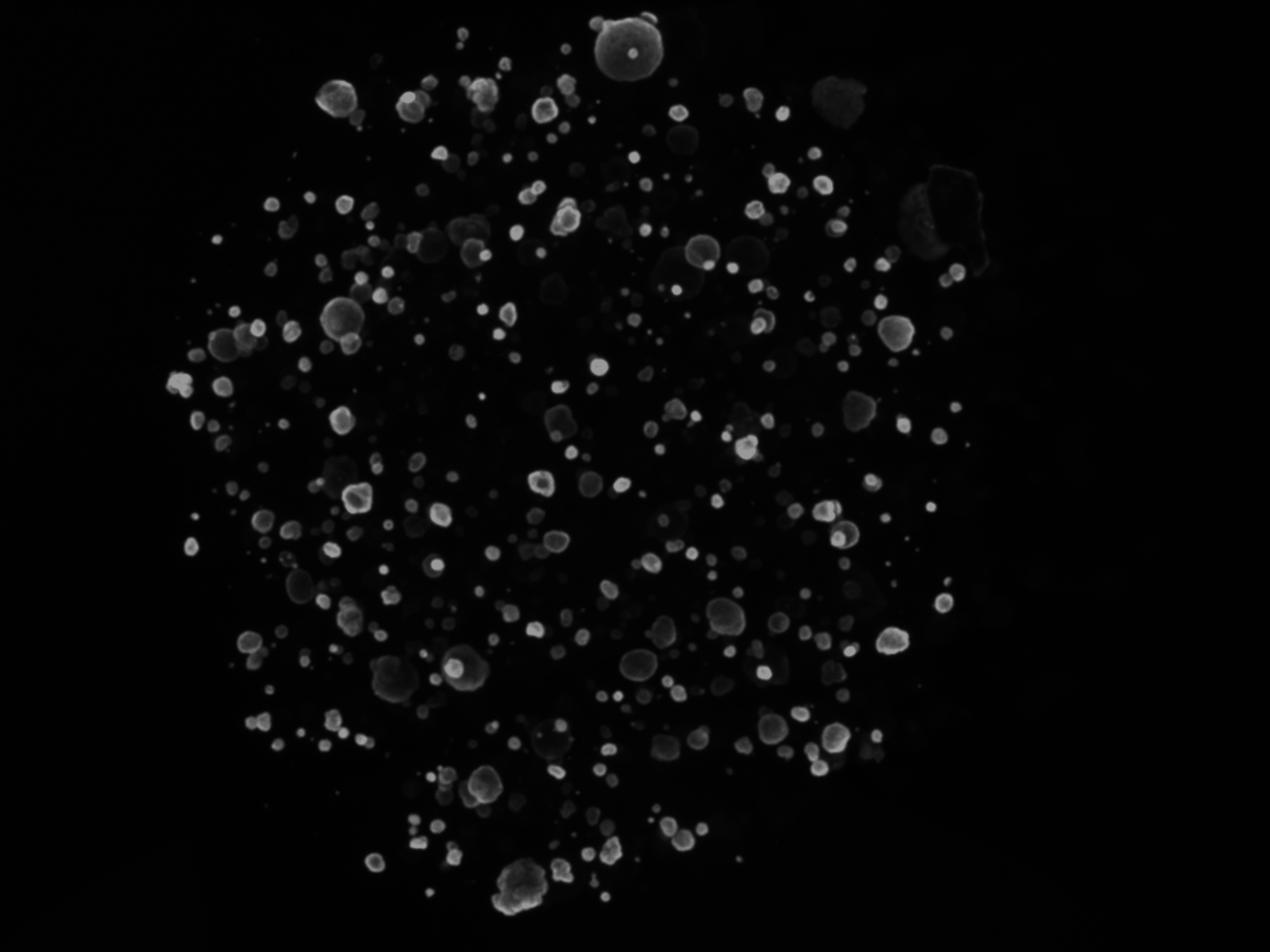

Supplement: Supplementary file 9 — Source data Fig. 3 [file 44319_2024_335_MOESM9_ESM.zip › Figure 3/3A/210212_RAR_inhibition_100nM_CalceinAM_d7_C57#1_mix_p23_CM+RARg_inh_100nM_d7_Calcein_5uM_1h_1.tif]

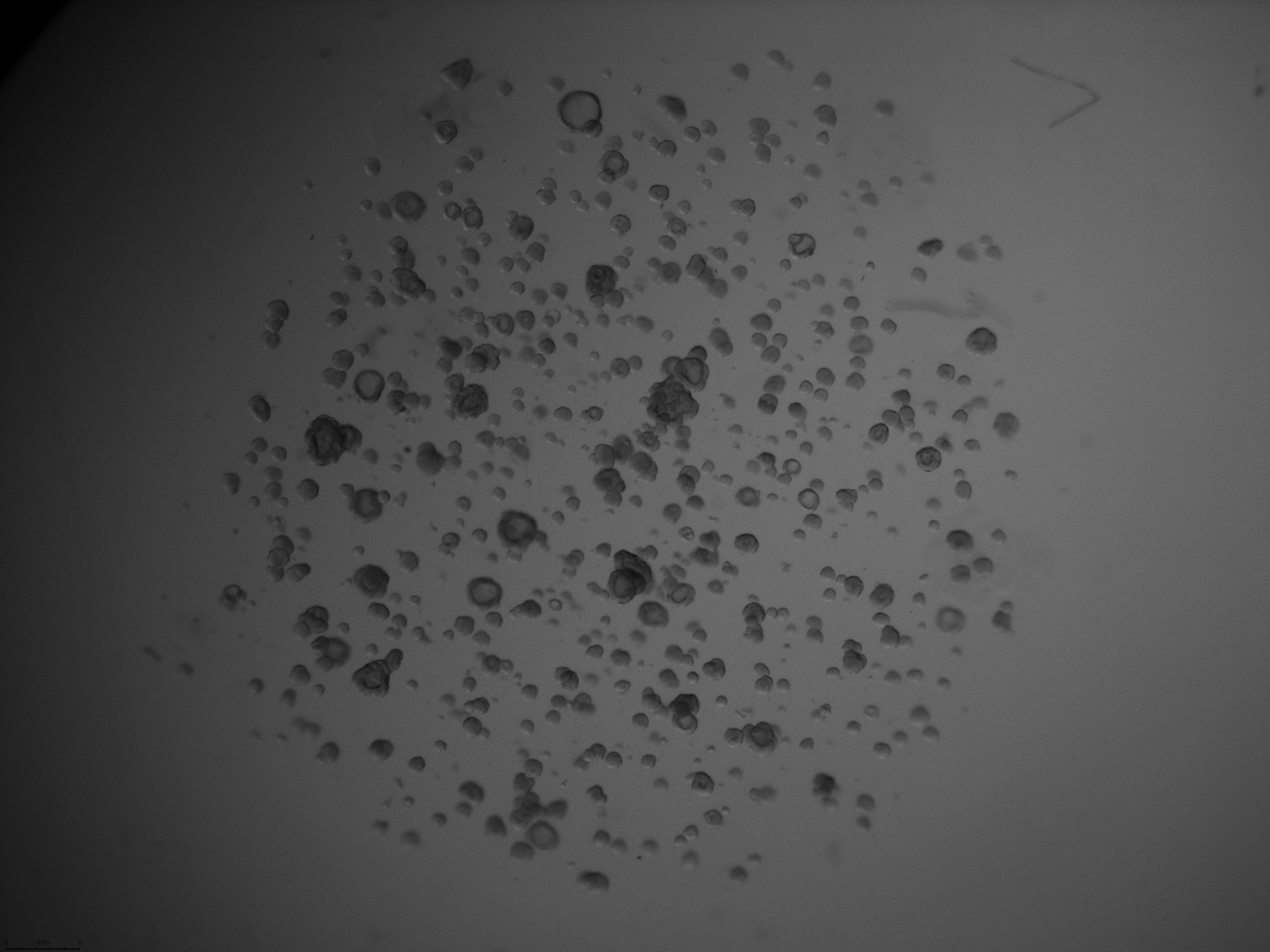

Supplement: Supplementary file 9 — Source data Fig. 3 [file 44319_2024_335_MOESM9_ESM.zip › Figure 3/3A/210212_RAR_inhibition_100nM_morphology_d7_C57#1_mix_p23_CM+panRAR_inh_100nM_d7_1.tif]

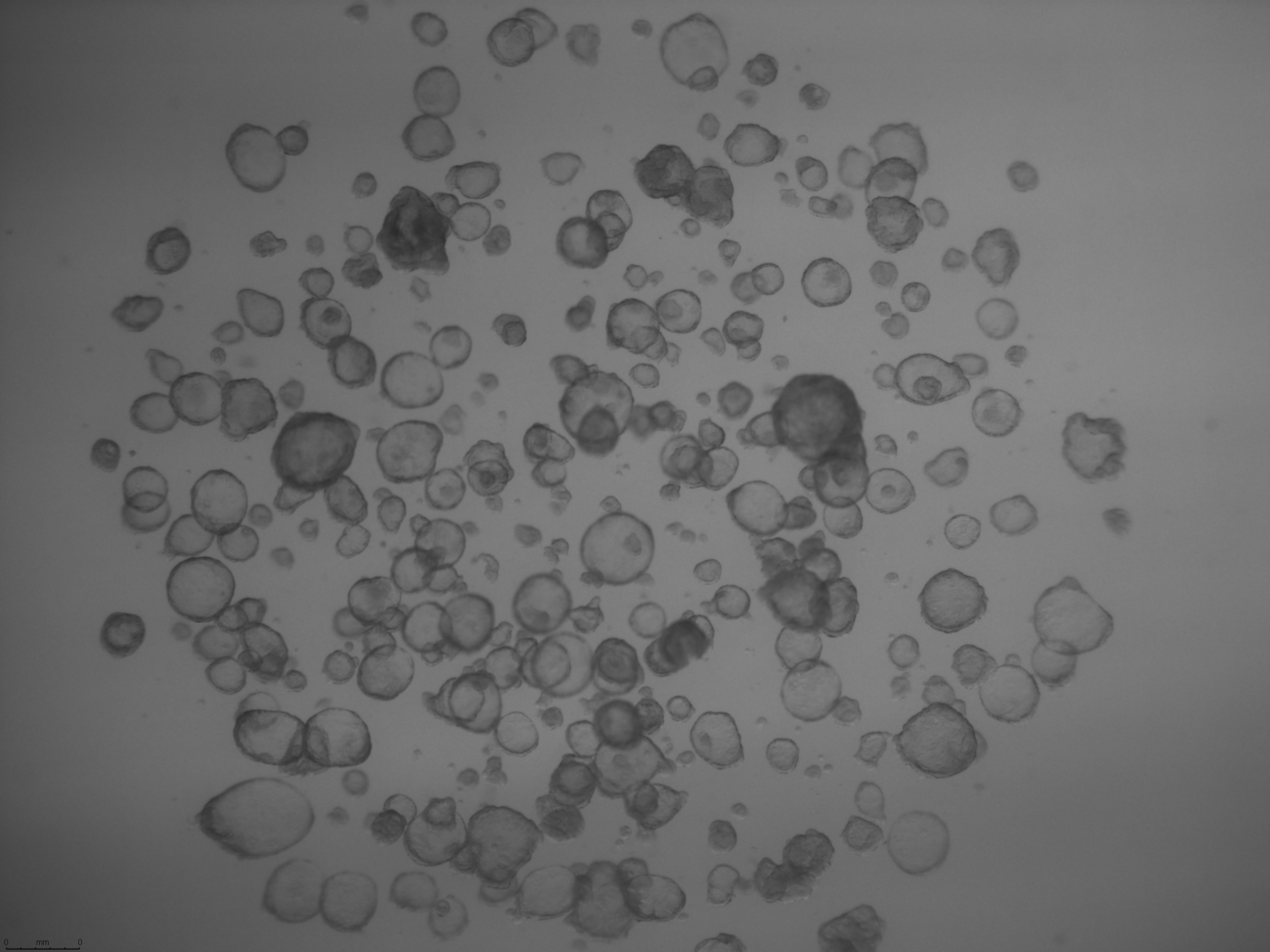

Supplement: Supplementary file 9 — Source data Fig. 3 [file 44319_2024_335_MOESM9_ESM.zip › Figure 3/3A/191209_RARa_inhibitor_C57#1_mix_p18_cm_RARa_inh_1uM_d7.tif]

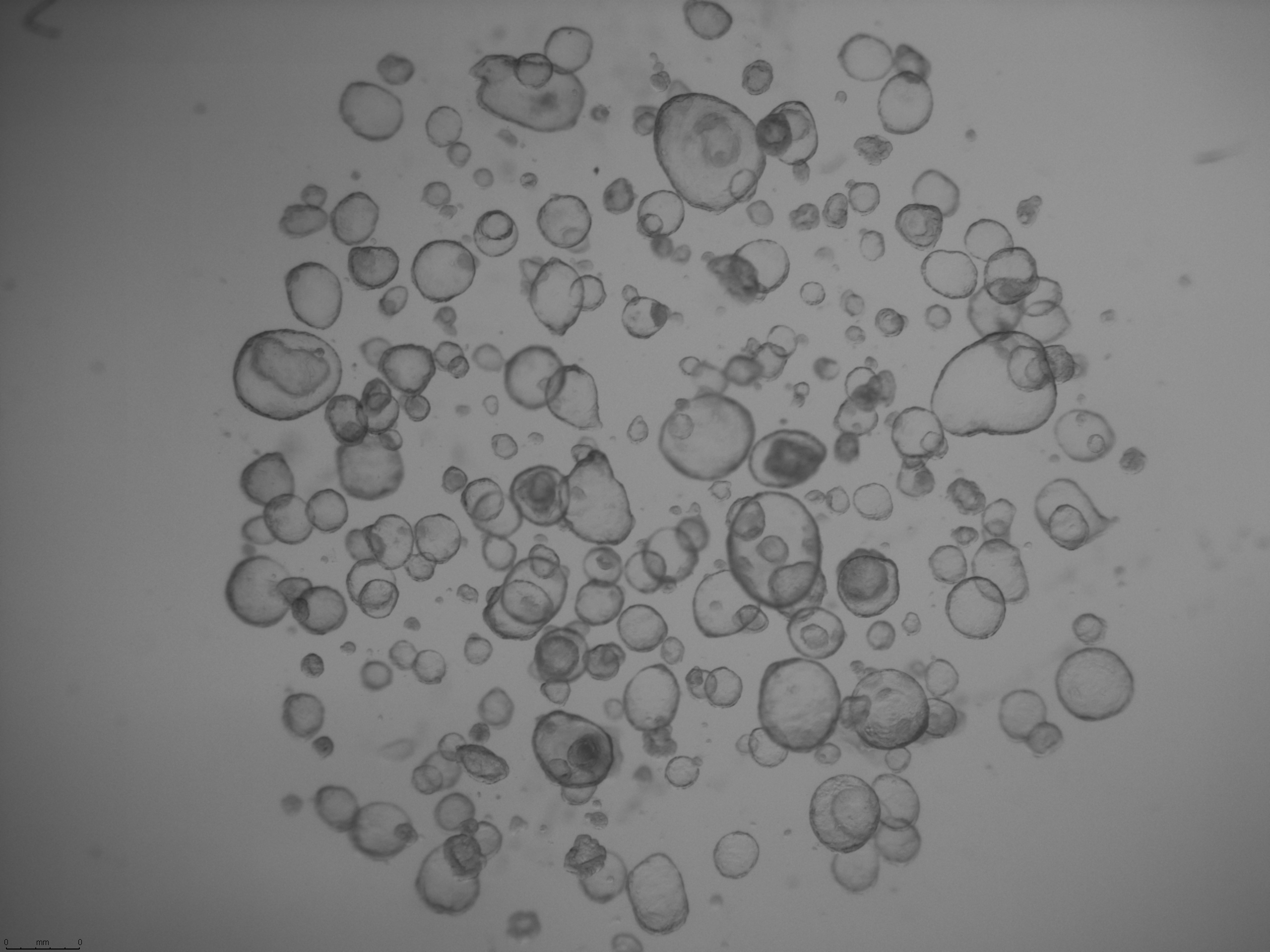

Supplement: Supplementary file 9 — Source data Fig. 3 [file 44319_2024_335_MOESM9_ESM.zip › Figure 3/3A/191209_DMSO_C57#1_mix_p18_cm_d7.tif]

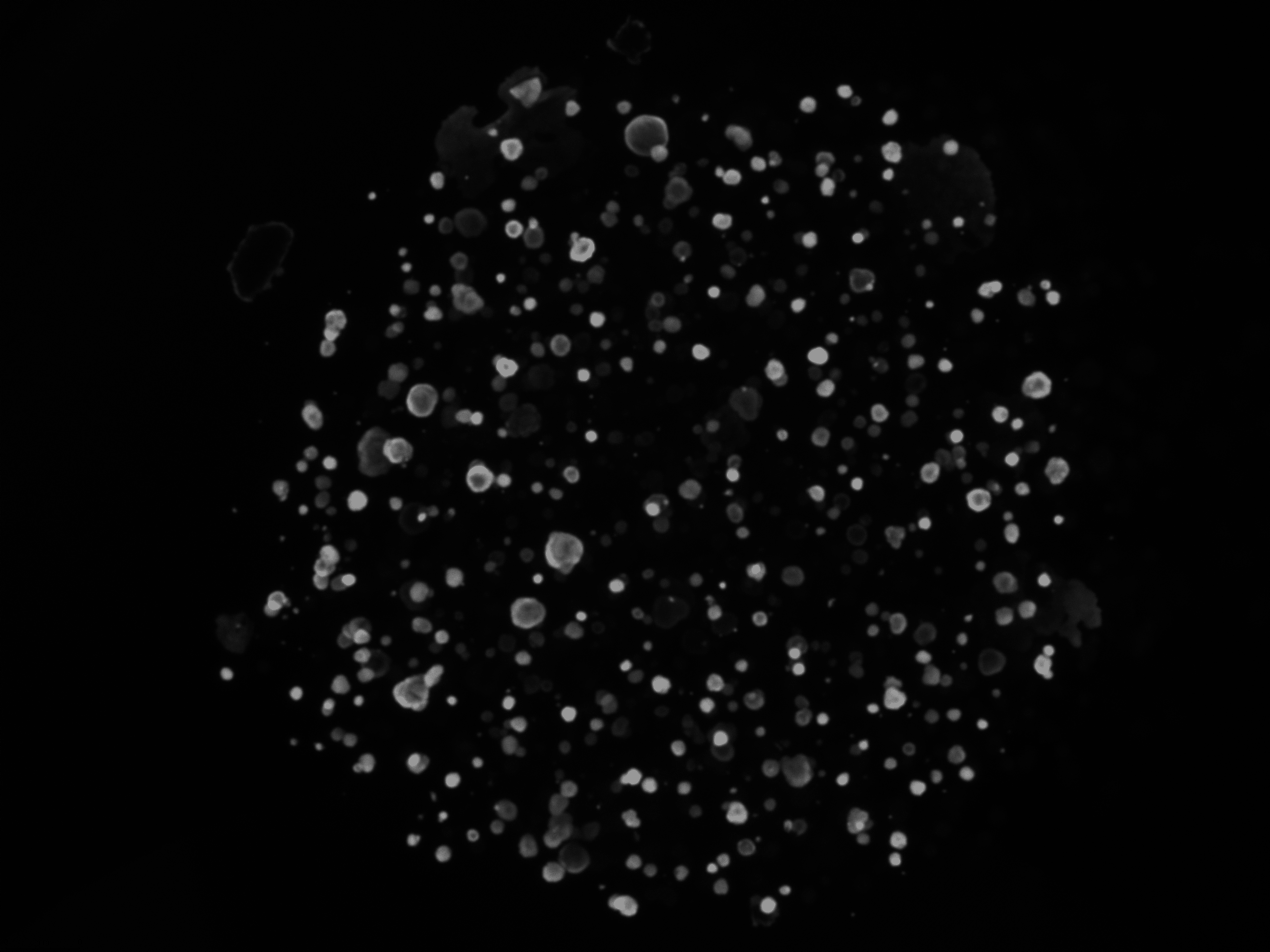

Supplement: Supplementary file 9 — Source data Fig. 3 [file 44319_2024_335_MOESM9_ESM.zip › Figure 3/3A/210212_RAR_inhibition_100nM_CalceinAM_d7_C57#1_mix_p23_CM+panRAR_inh_100nM_d7_Calcein_5uM_1h_1.tif]

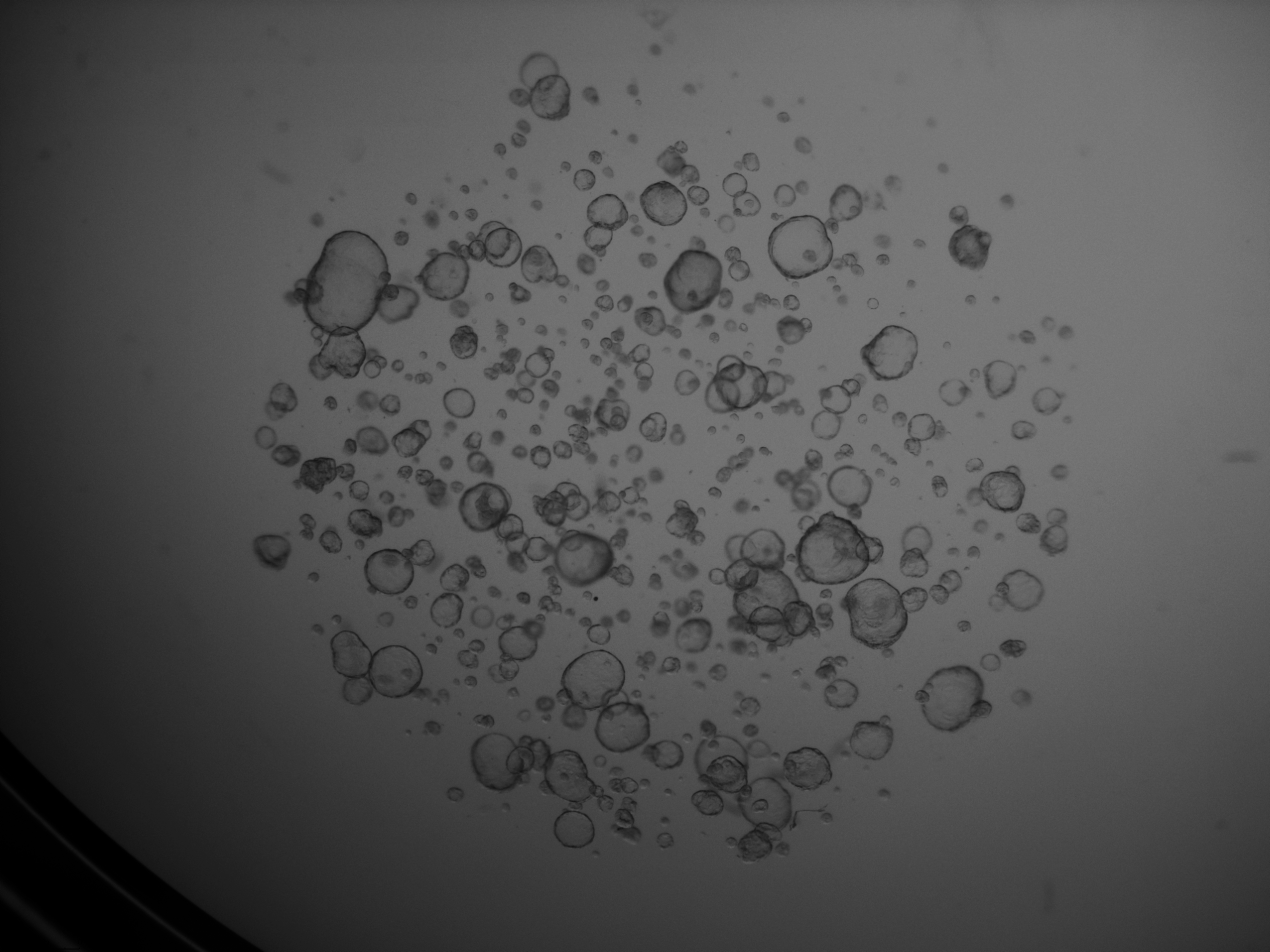

Supplement: Supplementary file 9 — Source data Fig. 3 [file 44319_2024_335_MOESM9_ESM.zip › Figure 3/3A/210212_RAR_inhibition_100nM_morphology_d7_C57#1_mix_p23_CM_DMSO_d7_3.tif]

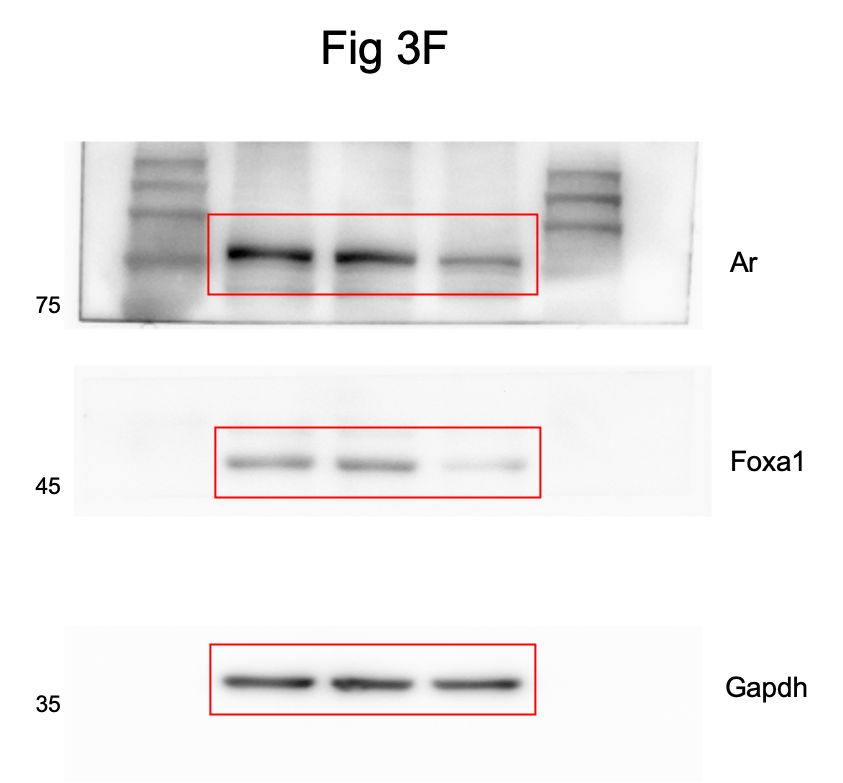

Supplement: Supplementary file 9 — Source data Fig. 3 [file 44319_2024_335_MOESM9_ESM.zip › Figure 3/3F/3F.png]

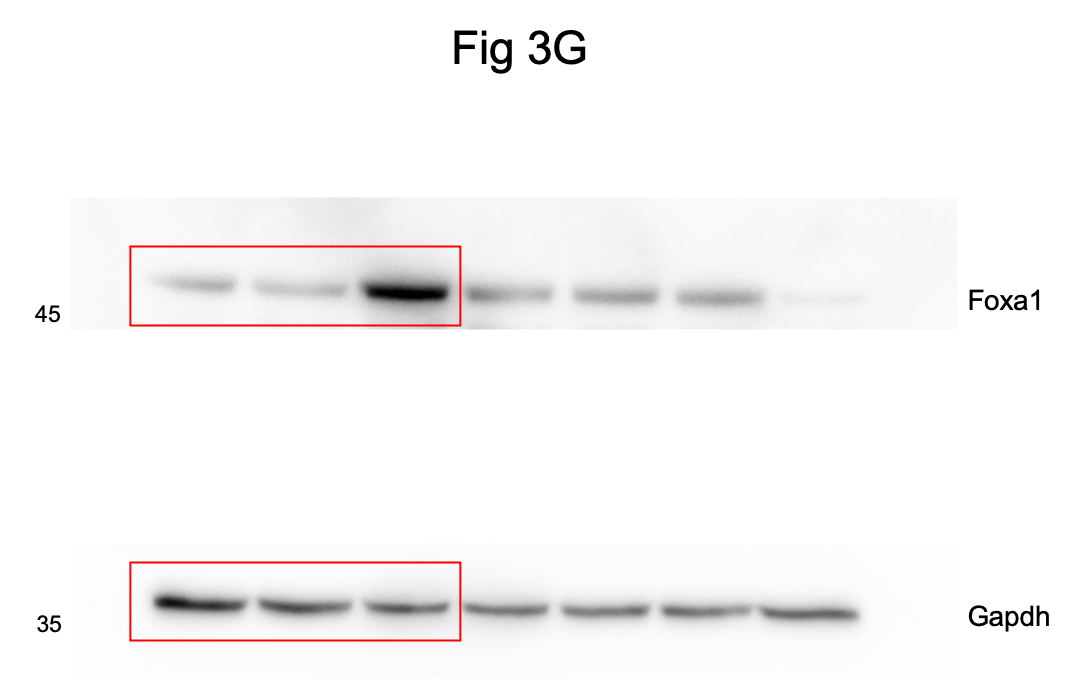

Supplement: Supplementary file 9 — Source data Fig. 3 [file 44319_2024_335_MOESM9_ESM.zip › Figure 3/3G/3G.png]

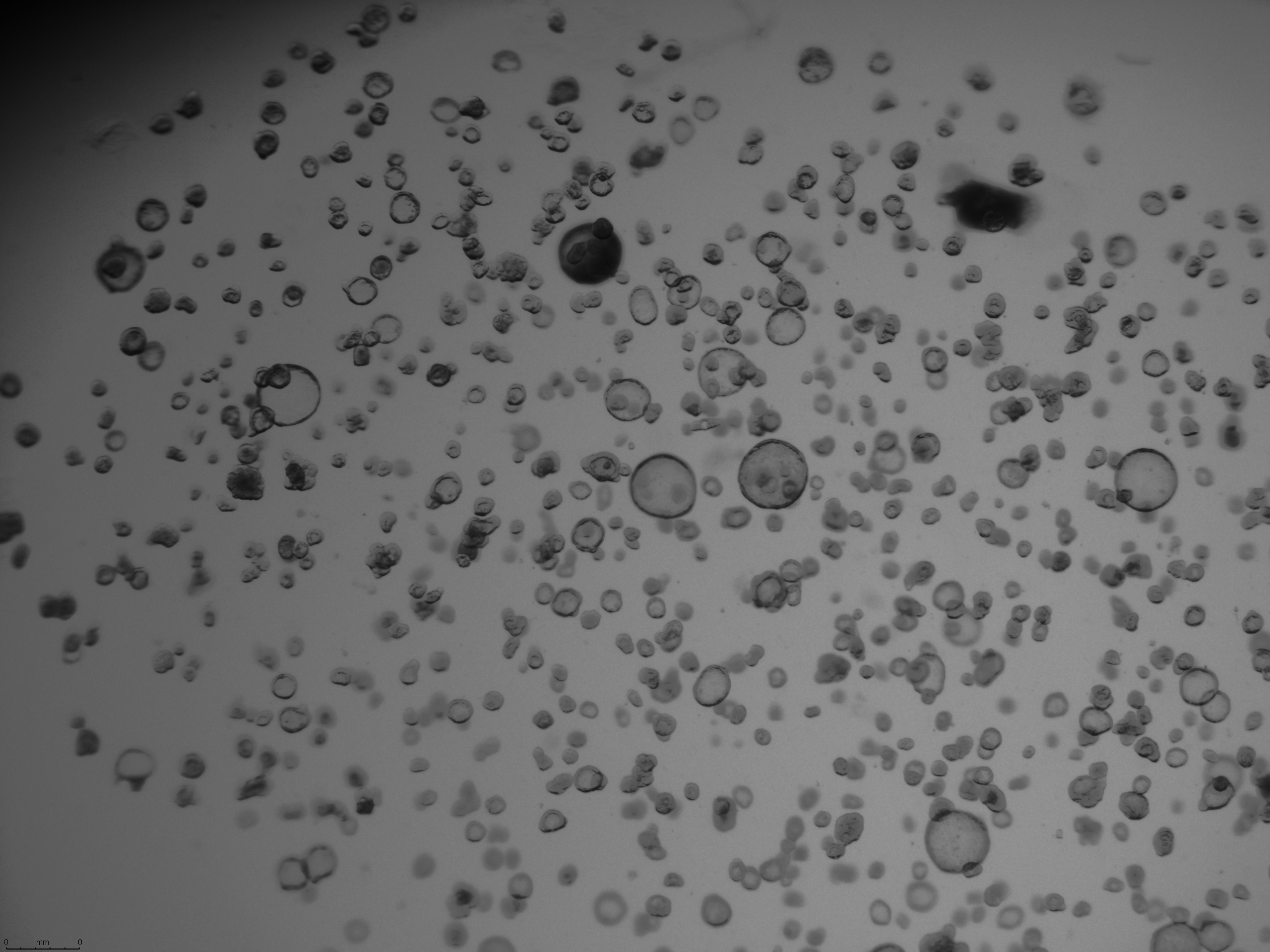

Supplement: Supplementary file 9 — Source data Fig. 3 [file 44319_2024_335_MOESM9_ESM.zip › Figure 3/3I/Image009.tif]

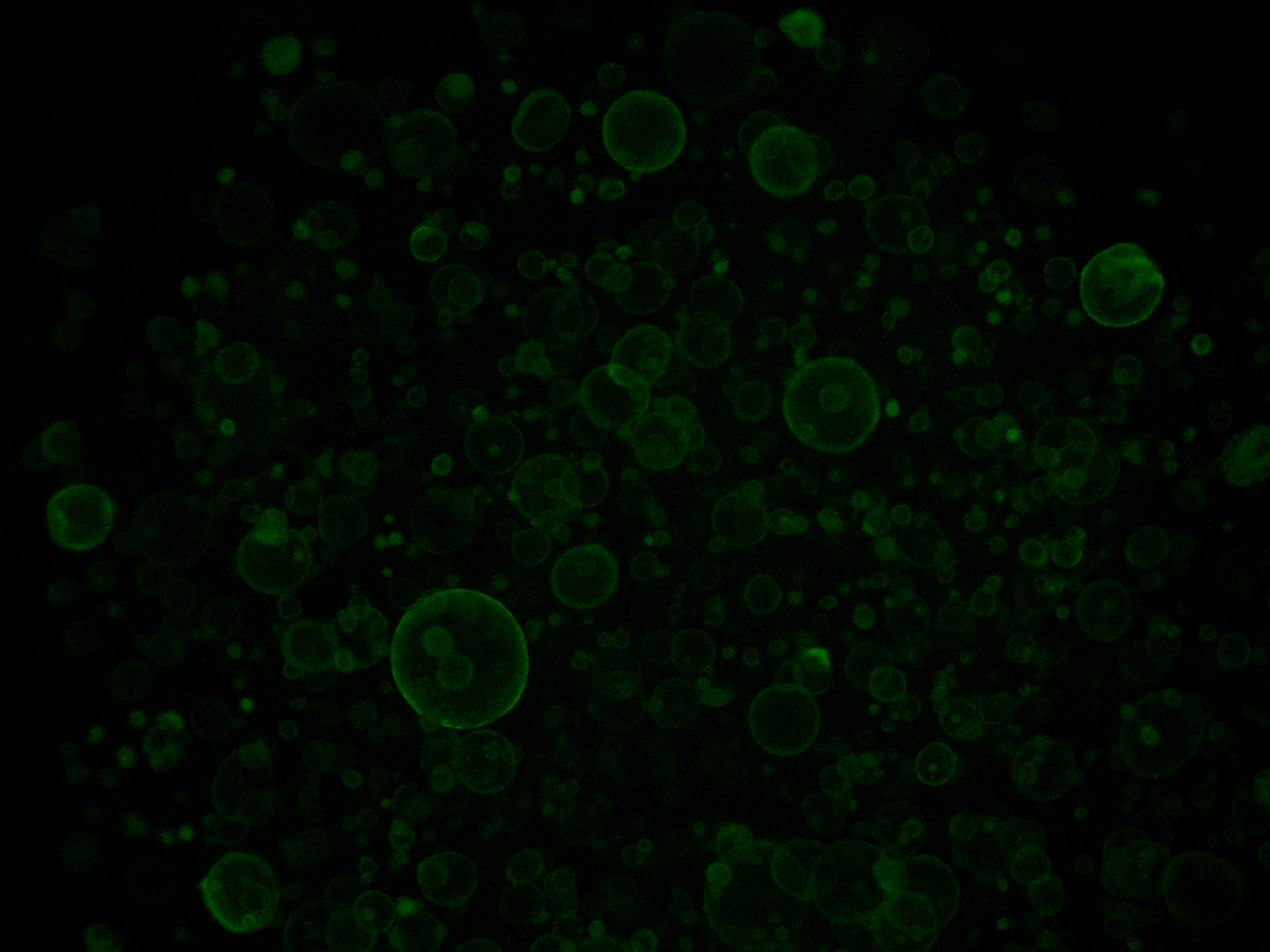

Supplement: Supplementary file 9 — Source data Fig. 3 [file 44319_2024_335_MOESM9_ESM.zip › Figure 3/3I/Image005.tif]

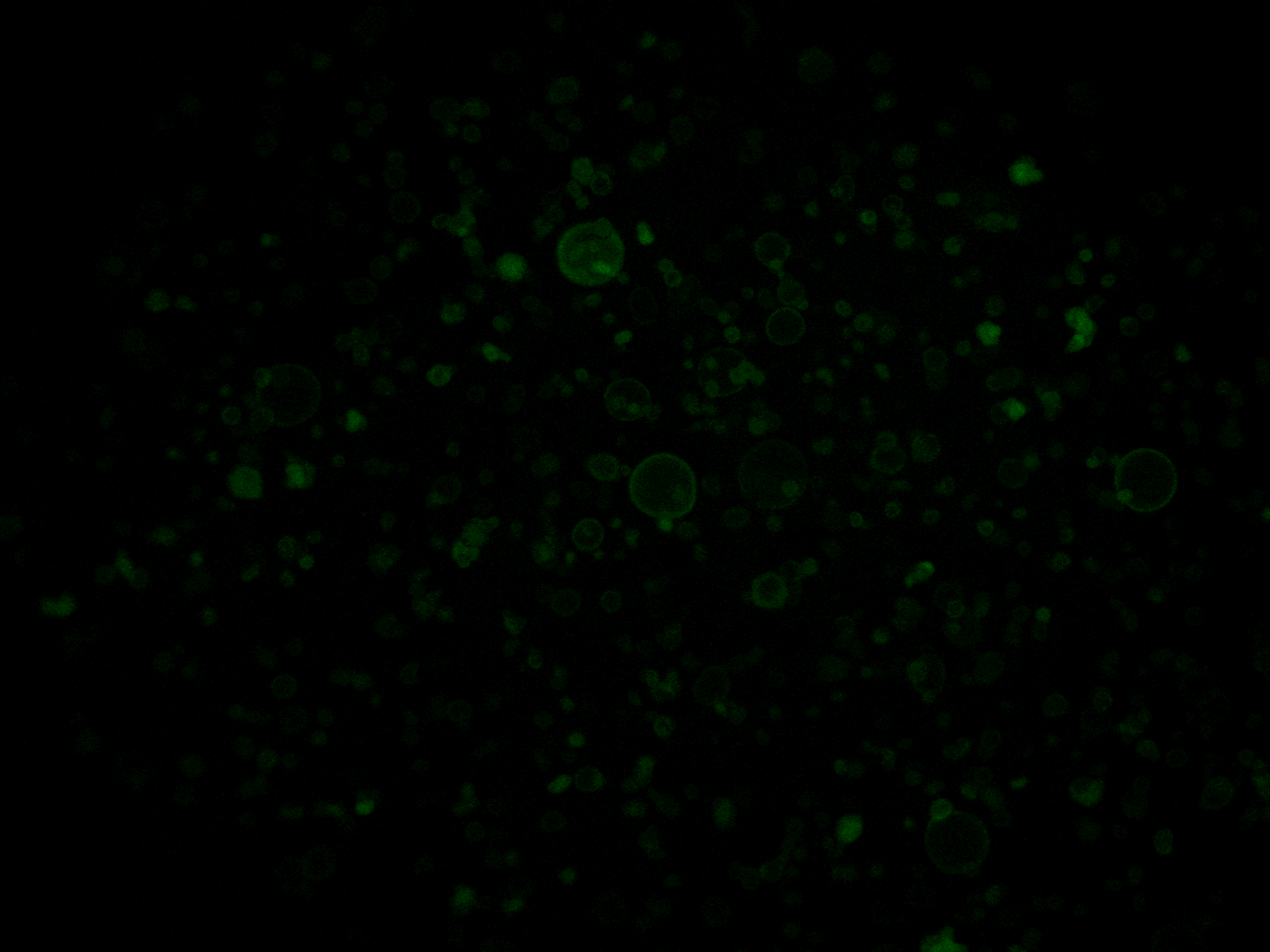

Supplement: Supplementary file 9 — Source data Fig. 3 [file 44319_2024_335_MOESM9_ESM.zip › Figure 3/3I/Image010.tif]

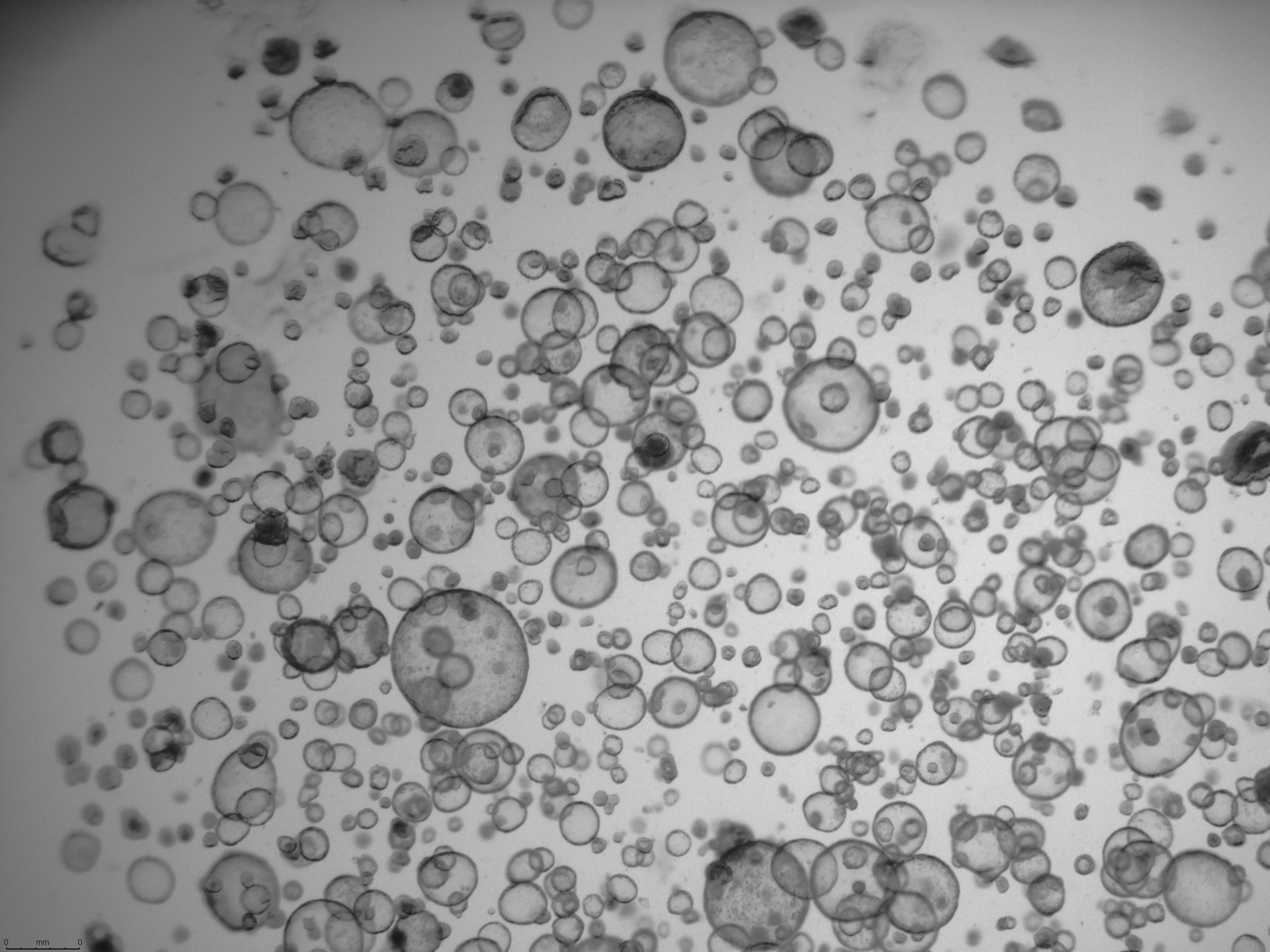

Supplement: Supplementary file 9 — Source data Fig. 3 [file 44319_2024_335_MOESM9_ESM.zip › Figure 3/3I/Image004.tif]

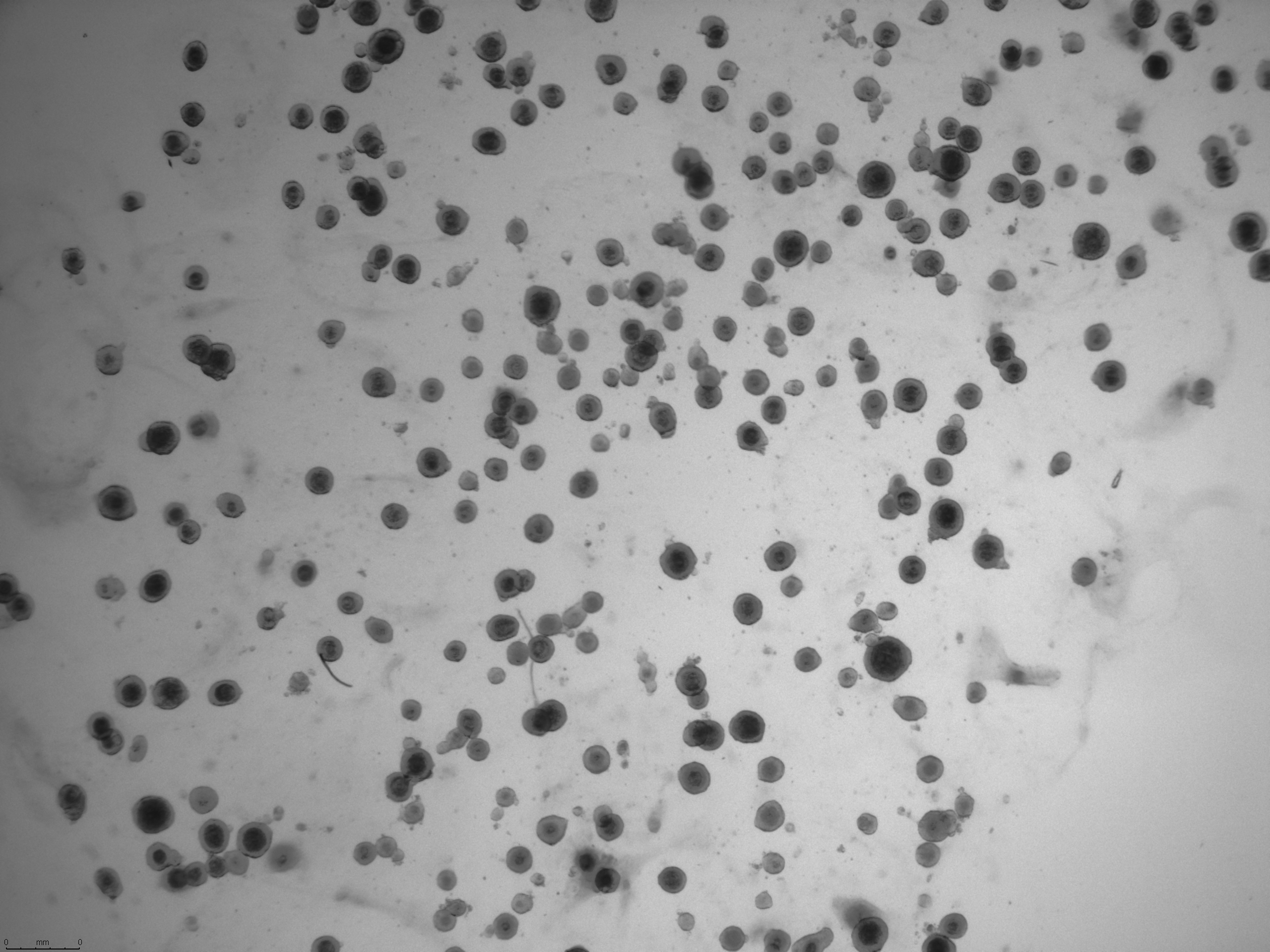

Supplement: Supplementary file 9 — Source data Fig. 3 [file 44319_2024_335_MOESM9_ESM.zip › Figure 3/3I/Image006.tif]

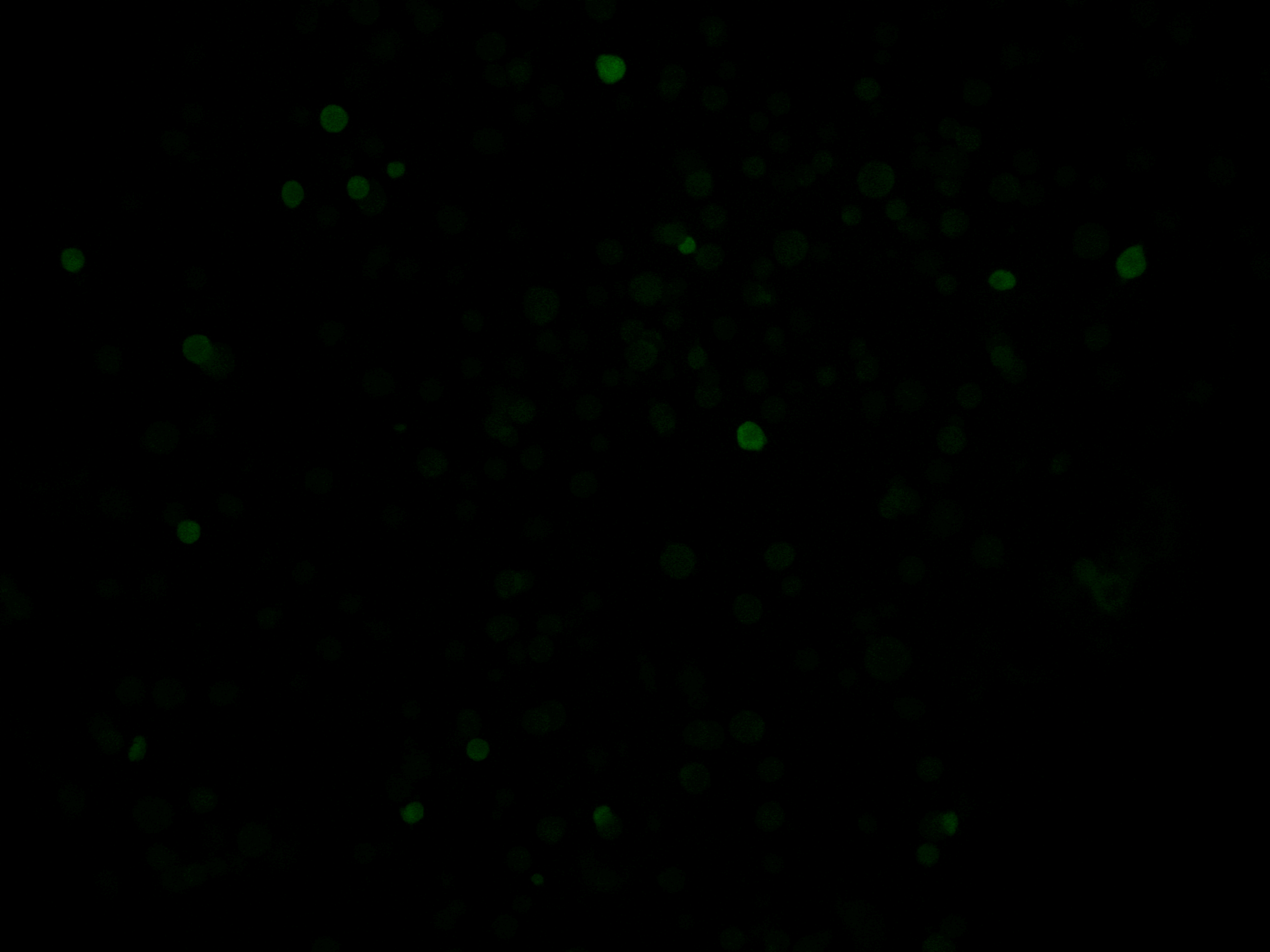

Supplement: Supplementary file 9 — Source data Fig. 3 [file 44319_2024_335_MOESM9_ESM.zip › Figure 3/3I/Image007.tif]

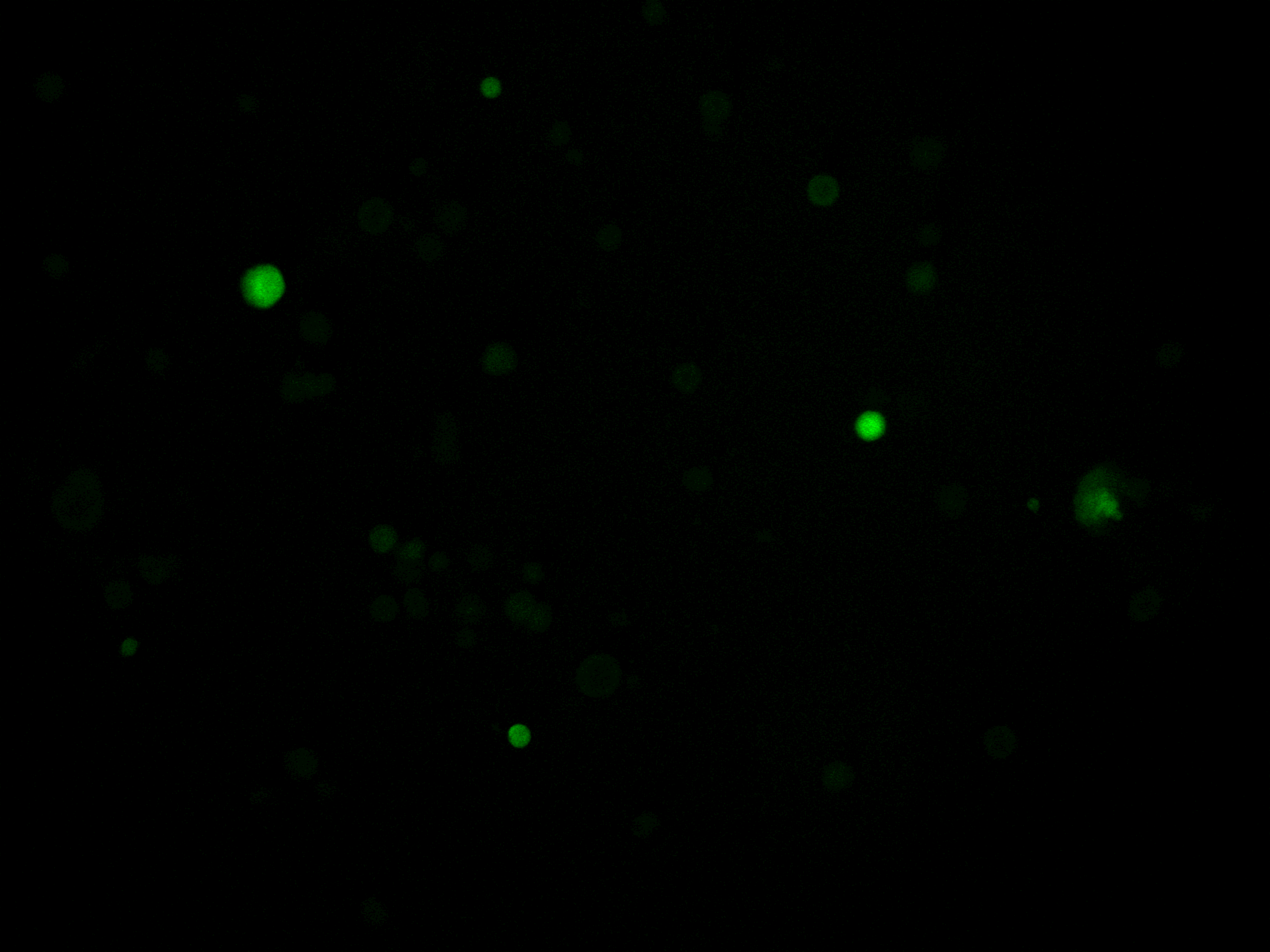

Supplement: Supplementary file 9 — Source data Fig. 3 [file 44319_2024_335_MOESM9_ESM.zip › Figure 3/3I/Image002.tif]

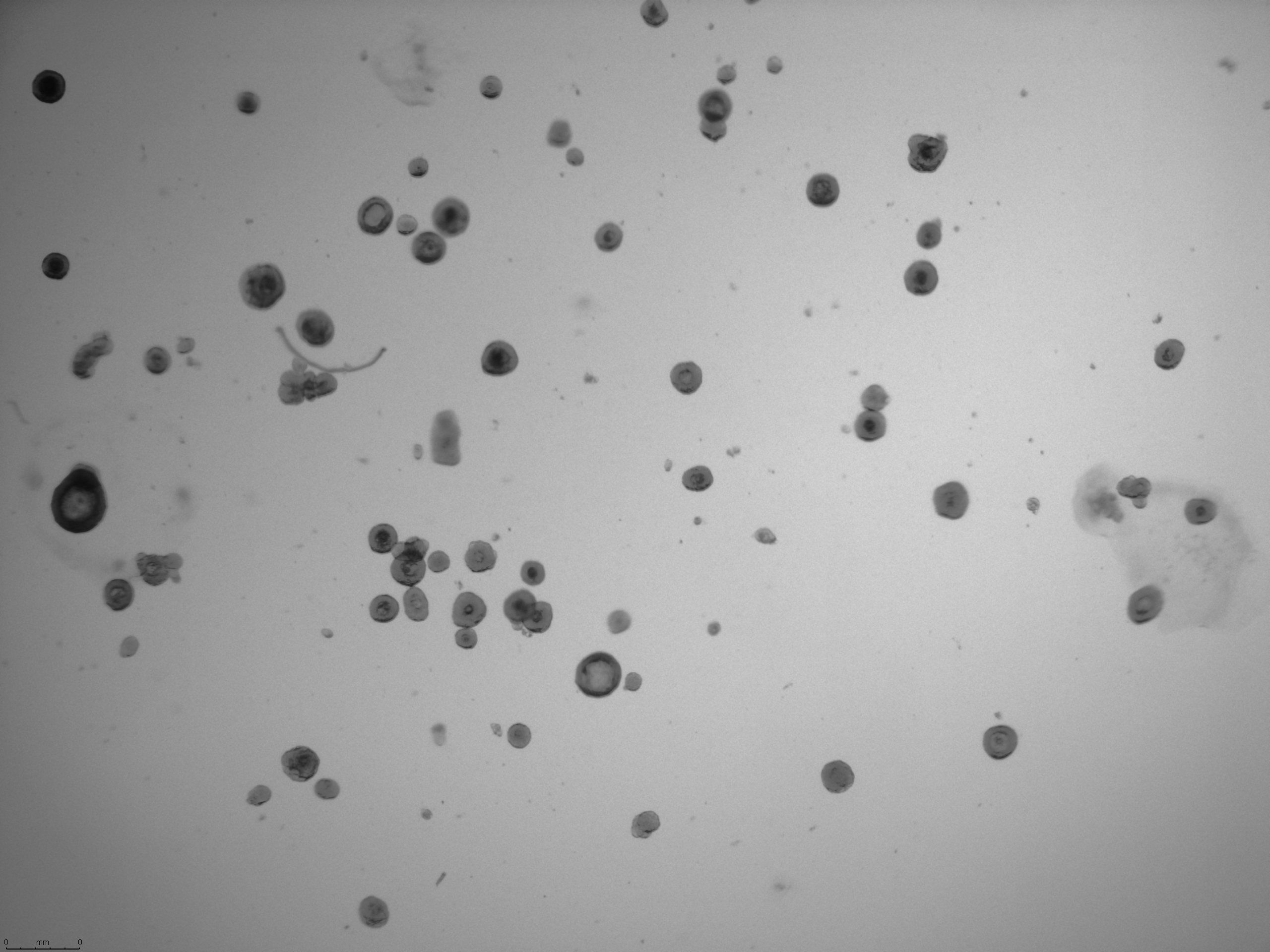

Supplement: Supplementary file 9 — Source data Fig. 3 [file 44319_2024_335_MOESM9_ESM.zip › Figure 3/3I/Image001.tif]

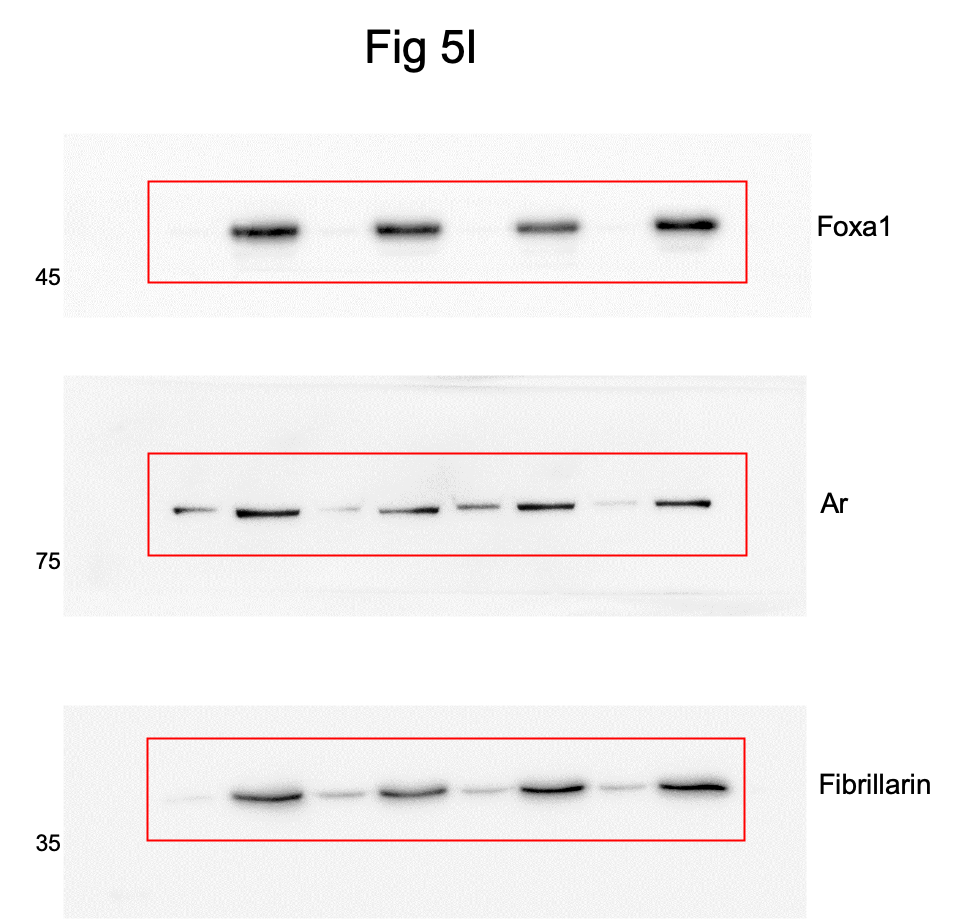

Supplement: Supplementary file 10 — Source data Fig. 5 [file 44319_2024_335_MOESM10_ESM.zip › Figure 5/5I/5I.png]
