# Supplementary figures and images for: Rarγ-Foxa1 signaling promotes luminal identity in prostate progenitors and is disrupted in prostate cancer (part 2 of 2)
Source: EMBO Rep. 2024 Dec 4;26(2):443–69. doi: 10.1038/s44319-024-00335-y (PMC11772605; doi:10.1038/s44319-024-00335-y)

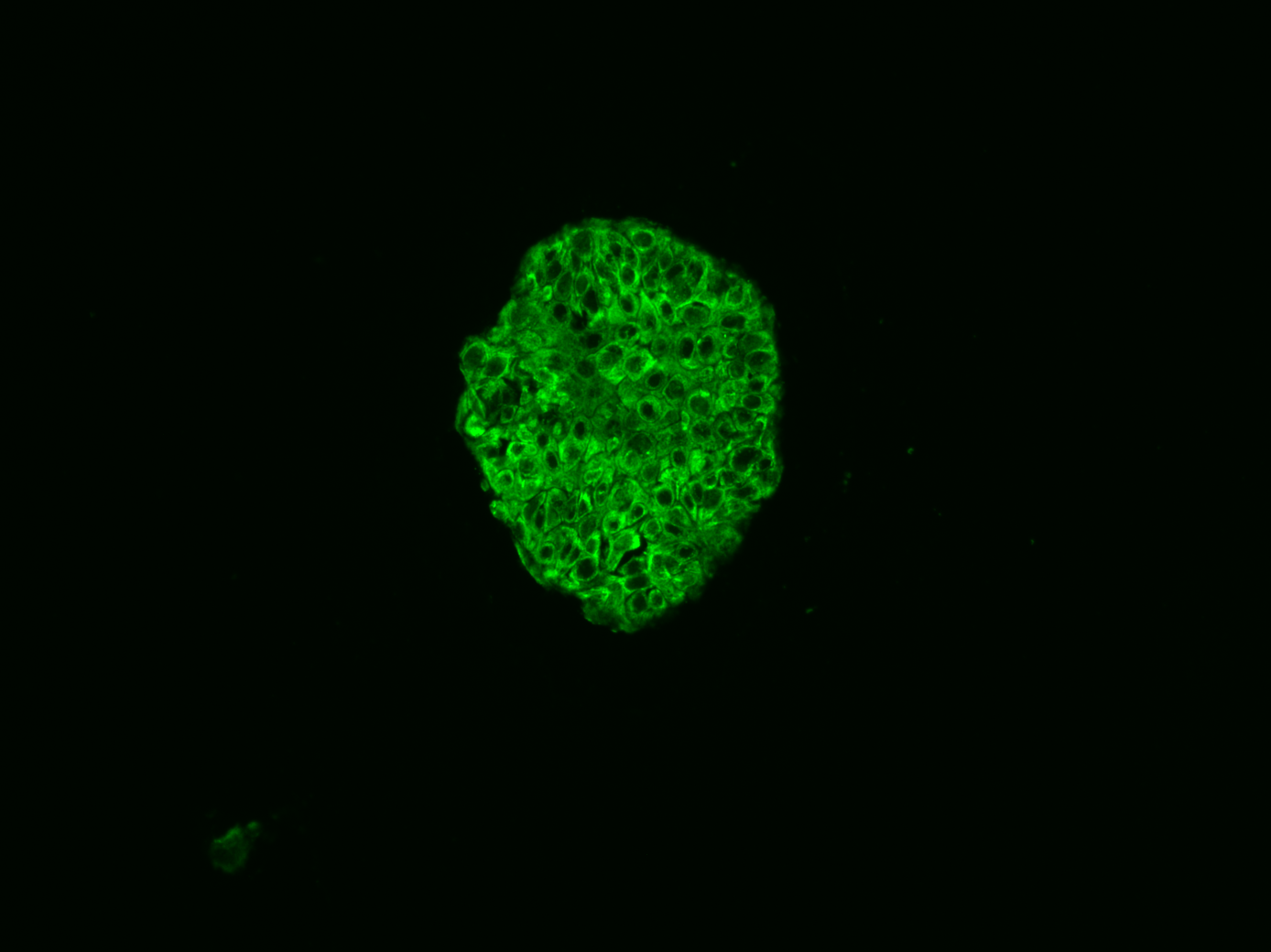

Supplement: Supplementary file 10 — Source data Fig. 5 [file 44319_2024_335_MOESM10_ESM.zip › Figure 5/5G/F254E255 Ck5 1.tif]

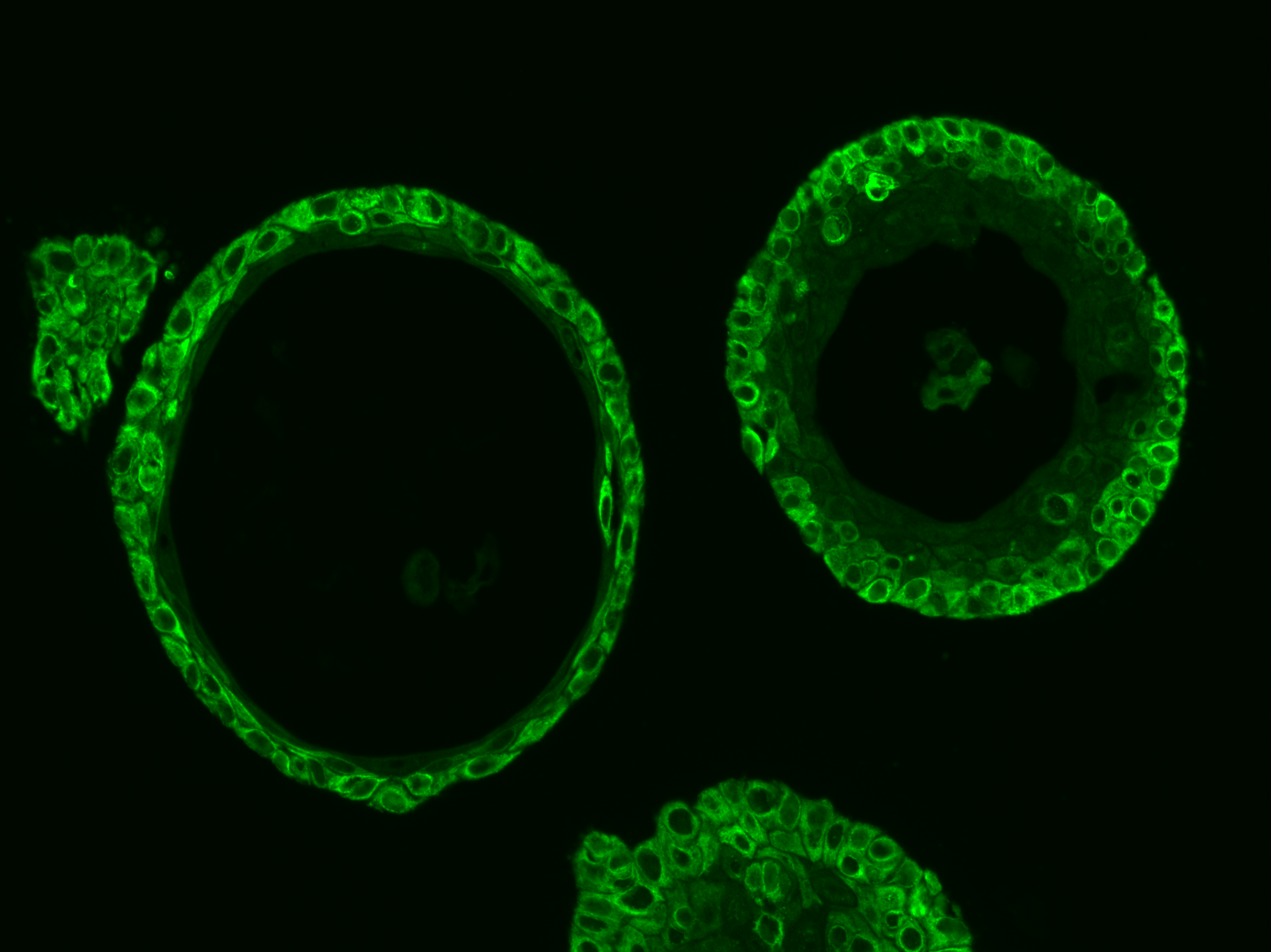

Supplement: Supplementary file 10 — Source data Fig. 5 [file 44319_2024_335_MOESM10_ESM.zip › Figure 5/5G/F254E255 Ck5 2.tif]

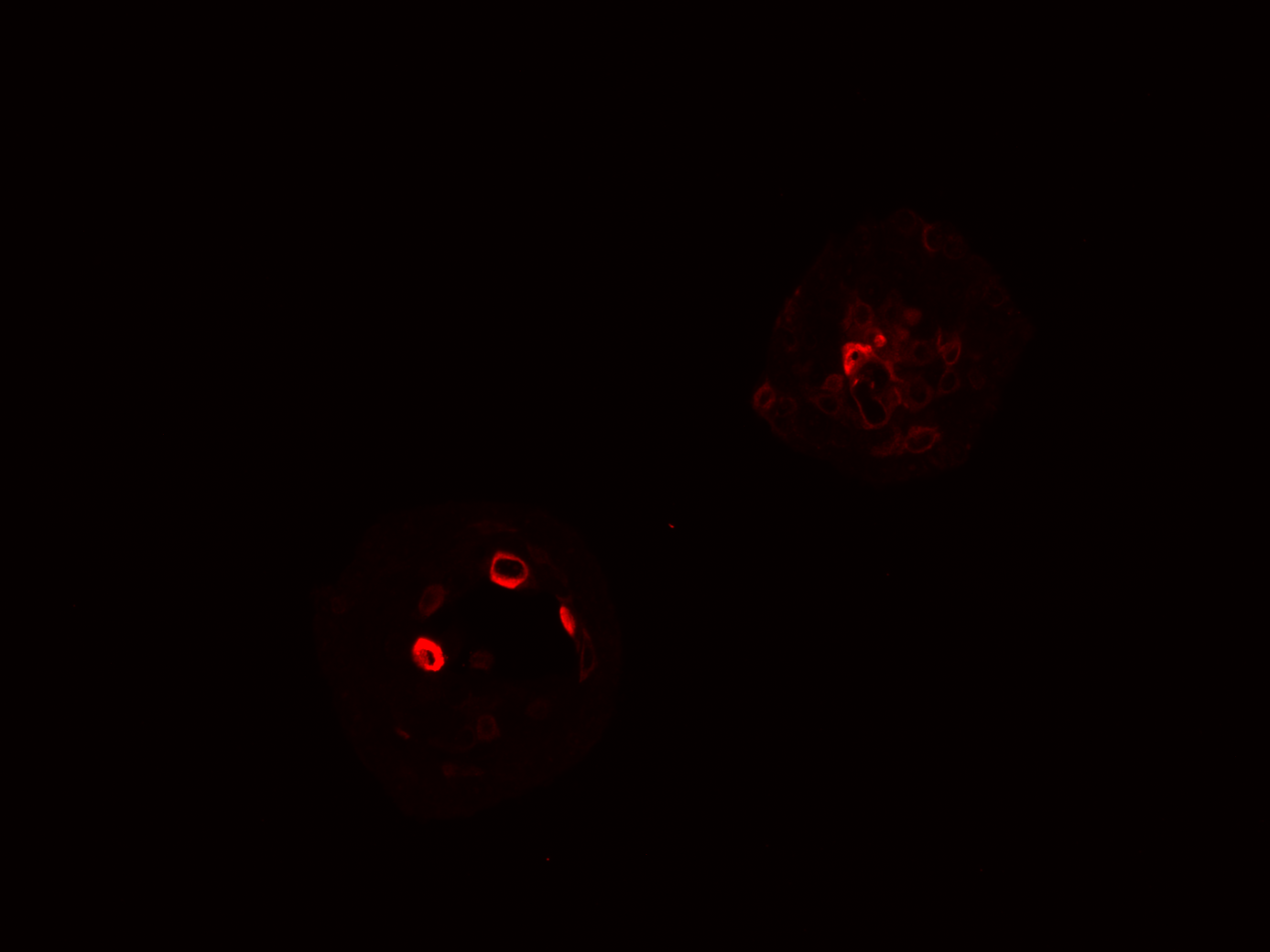

Supplement: Supplementary file 10 — Source data Fig. 5 [file 44319_2024_335_MOESM10_ESM.zip › Figure 5/5G/H247Q Ck8 1.tif]

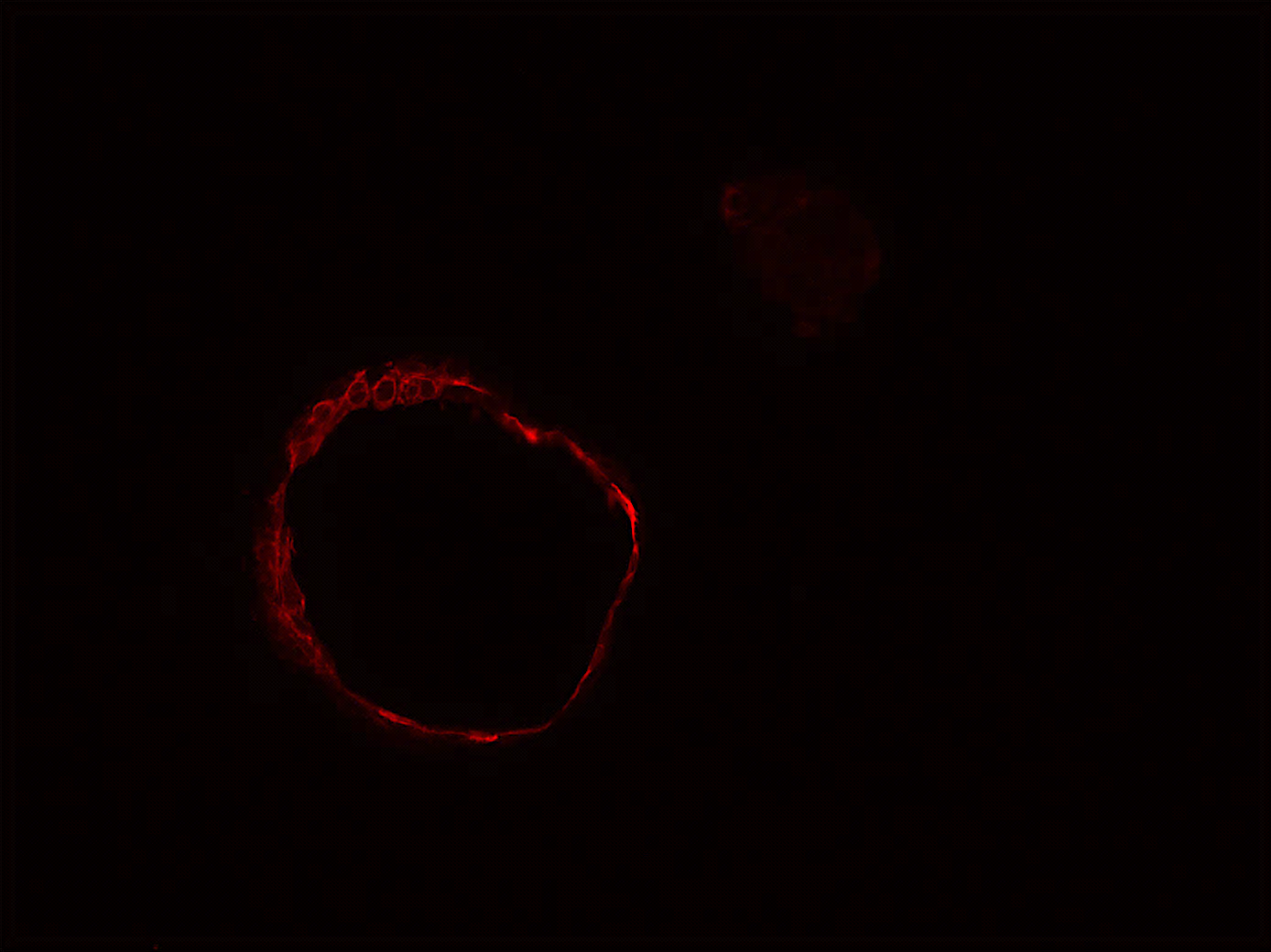

Supplement: Supplementary file 10 — Source data Fig. 5 [file 44319_2024_335_MOESM10_ESM.zip › Figure 5/5G/H247Q Ck8 2.tif]

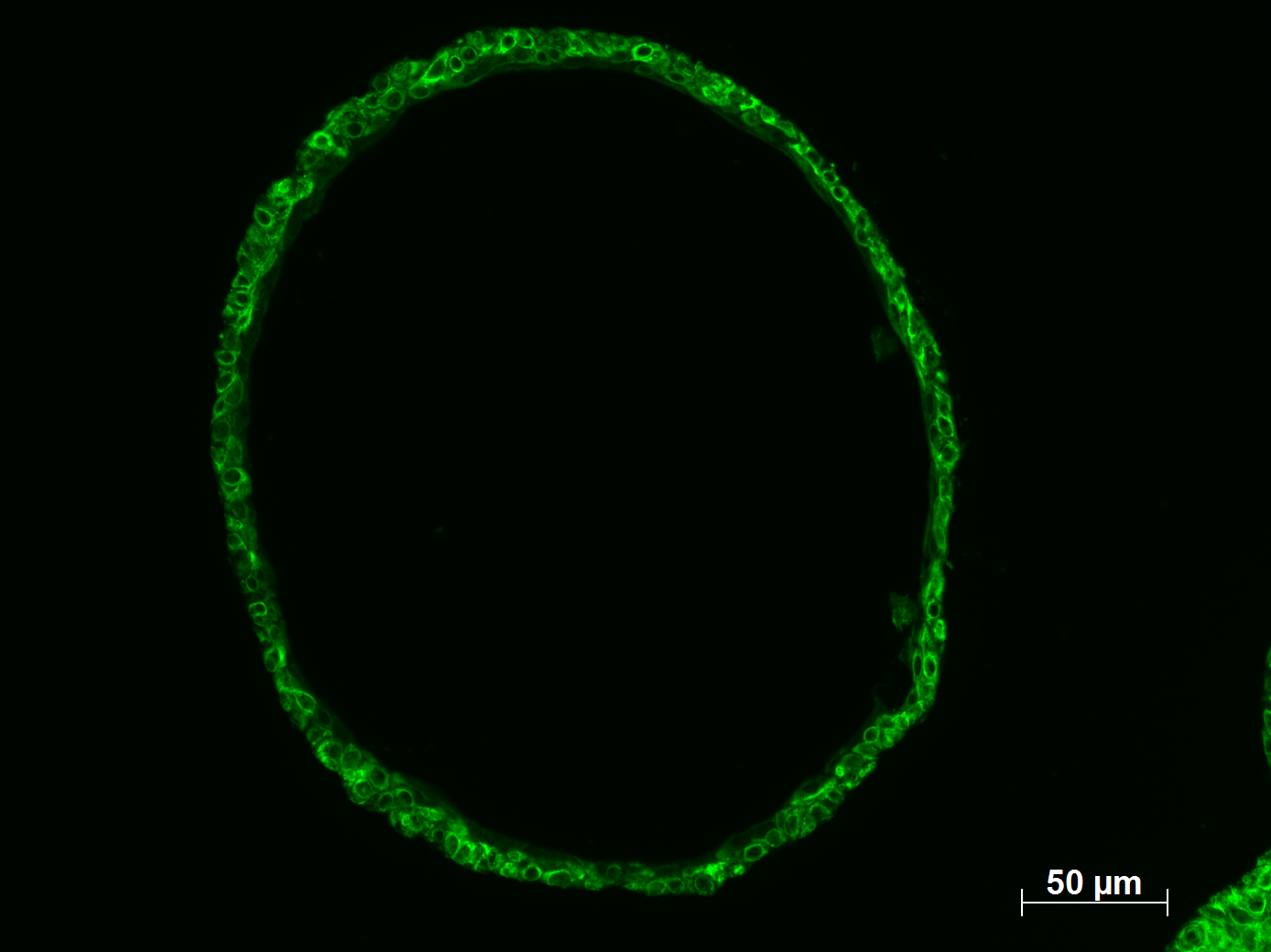

Supplement: Supplementary file 10 — Source data Fig. 5 [file 44319_2024_335_MOESM10_ESM.zip › Figure 5/5G/WT Ck5.tif]

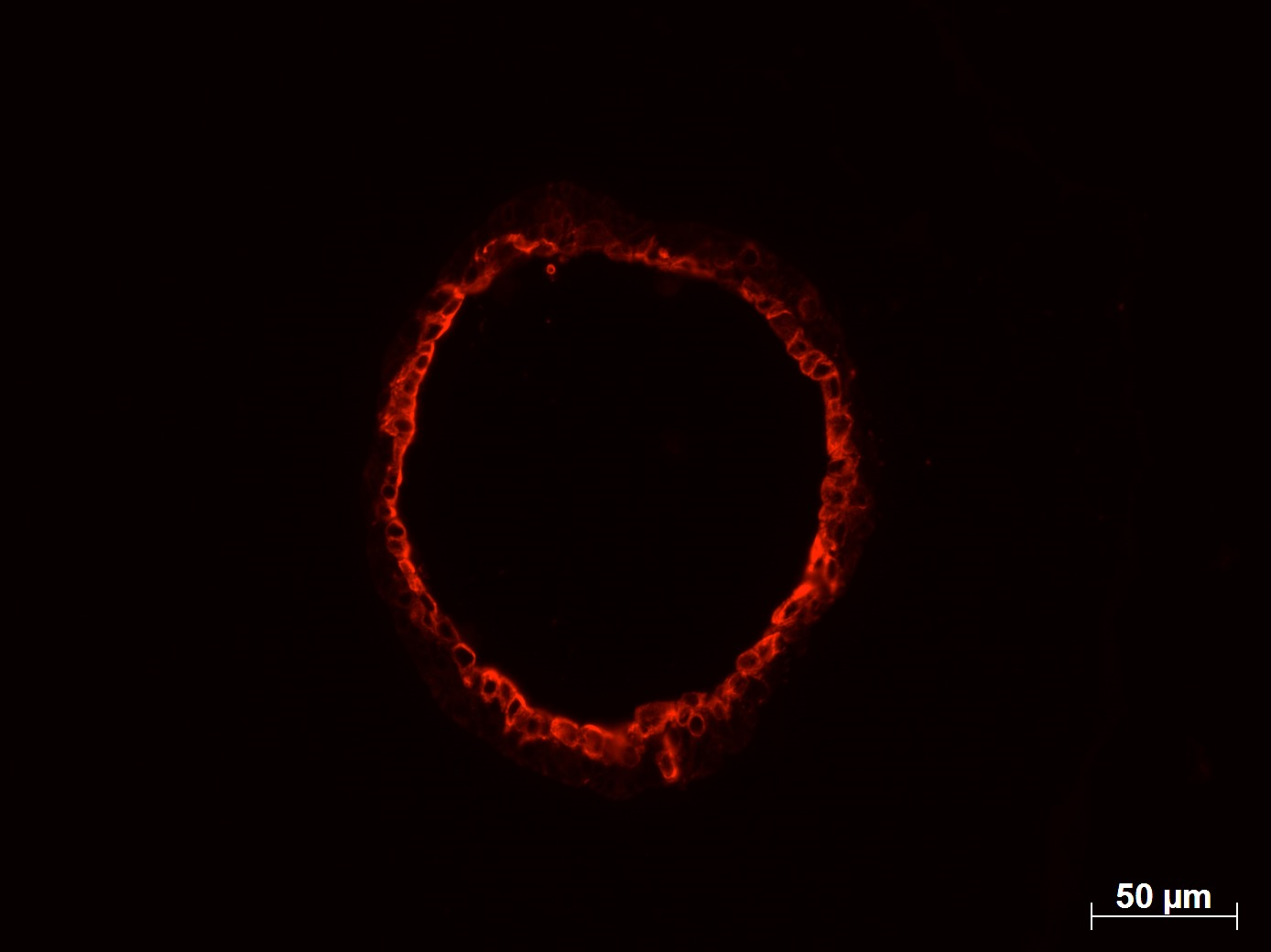

Supplement: Supplementary file 10 — Source data Fig. 5 [file 44319_2024_335_MOESM10_ESM.zip › Figure 5/5G/D226N Ck8 2.tif]

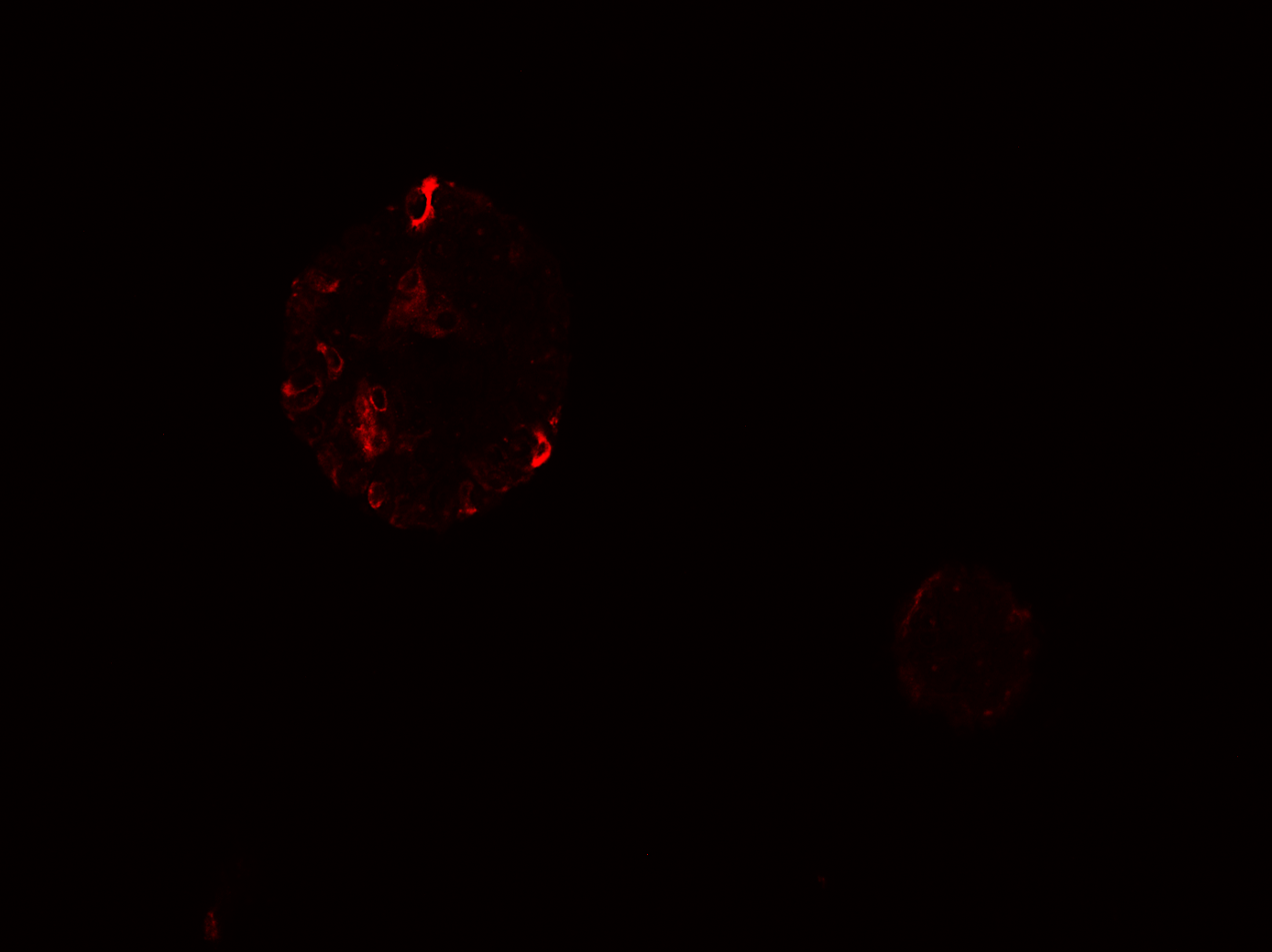

Supplement: Supplementary file 10 — Source data Fig. 5 [file 44319_2024_335_MOESM10_ESM.zip › Figure 5/5G/D226N Ck8 1.tif]

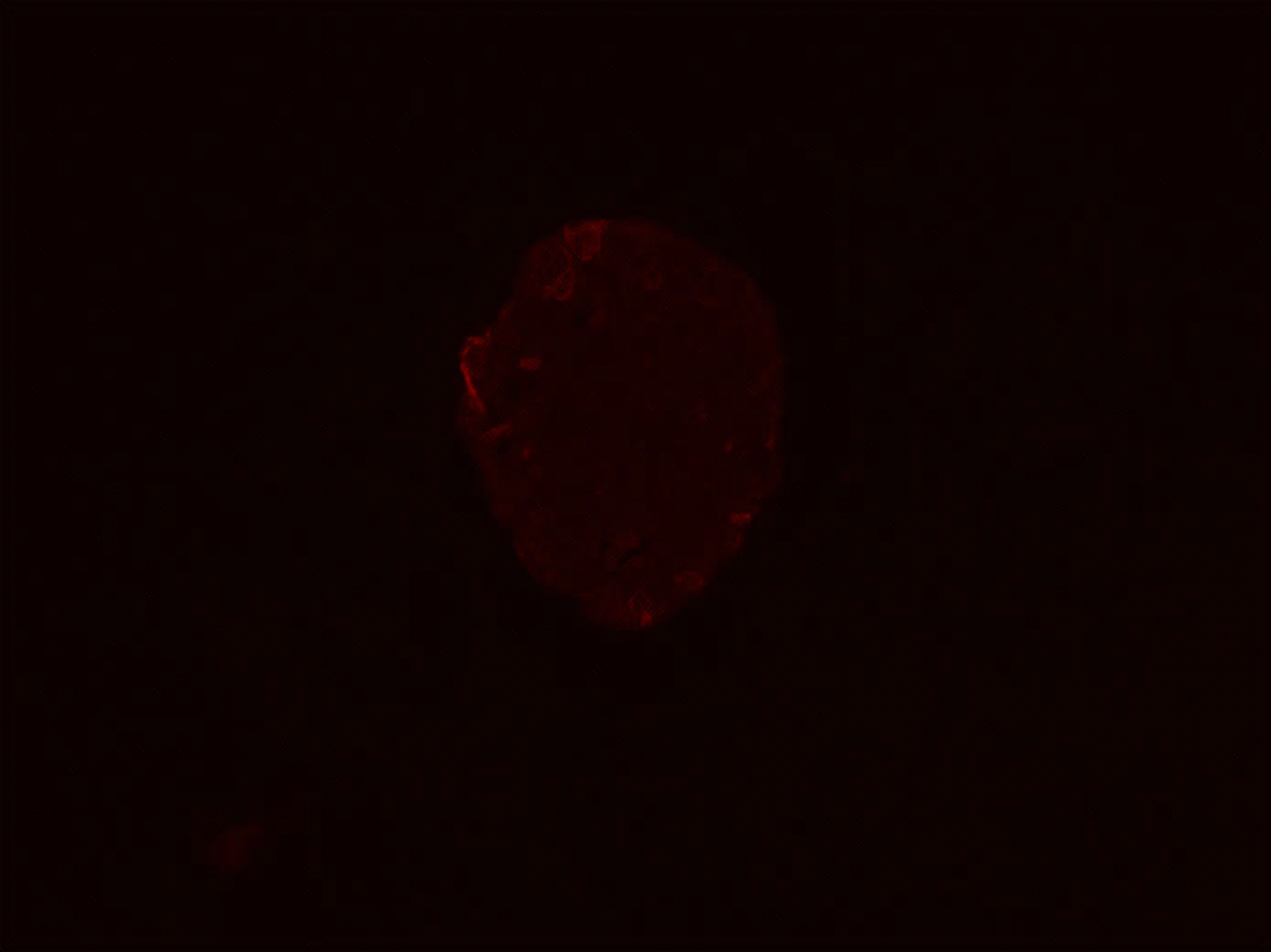

Supplement: Supplementary file 10 — Source data Fig. 5 [file 44319_2024_335_MOESM10_ESM.zip › Figure 5/5G/F254E255 Ck8 1.tif]

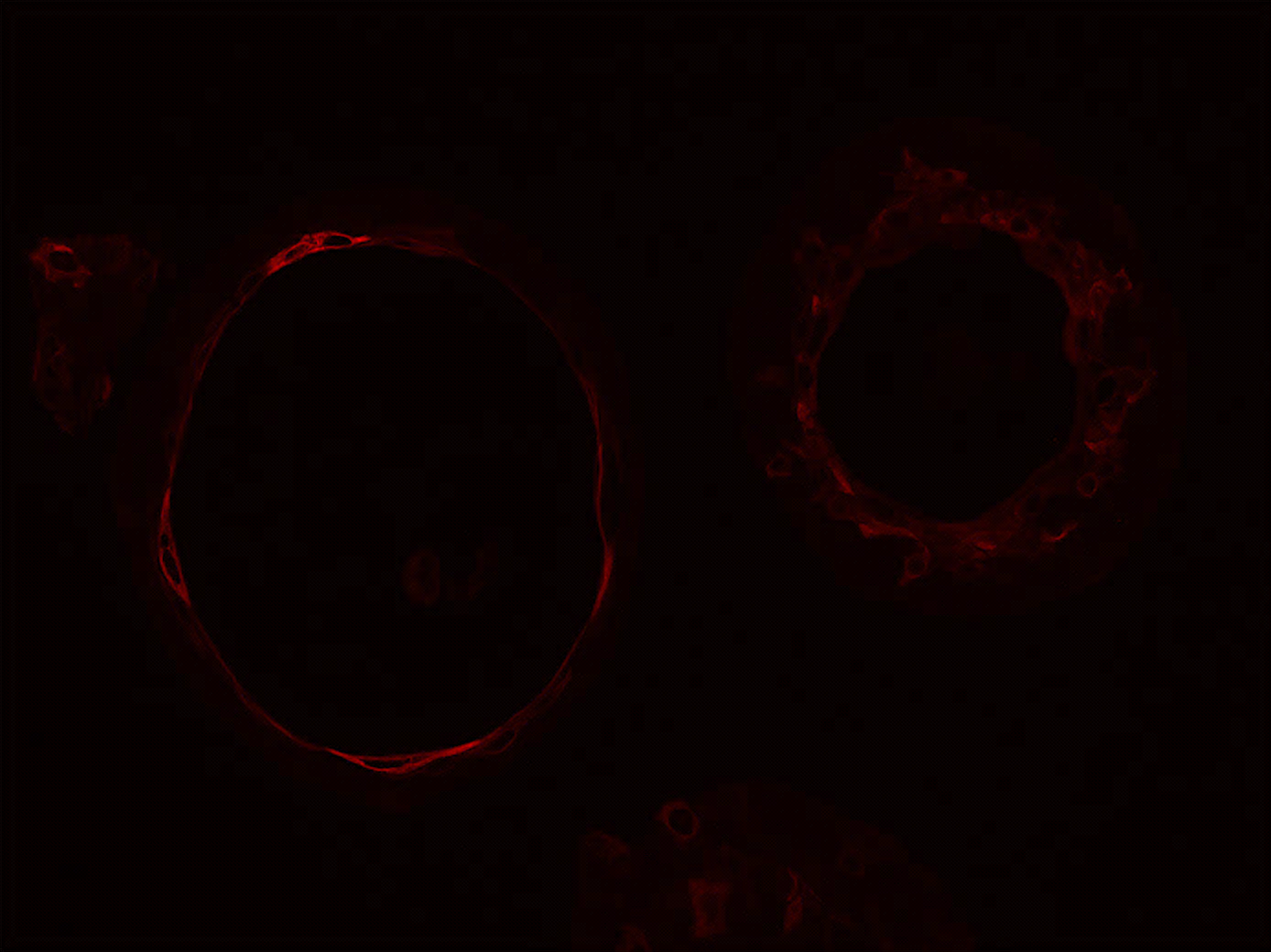

Supplement: Supplementary file 10 — Source data Fig. 5 [file 44319_2024_335_MOESM10_ESM.zip › Figure 5/5G/F254E255 Ck8 2.tif]

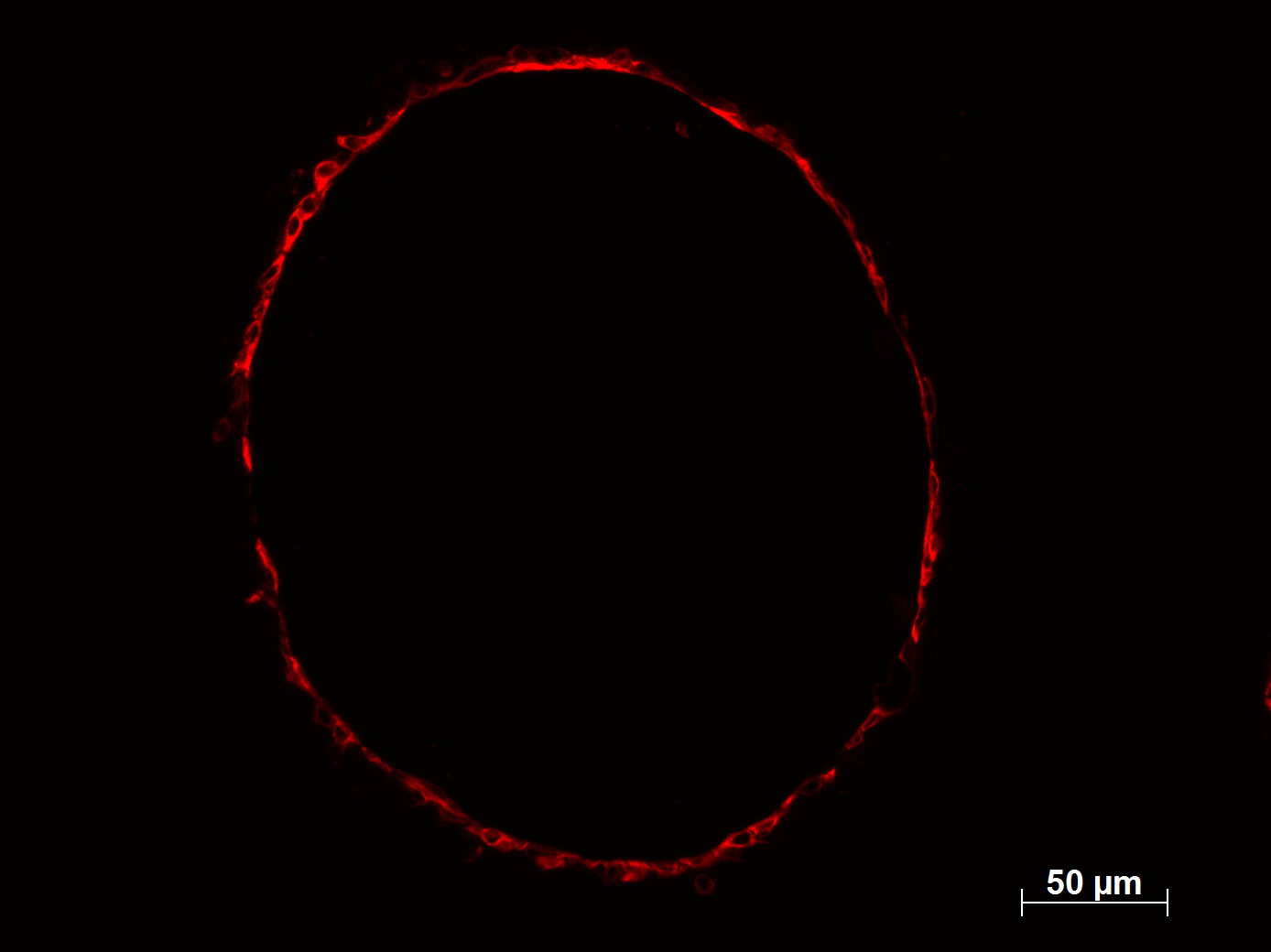

Supplement: Supplementary file 10 — Source data Fig. 5 [file 44319_2024_335_MOESM10_ESM.zip › Figure 5/5G/WT Ck8.tif]

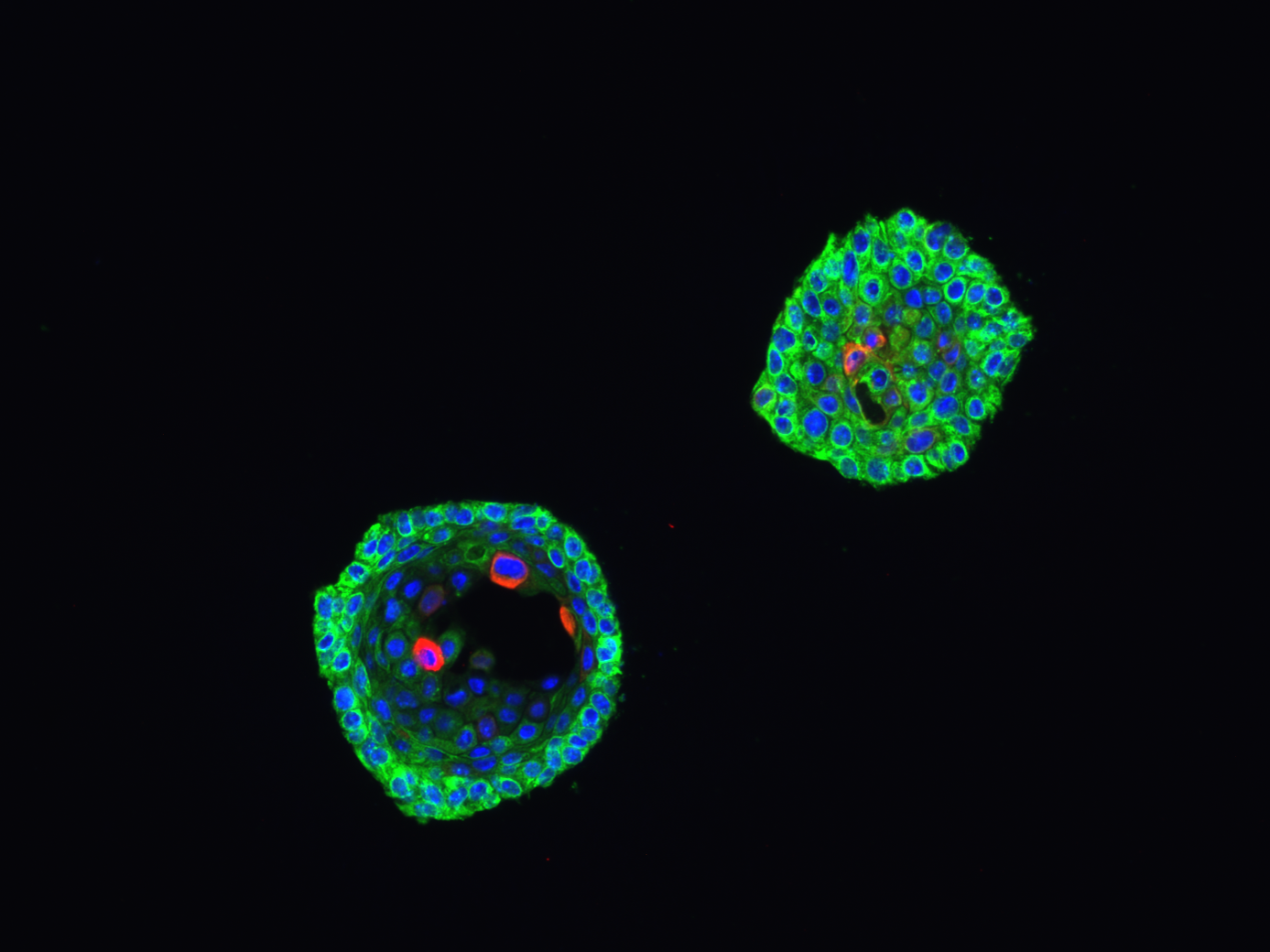

Supplement: Supplementary file 10 — Source data Fig. 5 [file 44319_2024_335_MOESM10_ESM.zip › Figure 5/5G/H247Q merge 1.tif]

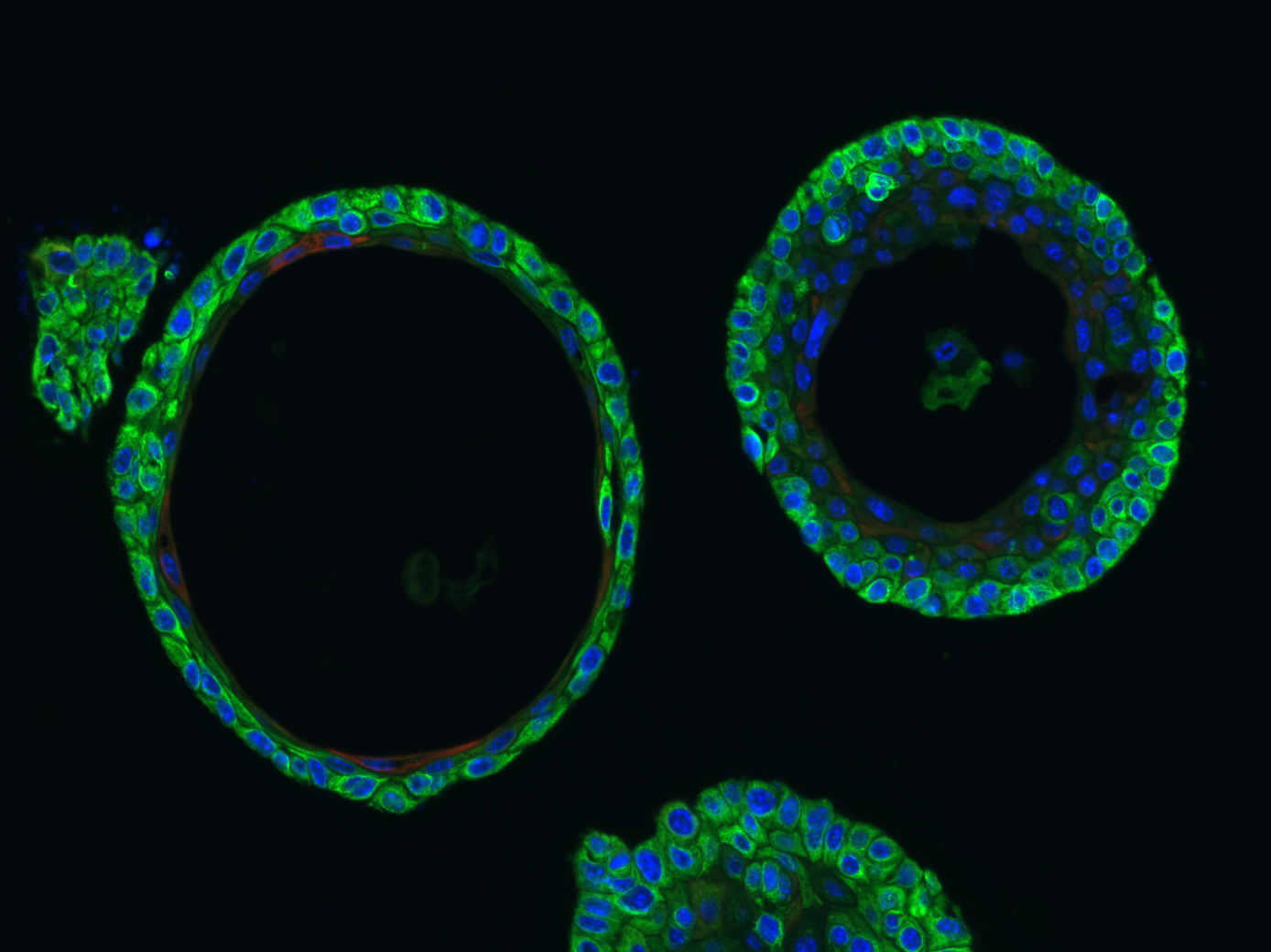

Supplement: Supplementary file 10 — Source data Fig. 5 [file 44319_2024_335_MOESM10_ESM.zip › Figure 5/5G/F254E255 merge 2.tif]

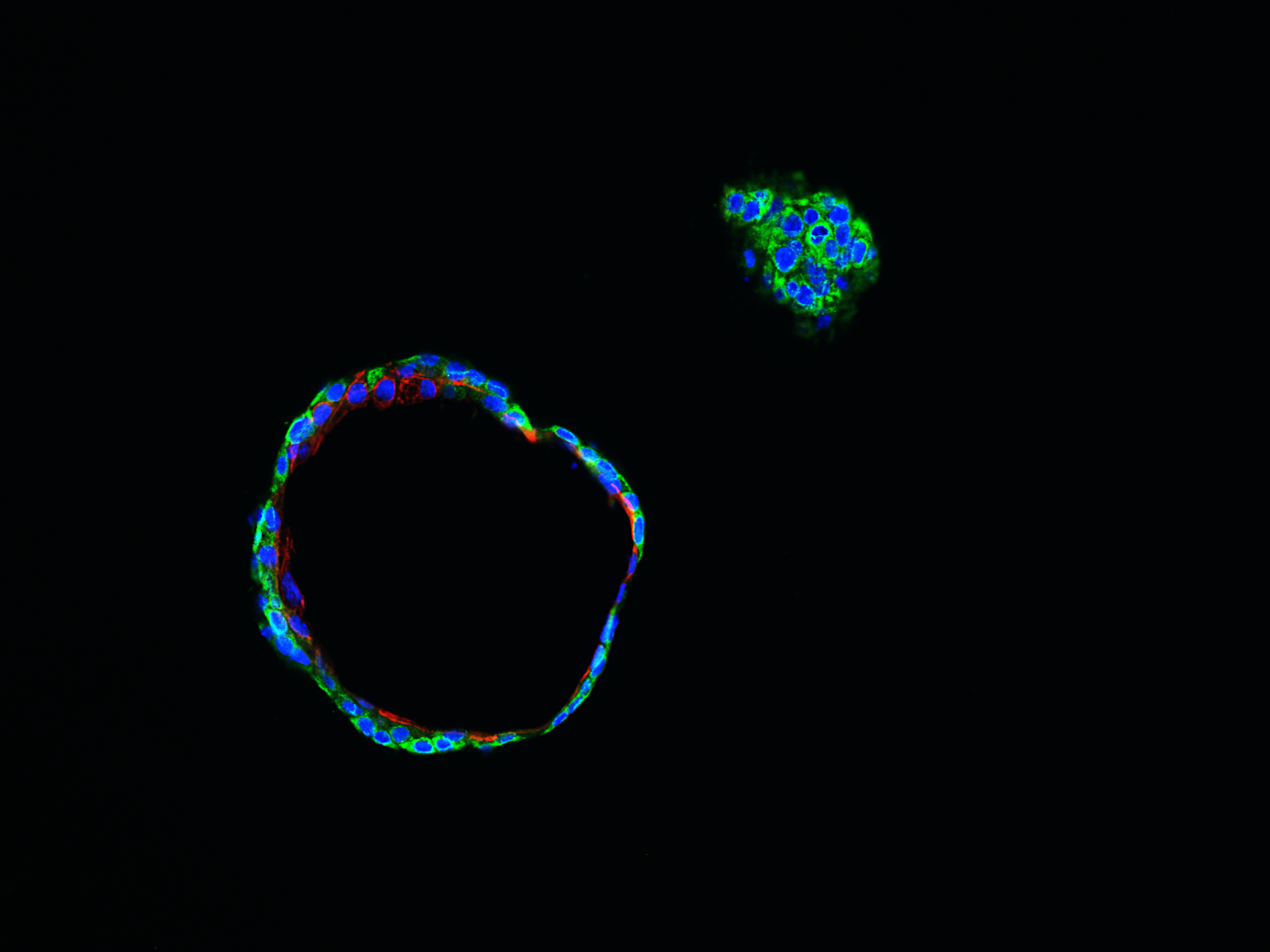

Supplement: Supplementary file 10 — Source data Fig. 5 [file 44319_2024_335_MOESM10_ESM.zip › Figure 5/5G/H247Q merge 2.tif]

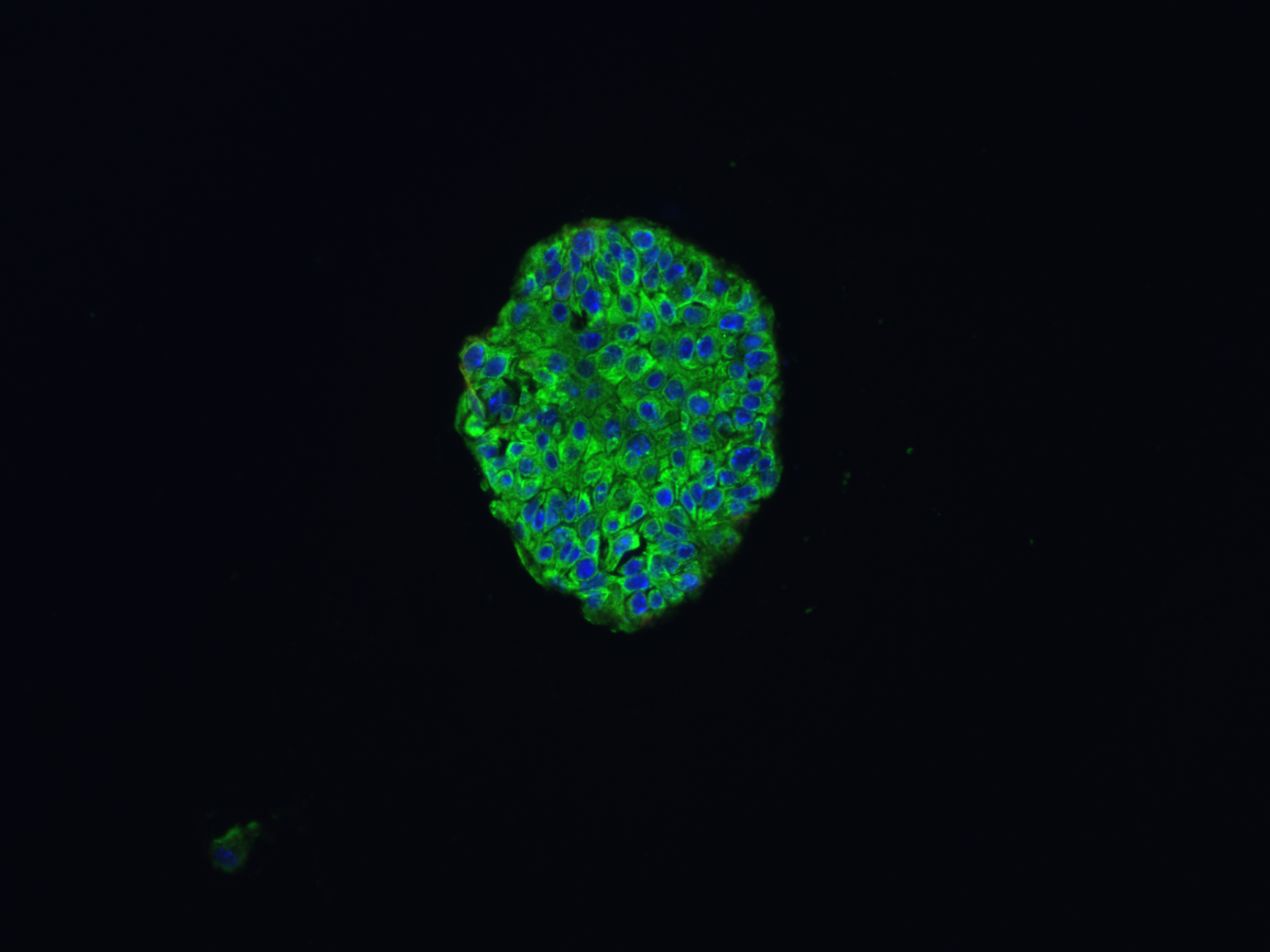

Supplement: Supplementary file 10 — Source data Fig. 5 [file 44319_2024_335_MOESM10_ESM.zip › Figure 5/5G/F254E255 merge 1.tif]

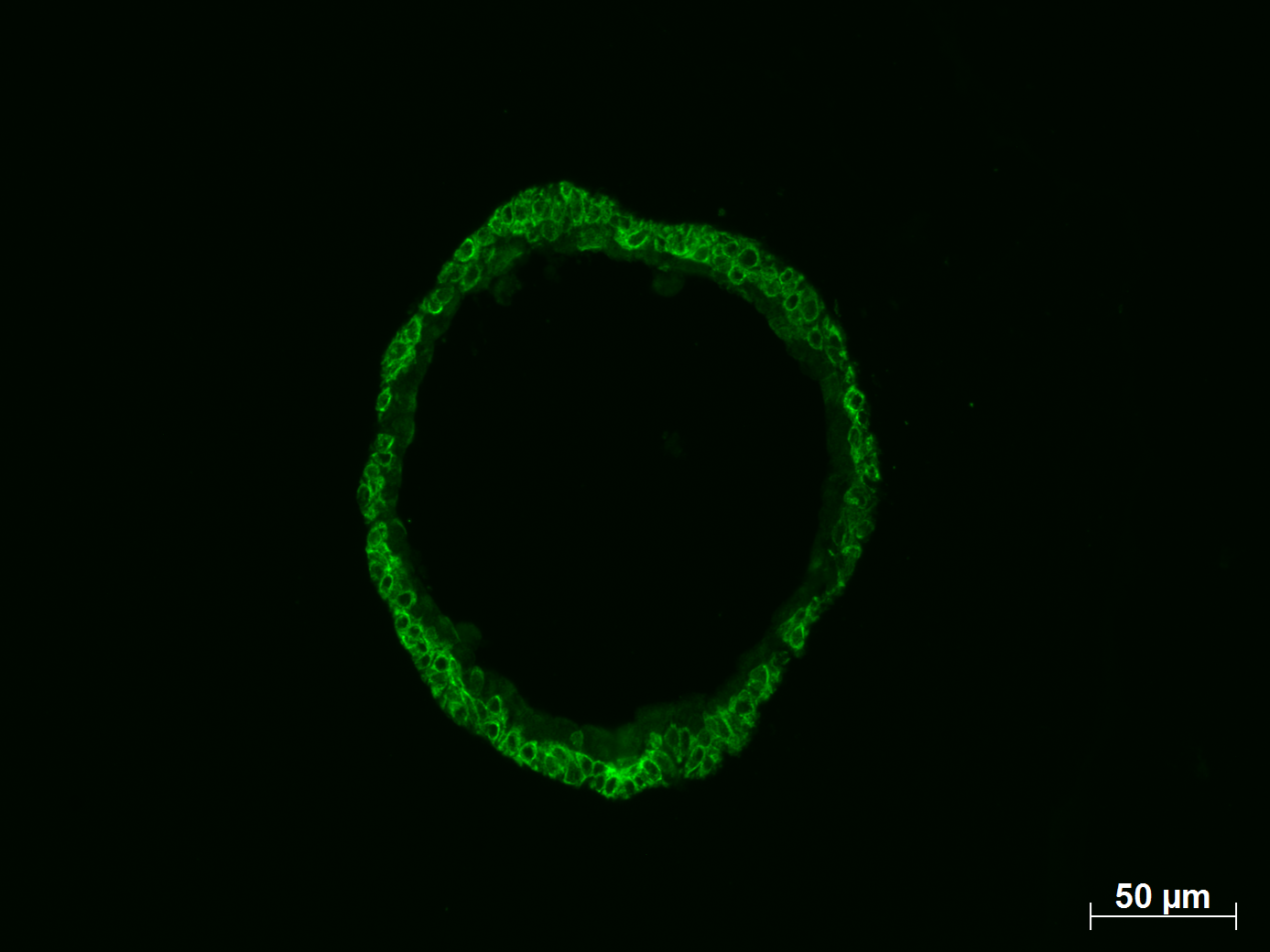

Supplement: Supplementary file 10 — Source data Fig. 5 [file 44319_2024_335_MOESM10_ESM.zip › Figure 5/5G/D226N Ck5 2.tif]

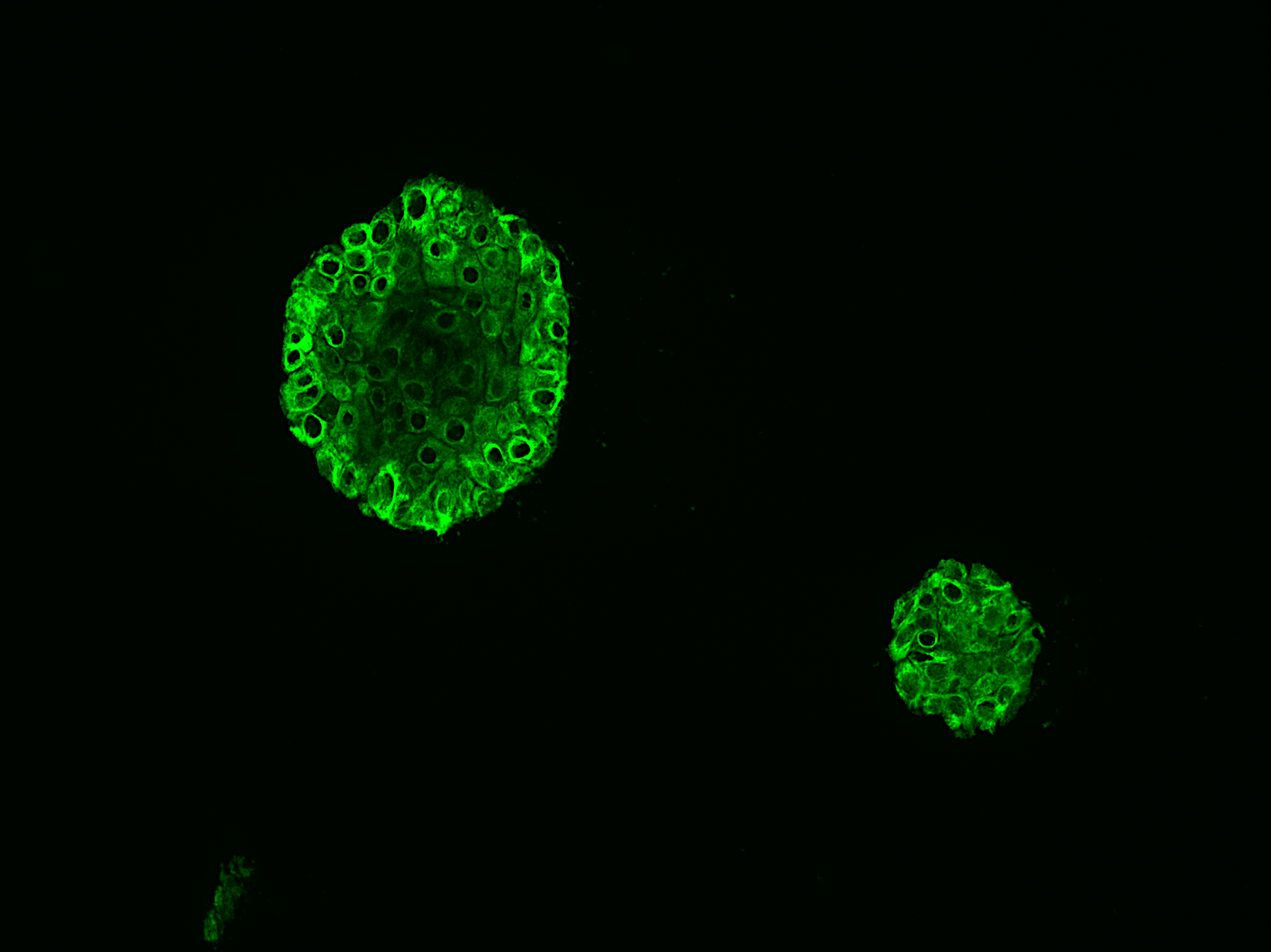

Supplement: Supplementary file 10 — Source data Fig. 5 [file 44319_2024_335_MOESM10_ESM.zip › Figure 5/5G/D226N Ck5 1.tif]

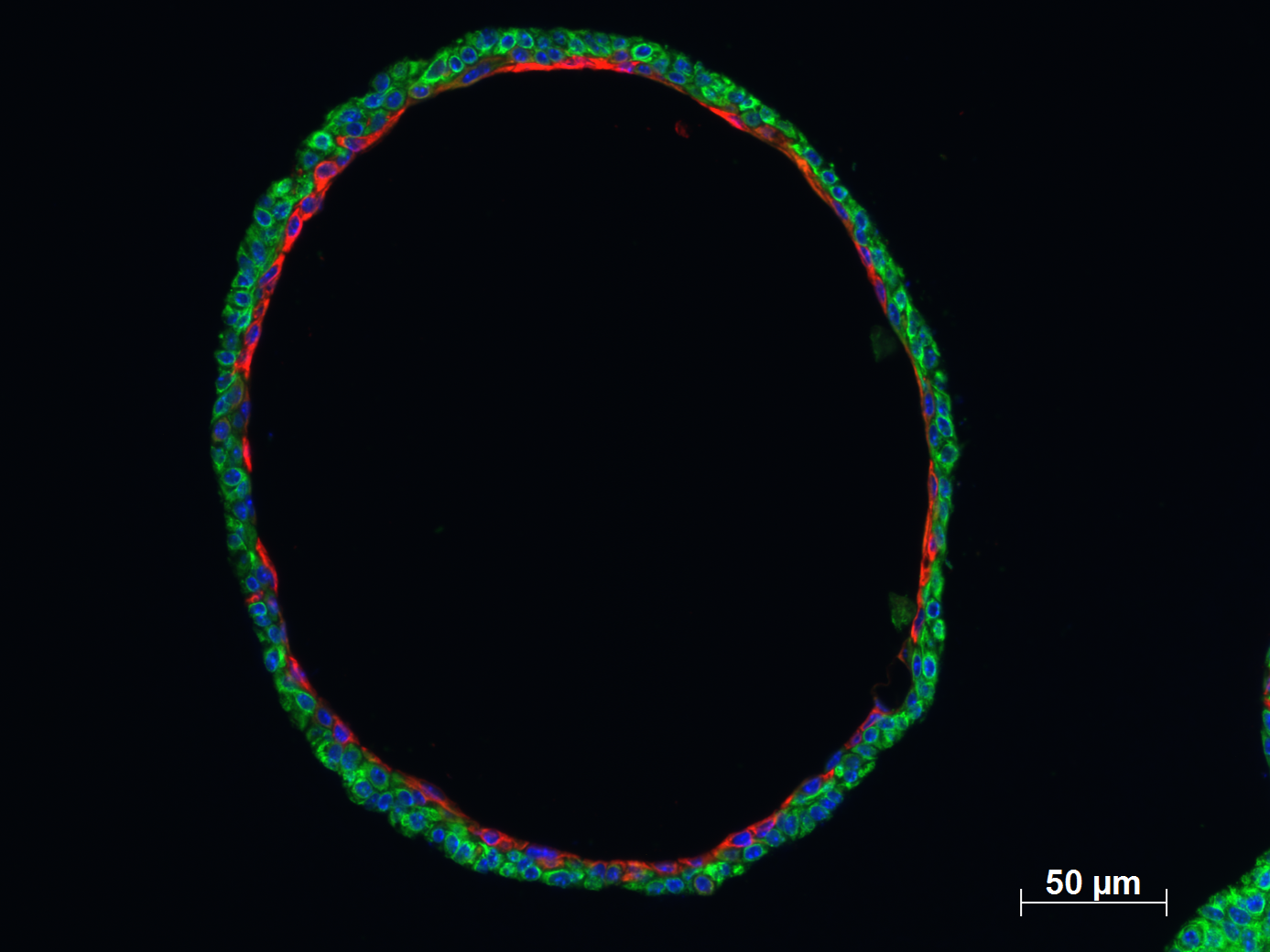

Supplement: Supplementary file 10 — Source data Fig. 5 [file 44319_2024_335_MOESM10_ESM.zip › Figure 5/5G/WT merge.tif]

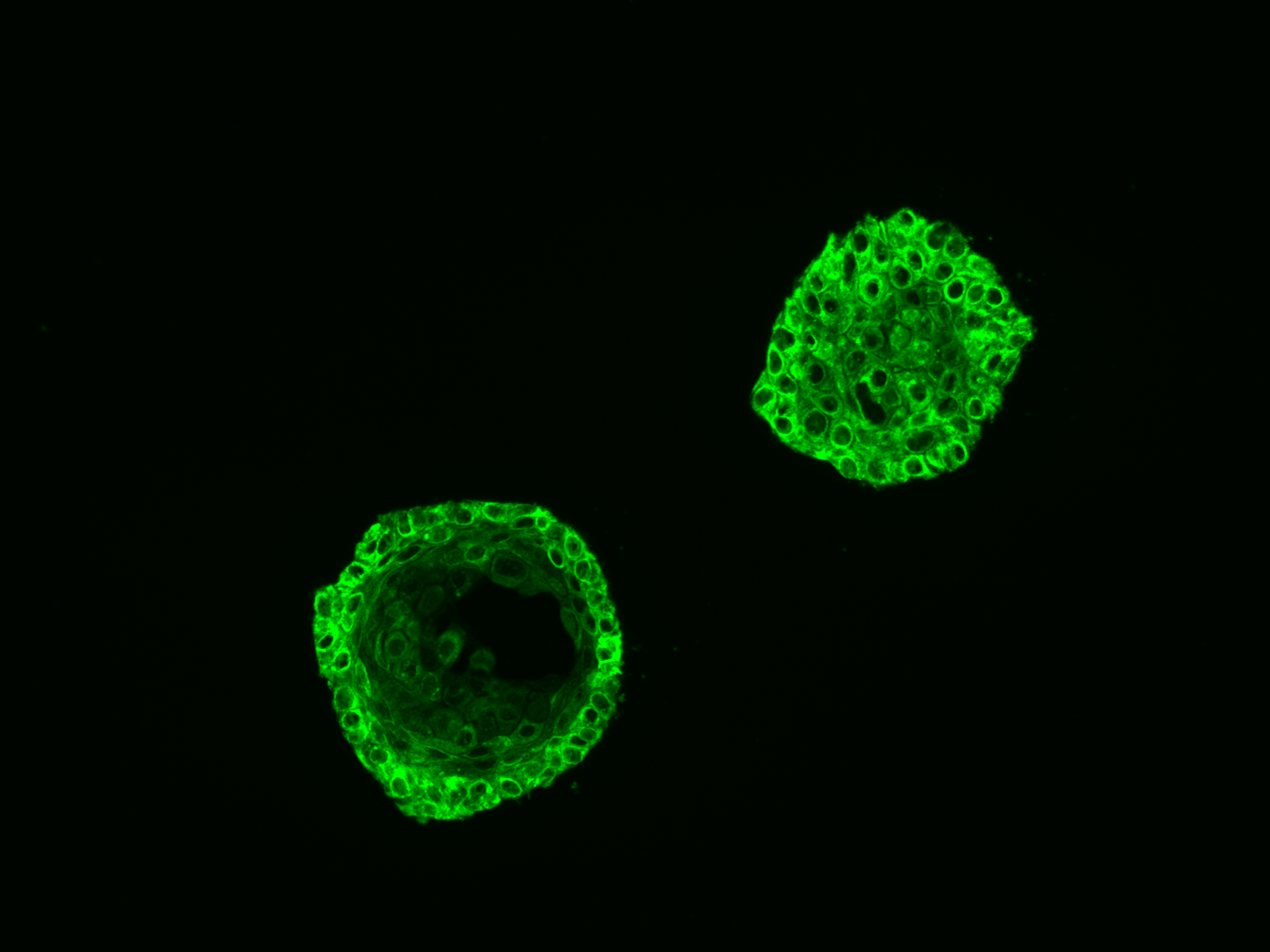

Supplement: Supplementary file 10 — Source data Fig. 5 [file 44319_2024_335_MOESM10_ESM.zip › Figure 5/5G/H247Q Ck5 1.tif]

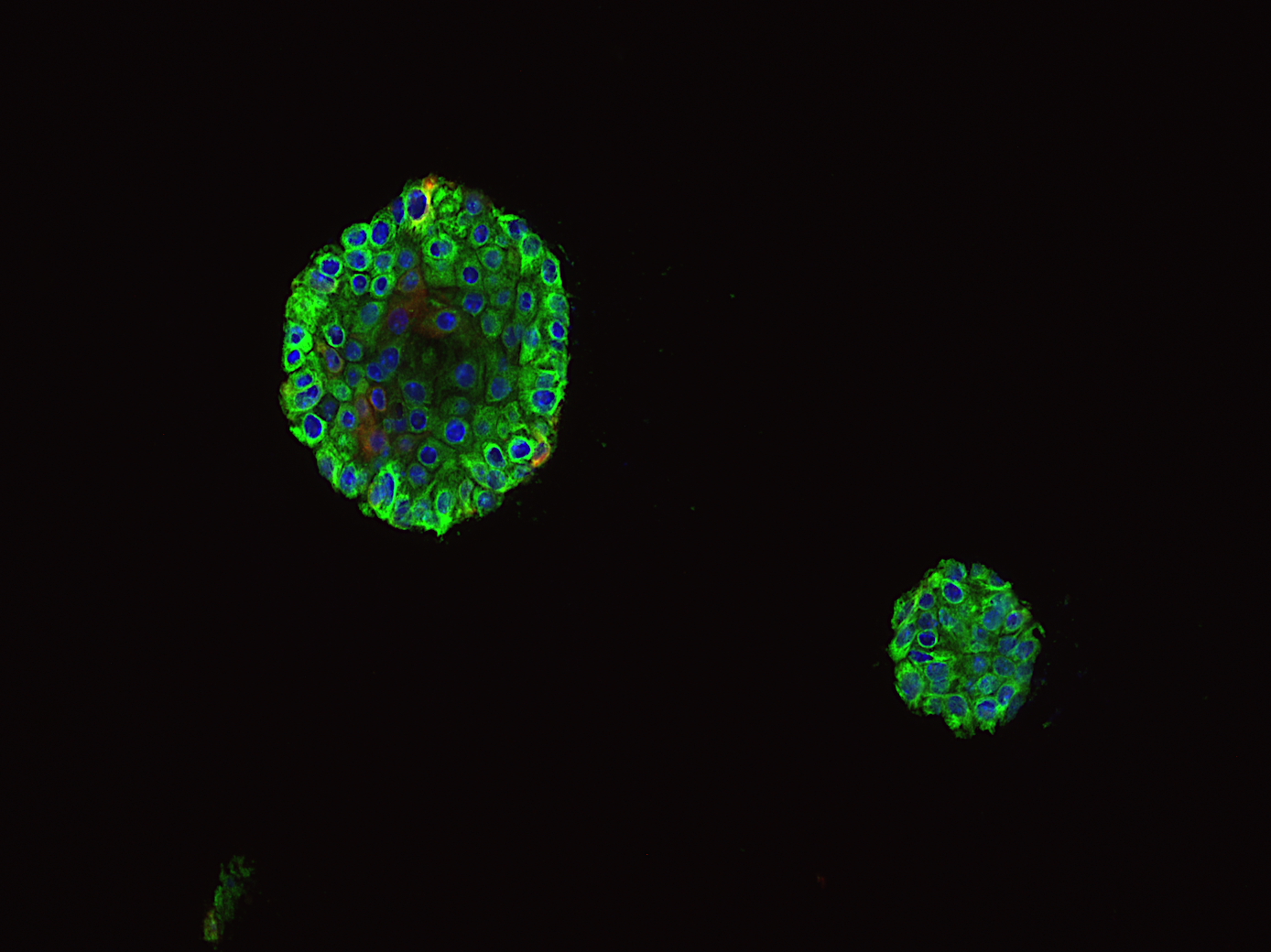

Supplement: Supplementary file 10 — Source data Fig. 5 [file 44319_2024_335_MOESM10_ESM.zip › Figure 5/5G/D226N merge 1.tif]

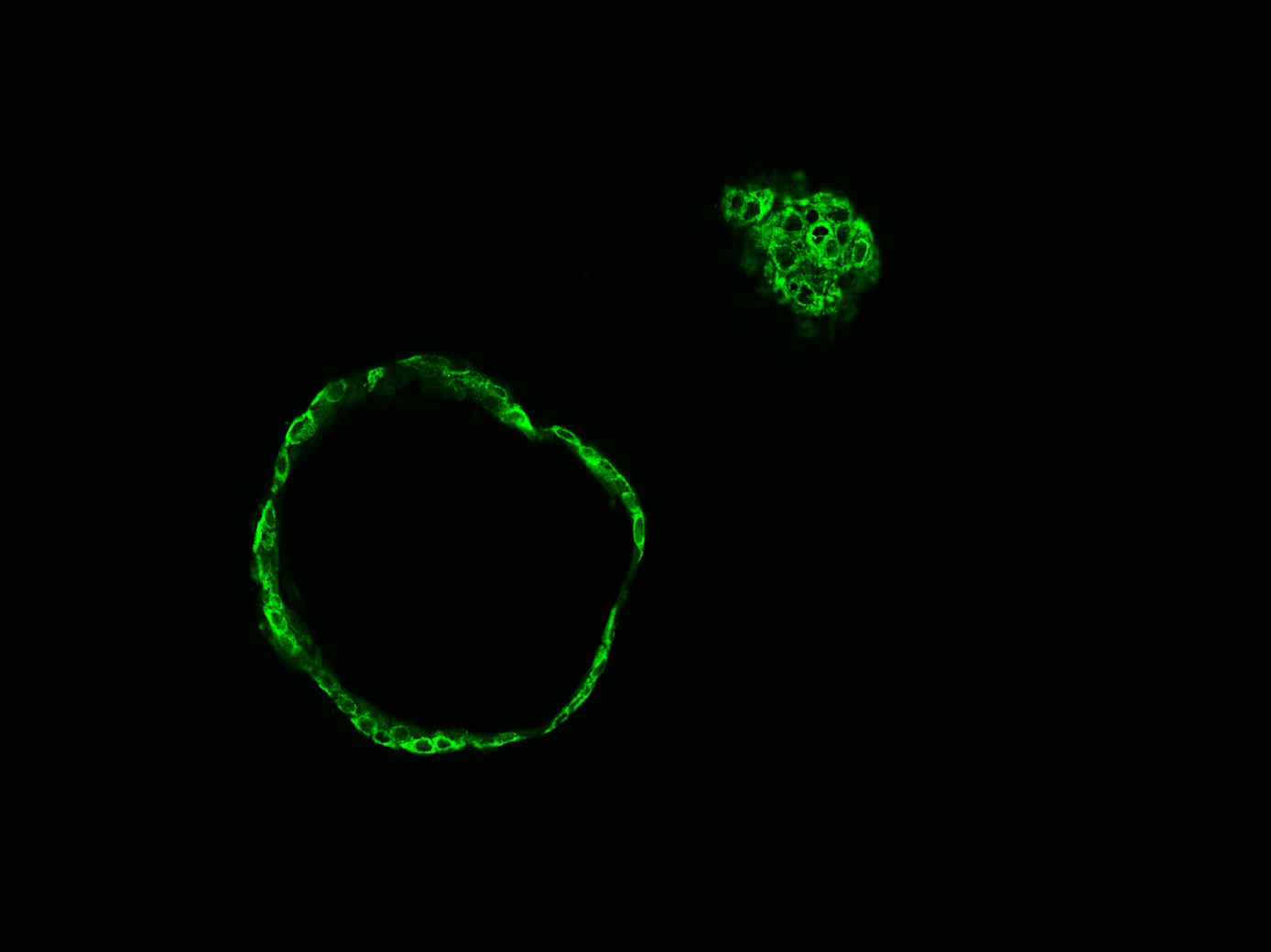

Supplement: Supplementary file 10 — Source data Fig. 5 [file 44319_2024_335_MOESM10_ESM.zip › Figure 5/5G/H247Q Ck5 2.tif]

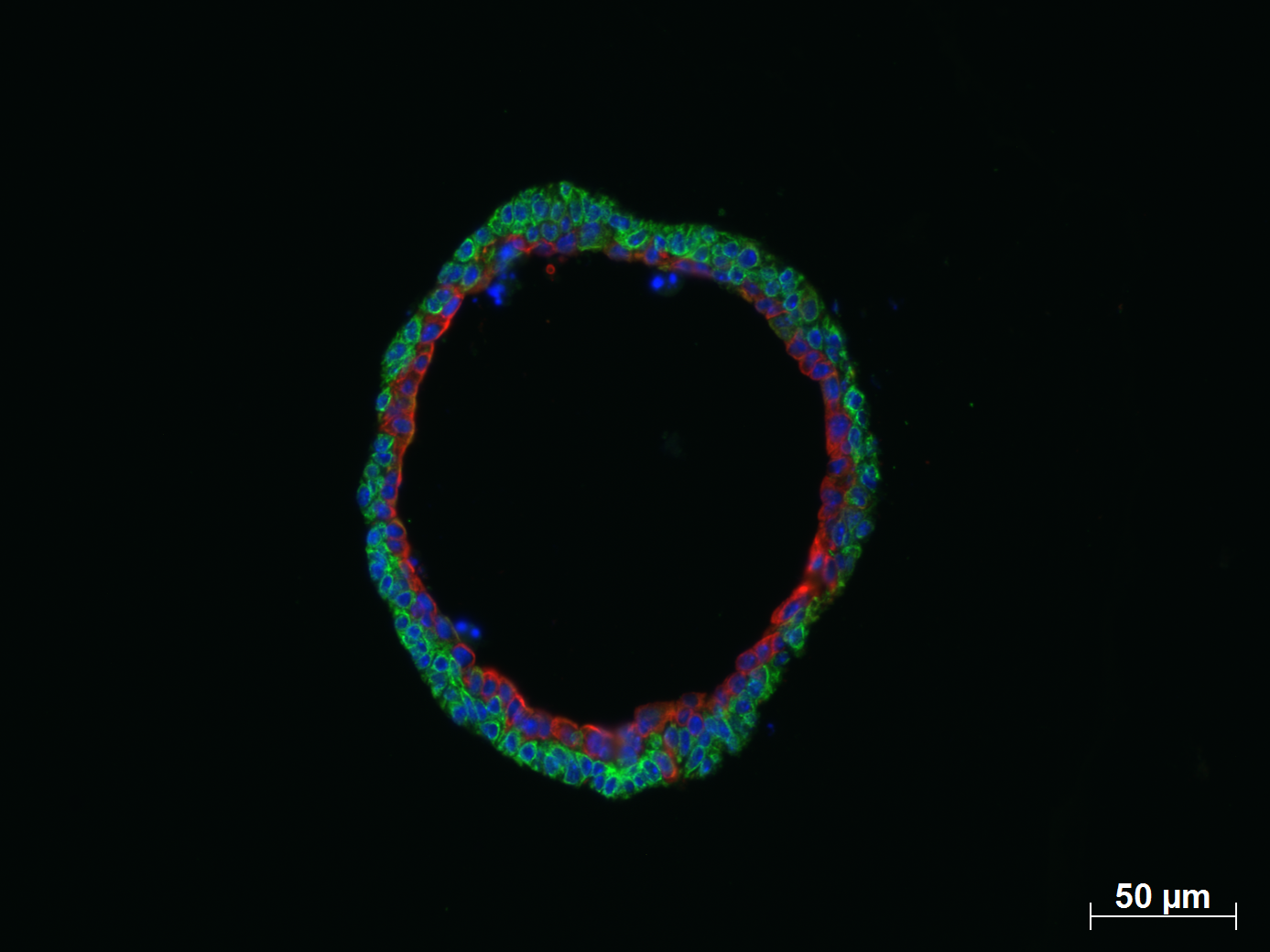

Supplement: Supplementary file 10 — Source data Fig. 5 [file 44319_2024_335_MOESM10_ESM.zip › Figure 5/5G/D226N merge 2.tif]

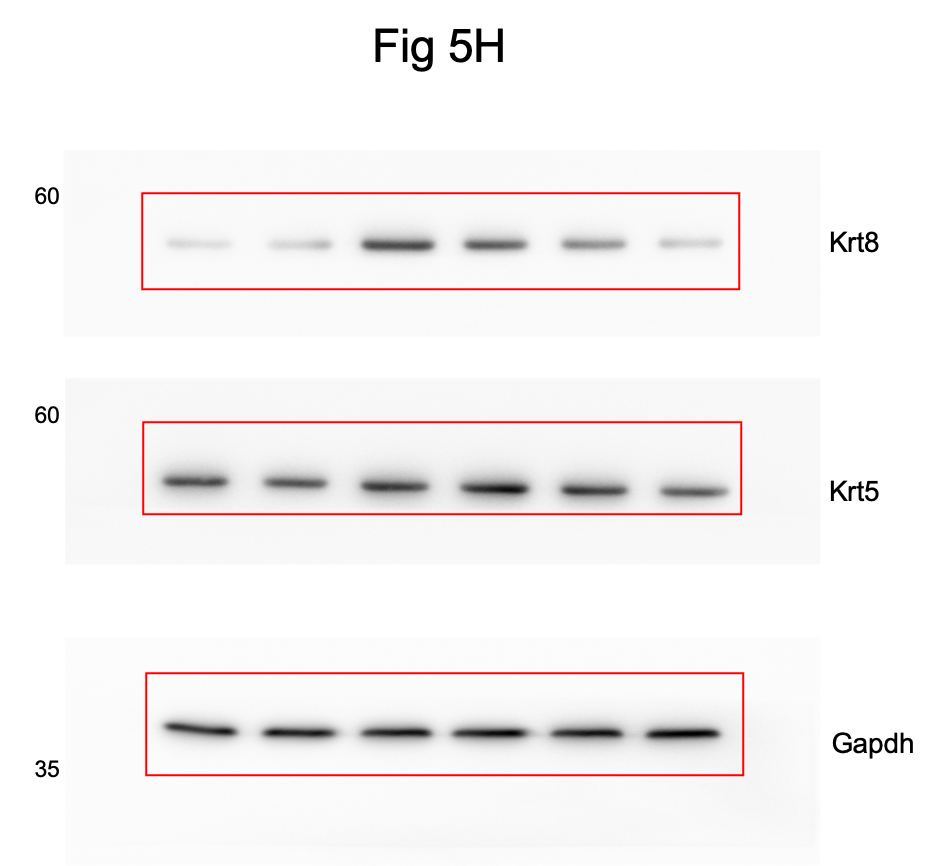

Supplement: Supplementary file 10 — Source data Fig. 5 [file 44319_2024_335_MOESM10_ESM.zip › Figure 5/5H/5H.png]

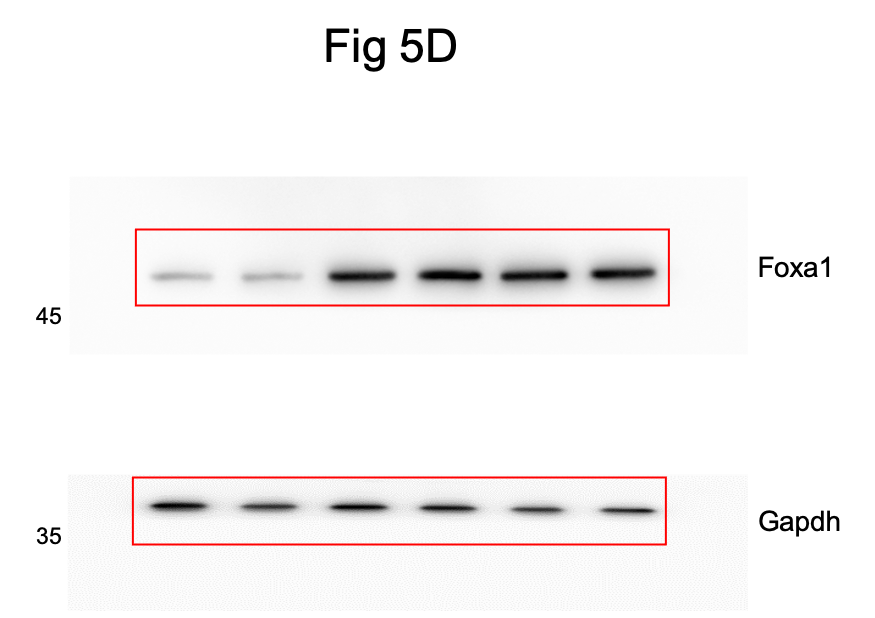

Supplement: Supplementary file 10 — Source data Fig. 5 [file 44319_2024_335_MOESM10_ESM.zip › Figure 5/5D/5D.png]

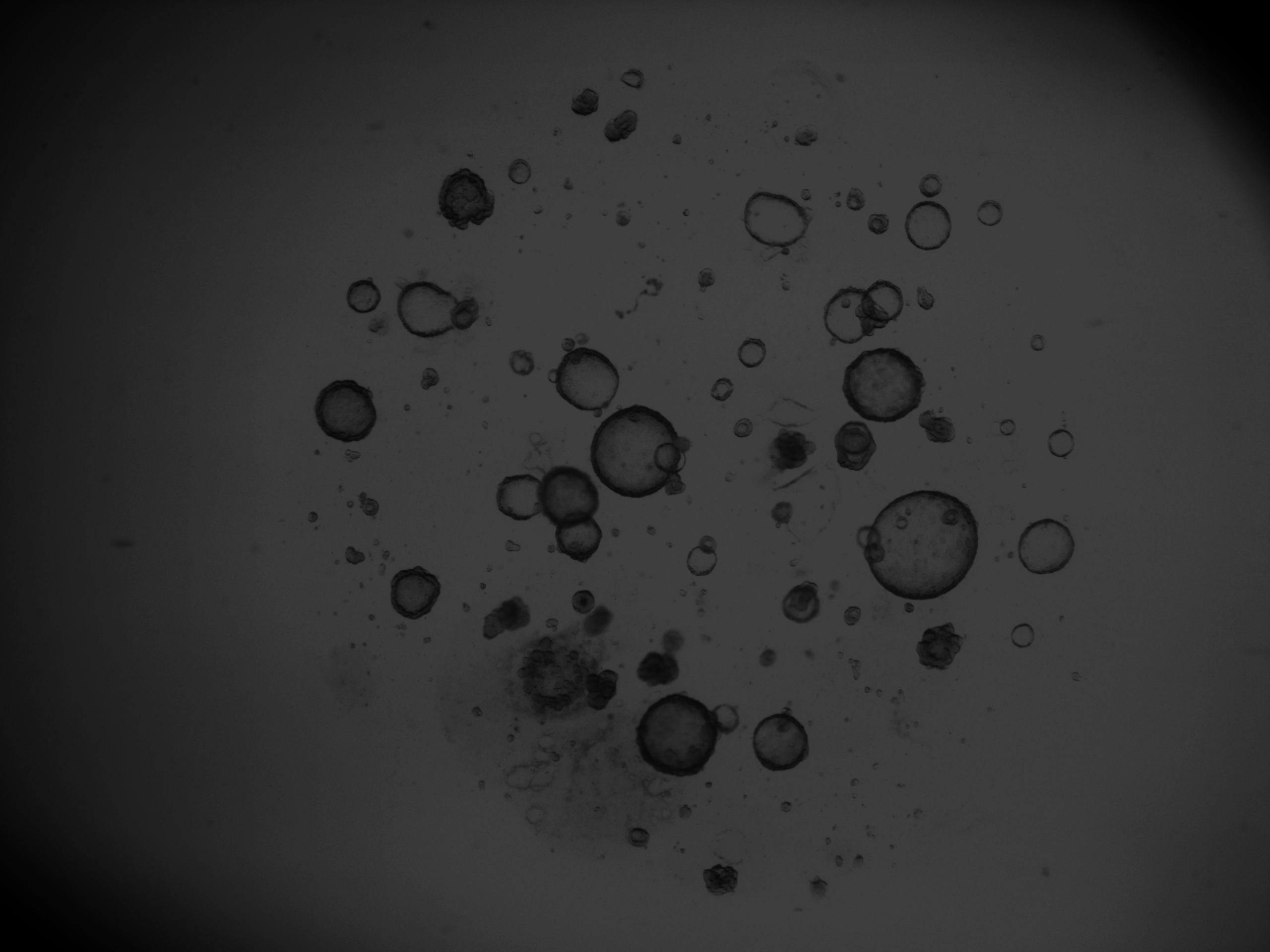

Supplement: Supplementary file 10 — Source data Fig. 5 [file 44319_2024_335_MOESM10_ESM.zip › Figure 5/5E/210426_FOXA1_mutants_exp_+DHT_+ATRA_C57#1_mix_p26_ENRA--_t16_FOXA1_D226_N226_t8_+DHT_d6.tif]

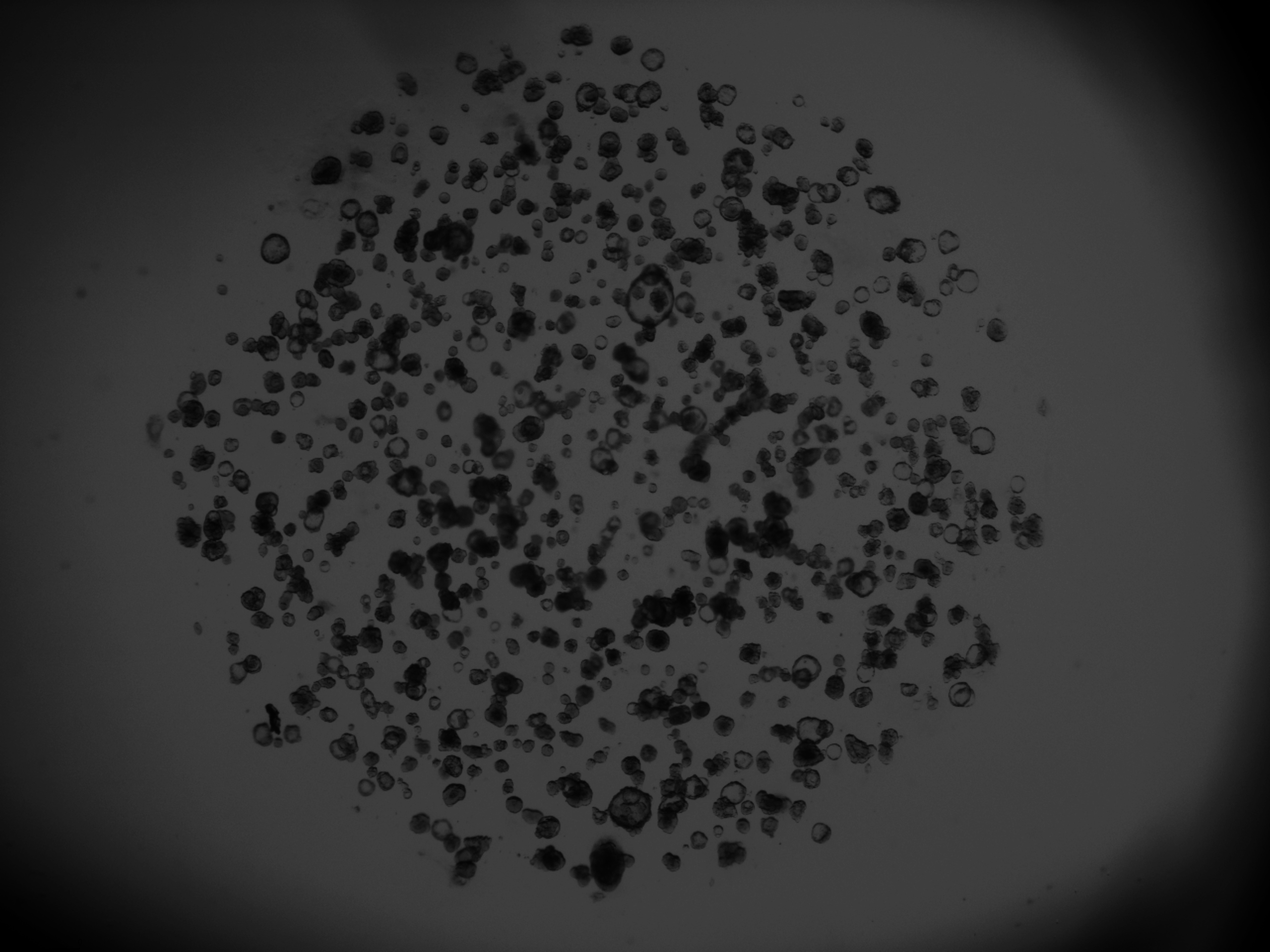

Supplement: Supplementary file 10 — Source data Fig. 5 [file 44319_2024_335_MOESM10_ESM.zip › Figure 5/5E/210426_FOXA1_mutants_exp_+DHT_+ATRA_C57#1_mix_p26_ENRA--_t16_FOXA1_D226_N226_t8_d6.tif]

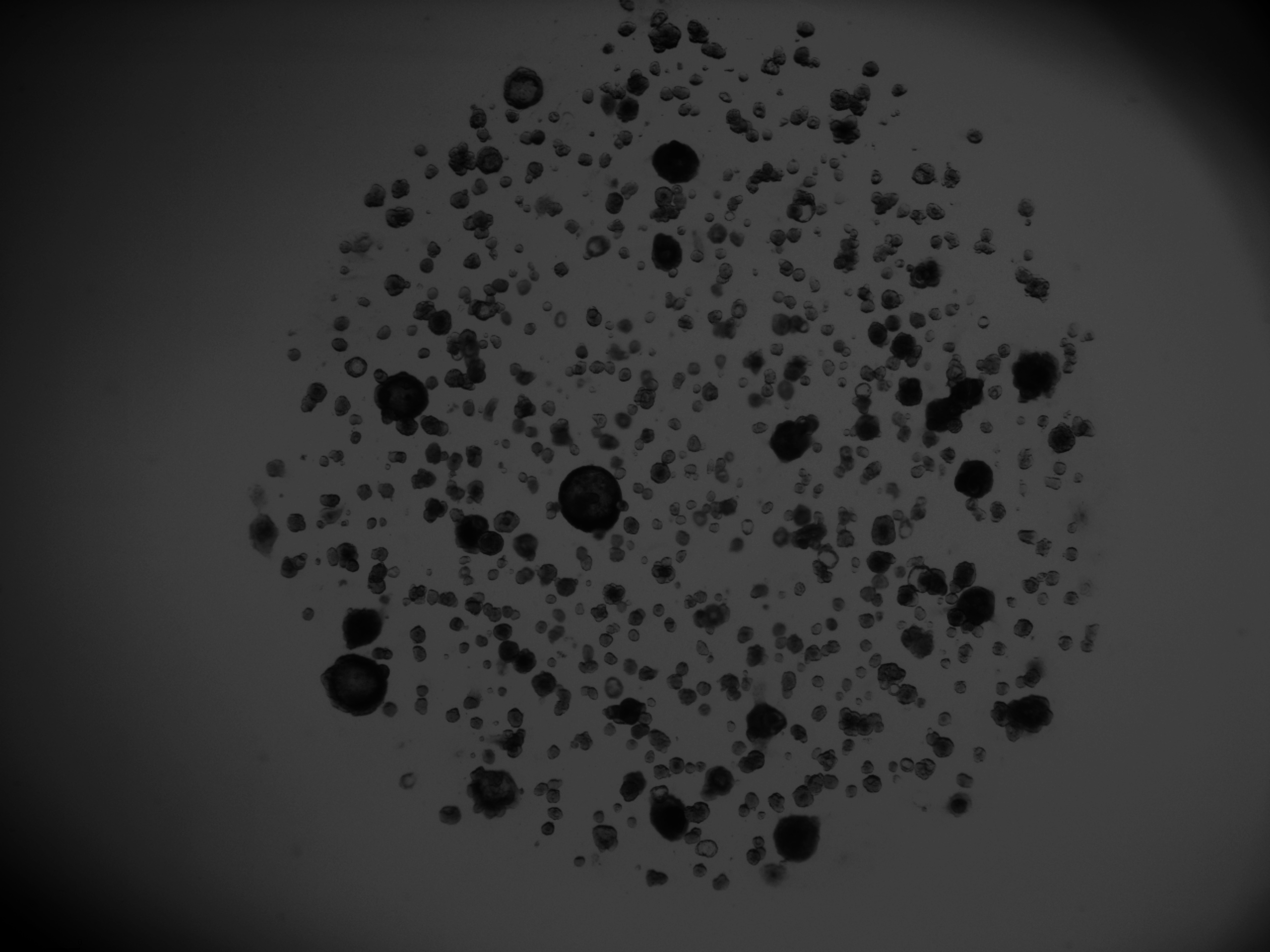

Supplement: Supplementary file 10 — Source data Fig. 5 [file 44319_2024_335_MOESM10_ESM.zip › Figure 5/5E/210426_FOXA1_mutants_exp_+DHT_+ATRA_C57#1_mix_p26_ENRA--_t16_FOXA1_H247_Q247_t8_d6.tif]

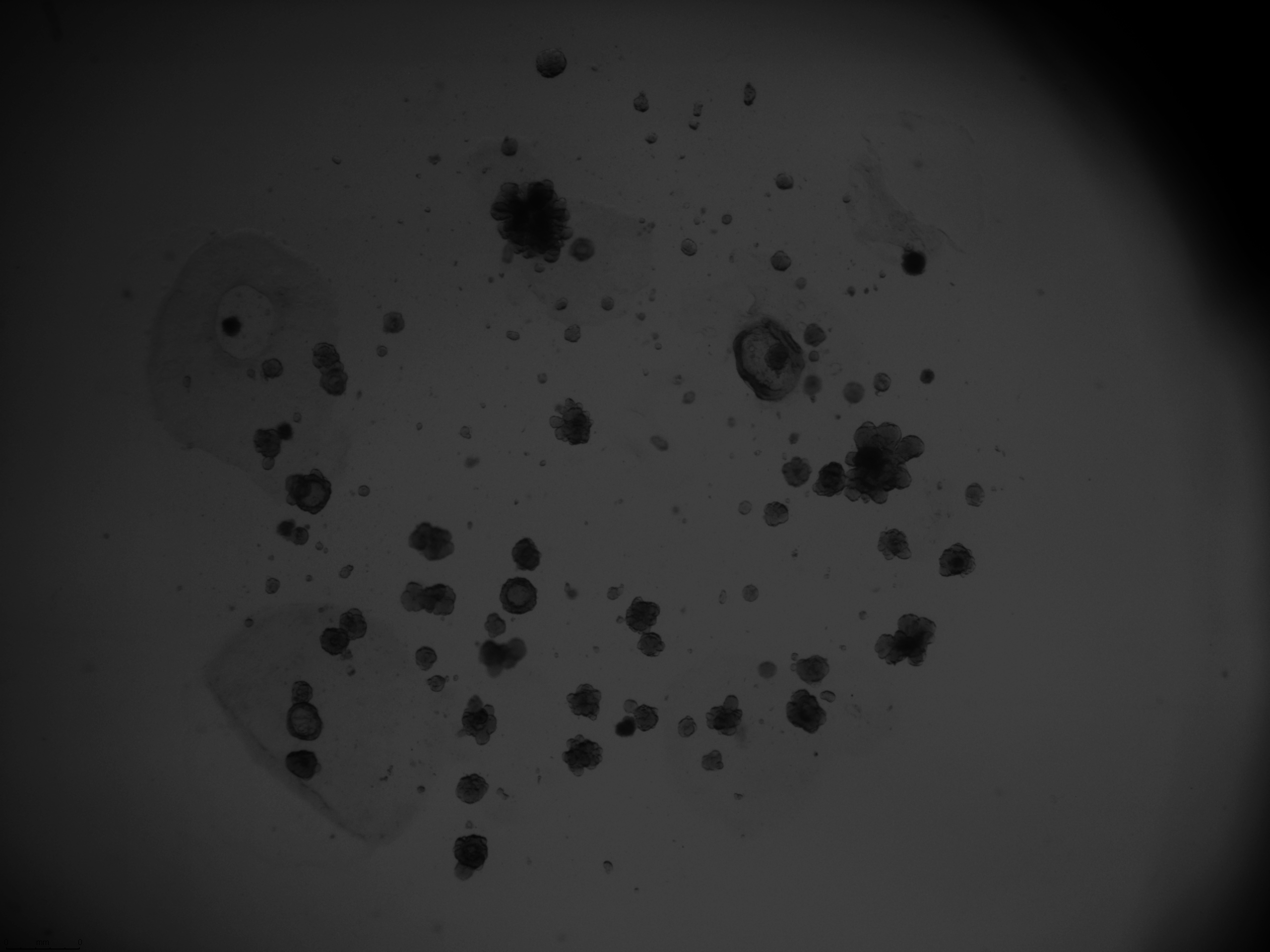

Supplement: Supplementary file 10 — Source data Fig. 5 [file 44319_2024_335_MOESM10_ESM.zip › Figure 5/5E/210426_FOXA1_mutants_exp_+DHT_+ATRA_C57#1_mix_p26_ENRA--_t16_FOXA1_DF254_255_t8_+DHT_d6.tif]

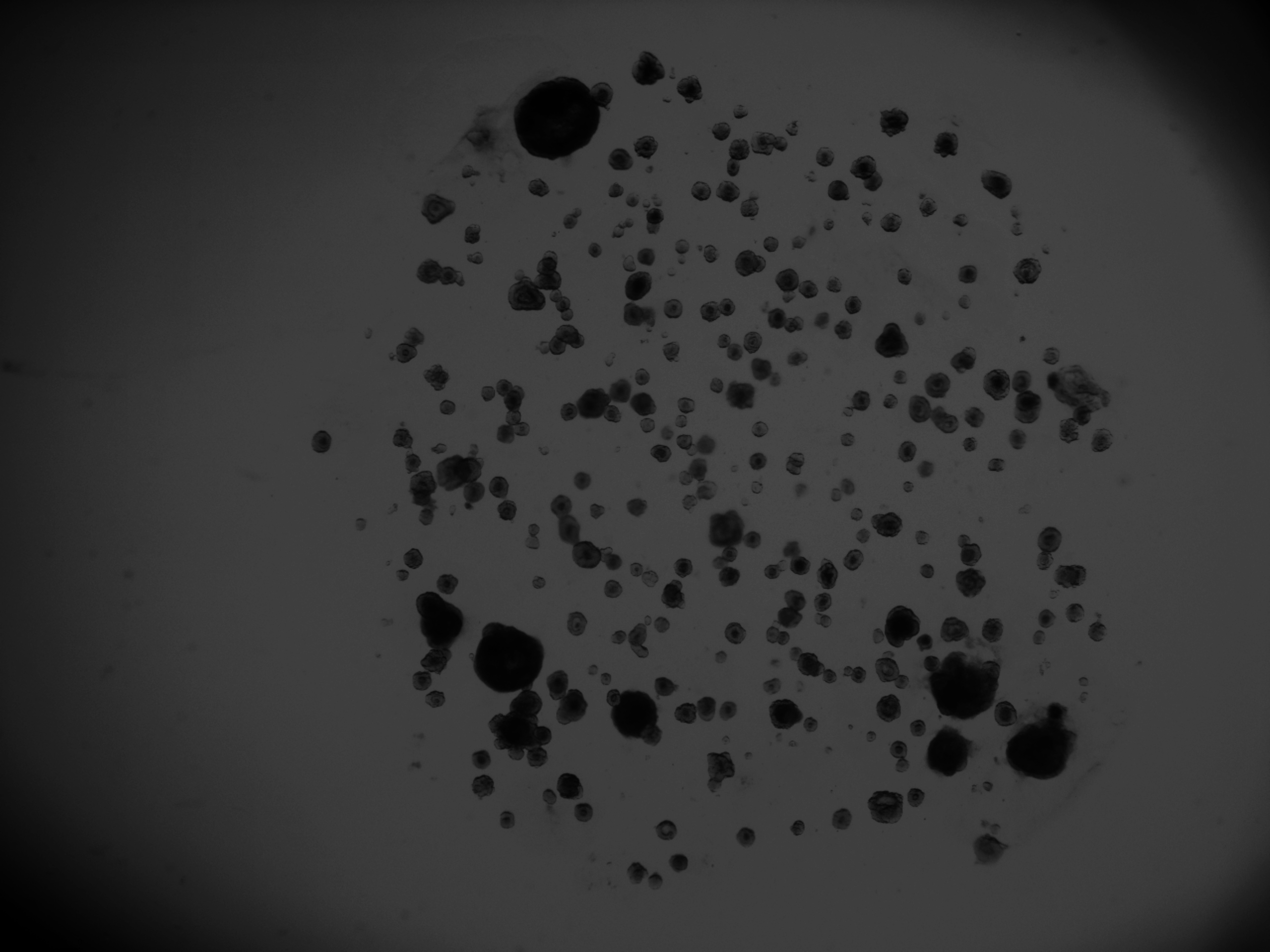

Supplement: Supplementary file 10 — Source data Fig. 5 [file 44319_2024_335_MOESM10_ESM.zip › Figure 5/5E/210426_FOXA1_mutants_exp_+DHT_+ATRA_C57#1_mix_p26_ENRA--_t16_FOXA1_DF254_255_t8_d6.tif]

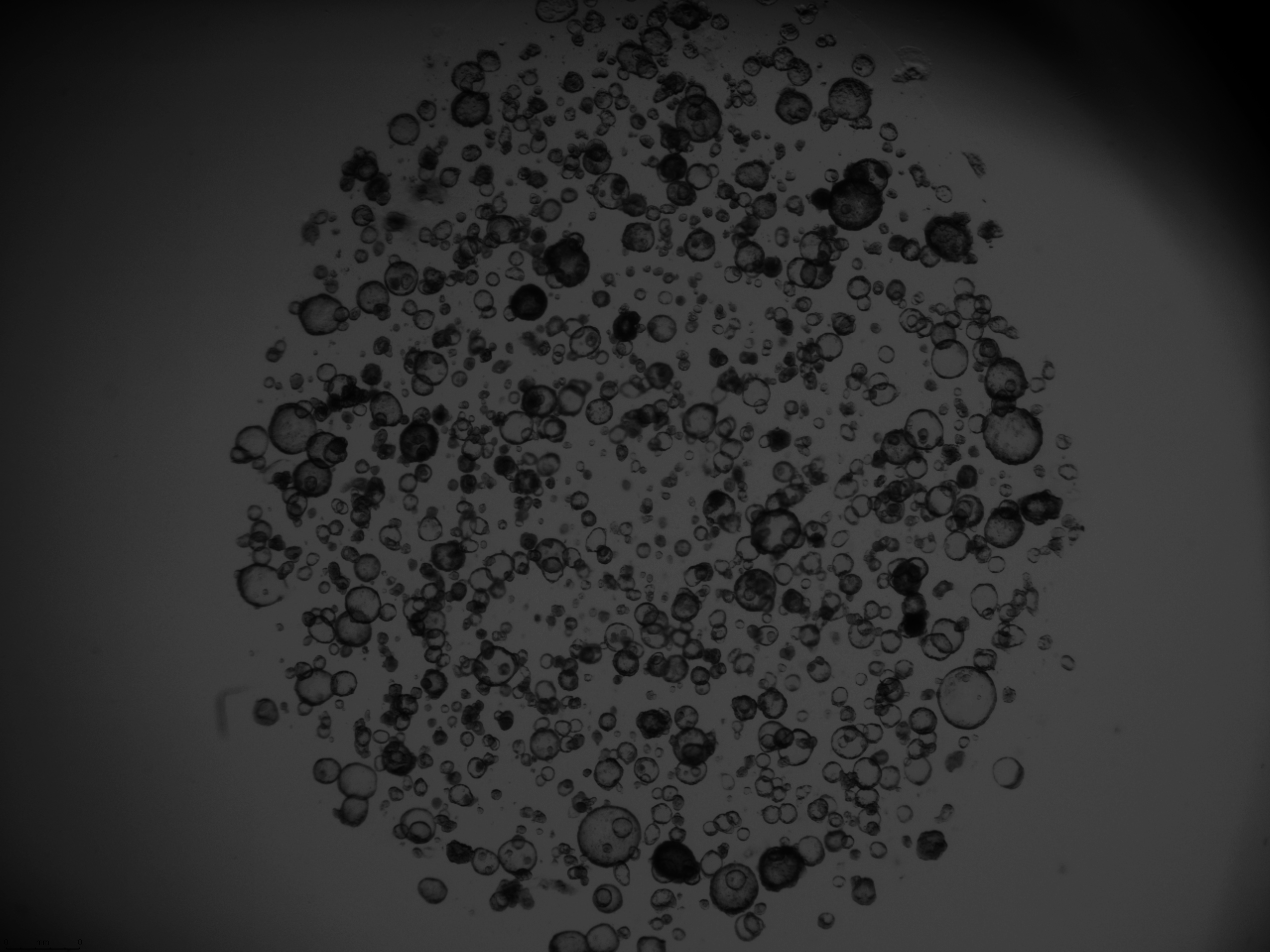

Supplement: Supplementary file 10 — Source data Fig. 5 [file 44319_2024_335_MOESM10_ESM.zip › Figure 5/5E/210426_FOXA1_mutants_exp_+DHT_+ATRA_C57#1_mix_p26_ENRA--_t16_FOXA1_WT_t8_d6.tif]

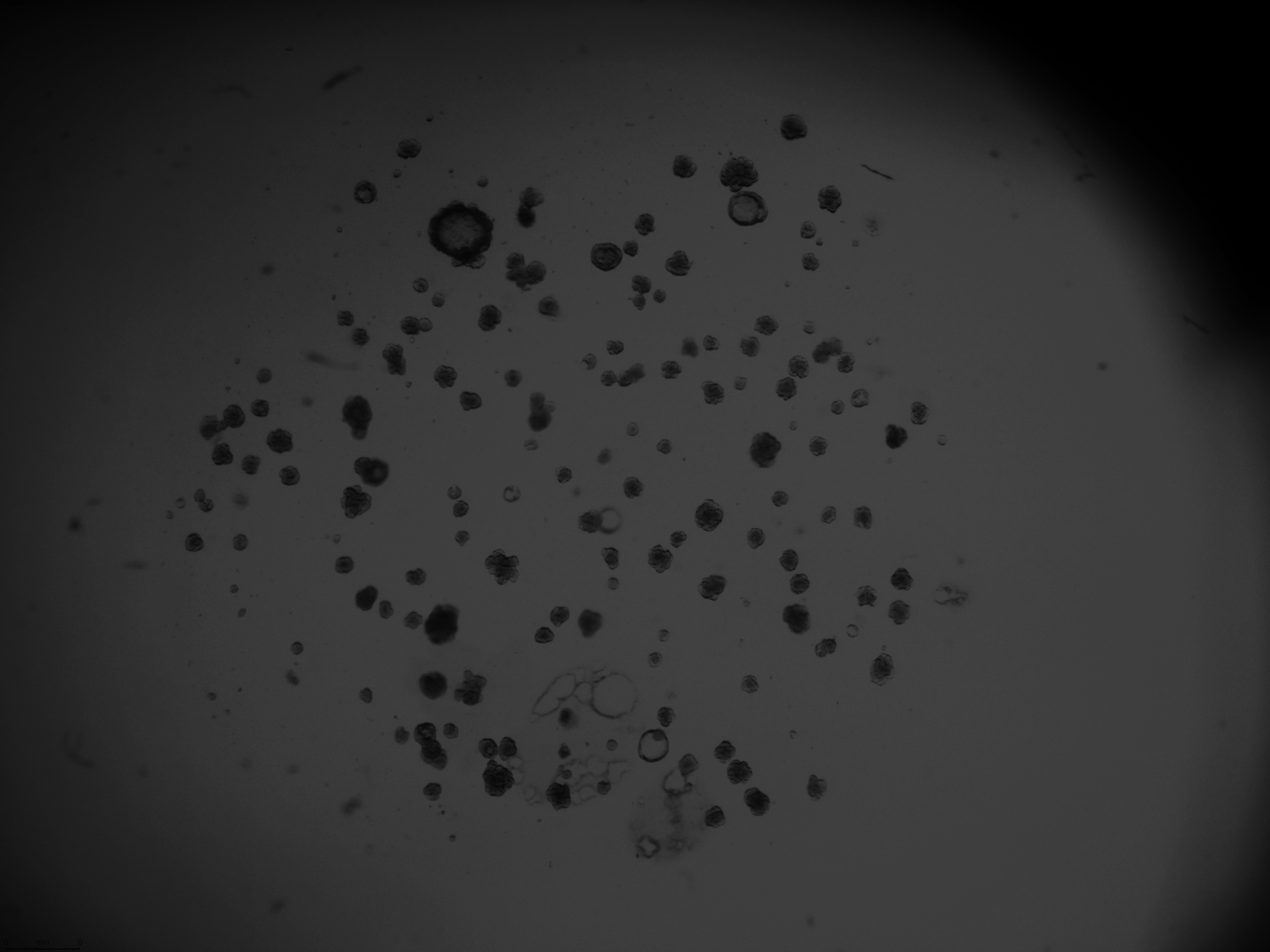

Supplement: Supplementary file 10 — Source data Fig. 5 [file 44319_2024_335_MOESM10_ESM.zip › Figure 5/5E/210426_FOXA1_mutants_exp_+DHT_+ATRA_C57#1_mix_p26_ENRA--_t16_EMPTYvec_t8_+DHT_d6.tif]

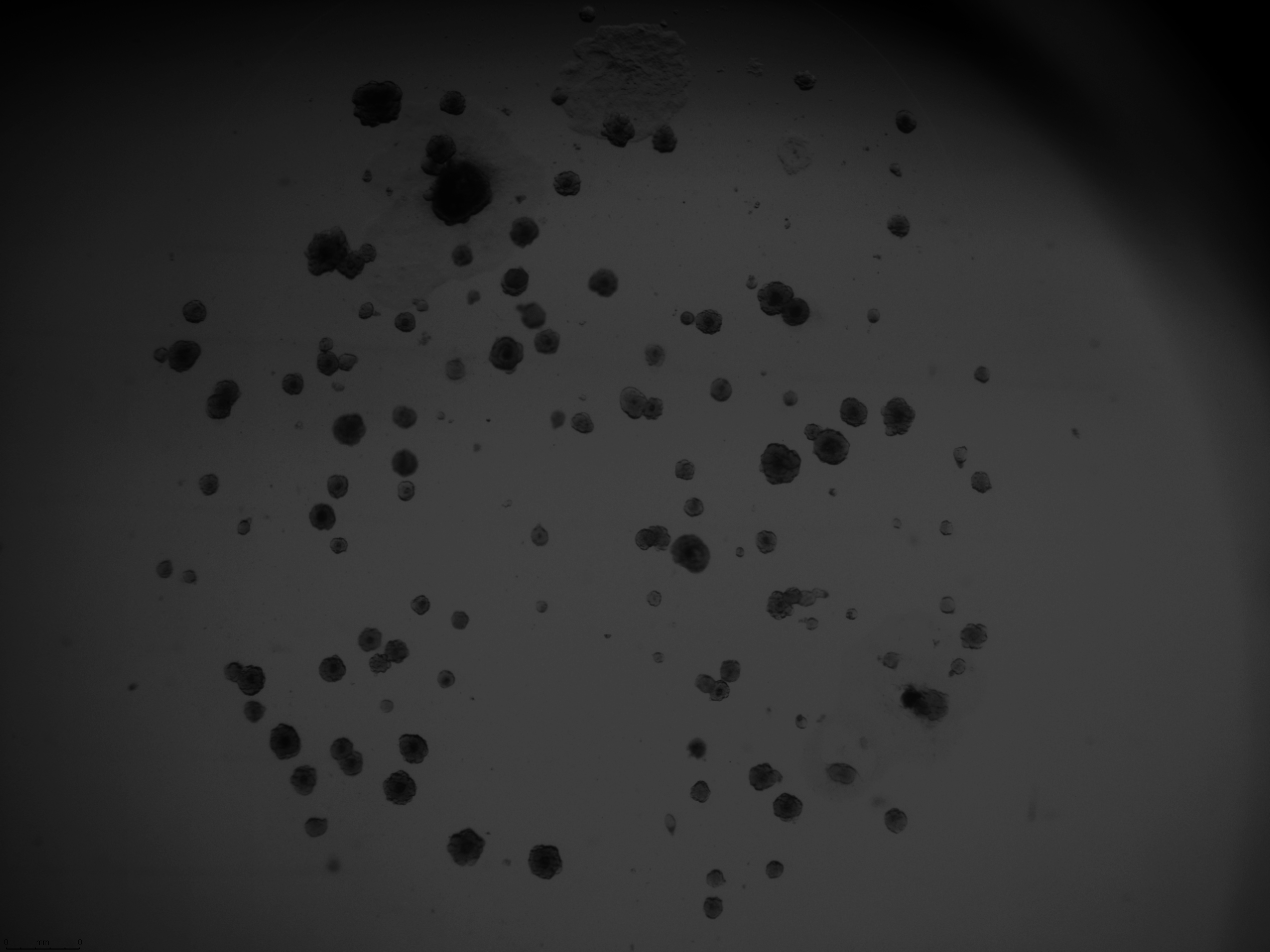

Supplement: Supplementary file 10 — Source data Fig. 5 [file 44319_2024_335_MOESM10_ESM.zip › Figure 5/5E/210426_FOXA1_mutants_exp_+DHT_+ATRA_C57#1_mix_p26_ENRA--_t16_EMPTYvec_t8_d6.tif]

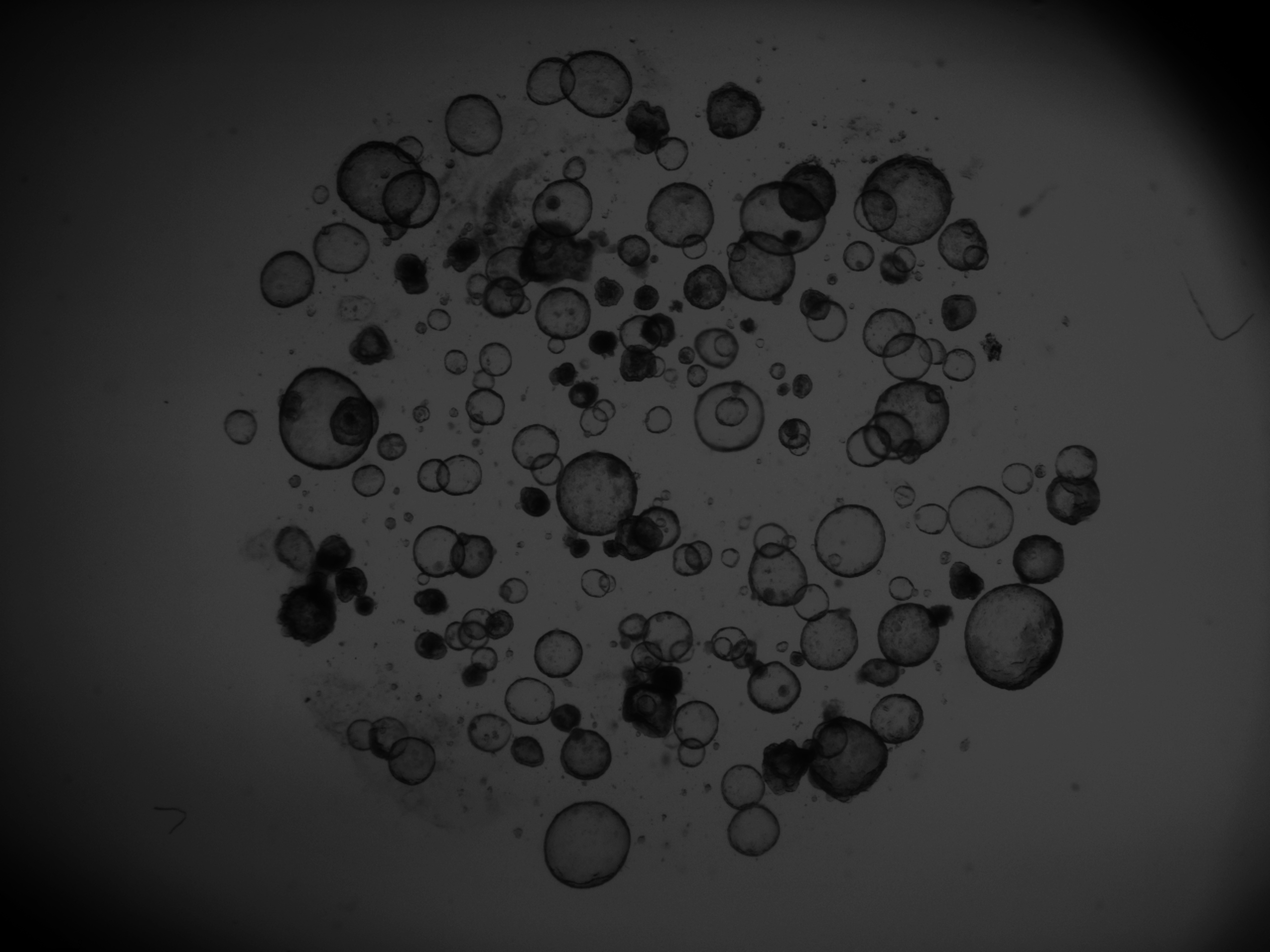

Supplement: Supplementary file 10 — Source data Fig. 5 [file 44319_2024_335_MOESM10_ESM.zip › Figure 5/5E/210426_FOXA1_mutants_exp_+DHT_+ATRA_C57#1_mix_p26_ENRA--_t16_FOXA1_WT_t8_+DHT_d6.tif]

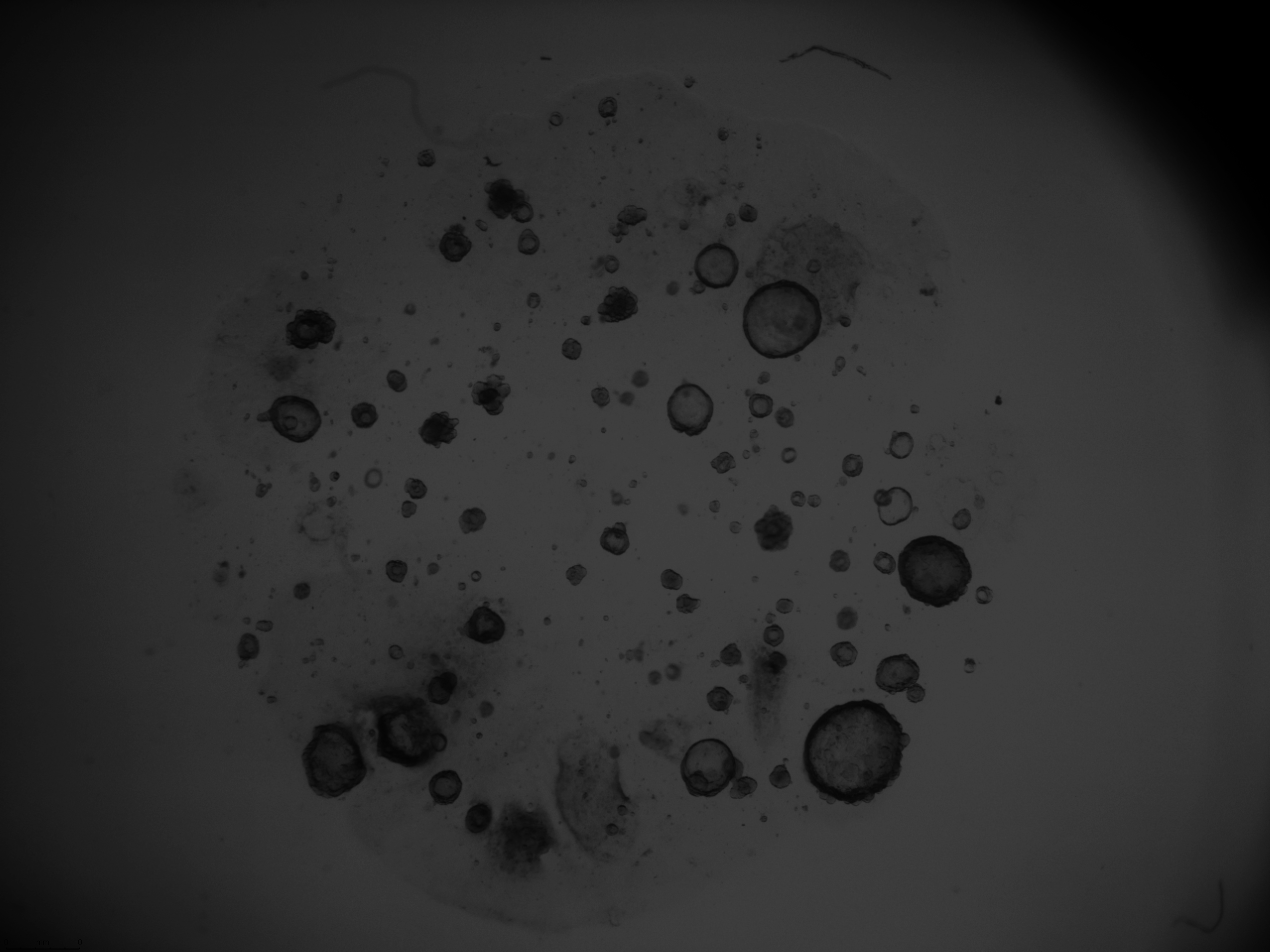

Supplement: Supplementary file 10 — Source data Fig. 5 [file 44319_2024_335_MOESM10_ESM.zip › Figure 5/5E/210426_FOXA1_mutants_exp_+DHT_+ATRA_C57#1_mix_p26_ENRA--_t16_FOXA1_H247_Q247_t8_+DHT_d6.tif]

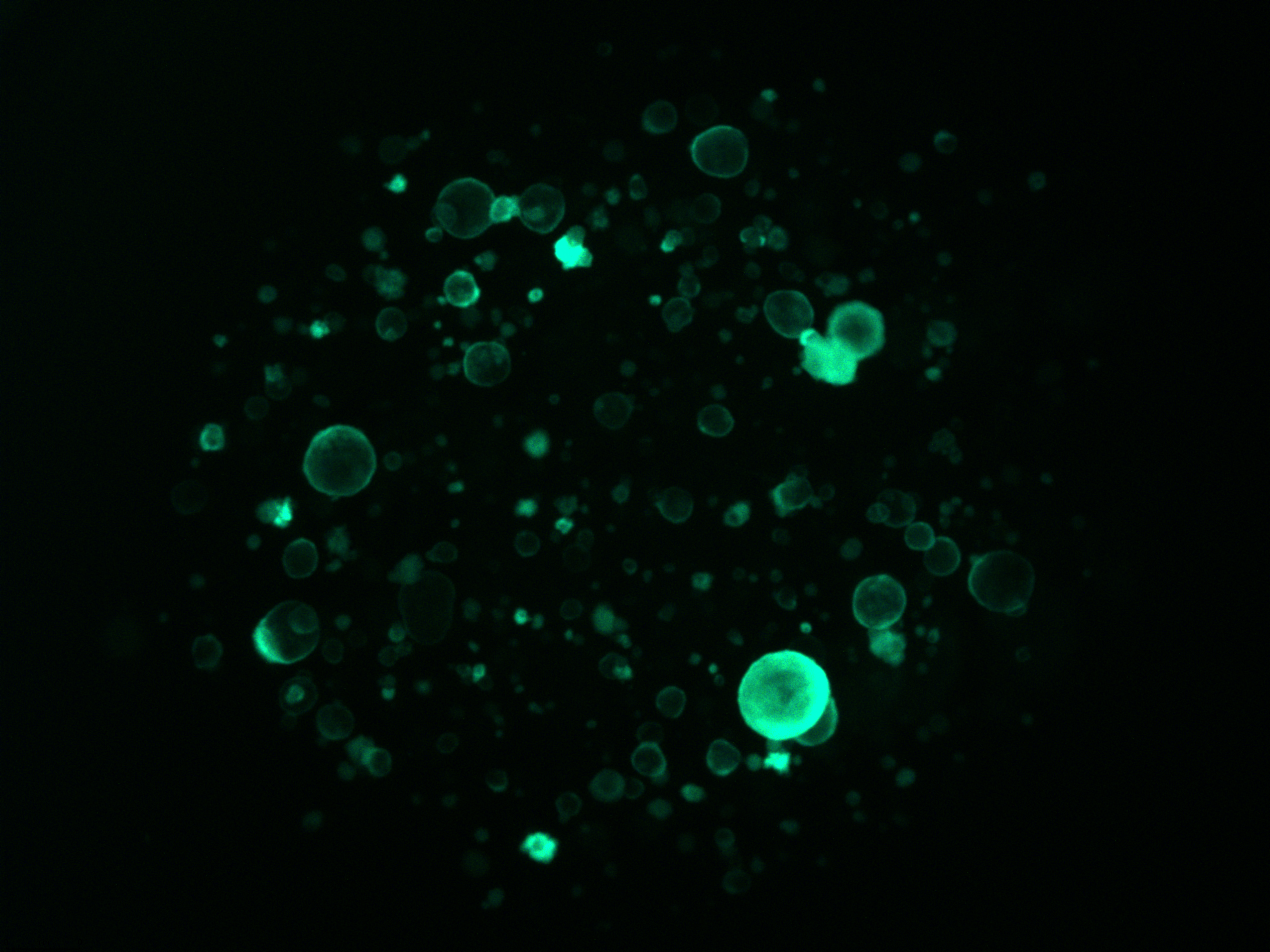

Supplement: Supplementary file 10 — Source data Fig. 5 [file 44319_2024_335_MOESM10_ESM.zip › Figure 5/5B/210323_pMSCV_neo_GFP_Foxa1_mutants_t3_fluorescence_210323_C57#1_mix_p21_ENRA--_t11_FOXA1_wt_vec_d6_1.tif]

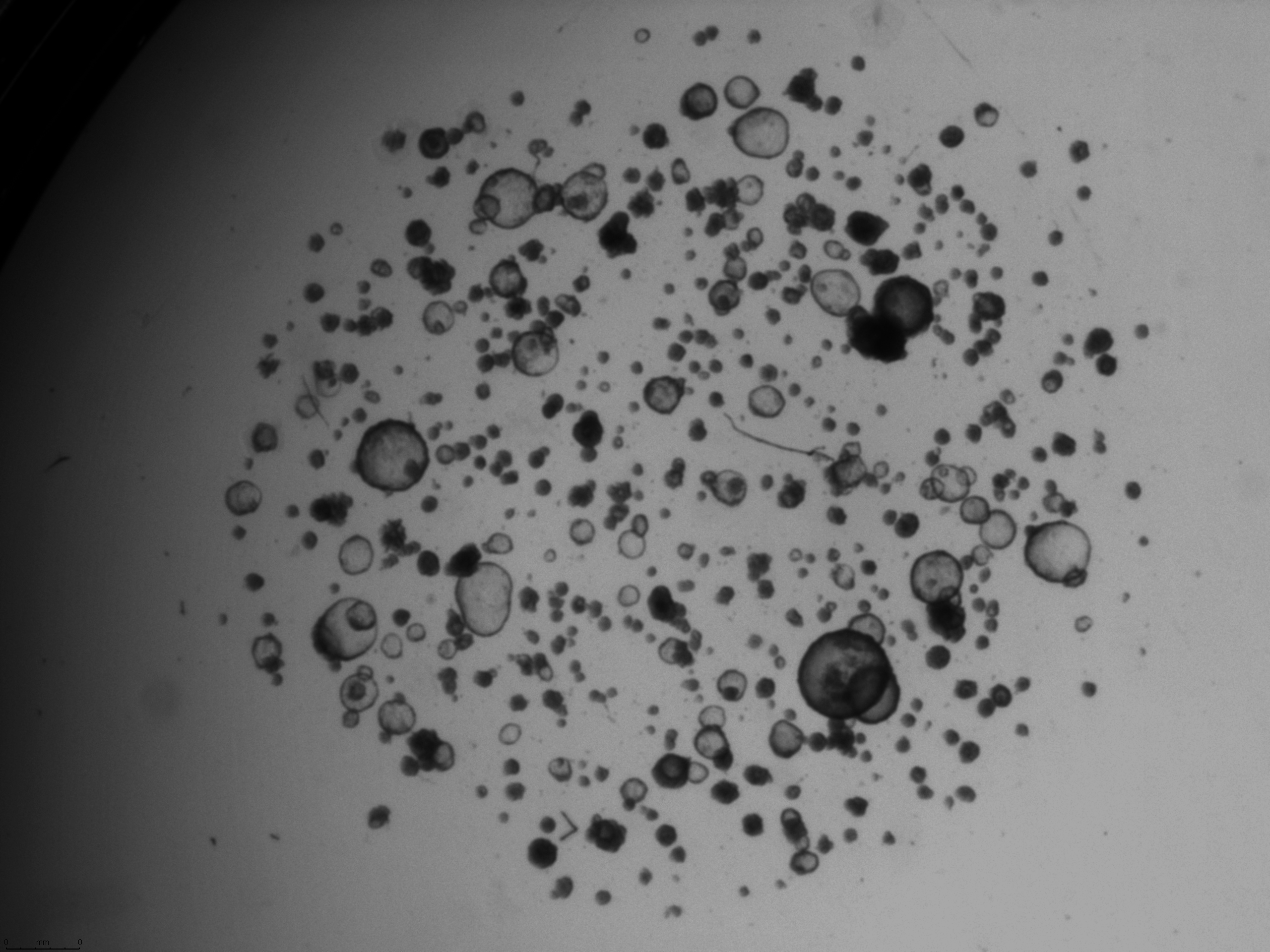

Supplement: Supplementary file 10 — Source data Fig. 5 [file 44319_2024_335_MOESM10_ESM.zip › Figure 5/5B/210323_pMSCV_neo_GFP_Foxa1_mutants_t3_brightfield_210323_C57#1_mix_p21_ENRA--_t11_FOXA1_wt_vec_d6_1.tif]

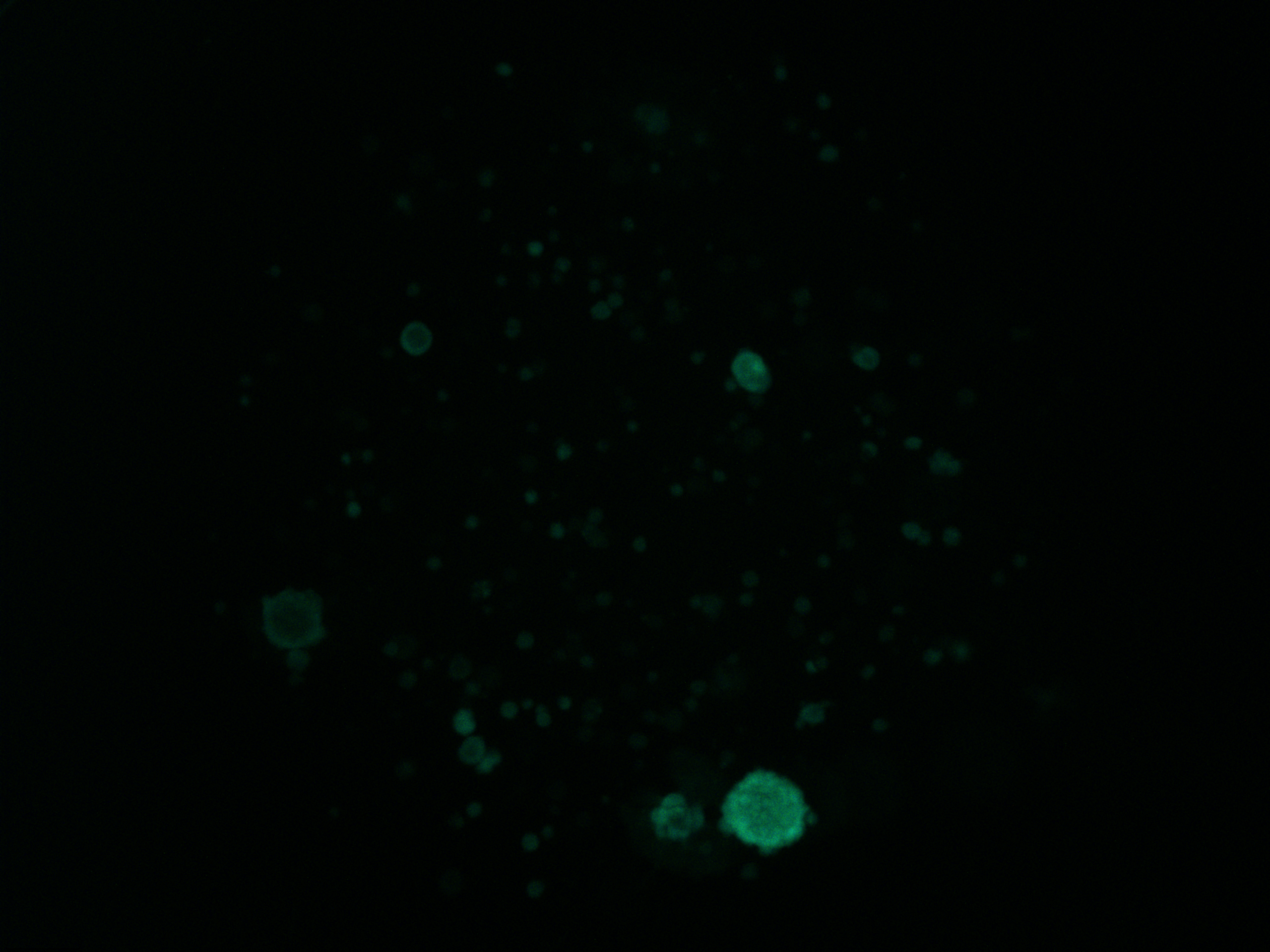

Supplement: Supplementary file 10 — Source data Fig. 5 [file 44319_2024_335_MOESM10_ESM.zip › Figure 5/5B/210323_pMSCV_neo_GFP_Foxa1_mutants_t3_fluorescence_210323_C57#1_mix_p21_ENRA--_t11_D226_N226_d6_1.tif]

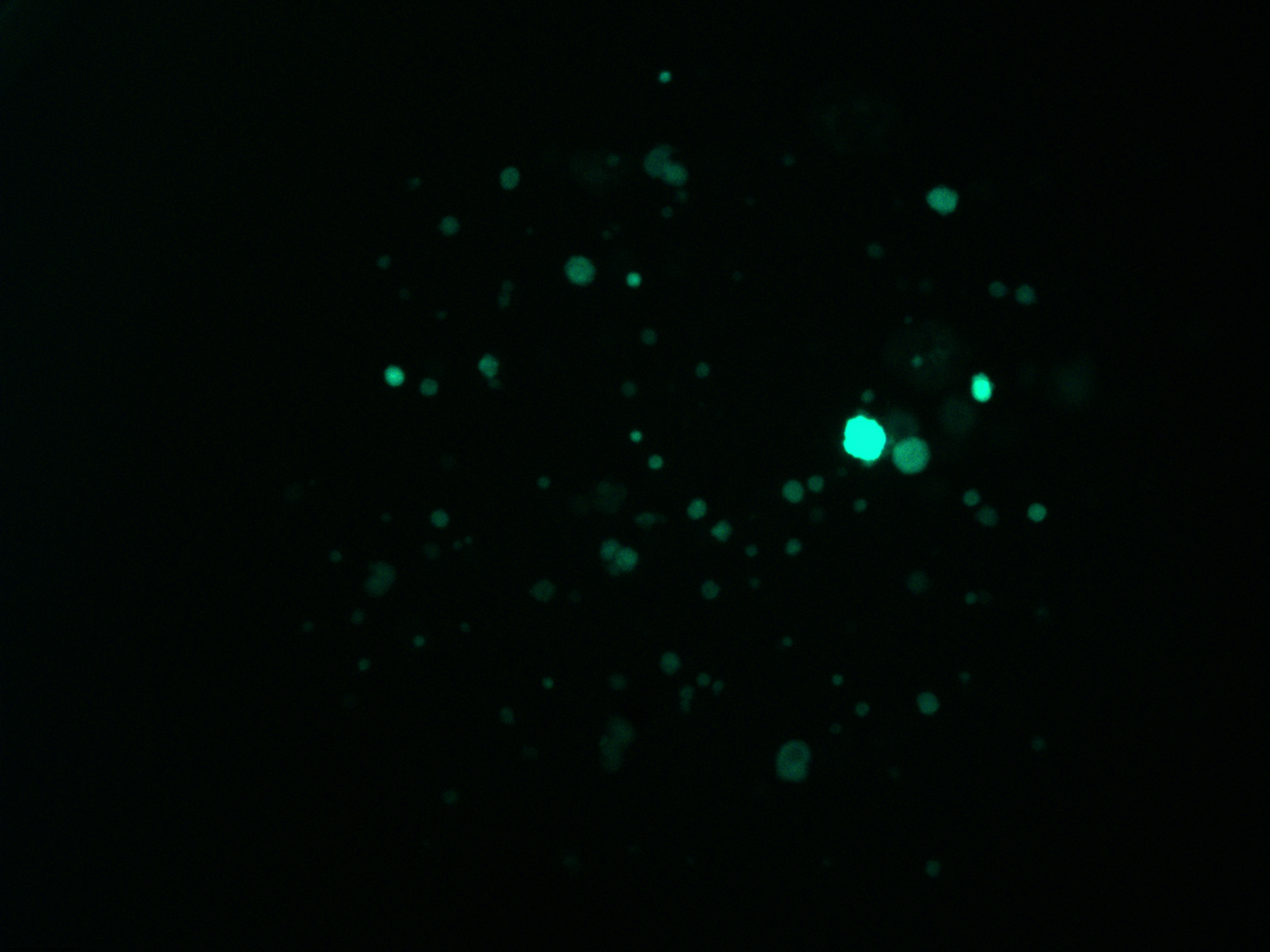

Supplement: Supplementary file 10 — Source data Fig. 5 [file 44319_2024_335_MOESM10_ESM.zip › Figure 5/5B/210323_pMSCV_neo_GFP_Foxa1_mutants_t3_fluorescence_210323_C57#1_mix_p21_ENRA--_t11_EMPTY_vec_d6_1.tif]

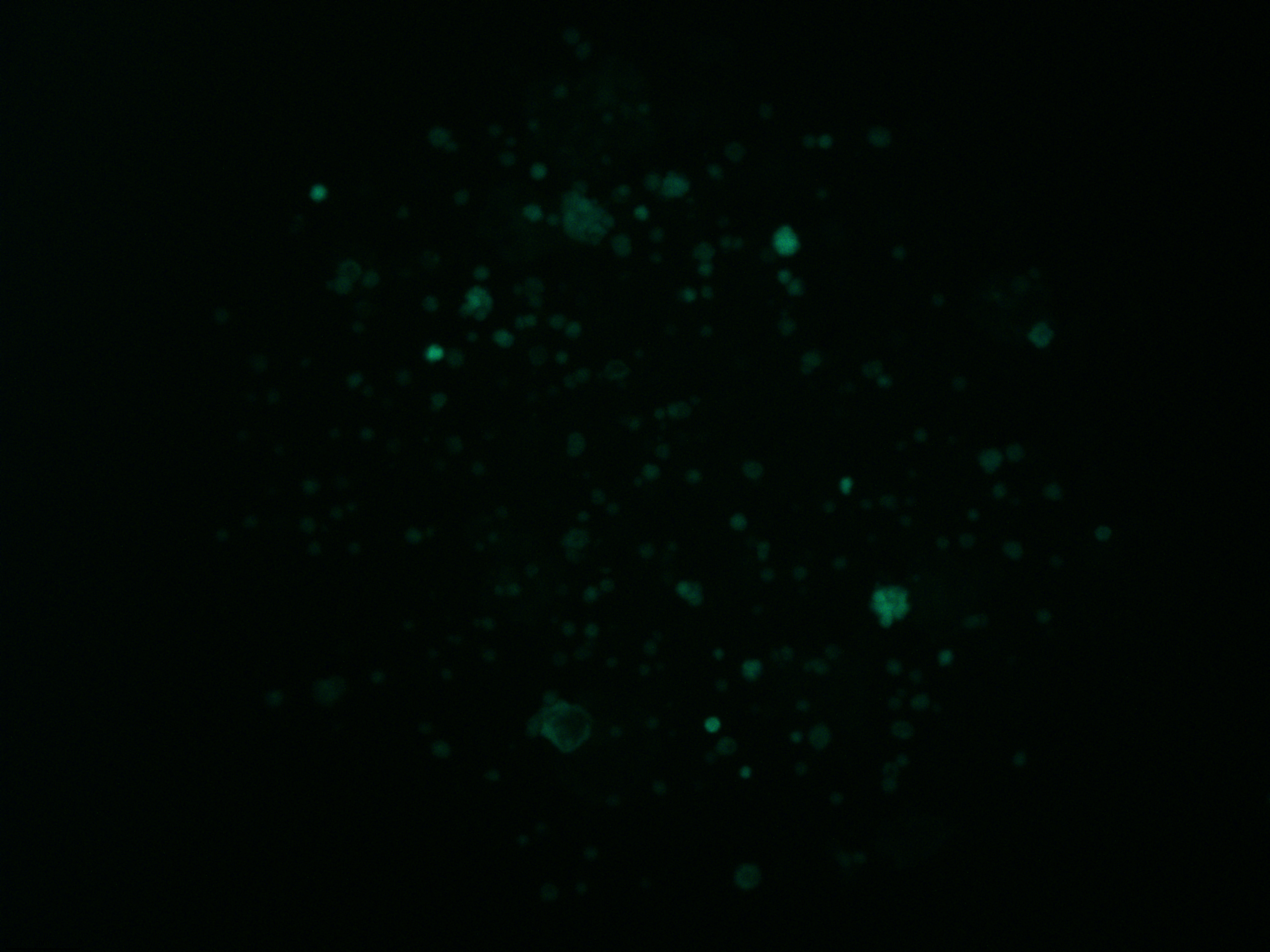

Supplement: Supplementary file 10 — Source data Fig. 5 [file 44319_2024_335_MOESM10_ESM.zip › Figure 5/5B/210323_pMSCV_neo_GFP_Foxa1_mutants_t3_fluorescence_210323_C57#1_mix_p21_ENRA--_t11_H247_Q247_d6_1.tif]

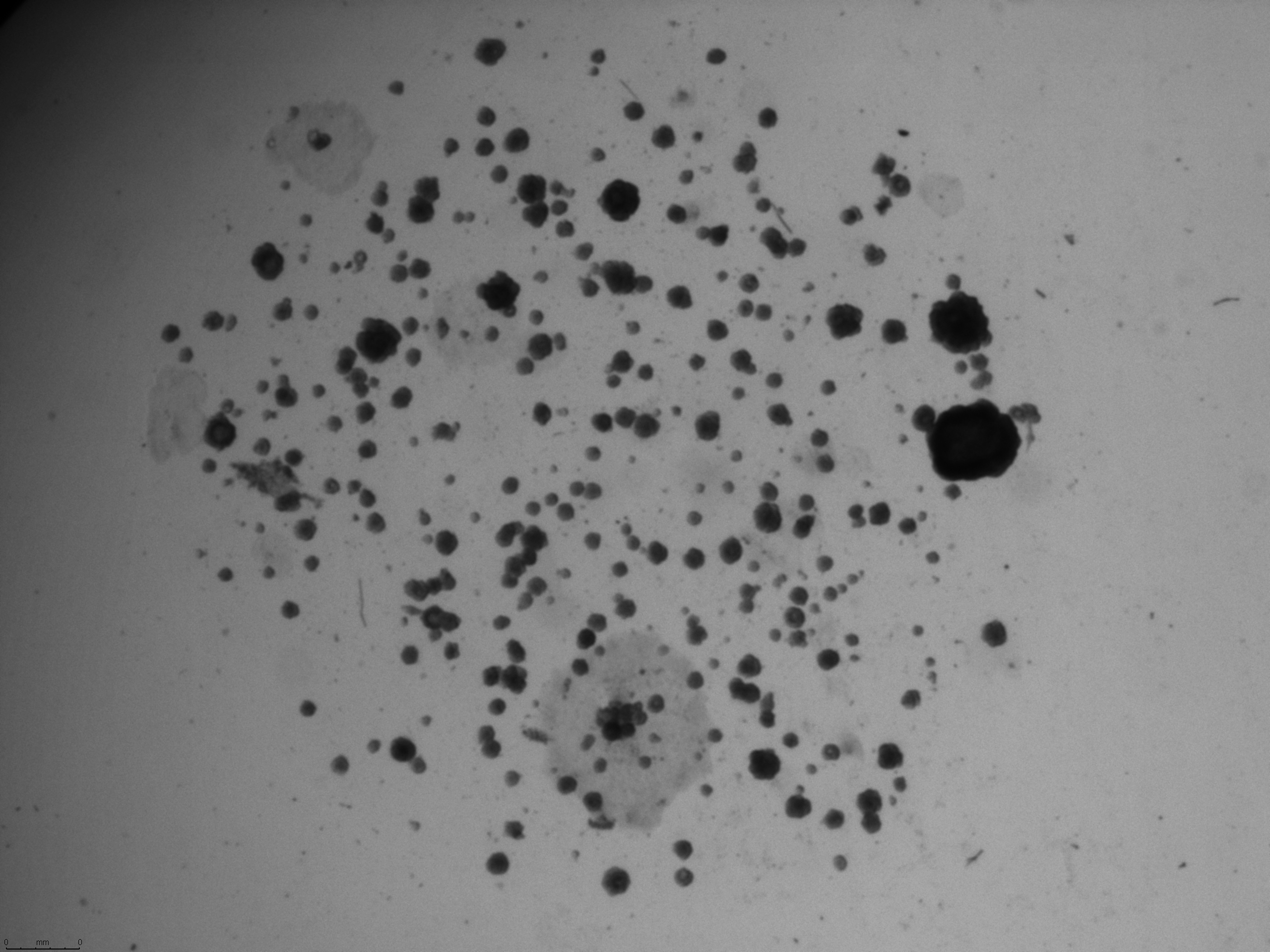

Supplement: Supplementary file 10 — Source data Fig. 5 [file 44319_2024_335_MOESM10_ESM.zip › Figure 5/5B/210323_pMSCV_neo_GFP_Foxa1_mutants_t3_brightfield_210323_C57#1_mix_p21_ENRA--_t11_Delta_F254_255_d6_1.tif]

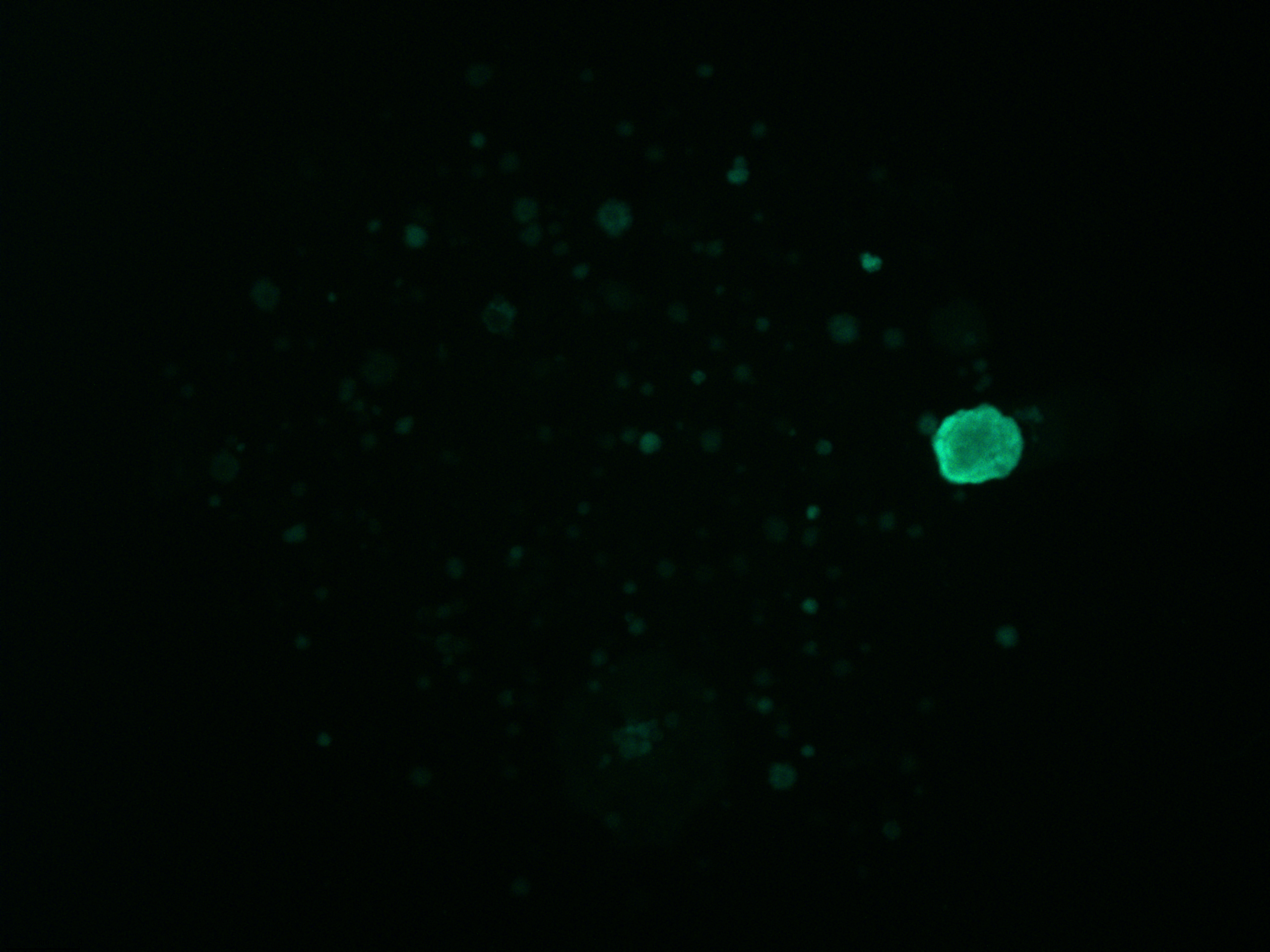

Supplement: Supplementary file 10 — Source data Fig. 5 [file 44319_2024_335_MOESM10_ESM.zip › Figure 5/5B/210323_pMSCV_neo_GFP_Foxa1_mutants_t3_fluorescence_210323_C57#1_mix_p21_ENRA--_t11_Delta_F254_255_d6_1.tif]

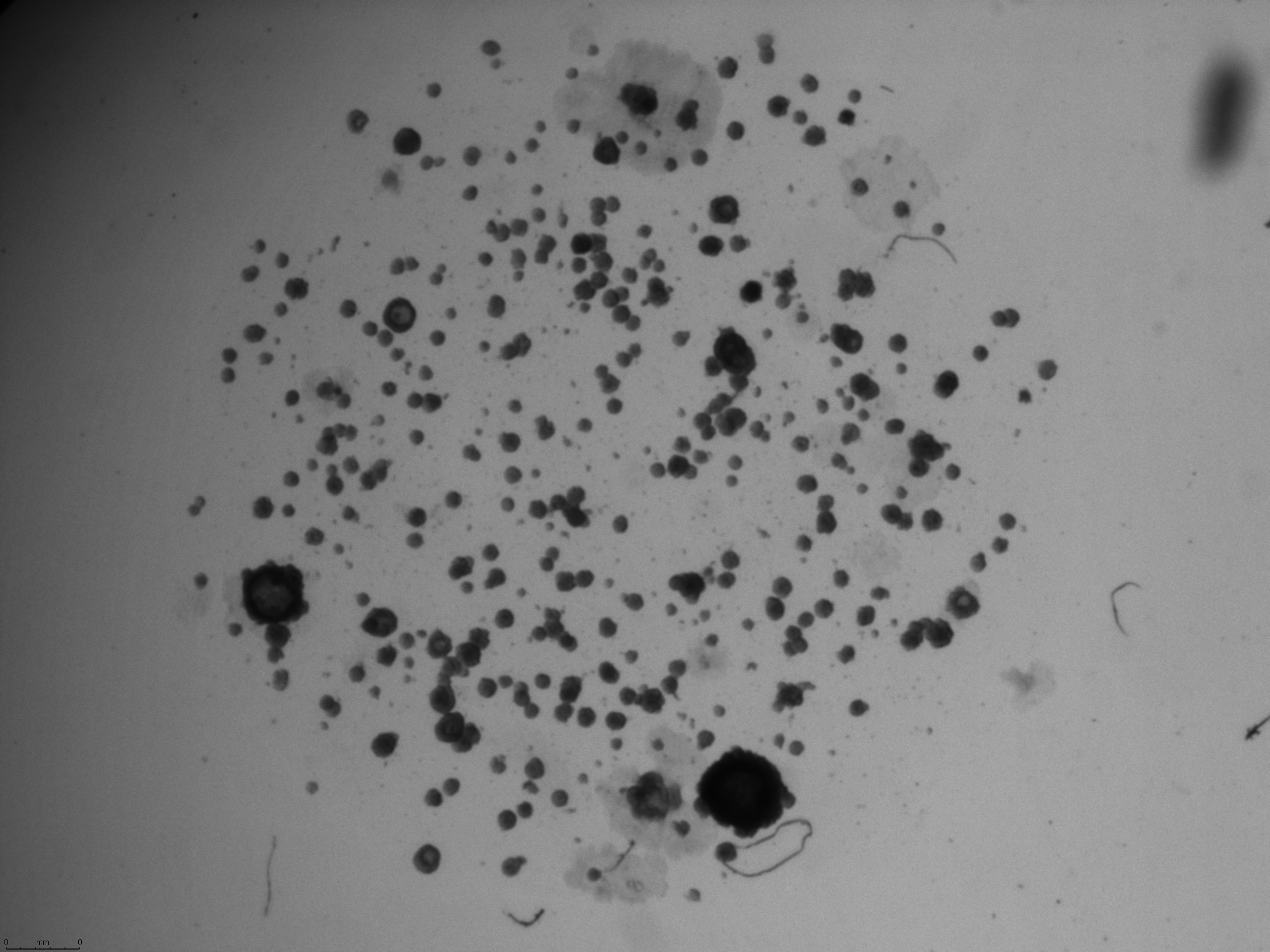

Supplement: Supplementary file 10 — Source data Fig. 5 [file 44319_2024_335_MOESM10_ESM.zip › Figure 5/5B/210323_pMSCV_neo_GFP_Foxa1_mutants_t3_brightfield_210323_C57#1_mix_p21_ENRA--_t11_D226_N226_d6_1.tif]

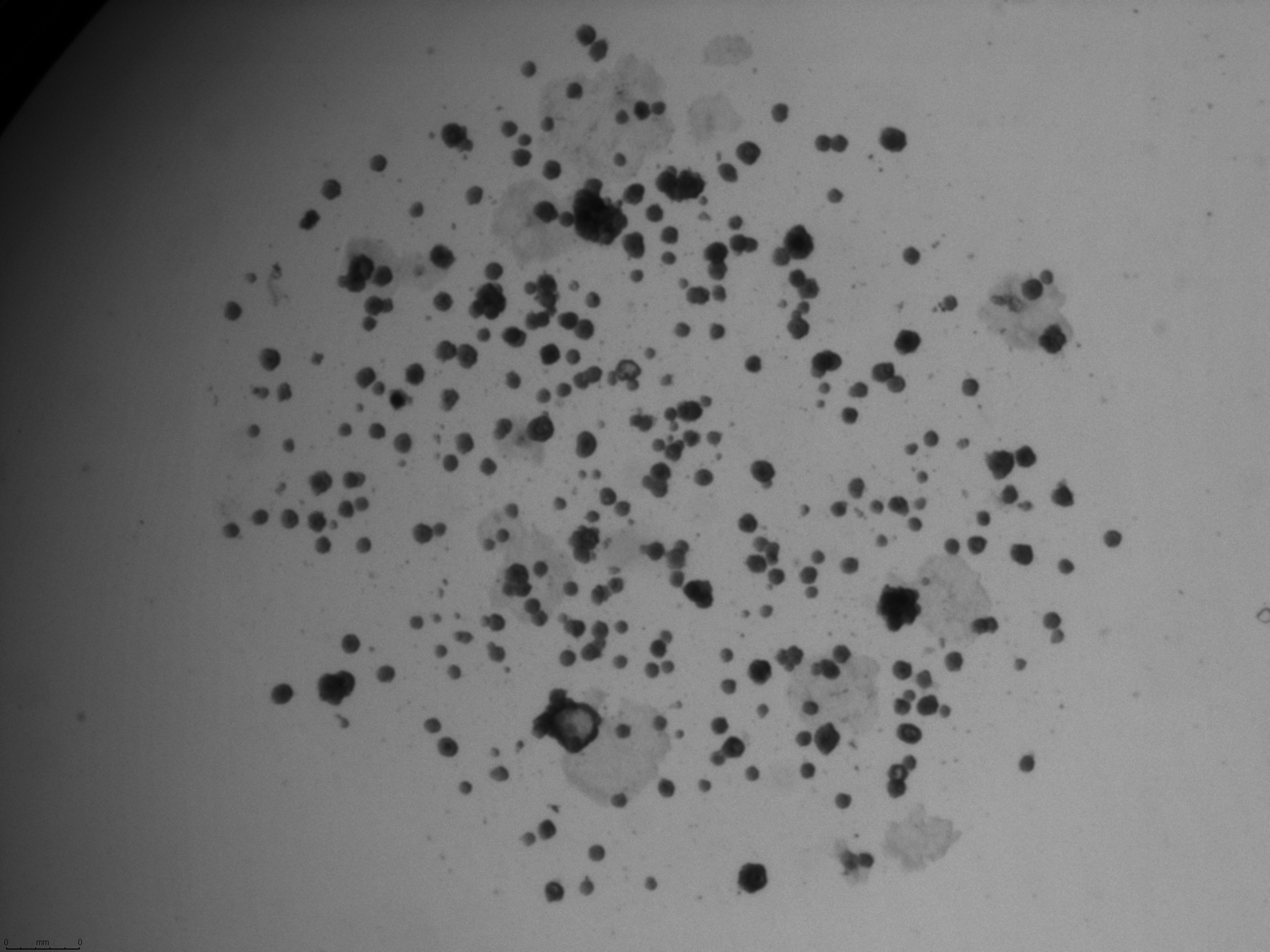

Supplement: Supplementary file 10 — Source data Fig. 5 [file 44319_2024_335_MOESM10_ESM.zip › Figure 5/5B/210323_pMSCV_neo_GFP_Foxa1_mutants_t3_brightfield_210323_C57#1_mix_p21_ENRA--_t11_H247_Q247_d6_1.tif]

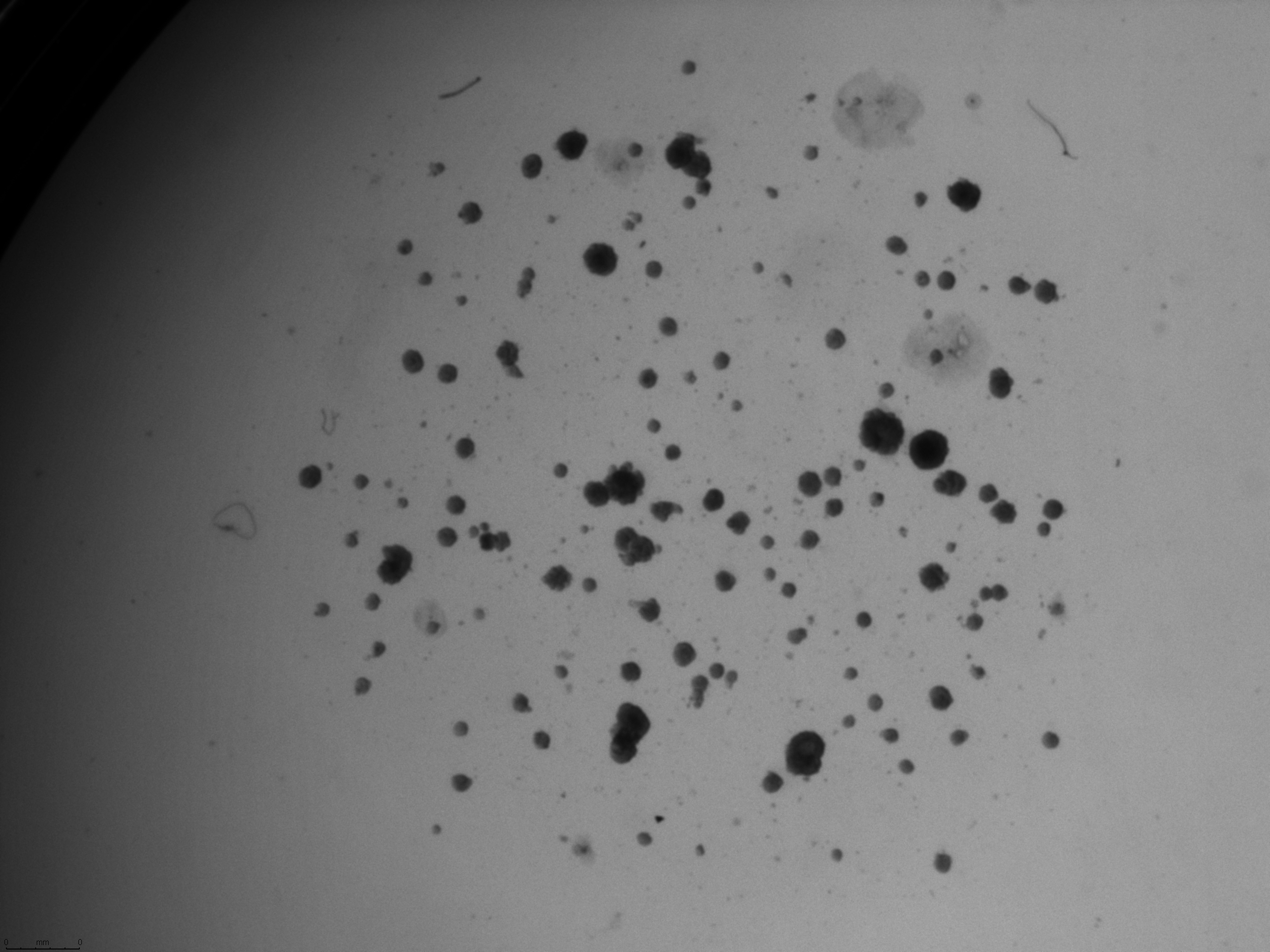

Supplement: Supplementary file 10 — Source data Fig. 5 [file 44319_2024_335_MOESM10_ESM.zip › Figure 5/5B/210323_pMSCV_neo_GFP_Foxa1_mutants_t3_brightfield_210323_C57#1_mix_p21_ENRA--_t11_EMPTY_vec_d6_1.tif]
