# Supplementary material for: Rarγ-Foxa1 signaling promotes luminal identity in prostate progenitors and is disrupted in prostate cancer
Source: EMBO Rep. 2024 Dec 4;26(2):443–69. doi: 10.1038/s44319-024-00335-y (PMC11772605; doi:10.1038/s44319-024-00335-y)
Supplement: Supplementary file 11 — Expanded View Figures [file 44319_2024_335_MOESM11_ESM.pdf]

Expanded View Figures

Figure EV1. Induction of RA signaling-responsive genes and lumen formation by ATRA and DHT treatment in prostate organoids.

(**A**) Schematic representation of the three main enzymatic steps of retinoid metabolism. (**B**) RNA-Seq analysis showing differentially expressed genes involved in the retinoid pathway upon single or combined administration of ATRA and DHT to mPrOs cultured in ENRA-- medium. Data are presented as mean value ± s.d. of $n = 3$ biological independent replicates. The indicated adjusted *p*-values were calculated with the Wald test followed by the Benjamini–Hochberg multiple test correction (default in DESeq2). (**C**) mPrOs (C57BL6/J-upper panel and CD1-lower panel) morphology after 6 days of administration of different concentration of ATRA. Scale bar, 1 mm. $N > 3$ independent biological replicates. (**D**) Phenotypic analysis of mPrOs cultured with 16 nM ATRA with or without DHT and Enzalutamide. Scale bar, 1 mm. $N = 3$ independent biological replicates.

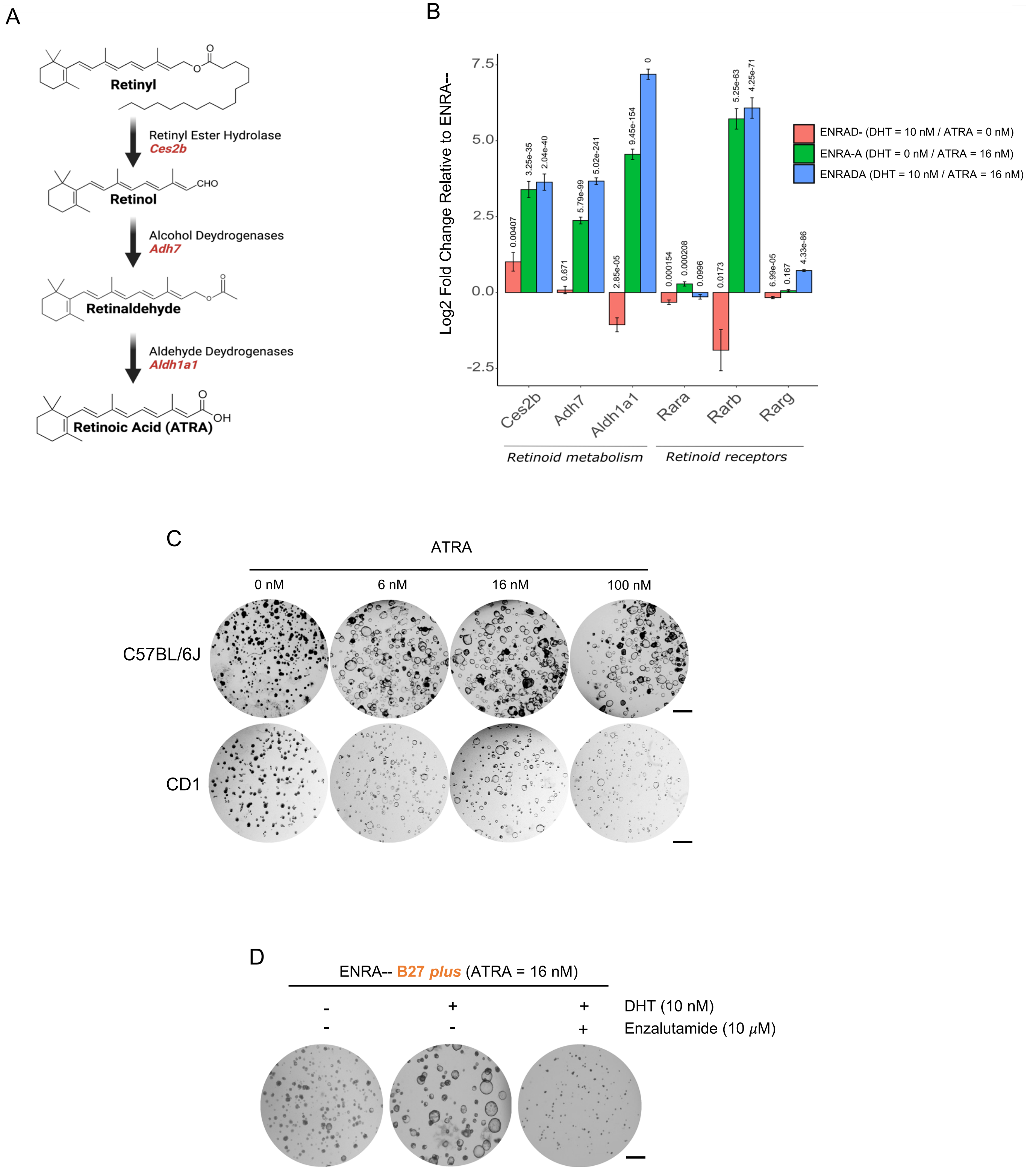
A
Retinyl
Retinyl Ester Hydrolase
Ces2b
CHO
Retinol
Alcohol Deydrogenases
Adh7
Retinaldehyde
Aldehyde Deydrogenases
Aldh1a1
OH
Retinoic Acid (ATRA)
B
Log2 Fold Change Relative to ENRA--
7.5
5.0
2.5
0.0
-2.5
0.00407
3.25e-35
2.04e-40
0.671
5.79e-99
5.02e-241
2.85e-05
9.45e-154
0
0.000154
0.000208
0.0996
0.0173
5.25e-63
4.25e-71
6.99e-05
0.167
4.33e-86
ENRAD- (DHT = 10 nM / ATRA = 0 nM)
ENRA-A (DHT = 0 nM / ATRA = 16 nM)
ENRADA (DHT = 10 nM / ATRA = 16 nM)
Ces2b
Adh7
Aldh1a1
Rara
Rarb
Rarg
Retinoid metabolism
Retinoid receptors
C
ATRA
0 nM
6 nM
16 nM
100 nM
C57BL/6J
CD1
D
ENRA-- B27 plus (ATRA = 16 nM)
- + + DHT (10 nM)
- - + Enzalutamide (10 μM)

 © The Author(s)

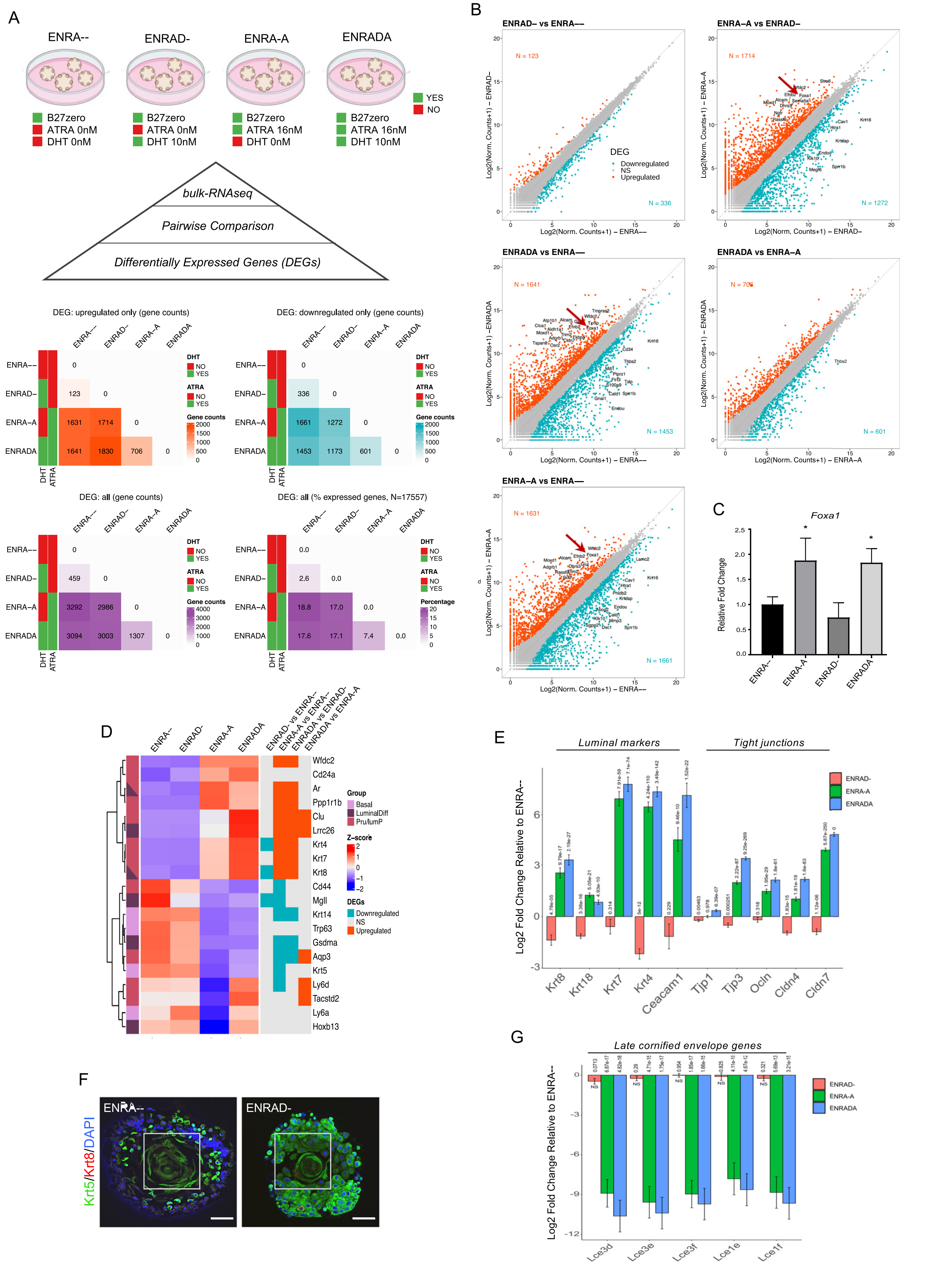
A
ENRA--
ENRAD-
ENRA-A
ENRADA
B27zero
ATRA 0nM
DHT 0nM
B27zero
ATRA 0nM
DHT 10nM
B27zero
ATRA 16nM
DHT 0nM
B27zero
ATRA 16nM
DHT 10nM
YES
NO
bulk-RNAseq
Pairwise Comparison
Differentially Expressed Genes (DEGs)
DEG: upregulated only (gene counts)
DEG: downregulated only (gene counts)
DEG: all (gene counts)
DEG: all (% expressed genes, N=17557)
DHT
ATRA
Gene counts
Percentage
B
ENRAD– vs ENRA––
ENRA–A vs ENRAD–
ENRADA vs ENRA––
ENRADA vs ENRA–A
ENRA–A vs ENRA––
N = 123
N = 336
N = 1714
N = 1272
N = 1641
N = 1453
N = 601
N = 1631
N = 1661
DEG
Downregulated
NS
Upregulated
Log2(Norm. Counts+1) – ENRA––
Log2(Norm. Counts+1) – ENRAD–
Log2(Norm. Counts+1) – ENRA–A
C
Foxa1
Relative Fold Change
D
Group
Basal
LuminalDiff
Pru/lumP
Z-score
DEGs
Downregulated
NS
Upregulated
Wfdc2
Cd24a
Ar
Ppp1r1b
Clu
Lrrc26
Krt4
Krt7
Krt8
Cd44
Mgll
Krt14
Trp63
Gsdma
Aqp3
Krt5
Ly6d
Tacstd2
Ly6a
Hoxb13
E
Luminal markers
Tight junctions
Log2 Fold Change Relative to ENRA--
Krt8
Krt18
Krt7
Krt4
Ceacam1
Tjp1
Tjp3
Ocln
Cldn4
Cldn7
ENRAD-
ENRA-A
ENRADA
F
Krt5/Krt8/DAPI
ENRA--
ENRAD-
G
Late cornified envelope genes
Log2 Fold Change Relative to ENRA--
Lce3d
Lce3e
Lce3f
Lce1e
Lce1f

© The Author(s)

Figure EV2. Transcriptional and phenotypic impact of ATRA treatment in prostate organoids.

(**A**) Schematic representation of the cross-comparison study of RNA-seq analysis performed in this study (upper panels) and heatmaps displaying the number of DEGs (upregulated, downregulated, and total) for the 6 comparisons between the experimental conditions (ENRA--, ENRAD-, ENRA-A, ENRADA) (lower panels). The number indicated inside each cell of the matrix is a gene count except for the bottom right heatmap, which shows the percentage of DEGs over the total number of expressed genes ($N = 17{,}557$). Each cell number is the result of the differential expression analysis between the condition indicated in the row and the one in the column (e.g., 1631 is the number of upregulated genes in the comparison ENRA-A vs ENRA--). Upregulated ($\log2FC > 1$) and downregulated ($\log2FC < -1$) genes are the ones with an adj. *p*-value lower than 0.05 (Wald test followed by the Benjamini–Hochberg multiple test correction, default in DESeq2). (**B**) Scatter plots representing the changes in gene expressions in mPrOs grown for 6 days in indicated media. The number of significant up ($\log2FC > 1$, orange) and down ($\log2FC < -1$, light blue) regulated genes is indicated as N in the figure. Significance is assigned if the gene has an adj. *p*-value lower than 0.05 (Wald test followed by the Benjamini–Hochberg multiple test correction, default in DESeq2). Red arrow indicates Foxa1. (**C**) RT-qPCR analysis of Foxa1 gene expression in mPrOs keept for 5 day in ENRA-- and treated for 24 h with DHT, ATRA or the combination of both (Data are presented as mean value ± s.d. of $n = 3$ independent biological replicates, one-way ANOVA $^{*}p = 0.022$). (**D**) Heatmap showing the expression of a selected panel of genes in mPrOs kept in the indicated culture conditions (mean of $n = 3$ biological independent replicates). Hierarchical clustering with average method has been applied on the heatmap rows. Genes are annotated as basal, luminal differentiated, and periurethral (PrU)/luminal progenitor (LumP) based on Crowley et al (2020) single-cell RNA sequencing analysis. Significant differentially expressed genes (DEGs) in the different comparisons are shown in red (upregulated) and turquoise (downregulated). Significance is assigned if the gene has an adj. *p*-value lower than 0.05 (Wald test followed by the Benjamini–Hochberg multiple test correction, default in DESeq2). (**E**) Differential expression of luminal marker and tight-junction genes in mPrOs grown under ENRADA, ENRA-A, ENRAD-, or ENRA-- culture conditions. Data are presented as mean value ± s.d. of $n = 3$ independent biological replicates. The indicated adjusted *p*-values were calculated with the Wald test followed by the Benjamini–Hochberg multiple test correction (default in DESeq2). (**F**) Immunofluorescence analysis of Krt5 and Krt8 in mPrOs cultured without DHT and ATRA (ENRA--) or with DHT only (ENRAD). The withe frame marks a peculiar cell morphology noticed only in the absence of ATRA. Scale bars, 100 µm. (**G**) RNA-seq bar plot representation of late cornified envelope genes (*LCE*) the expression of which is robustly repressed by RA signaling. Data are presented as mean value ± s.d. of $n = 3$ independent biological replicates. The indicated adjusted *p*-values were calculated with the Wald test followed by the Benjamini–Hochberg multiple test correction (default in DESeq2).

 © The Author(s)

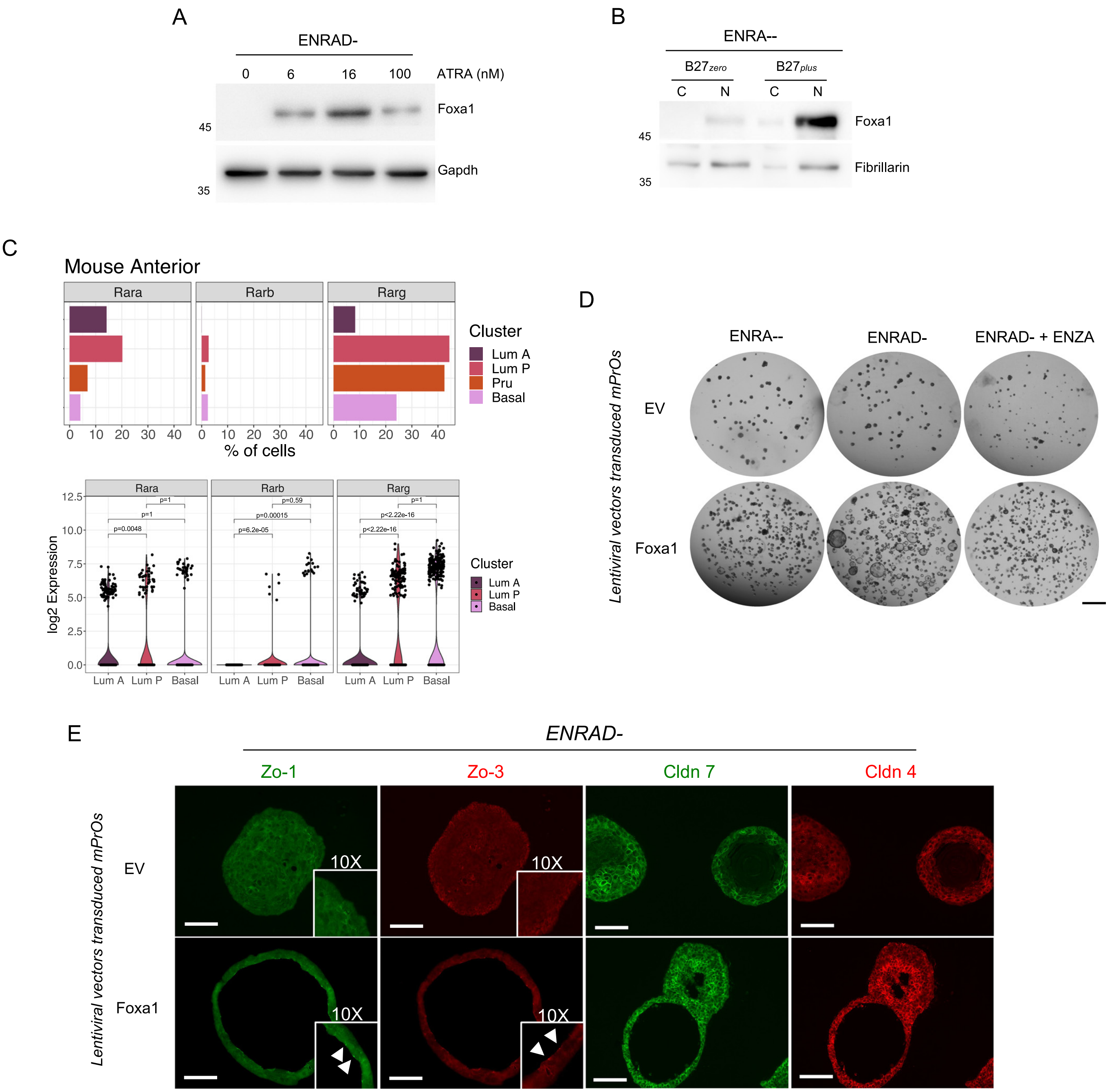

Figure EV3. Molecular and phenotypic impact of modulating RA signaling, AR signaling, and Foxa1 expression in prostate organoids.

(**A**) Western blot analysis of endogenous Foxa1 expression in mPrOs treated with different amounts of ATRA. Gapdh is used as loading control. $N = 2$ independent biological replicates. (**B**) Biochemical fractionation of cytosolic (C) and nuclear (N) compartments showing levels and localization of endogenous Foxa1 in the absence ($B27_{zero}$, ATRA 0 nM) or presence ($B27_{plus}$, ATRA 16 nM) of RA signaling. Fibrillarin is used as nuclear marker and loading control. (**C**) Percentage of cells (bar plots) and expression levels (violin plots) of, *Rarα Rarβ*, and *Rarγ* genes in epithelial cell populations of mouse normal prostate (Data ref: Crowley et al, 2020; Appendix Fig. S2). The *p*-values indicated in the boxplots were calculated with the Mann–Whitney U Test. (**D**) Morphological analysis of transduced mPrOs ((empty vector (EV) and Foxa1)) cultured without ATRA and DHT (ENRA--), without ATRA with DHT (ENRAD-), and without ATRA with DHT plus Enzalutamide (ENZA 10 μM). Scale bar, 1 mm. (**E**) Immunofluorescence analysis of Zo-1 (*Tjp1*), Zo-3 (*Tjp3*), Cldn 4, and Cldn 7 expression and localization in transduced mPrOs (EV and Foxa1) cultured without ATRA but with DHT (ENRAD-). Magnification (10x) of Zo-1 and Zo-3 immunostaining are shown to pointing out protein localization. Nuclei are stained with DAPI. Scale bars, 100 μm. White arrowhead indicates Zo-1 and Zo-3 proteins localization. $N = 2$ independent biological replicates.

© The Author(s)

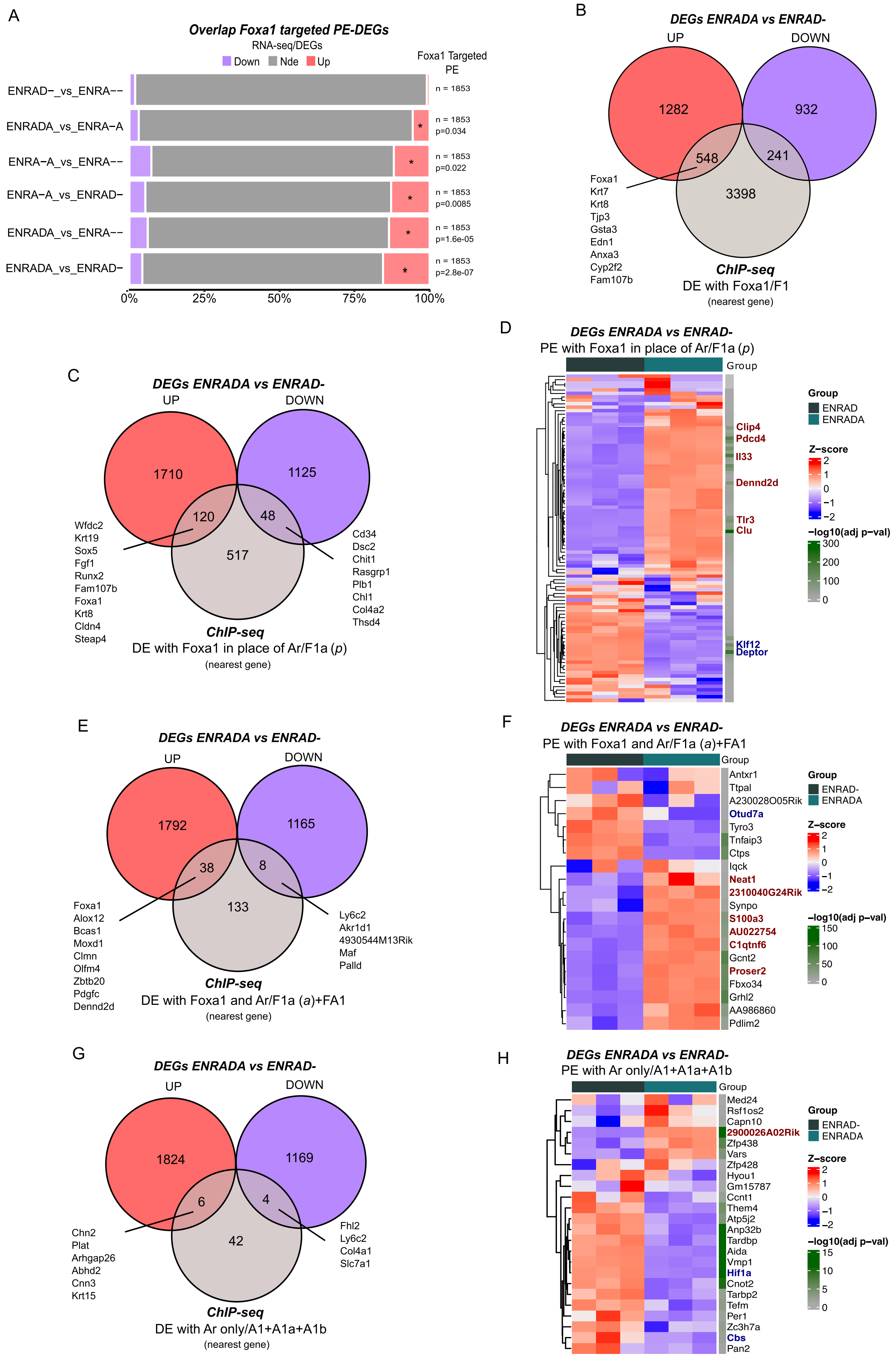
A
Overlap Foxa1 targeted PE-DEGs
RNA-seq/DEGs
Down Nde Up
Foxa1 Targeted PE
ENRAD−_vs_ENRA−− n = 1853
ENRADA_vs_ENRA−A n = 1853 p=0.034
ENRA−A_vs_ENRA−− n = 1853 p=0.022
ENRA−A_vs_ENRAD− n = 1853 p=0.0085
ENRADA_vs_ENRA−− n = 1853 p=1.6e-05
ENRADA_vs_ENRAD− n = 1853 p=2.8e-07
0% 25% 50% 75% 100%
B
DEGs ENRADA vs ENRAD-
UP DOWN
1282 932 548 241 3398
Foxa1 Krt7 Krt8 Tjp3 Gsta3 Edn1 Anxa3 Cyp2f2 Fam107b
ChIP-seq
DE with Foxa1/F1
(nearest gene)
C
DEGs ENRADA vs ENRAD-
UP DOWN
1710 1125 120 48 517
Wfdc2 Krt19 Sox5 Fgf1 Runx2 Fam107b Foxa1 Krt8 Cldn4 Steap4
Cd34 Dsc2 Chit1 Rasgrp1 Plb1 Chl1 Col4a2 Thsd4
ChIP-seq
DE with Foxa1 in place of Ar/F1a (p)
(nearest gene)
D
DEGs ENRADA vs ENRAD-
PE with Foxa1 in place of Ar/F1a (p)
Group
Clip4 Pdcd4 Il33 Dennd2d Tlr3 Clu Klf12 Deptor
Group ENRAD ENRADA
Z-score 2 1 0 −1 −2
−log10(adj p−val) 300 200 100 0
E
DEGs ENRADA vs ENRAD-
UP DOWN
1792 1165 38 8 133
Foxa1 Alox12 Bcas1 Moxd1 Clmn Olfm4 Zbtb20 Pdgfc Dennd2d
Ly6c2 Akr1d1 4930544M13Rik Maf Palld
ChIP-seq
DE with Foxa1 and Ar/F1a (a)+FA1
(nearest gene)
F
DEGs ENRADA vs ENRAD-
PE with Foxa1 and Ar/F1a (a)+FA1
Group
Antxr1 Ttpal A230028O05Rik Otud7a Tyro3 Tnfaip3 Ctps Iqck Neat1 2310040G24Rik Synpo S100a3 AU022754 C1qtnf6 Gcnt2 Proser2 Fbxo34 Grhl2 AA986860 Pdlim2
Group ENRAD- ENRADA
Z-score 2 1 0 −1 −2
−log10(adj p−val) 150 100 50 0
G
DEGs ENRADA vs ENRAD-
UP DOWN
1824 1169 6 4 42
Chn2 Plat Arhgap26 Abhd2 Cnn3 Krt15
Fhl2 Ly6c2 Col4a1 Slc7a1
ChIP-seq
DE with Ar only/A1+A1a+A1b
(nearest gene)
H
DEGs ENRADA vs ENRAD-
PE with Ar only/A1+A1a+A1b
Group
Med24 Rsf1os2 Capn10 2900026A02Rik Zfp438 Vars Zfp428 Hyou1 Gm15787 Ccnt1 Them4 Atp5j2 Anp32b Tardbp Aida Vmp1 Hif1a Cnot2 Tarbp2 Tefm Per1 Zc3h7a Cbs Pan2
Group ENRAD- ENRADA
Z-score 2 1 0 −1 −2
−log10(adj p−val) 15 10 5 0

 © The Author(s)

Figure EV4. Transcriptional impact of retinoic acid and testosterone signaling in prostate organoids.

(**A**) The barplot displays the overlap between differentially expressed genes (this work, comparison indicated on the left side) and all the Foxa1 PE-associated genes from ChIP-seq in the Foxa1 transgene expression condition (F1 + F1a + F1b + FA1). Genes are colored based on the RNA-seq status, i.e., upregulated (red), Downregulated (purple), or not differentially expressed (gray). The total number of FOXA1 PE-associated genes is 1853. The significance of the overlap between up- and down-regulated genes and the FOXA1 PE-associated genes has been determined by a hypergeometric test. The *p*-value is denoted by asterisks (***: 0–0.001, **: 0.001–0.01, *: 0.01–0.05, No symbol: 0.1–1.0). ChIP-seq data are from Adams et al, 2019. (**B**) Venn diagrams showing the overlap of differentially expressed genes in mPrOs cultured in ENRADA versus ENRAD, and exogenous Foxa1-bound distal elements (DE (F1)) in the genome. Relevant upregulated genes in the intersection are highlighted. (**C**) Venn diagram showing the overlap between DEGs in mPrOs cultured in ENRADA versus ENRAD and distal elements where exogenous Foxa1 replaces/displaces Ar. Relevant DEGs in the intersections are highlighted. (**D**) Heatmap showing DEGs in mPrOs cultured in ENRADA versus ENRAD on the promoter of which exogenous Foxa1 displaces/replaces Ar. The indicated adjusted *p*-values were calculated with the Wald test and then corrected with the Benjamini–Hochberg method (default method in DESeq2). Gene names highlighted in red indicate a significant upregulation (log2FC > 1, adj. *p*-value < 0.05), while gene names highlighted in blue indicate a significant downregulation (log2FC < −1, adj. *p*-value < 0.05). (**E**) Venn diagram showing the overlap between differentially expressed genes in mPrOs cultured in ENRADA versus ENRAD and distal elements concomitantly bound by both Ar and exogenous Foxa1. Relevant genes in the intersections are highlighted. (**F**) Heatmap showing DEGs in mPrOs cultured in ENRADA versus ENRAD whose promoter is concomitantly bound by both Ar and exogenous Foxa1. The indicated adjusted *p*-values were calculated with the Wald test and then corrected with the Benjamini–Hochberg method (default method in DESeq2). Gene names highlighted in red indicate a significant upregulation (log2FC > 1, adj. *p*-value < 0.05), while gene names highlighted in blue indicate a significant downregulation (log2FC < −1, adj. *p*-value < 0.05). (**G**) Venn diagram showing the overlap between DEGs in mPrOs cultured in ENRADA versus ENRAD and distal elements bound by Ar but not by exogenous Foxa1. Relevant genes in the intersections are highlighted. (**H**) Heatmap showing DEGs in mPrOs cultured in ENRADA versus ENRAD whose promoter is bound by Ar but not by exogenous Foxa1. The indicated adjusted *p*-values were calculated with the Wald test and then corrected with the Benjamini–Hochberg method (default method in DESeq2). Gene names highlighted in red indicate a significant upregulation (log2FC > 1, adj. *p*-value < 0.05), while gene names highlighted in blue indicate a significant downregulation (log2FC < −1, adj. *p*-value < 0.05).

© The Author(s)

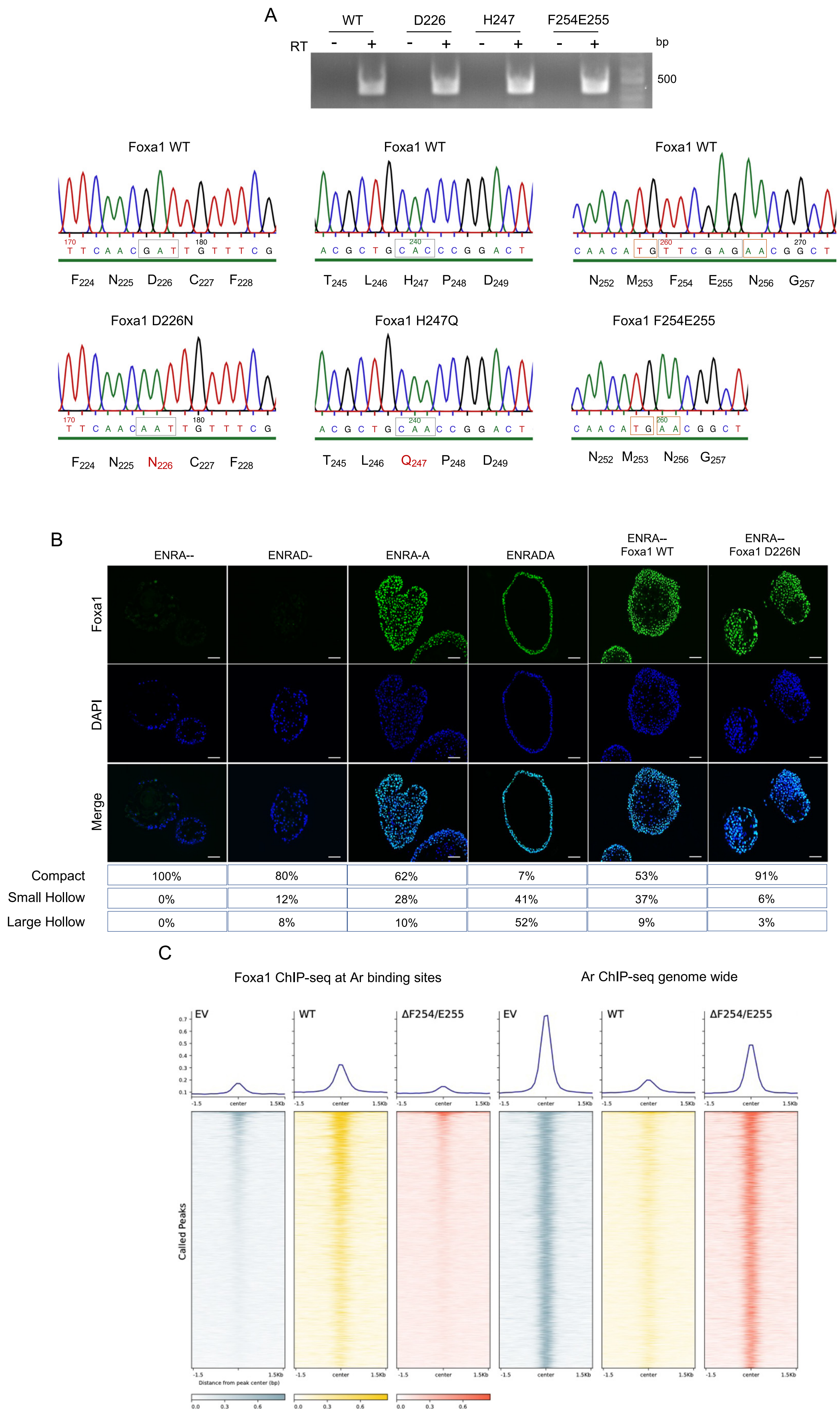
A
WT
D226
H247
F254E255
RT
bp
500
Foxa1 WT
Foxa1 D226N
Foxa1 H247Q
Foxa1 F254E255
F224 N225 D226 C227 F228
T245 L246 H247 P248 D249
N252 M253 F254 E255 N256 G257
F224 N225 N226 C227 F228
T245 L246 Q247 P248 D249
N252 M253 N256 G257
B
ENRA--
ENRAD-
ENRA-A
ENRADA
ENRA-- Foxa1 WT
ENRA-- Foxa1 D226N
Foxa1
DAPI
Merge
Compact 100% 80% 62% 7% 53% 91%
Small Hollow 0% 12% 28% 41% 37% 6%
Large Hollow 0% 8% 10% 52% 9% 3%
C
Foxa1 ChIP-seq at Ar binding sites
Ar ChIP-seq genome wide
EV
WT
ΔF254/E255
Called Peaks
Distance from peak center (bp)

Figure EV5. Genetic engineering of prostate organoids with Foxa1 mutant isoforms.

(**A**) RT-PCR and amplicons sequences of wild-type and mutant forms of Foxa1 stably expressed in mPrOs. Spectropherograms highlighting the mutated nucleotides in the different mPrOs lines. (**B**) Immunofluorescence analysis showing nuclear localization of endogenous and exogenous wild-type and mutant D226N Foxa1 in different growth culture conditions. Scale bar 50 µm. (**C**) Heatmap showing the signal intensity of Foxa1 and Ar binding over AR genome-wide binding sites (ChIP-seq from Adams et al, 2019). ChIP-seq was performed on mPrOs stably transduced with wild-type Foxa1, $Foxa1^{F254E255}$, or the empty vector (EV) and cultured with DHT but not ATRA.
